# Dynamic interplay between niche variation and flight adaptability drove a hundred million years’ dispersion in iconic lacewings

**DOI:** 10.1073/pnas.2414549122

**Published:** 2025-05-02

**Authors:** Haohong Ou, Jingtao Yang, Honglong Wang, Nuoyao Kang, Shumin Li, Yuting Chen, Zihao Peng, Xianzhe Xiang, Michael S. Engel, Shaun L. Winterton, Dong Ren, Qiang Yang, Chaofan Shi

**Affiliations:** ^a^School of Earth Sciences and Engineering, Guangdong Provincial Key Lab of Geological Processes and Mineral Resources, Sun Yat-sen University, Guangzhou 510275, China; ^b^School of Life Sciences, Key Laboratory of Conservation and Application in Biodiversity of South China, Guangzhou University, Guangzhou 510006, China; ^c^Division of Invertebrate Zoology, American Museum of Natural History, New York, NY 10024-5192; ^d^California State Collection of Arthropods, California Department of Food and Agriculture, Sacramento, CA 95832-1448; ^e^College of Life Sciences and Academy for Multidisciplinary Studies, Capital Normal University, Beijing 100048, China

**Keywords:** climate change, biogeography, deep time, niche evolution, flight aerodynamics

## Abstract

The form and change of animal biogeography reflects the long-term interplay between organisms and their environment, involving physiological limitation, dispersal capability, and adaptive evolution versus plate tectonics, global climatic shifts, and changing landscapes. This is especially manifest for lineages with extended geological histories, which, therefore, evokes questions as to the associated processes producing such patterns. Insects, as the earliest flying animals, have exceptional abilities for expanding their range and habitats and to avoid detrimental conditions. They are ideal for exploring historical biogeography augmented via adaptation. Here, we employ beaded lacewings as a model to explore such patterns and likely processes, particularly given that they differ notably from the commonly observed pattern of a latitudinal diversity gradient. Furthermore, owing to their good fossil record it can be observed that their distributions varied remarkably through time. Ecological niche modeling and evaluation demonstrate their niche variation and niche breadth expansion intermittently accompanying global climate change. However, different niche relevant variables changed under patterns of either phylogenetic conservatism or evolutionary lability. By assessing wing morphological disparity and modeling flight aerodynamics, we uncovered a continuous improvement of flight efficiency through beaded-lacewing history as well as a Paleogene divergence in strategy, which reveals a long-term associated path with the niche variation. Our results unveil the adaptive evolution and dispersal history of beaded lacewings through 170 My, achieved by dynamic strategies in niche shift and flight adaptation as responses to a changing planet.

## Significance

The distribution of organisms throughout the world has been a subject of intense exploration since the seminal work of Alfred Russel Wallace. The intertwined roles among niche specializations, physiological and behavioral adaptations, phylogenetic history, and climate changes through deep time are key to understanding patterns and processes of evolution, as well as predicting future responses to change. We use the iconic Berothidae to test their dynamic interplay with the environment over vast spans of geological time. Deploying niche modeling, paleoclimates, phylogeny, and flight aerodynamics, we found that berothids’ niches have evolved alongside improvements in flight capability over the course of 170 My. The result of these changes permitted global reorganization of their distribution, reflecting the aforementioned complex interactions.

The geographical distribution of terrestrial animals is shaped by the interplay between adaptability and dispersal (1, 2). The biogeographic pattern of lineages as well as their dispersal routes can be constrained by global or regional environmental variation and biological tolerance limits, while, conversely, driven by adaptive changes in physiology or improvement of dispersal strength (3–7). At a geochronologic scale, the tectonic history of Earth and the historical courses of phylogenetic diversification are backstage forces leading to present patterns (8–12). Collectively, biogeographic occurrences result from long-term intricate interactions involving numerous varying factors, reflecting the response of organisms to the environment via adaptation in physiology and/or functional morphology.

**Fig. 1.**
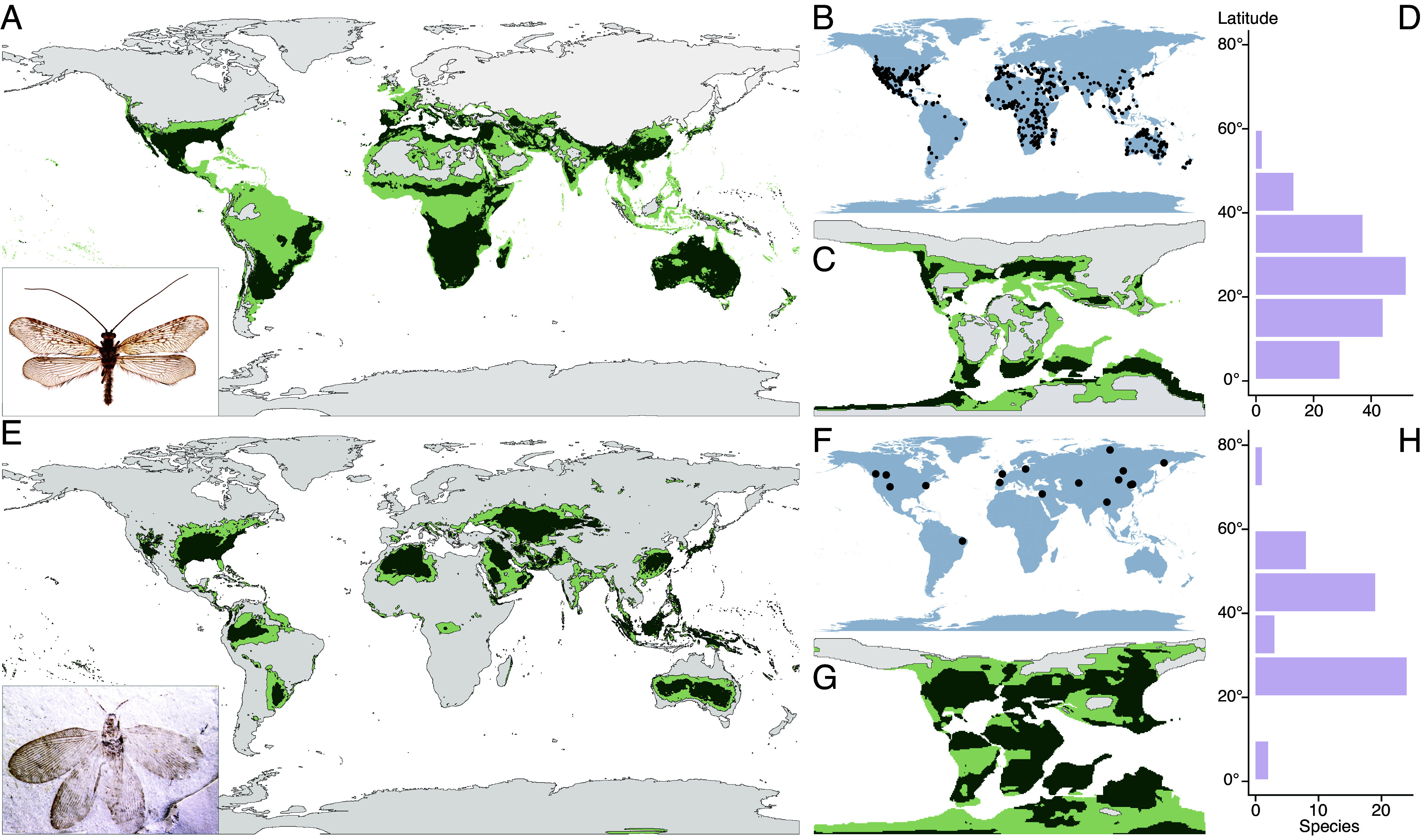
Beaded lacewings’ climate niches have shifted in parallel with 170 My of global climate change. (*A*) ENM of extant berothids. (*B*) Distribution of extant species. (*C*) ENM of extant berothids projected on paleogeography and paleoclimate at 100 Ma. (*D*) Absolute latitudinal distribution of extant species. (*E*) ENM of all fossil berothids projected on modern Earth and climate conditions. (*F*) Distribution of fossil species. (*G*) ENM of all fossil berothids projected on paleogeography and paleoclimate at 100 Ma. (*H*) Absolute latitudinal distribution of fossil species. The dark green regions in (*A*, *C*, *E*, and *G*) indicate suitable habitats (*P* > 0.5); light green regions indicate less suitable habitats (0.3 < *P* < 0.5); gray regions indicate unsuitable areas (*P* < 0.3). The horizontal axis in (*D* and *H*) implies species number, and the vertical axis implies absolute latitude bin. *Inset* image in (*A*) the extant *Trichoma gracilipenne* (photo: Yun Li); in (*E*) the Middle Jurassic *Sinosmylites rasnitsyni* (photo: Qiang Yang).

Flying animals exhibit typically superior dispersal ability, permitting them to surmount geographic barriers or surface obstacles (13–15). Insects, as the first flyers in Earth’s history (16, 17), have developed more varieties of dispersal means and spread over all continents and innumerable habitats during their evolutionary history (1, 18–20). Given the vast diversity and extensive history of insects, it is no surprise that some of the first explorations into the interaction between changing climates and shifting distributions were carried out through a study of their fossil record. As early as 1836, the Rev. Frederick Hope highlighted the occurrence of insects preserved in amber as keys to reconstructing the paleoclimate of Europe, emphasizing that the insects were wholly “extra-European” and demonstrated that the global climate must have undergone considerable change (21). The seminal works of Alfred R. Wallace in the Malay Archipelago demonstrated the contribution of insect and other animal distributions for discovering complex paleogeographic histories, patterns later revealed to derive from geologic changes in elevation, ocean depth, glaciation, as well as Earth climate (22, 23). Indeed, insects serve as an ideal model for exploring the intimate history of specialization and historical occurrence against the backdrop of phylogeny and global climatic evolution. The beaded lacewings (Berothidae) are a family of neuropteran insects, with a comparatively rich fossil history extending back to the Jurassic (24–26). Extant beaded lacewings are distributed across all modern zoogeographic realms (27, 28), while the extinct faunas, although also widely ranged, are greatly distinct from extant patterns of distribution (Fig. 1). Furthermore, beaded lacewings exhibit a great range of wingspans and divergent wing shapes, which is even dramatic among species from different geological periods. Having experienced the break-up of the supercontinent Pangaea (29, 30) and extensive global climate shifts (31, 32), beaded lacewings waxed and waned in species diversity and extent of distribution, and eventually radiated successfully in comparatively modern history. The rhythmic coevolution between global environment and beaded lacewings makes them an iconic case to test hypotheses regarding insect acclimatization and behavior adaptation through time.

Here, we reconstructed the historical biogeography of beaded lacewings based on a time-calibrated phylogeny encompassing nearly all fossil and extant genera. In addition, by integrating datasets of high-resolution paleogeography and climate variables of global and regional scales, with assessment of ecological niche variation and flight aerodynamic optimization using interdisciplinary analyses, the deep drivers for their dispersal routes over 170 My were explored. Our results reveal that the family underwent two distinct events of biogeographic dispersal simultaneous with species diversification during the Early Cretaceous and late Paleogene–Neogene. From the perspective of the environment, both events accompanied phases of global cooling. Plate tectonics played essential roles especially during the Neogene. In terms of biology, the first event was mainly associated with the expansion of ecological niches while the latter event aligned with enhancement and specializations in flight efficiency. The dispersal history of beaded lacewings unveils a multifactored coevolving course between the physical environment and the insects themselves, which constitutes a valuable reference for further exploration into the mechanism and dynamics of interactions between Earth systems and organismal change at the geochronologic scale.

## Results and Discussion

### Biogeography and Niche Shift through Geological History.

Berothidae have extended distributions, which exhibit a pronounced latitudinal conditioned pattern (Fig. 1). Extant beaded lacewings mostly occur between 20° and 40° with fewer species and individuals in the tropical region and poleward extending to 50° (Fig. 1 *B* and *D*). In contrast, fossil species are mostly recorded between 20° and 50°, poleward extending to 71° (Fig. 1 *F* and *H* and *SI Appendix*, Fig. S1). Noticeably, extant berothid distributions are distinguishable from the more common pattern of a latitudinal diversity gradient (33–37), and the same holds for the extinct faunas, which implies a unique environmental predilection for the family. Besides, fossil deposits reveal a higher latitudinal tendency than is observed among extant species. This could result from tectonics and consequent paleogeographic shifts (38, 39). However, by converting the fossil deposits to paleocoordinates of the corresponding stratigraphic age, the paleolatitudinal distribution of fossil species is still dissimilar to their extant counterparts (*SI Appendix*, Fig. S2). Knowing that fossil berothids are largely from the Mesozoic, when global land surface temperature was significantly higher than at present (31, 32), the discrepant patterns of distribution between fossil and extant beaded lacewings could be attributable to global climate change or alternatively to ecological niche variation of the family (40–43).

By extracting the explicit paleoclimate variables of the fossil berothid localities according to their corresponding paleocoordinates as well as modern climate variables of the extant species, we modeled their climatic niches and suitable habitats separately for the extinct and extant records. The modeled niche conditions were mapped on modern and deep-time geography based on respective climatic variables. Taking the mid-Cretaceous, 100 Ma, as a representative of a deep-time situation, comparison of the niche modeling between extinct and extant berothids reveals remarkable differences, either on the modern (Fig. 1 *A* and *E*) or Cretaceous climate conditions (Fig. 1 *C* and *G*), which suggests that the climatic niches of berothids have changed through time. Considering the extended chronologic range of the fossil records, niches of Mesozoic-only beaded lacewings were also modeled, which drew similar results to the all-fossil models (*SI Appendix*, Fig. S3). The analyses reveal that not only did Berothidae disperse distantly over the course of their evolutionary history accompanying global climate change, but also their own niches have varied significantly. The models also demonstrate that the niches of extant berothids have greater habitable ranges on modern Earth (Fig. 1*A*), while the niches of fossil berothids possess larger habitable ranges on the Mesozoic Earth (Fig. 1*G* and *SI Appendix*, Fig. S3) though the fossil niches represent a broader chronologic span, which implies that the niches of beaded lacewings plausibly have been better suited for the global climate of their respective ages. This raises questions concerning the explicit process and dominating factors of their niche variation and its change.

**Fig. 2.**
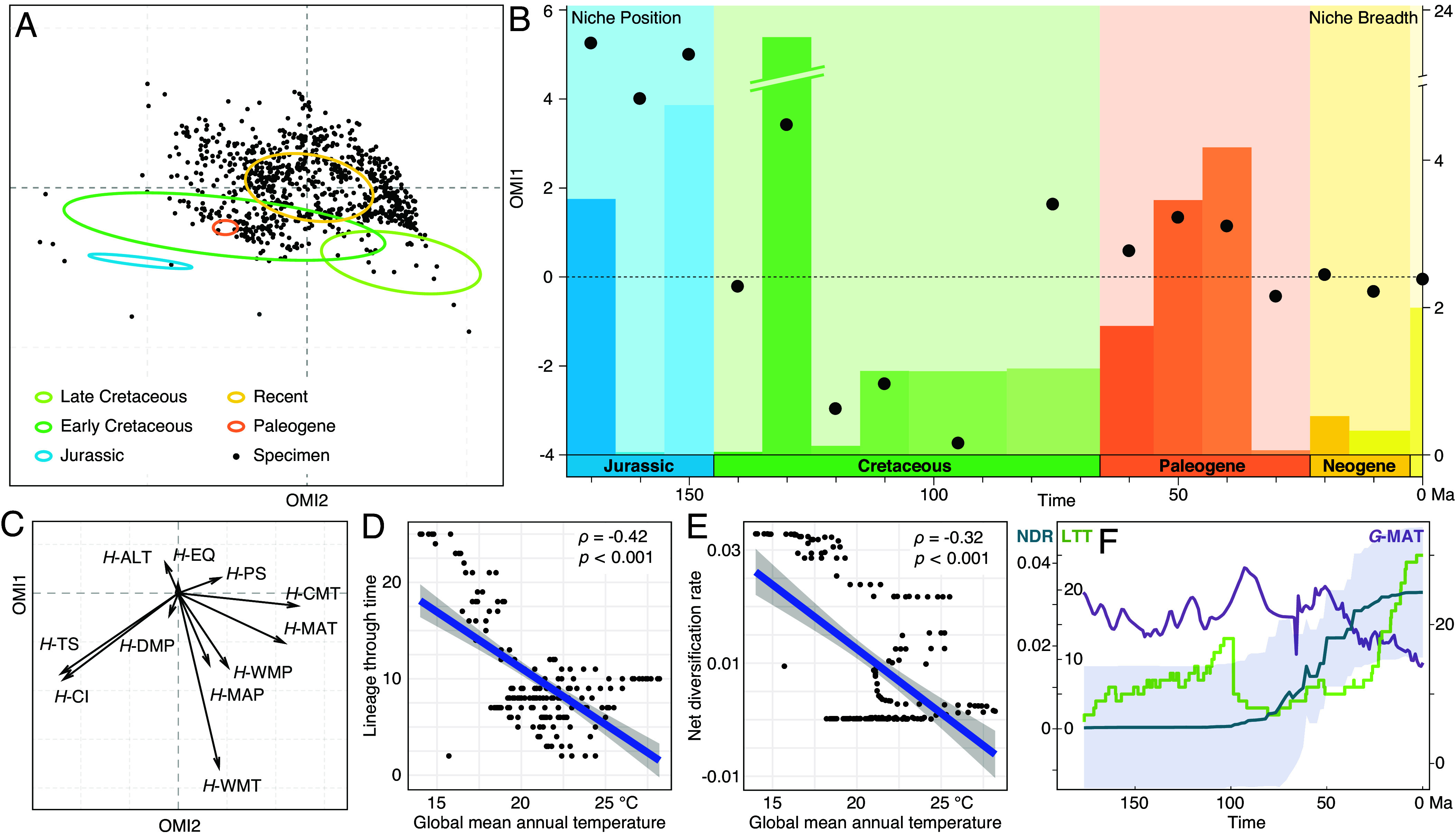
Beaded lacewings responded to global change via niche and diversification variation. (*A*) NPs of all berothid records (dots) and NBs of species divided into five time intervals (colored circles centered to NP centroid of each interval). (*B*) NBs (bars scaled to right vertical axis) and NPs (dots scaled to left vertical axis) in 10-My bins. (*C*) Canonical weights of berothid habitable environmental variables. Correlation analyses (*D*) between *G*-MAT and LTT; (*E*) between *G*-MAT and NDR showing data dots, regression line with 95% CI. (*F*) LTT, NDR with 95% CI and *G*-MAT variation through time.

We assessed niche breadth (NB) and niche position (NP) of Berothidae by incorporating eleven environmental variables (Fig. 2 and *SI Appendix*, Table S1). When integrating all fossil and extant records, the relevant temperature variables of berothid habitat display as main factors affecting their niches (Fig. 2*C*). Though the impact varied with species through geochronologic time, implying their specific sensitivity to climate conditions. The results show the NPs in different geochronological intervals are quite distinct from each other, and therefore have changed intensively through time (Fig. 2 *A* and *B*). Furthermore, their NBs are generally broader in deep time, especially in the Mesozoic, relative to recent (Fig. 2*B* and *SI Appendix*, Fig. S4). Consequently, the changing distribution pattern and the corresponding habitable conditions of berothids through geological history reflect complicated causes behind the process of long-term adaptation, involving external environmental influence and biological variation course.

### Global Change Impact and Responses by Berothidae.

To evaluate the environmental impact on berothids through their evolutionary history, we compiled the global climate change data and implemented causal inference. First, the driving factors for changes of NB and NP were assessed. The results show that NB change could be affected by the global driest month precipitation and warmest month mean temperature variation, while changing NP could be caused by global driest month precipitation and precipitation seasonality (*SI Appendix*, Fig. S5 *A*–*D*). Therefore, global climate change not only influenced the biogeographic distribution of beaded lacewings, but more severely it likely changed their NP and NB. This also accounted for the suitable habitat magnifying in varied periods given the changing climate (Fig. 1).

The effect of global climate change on niche variation could also act on the diversification of the family. To investigate this associated process, we conducted time-calibrated phylogenetic analyses based on an extensive sampling of fossil and extant genera. The analyses recovered Berothidae diverging in the Early Jurassic, around 178.7 Ma (*SI Appendix*, Fig. S6). The family diversified in the Jurassic and Early Cretaceous, and later during the Cenozoic. The two phases of significant radiative evolution respectively correspond to the Late Jurassic–Early Cretaceous Cool Interval and Late Cenozoic Icehouse, implying the insects’ predilection for a cooler climate (31, 44, 45). Derived from the tree, lineage through time (LTT) and net diversification rates (NDR) of Berothidae show subject to global temperature fluctuation, especially global mean annual temperature (*G*-MAT). Both LTT and NDR are significantly correlated with *G*-MAT, both negatively. Their rapid diversification associated with global cooling events combined with the negative correlation of LTT and NDR with *G*-MAT jointly indicate the relatively low temperature predilection by beaded lacewings, which accounts for the antilatitudinal diversity gradient distributing pattern of both fossil and extant species (Figs. 1 *D* and *H* and 2 *D*–*F*). Additionally, causal inference implies that the family NDR could be affected by *G*-MAT (*SI Appendix*, Fig. S5*E*). Global warmest month mean temperature is also found significantly and negatively correlated with LTT, and could be a causal factor to both LTT and NDR (*SI Appendix*, Fig. S5 *F*–*I*), which further indicates their intolerance to high temperature. Indeed, the extreme high temperature effect has been widely reported on insect groups at various levels (46, 47).

### Evolutionary Pathways of Niche Variation.

It is evident that global changes have notably influenced the history of beaded lacewings with regard to both their distribution and their diversity. It is also revealed that the lacewings responded to climate change via niche shifts, basically by means of physiological adaptation. Conceptually, organisms can change their niches via two alternative hypothetical pathways, either phylogenetic niche conservatism or evolutionary lability (48–50). Accordingly, we investigated the historical pattern of niche variation in beaded lacewings concerning their adaptation to specific climate factors in a phylogenetic framework. By synthesizing ancestral-state reconstruction (ASR), phylogenetic signal (PSig), and evolutionary rate (ER), we addressed the niche evolution of the family.

**Fig. 3.**
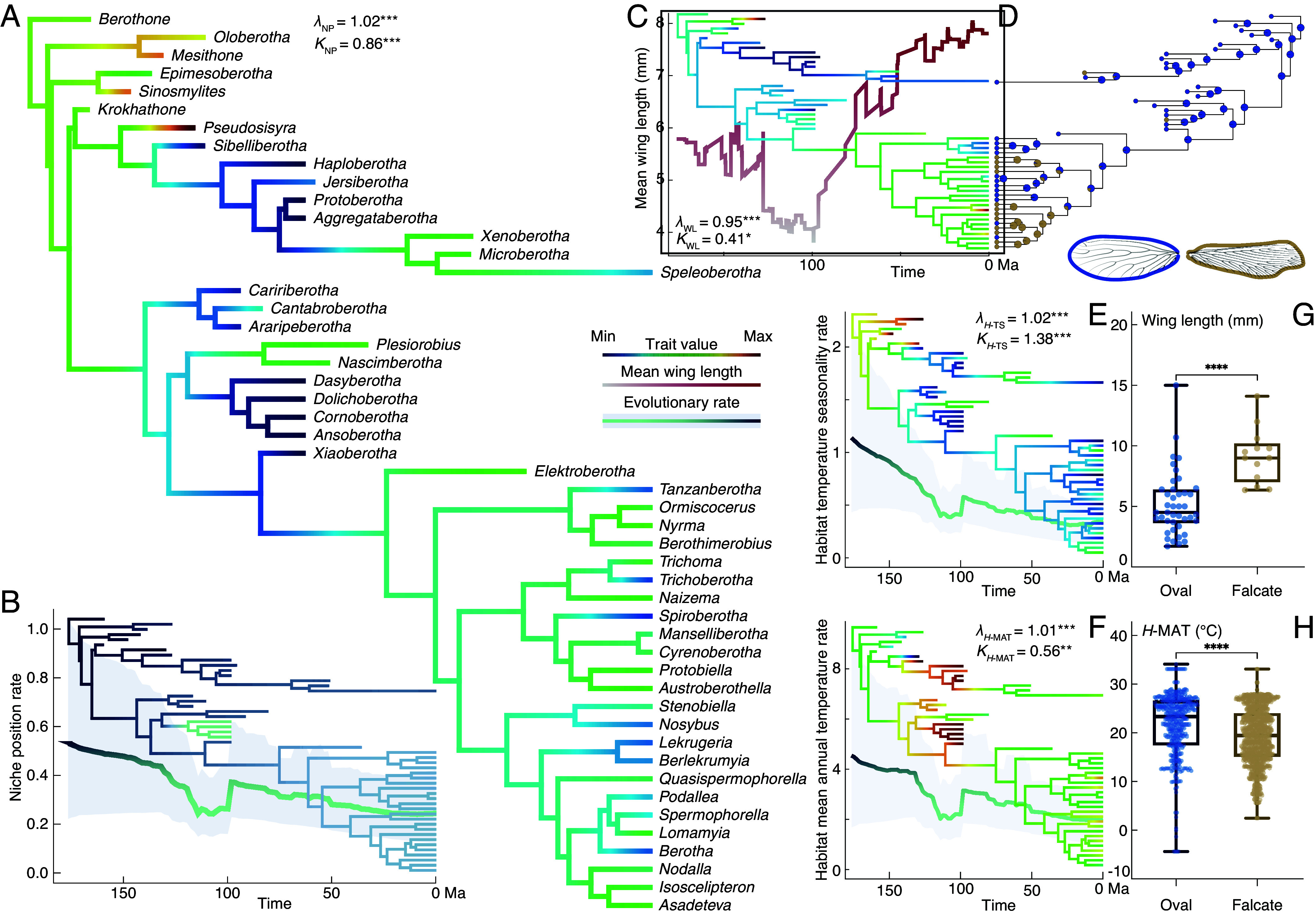
Evolutionary pathways of berothids’ niche variables and morphological traits. (*A*) ASR, PSig, and (*B*) ER across the cladogram and mean rate variation with 95% CI of NP. (*C*) PSig and ASR across the cladogram plotted on mean value variation of WL through time. (*D*) ASR of wing shapes. ASR, PSig, and ER with 95% CI of (*E*) berothid *H*-TS and (*F*) *H*-MAT. Statistical tests of species (*G*) WL and (*H*) *H*-MAT between oval and falcate wing shapes. Box plot shows lower and upper quartiles, median, minimum, and maximum. * indicates significance (*P < 0.05, **P < 0.01, ***P < 0.001, ****P < 0.0001).

The ASR of NP shows substantial similarities within clades (Fig. 3*A*). The states exhibit variation through stages. It reveals a significant PSig of considerable strength (*λ* = 1.02, *P* = 0.0001; *K* = 0.86, *P* = 0.0001). The NP ER indicates a high rate of change in the early stage of the family’s evolution, i.e., the Jurassic. Subsequently, the rate gradually slowed (Fig. 3*B*). We further studied evolutionary traits of the habitat individual climatic factors involving all fossil and extant specimen localities. The results indicate that most relevant temperature factors have significant and relatively strong PSig, the ERs of which were high in the original phase and generally declined through time, similar to the NP (Fig. 3 *E* and *F* and *SI Appendix*, Figs. S7–S9 and Table S2), which is reasonable given that the relevant temperature variables act as main factors of their niches (Fig. 2*C*). Among them, temperature seasonality has significant and exceptionally strong PSig, indicating evident phylogenetic niche conservatism, which is probably related to their seasonal life cycle (51–53) (Fig. 3*E*; *λ* = 1.02, *P* = 0.0001; *K* = 1.38, *P* = 0.0001). Conversely, most relevant precipitation factors have weaker PSig (significant or nonsignificant), the ERs of which did not change extensively (*SI Appendix*, Fig. S9 and Table S2). Our results reveal both phylogenetic niche conservatism and lability present among the evolutionary pathways concerning different relevant climate niche factors, which jointly have shaped the biogeographic pattern of beaded lacewings through time. Nevertheless, it could have also been regulated by other aspects of the insects.

### Dispersal Ability Enhancement by Optimizing Flight Aerodynamic Efficiency.

Beaded lacewings are notable for their distinctly divergent wing shapes and great range of wing lengths (WLs), which could be correlated with their flight adaptability and biogeographic distribution (54, 55). We first investigated the evolutionary traits of their forewing length and shape. It shows that forewing lengths of beaded lacewings varied significantly both in fossil and extant species, ranging from 1.7 to 15 mm. According to results of the ASR and PSig, forewing lengths do not show conservatism within clades. In general, forewing lengths exhibit a trend of elongation (Fig. 3*C*). The analysis recovered a trough around the mid-Cretaceous, but this could be reasonably explained by the abundant small-bodied species reported from Kachin amber and due to the preservational bias of fossil size in amber (25, 56). Similar troughs in this time interval are also found in the ER curves because of the discovery of plentiful species from a single stratum (57) (Fig. 3 *B*, *E*, and *F*).

The wing shape of beaded lacewings can be categorized into oval and falcate types. Oval-shaped wings are common in the family, and especially among fossil taxa. Falcate wings are mainly present in two extant lineages and a few fossil species. The ASR also verified that the oval-shaped wing is the primary state in the family (Fig. 3*D*). By plotting forewing lengths between the two types of wing shapes, a significant difference was uncovered. The length range of the oval type is larger, possibly due to the fact that they are more extensively represented. Nevertheless, most of the oval-shaped wings are notably shorter than the falcate wings. The falcate wings range from 6.4 to 14.1 mm, the minimum of which is still longer than the median and mean of the oval-shaped WLs (Fig. 3*G*). We tested the value distribution of climatic and atmospheric factors between habitats of the oval-shaped and falcate winged beaded lacewings. The results also find significant differences between the shape type habitats, involving habitat mean annual temperature (*H-*MAT), annual precipitation, air density, air viscosity, and airflow velocity (Fig. 3*H* and *SI Appendix*, Fig. S10). However, phylogenetic generalized least squares analyses found WL significantly but weakly correlated with wing shape, while the environmental factors were nonsignificant. Combined with the unconserved evolution of WL, the remarkable differentiation of WL and environmental factors between wing-shape types likely resulted from independent adaptation among the lacewings, reflecting adaptation to certain flight performances rather than shared evolutionary history (*SI Appendix*, Figs. S11 and S12).

**Fig. 4.**
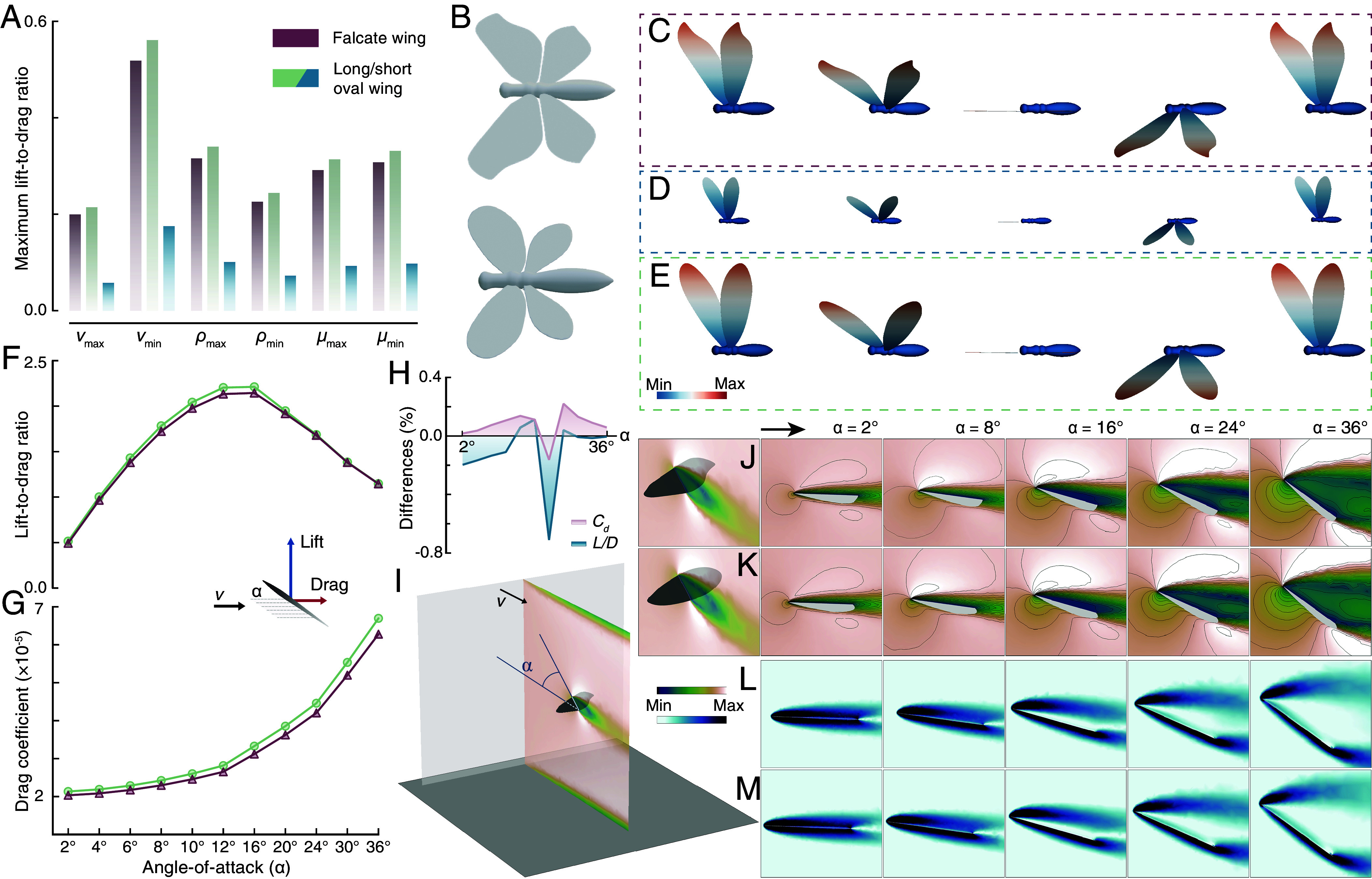
Flight aerodynamic efficiency optimization via wing transformation manifested by CFD modeling. (*A*–*E*) Transient analyses. (*A*) Maximum lift-to-drag ratios of falcate wing of 9 mm long, oval-shaped wings of 9 mm and 4.5 mm, under atmospheric conditions of maximum and minimum airflow velocity (*v*), air density (*ρ*), and air viscosity (*μ*). (*B*) 3D models of the falcate and oval-shaped wing insects. Airflow velocity distribution across the forewing surface of (*C*) falcate wing; (*D*) short oval-shaped wing; (*E*) long oval-shaped wing during a complete flutter process. (*F*–*M*) Steady analyses. (*F*) Lift-to-drag ratios; and (*G*) drag coefficients of falcate and oval-shaped wings of same length under successive angles-of-attack. (*H*) Differences enlargement in drag coefficients (*C_d_*) and lessening in lift-to-drag ratios (*L/D*) between falcate and oval-shaped wings in the lower temperature field compared to higher temperature through varied angles-of-attack. (*I*) Field vision showing angle-of-attack and airflow velocity distribution in cross-section through the wing. Velocity fields of (*J*) falcate wing; (*K*) oval-shaped wing under varied angles-of-attack. Vorticity fields of (*L*) falcate wing; (*M*) oval-shaped wing under varied angles-of-attack.

To address the differences of flight capability driven by WL and shape variation, we modeled the insects three-dimensionally and evaluated the aerodynamic efficiency by computational fluid dynamics (CFD). Considering that flight performance can be affected by atmospheric conditions, particularly air density, air viscosity, and airflow velocity (58, 59), we collected data for each of these features from each fossil and extant species site, and used minimal, maximal, and mean values of each atmospheric factor for different modeling field settings (*SI Appendix*, Table S3). Using the median values of WL of the oval-shaped wing (4.5 mm) and falcate wing (9 mm), transient analyses were performed. The analyses show the maximum lift-to-drag ratio over an entire stroke cycle varied greatly with airflow velocities, less significantly with air densities, and nonsignificantly with air viscosities. For each environmental set, falcate wings display exceptionally higher maximum lift-to-drag ratio than oval-shaped wings, suggesting enhancement of flight aerodynamic efficiency in the falcate wing regardless of atmospheric factor variation (Fig. 4 *A*–*E* and *SI Appendix*, Table S4). To clarify whether the aerodynamic differences were attributed to wing shape or WL, we added another set of modeling with oval-shaped wings of the same length as the falcate wings, since the broader range of WLs of the oval shape. The results show that long oval-shaped wings are generally equal to the falcate wing in maximum lift-to-drag ratio under all atmospheric conditions, and basically even a little higher. The transient analyses suggest that among environmental factors, airflow velocity and air density have negative and positive effects, respectively, on flight aerodynamic efficiency, whereas air viscosity acts negligibly. In the magnitude of berothids’ WLs, longer wings have prominent advantages in lift-to-drag ratio during flight performance, which could be a driving force for wing elongation during the evolution of beaded lacewings (Fig. 3*C*). The significant flight enhancement of longer wings also elucidates the niche-variation history of berothids. During the earlier stage, beaded lacewings with short wings correspond to wide NB and flexibly changing NPs, revealing that niche plasticity compensated for flight and dispersal deficiency in the face of climate change. After the wings generally elongated, with advanced flight capability to track their preferred climates, NB correspondingly narrowed, and the NPs tended to be consistent (Figs. 2*B* and 3*C*). Specifically, the NPs of beaded lacewings show disparate states during the earlier stage, exhibiting a rapid ER (Fig. 3 *A* and *B*). In the latter phase, especially in the Cenozoic, the NP states tend to be convergent, with a lower rate of change.

To further delve into the effect of wing-shape disparity on flight performance, we conducted steady analyses on the oval-shaped and falcate wings of same lengths. Under a series of successive angles-of-attack from 2° to 36°, the two types of wings have the same lift-to-drag ratio for most angles, though similarly, the oval-shaped wings show a slightly higher lift-to-drag ratio than the falcate wings between 10° and 16°. However, the falcate wings show lower drag coefficients especially when the angle-of-attack increases, which indicates improved flexibility and energy efficiency for flight during long-distance dispersal (20, 60) (Fig. 4 *F*–*M* and *SI Appendix*, Fig. S13 and Table S5). Moreover, considering the distinct climatic differentiation between the two wing shape habitats, we tested temperature influence on aerodynamic efficiency between them. Using the median value of either wing shape *H*-MAT, the steady analyses were performed for both wing shapes under the series of angles-of-attack. The results show consensus with the previous analyses, i.e., the falcate wings always show lower drag coefficients and lower lift-to-drag ratio than oval-shaped wings at all angles and temperature settings. However, when the temperature decreases, which is preferred by the falcate-winged beaded lacewings, their advantages of the lower drag coefficients become greater and their disadvantages of the lower lift-to-drag ratio reduce (Fig. 4*H* and *SI Appendix*, Fig. S14 and Table S6). This implies that the two-sided effects of the falcate wings exist in all conditions, but cooler temperature would strengthen their advantages and meanwhile lessen the disadvantages, which clarifies the differentiated *H*-MAT between the wing-shape types.

In general, beaded lacewings evolved longer wings for higher lift-to-drag ratio in all possibilities of environmental conditions, which reflects an improved aerodynamic efficiency and reveals an association with the niche-variation history. During the late phase of their evolutionary history, the wings diverged into two shape types, either of which performs better in regard to lift-to-drag ratio or drag coefficient. These aerodynamic differences can be magnified when local temperature changed, elucidating their environmental discrepancy. The strategy could be interpreted as a trade-off of flight capacities in different lineages.

### Dispersion Interacting with Global Changes through Geological History.

**Fig. 5.**
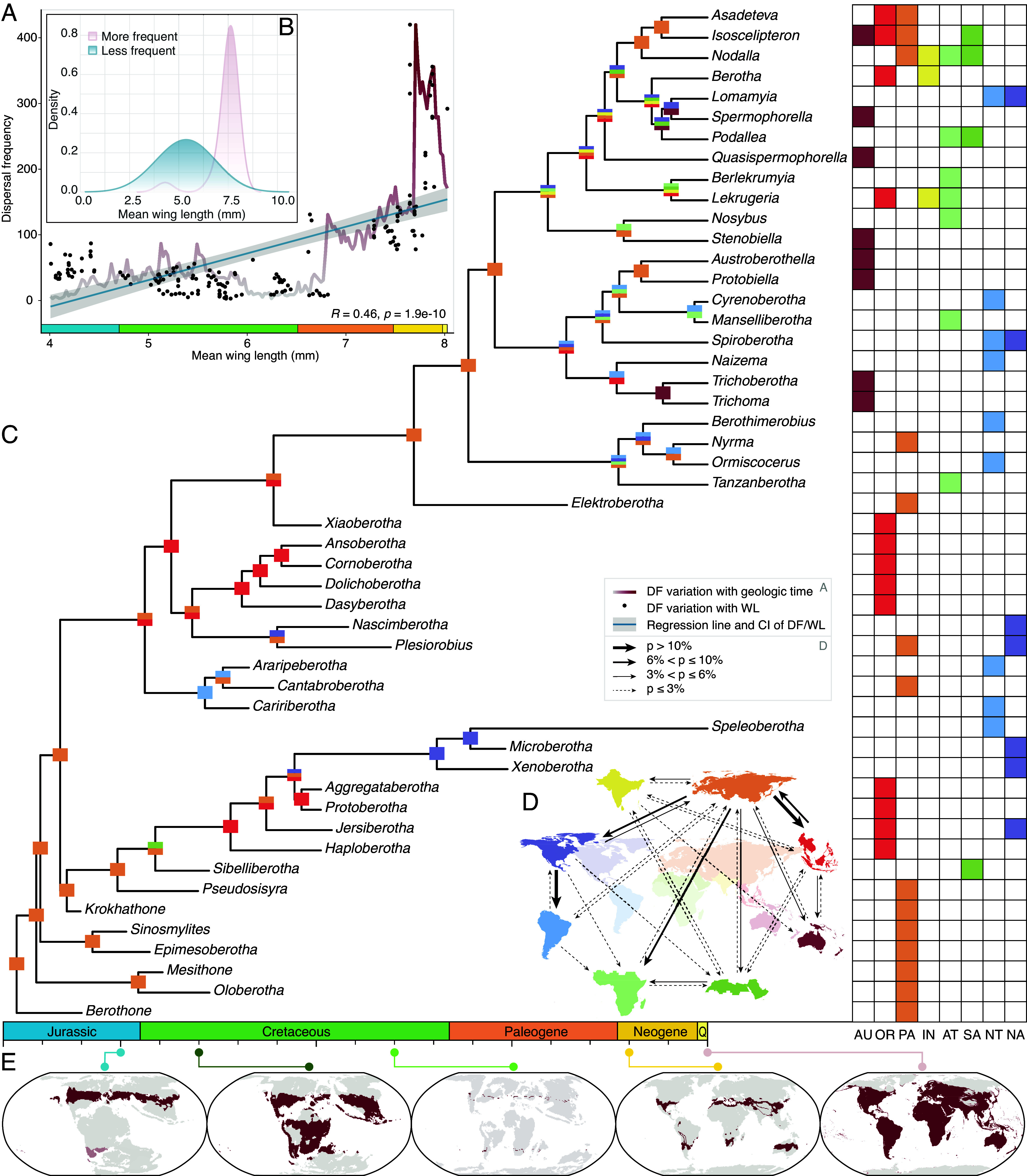
Historical biogeography of Berothidae resulted from variation of dispersal frequency and suitable habitats. (*A*) Dispersal frequency (DF: dispersal events per My) variation with mean WL (mean value per My; dots: raw data; blue line: regression line; gray shade: 95% CI horizontally scaled to mean WL), plotted on dispersal frequency variation through time (red line horizontally scaled to the geological time scale). The vertical axis represents dispersal frequency. (*B*) Probability density distribution of mean WL in two categories of dispersal frequency partitioned by the average number of dispersal events per My. (*C*) Historical biogeography with inferred ancestral realms shown on corresponding nodes and each genus distributed realms shown on the *Right* panel. (*D*) Percentages of dispersal events across biogeographic realms. (*E*) Potential habitats (red), habitable but inaccessible regions (shaded red), and uninhabitable regions (gray) projected by *H*-MAT in successive time intervals. AU: Australian, OR: Oriental, PA: Palearctic, IN: Indian, AT: Afrotropical, SA: Saharo-Arabian, NT: Neotropical, NA: Nearctic.

To track the consequences of all the variations through the geological history of beaded lacewings, we reconstructed their historical biogeography based on the phylogeny and biogeographic distribution of all fossil and extant taxa. The analyses uncovered that the family diverged in the Palearctic (PA) realm (Fig. 5), and that the earliest berothids persisted in the region for nearly 40 My. Along with their first phase of diversification (LTT in Fig. 2*F*), beaded lacewings started long-distance dispersal since the Early Cretaceous, leading to their first expansions in two directions: invading the Oriental (OR) realm for one lineage, and West Gondwana for another, including the modern Saharo-Arabian (SA), Afrotropical (AT), and Neotropical (NT) realms. During the epoch, the insects arrived in the southern hemisphere, as evidenced by the presence of fossil genera in Brazil, and separately, the Cenozoic to present lineage comprising the extinct *Xenoberotha*, *Microberotha*, and the extant *Speleoberotha*. Notably, *Xenoberotha* pioneeringly obtained falcate wings in the early Paleogene. The lowered drag coefficient resulting from the wing shape potentially strengthened individuals of the genus for long-distance dispersal and consequently established a foothold on the American continent.

We extracted and compiled the climatic niche variable threshold of beaded lacewings in each 10-My time bin and mapped them on the corresponding paleogeography. Among all of the environmental factors constituting the climatic niche, the habitable *H-*MAT patterns explained their large-scale dispersal in the first phase (Fig. 5*E*). During the initial 40 My, berothids preferred cooler weather, while global temperature was high, which resulted in the habitable areas for them separately restricted in the midlatitudes of the northern and southern hemispheres (31). Owing to the northern hemispheric origin of the family, during this prolonged span of time with an intolerable band in the tropical latitudes, the southern hemisphere was unattainable for them. Since the Early Cretaceous, their habitable temperature expanded, along with the decrease of global temperature, which provided the opportunity for them to cross the equator for the first time since their divergence (Figs. 2 *B* and *F*, 3*F*, and 5*E*). As a result, genera such as *Sibelliberotha* reached Arabia, and the common ancestor of *Araripeberotha* and *Caririberotha* arrived in South America during the Barremian and Aptian. The first radiative phase ended during the Late Cretaceous. Both lineage diversity and dispersal events dropped sharply, while the *H*-MAT mapping unveils that the habitable area during this interval contracted severely (Fig. 5*E* and *SI Appendix*, Fig. S15).

Beaded lacewings diversified rapidly and spread across the biogeographic realms for the second phase in the Cenozoic. The flight capability improvement via wing elongation and wing shape divergent since the latest Cretaceous (Fig. 3 *C* and *D*), in conjunction with the second episode of expansion in NB during the Paleogene (Fig. 2*B*), provided an impetus for the worldwide dispersal of extant beaded lacewings. Since the Paleogene, the collision of India with Asia made it possible for them to reach India (61–63). The accompanying orogeny of Himalayas also established elevational gradients for niche divergence and species diversification (64). Species of *Isoscelipteron*, *Berotha*, and *Lekrugeria* inhabit the plateau’s eastern and southern margin of elevation around 1,000 m, whereas species of *Asadeteva* and *Nodalla* on the plateau’s west reach the elevation of over 3,000 m. Along with species in the Rockies and Andes, their high-elevation adaptation contributes to the accelerated ER of habitat elevation in the Neogene, associated with the rapid diversification in this period (Fig. 2*F* and *SI Appendix*, Fig. S9). ASR and ER jointly demonstrate that the beaded lacewings had adapted high elevation temporarily in the Jurassic-Early Cretaceous and early Paleogene (*SI Appendix*, Figs. S7–S9). Their multiple and independent alpine exploration as well as consistent relatively high latitudinal tendency were likely driven by their cooler predilection. During the Miocene, beaded lacewings attained the Australian (AU) realm for the first time when the plate approached Asia, for which, tectonics set the stage once again (65, 66). Collectively, the dispersal frequency shows a significantly positive correlation with WL (Figs. 3*C* and 5 *A* and *B*). The berothids with forewings longer than 6.7 mm show notably more frequent dispersions (*SI Appendix*, Fig. S16). This further indicates the advanced flight efficiency from wing elongation benefited beaded lacewings for dispersal over large-scale geological history.

The evolution and dispersal history of beaded lacewings unveils an intricate interaction between Earth environmental change and biological adaptation for over 170 My and involving multiple factors. Concerning external circumstances, global climate change had a significant impact on the lineage’s diversity, a causal effect on NP and NB variation, and facilitated large-scale dispersal during their first radiative phase. Tectonics brought about necessary paths for the lacewings to occupy the final two available realms in the Cenozoic and shaped the biogeography of modern berothids. In regard to beaded lacewings per se, they accomplished lineage diversification and geographical dispersal by niche expansion in concert with dispersal ability enhancement, in response to a constantly changing Earth with intermittently advantageous or disadvantageous outcomes. The niche variation played key roles in their dispersal history, particularly in the first phase, by means of durational expansion of NB and adjusting relevant niche factors under patterns of phylogenetic conservatism and evolutionary lability with regard to different variables. This accounts for the early-stage elevated rate of change for most relevant niche factors (Fig. 3 *B*, *E*, and *F*). Since the late Paleogene, beaded lacewings dispersed rapidly and occupied all the zoogeographic realms, which can be attributed to the considerably increased lift-to-drag ratio through wing elongation and trade-offs between raising lift-to-drag ratio and reducing drag coefficient by the divergence of wing shapes co-occurring in the Cenozoic. By compiling paleo/modern climate and fossil/extant species data, synthesizing phylogenetic analyses, niche modeling, CFD modeling, and historical biogeographic analyses, a dispersal map of beaded lacewings through over 170 My was reconstructed, revealing a long-term game between the evolving Earth and the organisms’ changing adaptive strategies.

## Materials and Methods

### Data Compilation.

#### Taxa selection.

The data matrix consists of 103 morphological characters and 53 taxa (Dataset S6), including 50 genera of Berothidae and three outgroup genera: *Nallachius* (Dilaridae), *Plega* (Mantispidae), and *Mucroberotha* (Rhachiberothidae). All operational taxonomic units (OTUs) in the matrix are genera. The ingroups include all 25 extant genera of Berothidae. Additionally, 25 fossil genera were included in the ingroups, encompassing representatives from all geological strata with fossil berothid records, which ranges from the Middle Jurassic to the Eocene (26). Only genera from a same stratum of ingroups but with limited available characters were excluded.

Fossil ages were used for estimating divergence times in Bayesian tip-dating analyses, referring to the lower and upper boundaries of the fossil yielding stratigraphic ages. The chronological constraints of fossil taxa were derived from the original literature and referred to the database Lacewing Digital Library (26) (LDL: https://lacewing.tamu.edu).

#### Morphological characters and molecular sequences in the matrix.

Totally 103 morphological characters of adult berothids were collected from the head, thoraces, legs, wings, abdomen, and genitals. Characters were coded from the original literature, photos, and examination of type specimens. All character states were treated as discrete and unordered. Taxa were coded as polymorphic when a taxon has more than one state. Unknown character state is coded as “?,” and inapplicable character is coded as “−.” Most of the genera were coded according to the holotypes. When some characters are not preserved in the holotype, the matrix refers to other specimens or species in the same genus. Sequences of four genes (16S, 18S, *CO1*, and *CAD*) for species of nine extant genera (*Berotha*, *Isoscelipteron*, *Lomamyia*, *Naizema*, *Ormiscocerus*, *Podallea*, *Quasispermophorella*, *Spiroberotha*, and *Stenobiella*) of Berothidae and three outgroups (*Mucroberotha*, *Plega*, and *Nallachius*) were retrieved from GenBank (Dataset S8). The sequences were aligned using MEGA X (67). The morphological and molecular matrices were coded and concatenated in Mesquite ver. 3.70 (68).

#### Distribution data of fossil and extant species.

The distribution data of fossil and extant species of Berothidae were compiled from databases, including Fossilworks (http://www.fossilworks.org), LDL (26) (https://lacewing.tamu.edu), and literature (27, 28, 69, 70). The fossil data were subsequently processed based on the modern coordinates and stratigraphic ages of each fossil deposit. Using the R package “rgplates,” the fossil species distribution data were converted to the paleocoordinates of their corresponding time (71, 72).

#### Paleo- and modern climate variables.

The environmental variables were selected based on the rationale of *i*. the environmental variables that may influence or restrict the terrestrial biogeography, and *ii*. the compatibility of the available data in modern and deep time. Eventually, eleven variables were used in this research (*SI Appendix*, Table S1). The global modern environmental raw data were collected from the database WorldClim (73) (https://worldclim.org) at 0.167° × 0.167° resolution. For the unavailable variables in the database, they were calculated using netCDF Operators according to criteria in ref. 74. The global paleoenvironmental data were extracted for every 10-My interval from literature (32, 75), then processed and applied at 1° × 1° resolution in latitude and longitude (Global background variables in *SI Appendix*, Table S1). Then the global mean values were calculated for each 10-My interval. The global mean MAT was derived from ref. 31, which provides data of finer time scale.

The environmental data of each individual specimen locality were extracted according to their (paleo)coordinates of corresponding time (berothid habitat variables in *SI Appendix*, Table S1). In total, data were compiled from 67 fossil and 1,136 extant recorded specimens covering all the valid berothid species.

### Ecological Niche Modeling (ENM) for Extant and Fossil Berothids.

#### Data processing.

Eleven (paleo)environmental variables of all fossil and extant berothid specimen localities were collected for ENM. First, the correlations among environmental variables were tested using the Pearson correlation coefficient to avoid overfitting in the modeling (*SI Appendix*, Table S7). The high correlated variables were grouped for ensuing selection. Next, principal component analysis (PCA) was performed on all variables. According to the contributions of the variables to the principal components, the most contributable variable in each high correlated group was selected for the subsequent modeling. After screened, six environmental variables were used in the modeling (*SI Appendix*, Table S8).

The fossil and extant specimens were divided into two datasets for separately modeling, in order to compare their niche difference or similarity. Considering the numerous and densely distributed extant specimens and the possibly consequent overfitting geographically, the data were screened on the basis of 6-km buffer.

#### Hyperparameters optimization.

The hyperparameters were pretested and selected using the R package “ENMeval” before the modeling (76). According to the results (*SI Appendix*, Figs. S17 and S19), we used all linear features for modeling of the fossil sets and autofeatures for the extant set. Besides, random seeds were set as 20% for the random test, and the regularization multiplier was set as 1.5.

#### Ecological niche modeling.

The modeling was performed using MaxEnt separately for *i*. all fossils, *ii*. Mesozoic-only fossils, and *iii*. extant specimen datasets (77). Each set was repeated ten times and the averages were used as the final results. The models were evaluated by AUC (area under curve of receiver operating characteristic) (*SI Appendix*, Figs. S18 and S20). Since the fossil species were from different geologic times, the environmental variables of each fossil specimen were extracted according to its geochronologic time and paleocoordinates. Then, the exact environmental data of each fossil were projected to replace the corresponding paleocoordinates on the paleoclimate and paleogeographic map at 100 Ma as a representative in the deep time. The same procedure was applied to the modern climate and geographic map. So, the fossil dataset was modeled independently on the global circumstances at 100 Ma and the present. For comparison, the modeled niches of extant species based on the modern Earth was projected on the paleoclimate and paleogeographic map at 100 Ma too.

### Phylogenetic Analyses.

#### Phylogenetic analyses and divergence time calibration.

Bayesian tip-dating analyses were performed to assess the phylogenetic relationships among genera of Berothidae by MrBayes 3.2.7 (78). The fossilized birth–death process (FBD) was used as a prior for the time tree (79). The tree age prior for the FBD process was assigned an offsetgamma prior with mean age of 165.9 Ma and minimal age of 163.5 Ma, derived from the oldest fossil berothid species of *Sinosmylites* (24, 80). The uncorrelated independent gamma rates (IGR) clock model (81) combined with the gamma-distributed rate model were selected by stepping-stone sampling and estimating marginal likelihoods. Two simultaneous, completely independent runs were executed in Markov chain Monte Carlo, with four chains (three heated chains and one cold chain with temperature 0.04) per run for 80 million generations and sampled every 2,000 generations. Two runs converged when the average SD of split frequencies is less than 0.005. The first 25% of samples were discarded as burn-in. The remaining samples from the two runs were combined. Parsimony analyses were conducted using TNT ver. 1.6 (82). All characters were treated as unordered and equally weighted. Bootstrap support values were calculated from 1,000 heuristic search (TBR) pseudoreplicates of resampled datasets.

#### Diversification and ER.

Based on the time-calibrated phylogeny, LTT plot was obtained utilizing the R package “phytools” (83). NDR, speciation rate, extinction rate of the family, and ER of morphological traits and niche variables were estimated using BAMM v2.6 (84) (*SI Appendix*, Fig. S21). The prior parameters used in the analyses were evaluated using the R package “BAMMtools” (85). Each analysis utilized four reversible Markov chains and ran for over 2 million iterations, outputting every 2,000 generations. The outputs were subsequently analyzed using BAMMtools to burn in the first 20% of the samples and evaluate convergent by referring to effective sample size threshold of 200.

#### ASR.

ASR for continuous trait data was analyzed using R phytools (83), and for discrete trait data using R “MBASR” (86). The continuous traits include the morphological forewing length, and the niche variables, i.e., NP, *H-*MAT, habitat temperature seasonality (*H-*TS), *H-*MAP, *H-*WMP, *H-*DMP, *H-*PS, *H-*EQ, *H-*CI, *H-*WMT, *H-*CMT, *H-*EL. The discrete data contain one morphological trait, the wing shape.

For further analyses, the reconstructed values on the ancestral nodes were extracted for all the niche variables. We divided the entire time interval into 10-My bins and calculated the maximum and minimum values of each niche variable for the bin as the ecological threshold. Using the thresholds and paleoclimate data, the potential suitable area through time bin series according to each niche variable was projected on the paleogeographic map of corresponding age (*SI Appendix*, Figs. S15, S22, and S23). Besides, the mean value of branch portion in each time bin was calculated using Matlab script for further niche analyses.

#### PSig and evolution model.

Pagel’s *λ* and Blomberg’s *K* were both used to detect PSig, using the R package “phylosignal” (87–89). Subsequently, we utilized the R package “geiger” to assess models of evolution for all of the morphological and niche traits (90). The testing models include white-noise (nonphylogenetic) model (WN), Brownian motion model (BM), rate trend model (RT), mean trend model (MT), delta model (DT), lambda model (LB), kappa model (KP), Early-burst model (EB), and Ornstein–Uhlenbeck model (OU).

### Historical Biogeography.

#### Criterion and parameter settings.

For the realm division and definition, we primarily referred to the world zoogeographic regions in ref. 91. But concerning the plate tectonics over a 170-My geological history, modifications were made to better depict the adjacency and separation of terranes through the periods. The Sino-Japanese was unified within PA, Oceanian unified within AU, Panamanian unified within NT, Madagascan unified within AT. The Indian (IN) was separated from OR. Margins of the SA and IN were modified too. In our final division scenario, eight biogeographic realms are identified. They are the PA, OR, IN, SA, AT, AU, Nearctic (NA), NT (*SI Appendix*, Fig. S24).

A time-stratified model was set where the spatial relationships and dispersion multiplier were variable over different periods according to paleogeographic maps in ref. 38. Regarding the dispersal multipliers, when two plates were adjacent to each other, the dispersal multipliers between them were set as 0.75. If a geographic barrier exists(ed) between two regions, then the dispersal multipliers between them were set as 0.5. If two or more geographic barriers or other terranes exist(ed) between two regions, then the dispersal multipliers between them were set as 0.000001 (Dataset S10).

#### Historical biogeography analyses and dispersal frequencies estimation.

The maximum likelihood estimations of ancestral area of the inner nodes in Berothidae phylogeny were assessed using the R package “BioGeoBEARS” (92, 93). Initially, all models were tested, including the LAGRANGE Dispersal-Extinction-Cladogenesis (DEC) model (94), the Dispersal-Vicariance Analysis like (DIVALIKE) model (95), BAYAREALIKE model (96), and their respective derivative founder-event jump dispersal (+j) models (founder-event speciation).

Besides the model evaluating process by the package, we also referred to the reconstructed habitable areas in the deep time from the preceding analyses. Combining the AICc value and niche verification, the DIVALIKE model was selected for the final estimating analyses. Using the best-fitting model, 200 Biogeographic Stochastic Mappings (BSM) were performed, which calculated the biogeographical dispersal events and the corresponding percentages. The dispersal events were estimated for the entire family.

The number of dispersal events per My from the biogeographic analysis was extracted. The average WL for each 1-My time bin was derived from the ASR result. Spearman correlation coefficient between dispersal events per My and WL was calculated. Afterward, the dispersal event data were divided into two categories: *i*. the more frequent dispersal group valued 1 for bins with dispersal events exceeding the mean value, and *ii*. the less frequent dispersal groups valued 0 for bins with dispersal events less than the mean value. A hidden Markov model was constructed by integrating the categorized dispersal data and WL data, employing the R package “HMM” (97). Logistic regression was then performed in the R package “ggplot2” (98) using 10,000 iterations generated by the hidden Markov model. For both raw data and hidden Markov model simulated data, kernel density estimations were conducted to model their probability distribution.

### NB and NP.

The NB and NP were assessed using Outlying Mean Index analyses by the R package “ade4” (99, 100). Two datasets are used for the analyses. One is the environmental data of different periods, and another one is the abundance of berothids in the corresponding period and site. We proceeded with two sets of experiments according to different age division schemes. In the first scheme, all the environmental conditions of fossil and extant species were compiled, and then divided into five geochronological intervals (*SI Appendix*, Table S9). For the other one, we extracted and supplemented the environmental variables from all the inner nodes according to the results of ASR. Most environmental variables were divided into time bins of 10 My, except the period from 105 to 65 Ma. The time bins in this period were divided for every 20 My owing to limited records and data during this interval. Totally, there were 16 bins in the second scheme (*SI Appendix*, Table S10).

### Causal Inference.

Convergent Cross-mapping analyses were performed to infer the causality between the global climate changes and biological variation involving NB, NP, LTT, and NDR (101) (*SI Appendix*, Figs. S25 and S26). The analyses were conducted using the Python package “causal_ccm” (102). Two opposite causal directions between the climatic and biological variables of each set were tested in the analysis. The results were evaluated by comparing the prediction skills through test length and *P*-values between the two directions. Thus, only the direction with mostly higher prediction skill as well as lower and significant *P*-value indicates the significant and more probable causality direction between the two variables, while the other or both direction(s) are denied.

### CFD Modeling.

#### Data compilation and statistical analyses.

Atmospheric variables of all the extant berothid individual localities were collected from the database WorldClim (73) (https://worldclim.org) and further processed for testing their influences on flight aerodynamic efficiency in the subsequent analyses. The air density (*ρ*) is calculated according to Dalton’s law of partial pressure (103) (Formula 1), and the air viscosity (*μ*) is calculated by Sutherland’s law (104) (Formula 2), as follows:

$$\rho =\frac{P}{R_{g,d}T}\left[1-\frac{R_{g,d}P_{v}}{P}\left(\frac{1}{R_{g,d}}-\frac{1}{R_{g,v}}\right)\right],$$

$$\mu =\mu_{\mathrm{ref}}{\left(\frac{T}{T_{\mathrm{ref}}}\right)}^{3/2}\cdot \frac{T_{\mathrm{ref}}+S}{T+S},$$

where *P* is the atmospheric pressure; *P_v_* refers to the partial pressure of water vapor, reflecting the moisture content of the air; *T* is the absolute temperature; *R_g,v_* is the gas constant for water vapor, taken as 461.5 J/(kg·K); and *R_g,d_* is the gas constant for dry air, taken as 287.1 J/(kg·K). In addition, *T_ref_* is a reference temperature; *μ_ref_* is the viscosity at the reference temperature *T_ref_*; *S* is the Sutherland temperature, taken as 110.4 K.

The forewing lengths, the atmospheric variables, and relevant niche climate factors of all berothid species and individual localities were compiled and computed. The forewing lengths of falcate wings ranged from 6.4 to 14.1 mm. In contrast, the oval-shaped wings ranged from 1.7 to 15 mm, but mostly in short length. The data of forewing lengths, the atmospheric variables, and relevant niche climate factors between two shape types were tested for independent conditions by Welch’s *t* test and Mann–Whitney test. The correlations of habitat environmental variables with WL/shape were analyzed using phylogenetic generalized least squares analysis with the R package “caper” (105).

#### Three-dimensional models establishment.

One species of each wing-shape type was selected for modeling, i.e., the falcate *Isoscelipteron pectinatum* and the oval-shaped *Iceloberotha simulatrix*. 3D models were created using Blender (v. 3.6) and further processed in Ansys SpaceClaim for the subsequent CFD analyses. We utilized the Meshing component in Ansys Workbench to implement mesh generation, and performed the final simulations in Fluent (v. 2022 R1).

The field domain settings in the CFD modeling were based on the berothid habitat environmental conditions, involving airflow velocity (*v*), air density (*ρ*), and viscosity (*μ*). After calculated and compiled these three atmospheric variables of all extant berothid individual specimen records, we derived the maximum, minimum, and mean values of each variable in the dataset (*SI Appendix*, Fig. S27 and Table S11). In the transient analyses, six sets of field domain conditions were constructed using the maximum or minimum value of one of the variables combined with the mean value of the remaining variables, which signified the extremum of each variable (*SI Appendix*, Table S3). In the ensuing steady analyses, the field domain conditions were set as the mean values of airflow velocity, air viscosity, and the minimum value of air density for a relatively adverse circumstance. Additionally, for testing temperature influence on aerodynamic efficiency between wing shapes, the median value of *H*-MAT of each wing shape habitats as well as the atmospheric variables of the corresponding habitat were used to perform steady analyses for each wing shape.

#### Fluent setup.

The airflow velocity of each domain was set at the inlet boundary, which is anterior to the insect. The turbulence intensity at the inlet boundary was set as 5% and a zero-pressure at the outlet boundary, which is posterior to the insect. The default settings of the physical model SST k-*ω* were used uniformly for all simulations, with the effects of gravity considered. During the modeling, the pressure-based solver and coupled algorithm were used to solve the Reynolds-averaged Navier–Stokes equations, till the residual reaching convergence. In the transient analyses, the Bounded second-order implicit formulation was applied and the Flow Courant Number was set to 200. The pseudotime method was used in steady analyses. The lift coefficient and drag coefficient were monitored during the analyses. For each steady simulation and each time step in the transient analyses, the computation converged when residuals reduced to less than 0.001. In the analyses for temperature impact, the absolute criteria for residuals were set to 1e-06.

#### Transient analyses.

In the transient analyses, CFD simulations of berothid with each wing shape/length were performed in six flow field conditions. The computational domain is composed of a cube with length of each side as 100 mm. For oval-shaped wing of 4.5 mm long, the side length of the domain cube is 50 mm instead. The field model was meshed using an overset mesh approach in Ansys-Meshing. The component domains were constructed by applying Boolean operations. By resizing the global and local dimensions and capturing the curvature and proximity, the model was meshed until reaching quality criterion, which is justified by the minimum orthogonal quality greater than 0.2 and no orphan meshes present. During the modeling, the wing vibration frequency was 20 Hz and the flapping angle amplitude was 120°, referring to the observation data of lacewings and other insects (106, 107). The maximum lift-to-drag ratio (*L/D*_max_) over an entire stroke cycle of each simulation was extracted and used to compare the flight efficiency among wing shapes and lengths under different environmental conditions. The median values of the forewing lengths of either wing shape were used in the simulation (9 mm for falcate wing, 4.5 mm for oval-shaped wing). Afterward, oval-shaped wing of 9 mm long was also simulated for comparison.

#### Steady analyses.

In the steady analyses, the fluid domain was established in SpaceClaim by constructing a shell for each case of modeling with a default cushion of 200%. The size of cubic domain is approximately 45 × 40 × 36 mm^3^. By adjusting the mesh sizes and capturing the curvature and proximity in Ansys-Meshing, the model was meshed until reaching quality criterion, which is justified by the minimum orthogonal quality greater than 0.2. The lift coefficient (*C_l_*), drag coefficient (*C_d_*), and lift-to-drag ratio (*L/D*) of the two wing shapes were calculated for comparison. Both types of wings were of the same length in 9 mm, at wing angles-of-attack (α) of 2°, 4°, 6°, 8°, 10°, 12°, 16°, 20°, 24°, 30°, and 36°. In the steady analyses, the wings are assumed to be rigid flat plates and their aerodynamic effects are considered as symmetric. Therefore, only one wing was simulated in the analyses.

## Supplementary Material

Appendix 01 (PDF)

Dataset S01 (XLSX)

Dataset S02 (XLSX)

Dataset S03 (XLSX)

Dataset S04 (XLSX)

Dataset S05 (XLSX)

Dataset S06 (RTF)

Dataset S07 (RTF)

Dataset S08 (XLSX)

Dataset S09 (PDF)

Dataset S10 (XLSX)

## Data Availability

All study data are included in the article and/or supporting information.

## References

[1] A. R. Wallace, The geographical distribution of animals, with a study of the living and extinct faunas, as elucidating the past changes of the Earth’s surface. Nature **14**, 186–189 (1876).

[2] J. L. Rocha , North African fox genomes show signatures of repeated introgression and adaptation to life in deserts. Nat. Ecol. Evol. **7**, 1267–1286 (2023). 37308700 10.1038/s41559-023-02094-w PMC10527534

[3] C. T. Griffin , Africa’s oldest dinosaurs reveal early suppression of dinosaur distribution. Nature **609**, 313–319 (2022). 36045297 10.1038/s41586-022-05133-x

[4] R. J. Whittaker, J. M. Fernández-Palacios, T. J. Matthews, M. K. Borregaard, K. A. Triantis, Island biogeography: Taking the long view of nature’s laboratories. Science **357**, eaam8326 (2017). 28860356 10.1126/science.aam8326

[5] D. Lai , The associated evolution of raptorial foreleg and mantispid diversification during 200 million years. Natl. Sci. Rev. **10**, nwad278 (2023). 38033734 10.1093/nsr/nwad278 PMC10686013

[6] B. Ochocki, T. Miller, Rapid evolution of dispersal ability makes biological invasions faster and more variable. Nat. Commun. **8**, 14315 (2017). 28128215 10.1038/ncomms14315 PMC5290149

[7] L. T. Lancaster, R. Y. Dudaniec, B. Hansson, E. I. Svensson, Latitudinal shift in thermal niche breadth results from thermal release during a climate-mediated range expansion. J. Biogeogr. **42**, 1953–1963 (2015).

[8] C. O’Donovan, A. Meade, C. Venditti, Dinosaurs reveal the geographical signature of an evolutionary radiation. Nat. Ecol. Evol. **2**, 452–458 (2018). 29403079 10.1038/s41559-017-0454-6

[9] W. T. T. Taylor , Early dispersal of domestic horses into the Great Plains and northern Rockies. Science **379**, 1316–1323 (2023). 36996225 10.1126/science.adc9691

[10] H. Tejero-Cicuéndez , Reconstructing squamate biogeography in Afro-Arabia reveals the influence of a complex and dynamic geologic past. Syst. Biol. **71**, 261–272 (2022). 33787928 10.1093/sysbio/syab025 PMC8830062

[11] C. Hsieh , Evolutionary history and environmental variability structure contemporary tropical vertebrate communities. Glob. Ecol. Biogeogr. **33**, e13829 (2024).

[12] P. Descombes, F. Leprieur, C. Albouy, C. Heine, L. Pellissier, Spatial imprints of plate tectonics on extant richness of terrestrial vertebrates. J. Biogeogr. **44**, 1185–1197 (2017).

[13] A. Skeels , Paleoenvironments shaped the exchange of terrestrial vertebrates across Wallace’s Line. Science **381**, 86–92 (2023). 37410831 10.1126/science.adf7122

[14] R. P. Moore, W. D. Robinson, I. J. Lovette, T. R. Robinson, Experimental evidence for extreme dispersal limitation in tropical forest birds. Ecol. Lett. **11**, 960–968 (2008). 18513315 10.1111/j.1461-0248.2008.01196.x

[15] L. O. Loureiro, M. D. Engstrom, B. K. Lim, Biogeography of Neotropical mastiff bats: A case of multiple dispersals between the Caribbean and mainland. J. Biogeogr. **48**, 1353–1365 (2021).

[16] M. S. Engel, S. R. Davis, J. Prokop, “Insect wings: The evolutionary developmental origins of Nature’s first flyers” in Arthropod Biology and Evolution, A. Minelli, G. Boxshall, G. Fusco, Eds. (Springer, 2013), pp. 269–298.

[17] M. S. Engel, Insect evolution. Curr. Biol. **25**, 868–872 (2015). 26439349 10.1016/j.cub.2015.07.059

[18] D. Grimaldi, M. S. Engel, Evolution of the Insects (Cambridge University Press, 2005).

[19] J. L. Gressitt, Insect biogeography. Annu. Rev. Entomol. **19**, 293–321 (1974).

[20] R. Dudley, The Biomechanics of Insect Flight: Form, Function, Evolution (Princeton University Press, 2000).

[21] F. W. Hope, Observations on succinic insects. Trans. R. Entomol. Soc. Lond. **1**, 133–147 (1836).

[22] A. R. Wallace, On the zoological geography of the Malay Archipelago. Zool. J. Linn. Soc. **4**, 172–184 (1859).

[23] A. R. Wallace, The Malay Archipelago: The Land of the Orang-Utan, and the Bird of Paradise. A Narrative of Travel, with Studies of Man and Nature (Macmillan and Co., 1869).

[24] V. N. Makarkin, Q. Yang, D. Ren, Two new species of *Sinosmylites* Hong (Neuroptera, Berothidae) from the Middle Jurassic of China, with notes on Mesoberothidae. ZooKeys **130**, 199–215 (2011). 10.3897/zookeys.130.1418 PMC3260760 22259277

[25] Q. Yang, C. Shi, D. Ren, A new genus and species of berothids (Insecta, Neuroptera) from the Late Cretaceous Myanmar amber. ZooKeys **864**, 99–109 (2019). 31367178 10.3897/zookeys.864.35271 PMC6658572

[26] J. D. Oswald, Data from “Lacewing digital library”. http://lacewing.tamu.edu. Accessed 15 January 2023.

[27] U. Aspöck, S. Randolf, Beaded lacewings—A pictorial identification key to the genera, their biogeographics and a phylogenetic analysis (Insecta: Neuroptera: Berothidae). Dtsch. Entomol. Z. **61**, 155–172 (2014).

[28] R. J. P. Machado, C. C. Martins, H. Aspöck, L. G. De Miranda Tavares, U. Aspöck, The first cave associated genus of Berothidae (Insecta: Neuroptera), and a new interpretation of the subfamily Cyrenoberothinae. Zool. J. Linn. Soc. **195**, 1422–1444 (2022).

[29] R. S. Dietz, J. C. Holden, Reconstruction of Pangaea: Breakup and dispersion of continents, Permian to present. J. Geophys. Res. **75**, 4939–4956 (1970).

[30] W. Frisch, M. Meschede, R. C. Blakey, Plate Tectonics: Continental Drift and Mountain Building (Springer, 2011).

[31] C. R. Scotese, H. Song, B. J. W. Mills, D. G. van der Meer, Phanerozoic paleotemperatures: The earth’s changing climate during the last 540 million years. Earth-Sci. Rev. **215**, 103503 (2021).

[32] X. Li , A high-resolution climate simulation dataset for the past 540 million years. Sci. Data **9**, 371 (2022). 35764652 10.1038/s41597-022-01490-4 PMC9240078

[33] J. R. Forster, Observations Made during a Voyage Round the World: On Physical Geography, Natural History, and Ethnic Philosophy (G. Robinson, 1778).

[34] A. von Humboldt, Ansichten der Natur mit wissenschaftlichen Erläuterungen (J. G. Cotta, 1808).

[35] M. R. Willig, D. M. Kaufman, R. D. Stevens, Latitudinal gradients of biodiversity: Pattern, process, scale, and synthesis. Annu. Rev. Ecol. Evol. Syst. **34**, 273–309 (2003).

[36] E. E. Saupe , Spatio-temporal climate change contributes to latitudinal diversity gradients. Nat. Ecol. Evol. **3**, 1419–1429 (2019). 31501506 10.1038/s41559-019-0962-7

[37] I. S. Fenton, T. Aze, A. Farnsworth, P. Valdes, E. E. Saupe, Origination of the modern-style diversity gradient 15 million years ago. Nature **614**, 708–712 (2023). 36792825 10.1038/s41586-023-05712-6

[38] C. R. Scotese, An atlas of Phanerozoic paleogeographic maps: The seas come in and the seas go out. Annu. Rev. Earth Planet. Sci. **49**, 679–728 (2021).

[39] Á. T. Kocsis, C. R. Scotese, Mapping paleocoastlines and continental flooding during the Phanerozoic. Earth-Sci. Rev. **213**, 103463 (2021).

[40] J. H. Brown, On the relationship between abundance and distribution of species. Am. Nat. **124**, 255–279 (1984).

[41] J. Heino, G. de Mendoza, Predictability of stream insect distributions is dependent on niche position, but not on biological traits or taxonomic relatedness of species. Ecography **39**, 1216–1226 (2016).

[42] Y. Li , Climate and topography explain range sizes of terrestrial vertebrates. Nat. Clim. Chang. **6**, 498–502 (2016).

[43] H. Liu , Niche evolution and historical biogeography of lady slipper orchids in North America and Eurasia. J. Biogeogr. **48**, 2727–2741 (2021).

[44] T. Westerhold , An astronomically dated record of Earth’s climate and its predictability over the last 66 million years. Science **369**, 1383–1387 (2020). 32913105 10.1126/science.aba6853

[45] K. G. Miller , Cenozoic sea-level and cryosphere evolution from deep-sea geochemical and continental margin records. Sci. Adv. **6**, eaaz1346 (2020). 32440543 10.1126/sciadv.aaz1346 PMC7228749

[46] C. S. Ma, G. Ma, S. Pincebourde, Survive a warming climate: Insect responses to extreme high temperatures. Annu. Rev. Entomol. **66**, 163–184 (2021). 32870704 10.1146/annurev-ento-041520-074454

[47] J. F. Terlau , Microhabitat conditions remedy heat stress effects on insect activity. Glob. Change Biol. **29**, 3747–3758 (2023). 10.1111/gcb.16712 37186484

[48] J. B. Losos , Niche lability in the evolution of a Caribbean lizard community. Nature **423**, 542–545 (2003). 10.1038/nature01814 12891355

[49] J. B. Losos, Phylogenetic niche conservatism, phylogenetic signal and the relationship between phylogenetic relatedness and ecological similarity among species. Ecol. Lett. **11**, 995–1003 (2008). 18673385 10.1111/j.1461-0248.2008.01229.x

[50] J. J. Wiens, C. H. Graham, Niche conservatism: Integrating evolution, ecology, and conservation biology. Annu. Rev. Ecol. Evol. Syst. **36**, 519–539 (2005).

[51] J. R. Brushwein, Bionomics of *Lomamyia hamata* (Neuroptera: Berothidae). Ann. Entomol. Soc. Am. **80**, 671–679 (1987).

[52] C. A. Tauber, M. J. Tauber, *Lomamyia latipennis* (Neuroptera: Berothidae) life history and larval descriptions. Can. Entomol. **100**, 623–629 (1968).

[53] N. D. Penny, J. R. Arias, J. S. Armistead, Seasonal emergence of Neuroptera in Fairfax County, Virginia. Proc. Calif. Acad. Sci. **58**, 7–19 (2007).

[54] C. Le Roy , Adaptive evolution of flight in *Morpho* butterflies. Science **374**, 1158–1162 (2021). 34822295 10.1126/science.abh2620

[55] G. A. McCulloch, G. P. Wallis, J. M. Waters, Does wing size shape insect biogeography? Evidence from a diverse regional stonefly assemblage Glob. Ecol. Biogeogr. **26**, 93–101 (2017).

[56] M. S. Engel, D. A. Grimaldi, Diverse Neuropterida in Cretaceous amber, with particular reference to the paleofauna of Myanmar (Insecta). Nova Suppl. Entomol. **20**, 1–86 (2008).

[57] A. J. Ross, Complete checklist of Burmese (Myanmar) amber taxa 2023. Mesozoic **1**, 21–57 (2024).

[58] T. Maxworthy, The fluid dynamics of insect flight. Annu. Rev. Fluid Mech. **13**, 329–350 (1981).

[59] M. Dickinson, Solving the mystery of insect flight. Sci. Am. **284**, 48–57 (2001). 11396342 10.1038/scientificamerican0601-48

[60] A. Hedenström, F. Liechti, Field estimates of body drag coefficient on the basis of dives in passerine birds. J. Exp. Biol. **204**, 1167–1175 (2001). 11222132 10.1242/jeb.204.6.1167

[61] P. Patriat, J. Achache, India-Eurasia collision chronology has implications for crustal shortening and driving mechanism of plates. Nature **311**, 615–621 (1984).

[62] R. A. Beck , Stratigraphic evidence for an early collision between northwest India and Asia. Nature **373**, 55–58 (1995).

[63] X. Hu , The timing of India-Asia collision onset—Facts, theories, controversies. Earth-Sci. Rev. **160**, 264–299 (2016).

[64] C. Rahbek , Humboldt’s enigma: What causes global patterns of mountain biodiversity? Science **365**, 1108–1113 (2019). 31515383 10.1126/science.aax0149

[65] P. H. Raven, D. I. Axelrod, Plate tectonics and Australasian paleobiogeography: The complex biogeographic relations of the region reflect its geologic history. Science **176**, 1379–1386 (1972). 17834636 10.1126/science.176.4042.1379

[66] R. Hall, M. A. Cottam, M. E. J. Wilson, The SE Asian Gateway: History and Tectonics of the Australia-Asia Collision (Geological Society of London, 2011).

[67] S. Kumar , MEGA X: Molecular evolutionary genetics analysis across computing platforms. Mol. Biol. Evol. **35**, 1547–1549 (2018). 29722887 10.1093/molbev/msy096 PMC5967553

[68] W. P. Maddison, D. R. Maddison, Mesquite: A modular system for evolutionary analysis (Version 3.70, 2021). https://www.mesquiteproject.org/home.html. Deposited 21 August 2021.

[69] D. Li, H. Aspöck, U. Aspöck, X. Liu, A review of the beaded lacewings (Neuroptera: Berothidae) from China. Zootaxa **4500**, 235–257 (2018). 30486059 10.11646/zootaxa.4500.2.5

[70] D. Li, H. Aspöck, U. Aspöck, X. Liu, New beaded lacewings (Insecta: Neuroptera: Berothidae) from Indochina. Zootaxa **4890**, 509–520 (2020). 10.11646/zootaxa.4890.4.4 33311106

[71] D. R. Müller , GPlates: Building a virtual Earth through deep time. Geochem. Geophys. Geosyst. **19**, 2243–2261 (2018).

[72] Á. T. Kocsis, N. B. Raja, S. Williams, rgplates: R interface for the GPlates web service and desktop application (R package Version 0.5.0., 2024). https://adamtkocsis.com/rgplates/. Deposited 13 August 2024.

[73] S. E. Fick, R. J. Hijmans, WorldClim 2: New 1-km spatial resolution climate surfaces for global land areas. Int. J. Climatol. **37**, 4302–4315 (2017).

[74] S. Noce, L. Caporaso, M. Santini, A new global dataset of bioclimatic indicators. Sci. Data **7**, 398 (2020). 33199736 10.1038/s41597-020-00726-5 PMC7670417

[75] C. R. Scotese, N. Wright, PALEOMAP paleodigital elevation models (PaleoDEMS) for the phanerozoic PALEOMAP project. http://www.earthbyte.org/paleodem-resource-scotese-and-wright-2018/. Deposited 11 August 2018.

[76] J. M. Kass , ENMeval 2.0: Redesigned for customizable and reproducible modeling of species’ niches and distributions. Methods Ecol. Evol. **12**, 1602–1608 (2021).

[77] S. J. Phillips, M. Dudík, R. E. Schapire, Maxent software for modeling species niches and distributions (Version 3.4.1, 2016). https://biodiversityinformatics.amnh.org/open_source/maxent. Deposited 1 December 2020.

[78] F. Ronquist , MrBayes 3.2: Efficient Bayesian phylogenetic inference and model choice across a large model space. Syst. Biol. **61**, 539–542 (2012). 22357727 10.1093/sysbio/sys029 PMC3329765

[79] T. A. Heath, J. P. Huelsenbeck, T. Stadler, The fossilized birth-death process for coherent calibration of divergence-time estimates. Proc. Natl. Acad. Sci. U.S.A. **111**, E2957–E2966 (2014). 25009181 10.1073/pnas.1319091111 PMC4115571

[80] Y. Hong, Middle Jurassic Fossil Insects in North China (Geological Publishing House, 1983).

[81] A. J. Drummond, S. Y. W. Ho, M. J. Phillips, A. Rambaut, Relaxed phylogenetics and dating with confidence. PLoS Biol. **4**, e88 (2006). 16683862 10.1371/journal.pbio.0040088 PMC1395354

[82] P. A. Goloboff, M. E. Morales, TNT version 1.6, with a graphical interface for MacOS and Linux, including new routines in parallel. Cladistics **39**, 144–153 (2023). 36682054 10.1111/cla.12524

[83] L. J. Revell, phytools: An R package for phylogenetic comparative biology (and other things). Methods Ecol. Evol. **3**, 217–223 (2012).

[84] J. S. Mitchell, R. S. Etienne, D. L. Rabosky, Inferring diversification rate variation from phylogenies with fossils. Syst. Biol. **68**, 1–18 (2019). 29788398 10.1093/sysbio/syy035

[85] D. L. Rabosky , BAMMtools: An R package for the analysis of evolutionary dynamics on phylogenetic trees. Methods Ecol. Evol. **5**, 701–707 (2014).

[86] S. Heritage, MBASR: Workflow-simplified ancestral state reconstruction of discrete traits with MrBayes in the R environment. bioRxiv [Preprint] (2021). 10.1101/2021.01.10.426107 (Accessed 1 July 2023).

[87] M. Pagel, Inferring the historical patterns of biological evolution. Nature **401**, 877–884 (1999). 10553904 10.1038/44766

[88] S. P. Blomberg, T. Garland, A. R. Ives, Testing for phylogenetic signal in comparative data: Behavioral traits are more labile. Evolution **57**, 717–745 (2003). 12778543 10.1111/j.0014-3820.2003.tb00285.x

[89] F. Keck, F. Rimet, A. Bouchez, A. Franc, Phylosignal: An R package to measure, test, and explore the phylogenetic signal. Ecol. Evol. **6**, 2774–2780 (2016). 27066252 10.1002/ece3.2051 PMC4799788

[90] M. W. Pennell , geiger v2.0: An expanded suite of methods for fitting macroevolutionary models to phylogenetic trees. Bioinformatics **30**, 2216–2218 (2014). 24728855 10.1093/bioinformatics/btu181

[91] B. G. Holt , An update of Wallace’s zoogeographic regions of the world. Science **339**, 74–78 (2013). 23258408 10.1126/science.1228282

[92] N. J. Matzke, BioGeoBEARS: BioGeography with Bayesian (and likelihood) evolutionary analysis in R scripts (R package version 0.2.1, 2014). http://CRAN.R-project.org/package=BioGeoBEARS. Deposited 18 May 2014.

[93] N. J. Matzke, Model selection in historical biogeography reveals that founder-event speciation is a crucial process in island clades. Syst. Biol. **63**, 951–970 (2014). 25123369 10.1093/sysbio/syu056

[94] R. H. Ree, S. A. Smith, Maximum likelihood inference of geographic range evolution by dispersal, local extinction, and cladogenesis. Syst. Biol. **57**, 4–14 (2008). 18253896 10.1080/10635150701883881

[95] F. Ronquist, Dispersal-vicariance analysis: A new approach to the quantification of historical biogeography. Syst. Biol. **46**, 195–203 (1997).

[96] M. J. Landis, N. J. Matzke, B. R. Moore, J. P. Huelsenbeck, Bayesian analysis of biogeography when the number of areas is large. Syst. Biol. **62**, 789–804 (2013). 23736102 10.1093/sysbio/syt040 PMC4064008

[97] W. Lin, HMM: Hidden Markov Models (R package version 1.0.1, 2022). https://CRAN.R-project.org/package=HMM. Deposited 16 October 2024.

[98] H. Wickham , ggplot2: Create elegant data visualisations using the grammar of graphics (R package version 3.5.1.). https://CRAN.R-project.org/package=ggplot2. Deposited 11 March 2024.

[99] S. Dolédec, D. Chessel, C. Gimaret-Carpentier, Niche separation in community analysis: A new method. Ecology **81**, 2914–2927 (2000).

[100] S. Dray, A.-B. Dufour, The ade4 package: Implementing the duality diagram for ecologists. J. Stat. Softw. **22**, 1–20 (2007).

[101] G. Sugihara , Detecting causality in complex ecosystems. Science **338**, 496–500 (2012). 22997134 10.1126/science.1227079

[102] P. J. E. Javier, Causal-ccm a python implementation of convergent cross mapping (Version 0.3.3, 2021). https://pypi.org/project/causal-ccm/. Deposited 8 June 2021.

[103] F. B. Dutton, Dalton’s law of partial pressures. J. Chem. Educ. **38**, A545 (1961).

[104] W. Sutherland, The viscosity of gases and molecular force. Philos. Mag. **36**, 507–531 (1893).

[105] D. Orme , caper: Comparative analyses of phylogenetics and evolution in R (R package version 1.0.3., 2023). https://CRAN.R-project.org/package=caper. Deposited 6 July 2023.

[106] O. Sotavalta, The flight-tone (wing-stroke frequency) of insects. Acta Entomol. Fenn. **4**, 1–117 (1947).

[107] W. Chen, Z. Zhang, Q. Fu, The wingbeat patterns and frequencies of some insects. Acta Entomol. Sin. **39**, 246–252 (1996).

