# Supplementary material for: Dynamic interplay between niche variation and flight adaptability drove a hundred million years’ dispersion in iconic lacewings
Source: Proc Natl Acad Sci U S A. 2025 May 2;122(19):e2414549122. doi: 10.1073/pnas.2414549122 (PMC12087969; doi:10.1073/pnas.2414549122)
Supplement: Supplementary file 1 — Appendix 01 (PDF) [file pnas.2414549122.sapp.pdf]

PNAS

Supporting Information for

Dynamic interplay between niche variation and flight adaptability drove a hundred million years' dispersion in iconic lacewings.

Haohong Ou, Jingtao Yang, Honglong Wang, Nuoyao Kang, Shumin Li, Yuting Chen, Zihao Peng, Xianzhe Xiang, Michael S. Engel, Shaun L. Winterton, Dong Ren, Qiang Yang, Chaofan Shi

Emails: shichf5@mail.sysu.edu.cn; yq11_1984@126.com; rendong@mail.cnu.edu.cn

This PDF file includes:

Supporting text
Figures S1 to S27
Tables S1 to S11
Legends for Datasets S1 to S10
SI References

Supporting Information

Contents

Ecological niche

- Paleogeographic distribution of fossil berothids
- Statistical analyses on berothid-habitat environmental variables
- Ecological niche modeling for extant and extinct berothids
- Niche breadth and niche position quantification
- Causal inference for niche changes

Phylogeny

- Phylogenetic analyses and divergent time estimation
- Diversification assessment

Phylogenetic patterns evaluation

- Ecological niche changes
- Wing morphological divergence

Computational fluid dynamics

- Transient analyses
- Steady analyses

Figures and Tables

Datasets list

SI References

1. Ecological niche

(1) Paleogeographic distribution of fossil berothids

To further examine the latitudinal distribution pattern of fossil berothids, we converted their paleocoordinates according to the modern coordinates of fossil deposits and the corresponding stratigraphic age (*SI Appendix*, Fig. S2). The results reveal a similar pattern as in Fig. 1*H*, also inconsistent with LDG. Species are scarce between 10°, whereas regions between 10° to 20° possess the maximal species richness, although this could be attributable to the exceptionally abundant fossil records in the Late Cretaceous Kachin amber deposit. Besides, regions between 40° to 50° possess the second largest species richness, followed by those between 50° to 60°. The berothids reached nearly 70° to the poleward. The distribution also exhibits heterogeneity among epochs. During the earliest period, *i.e.*, Jurassic, all species were recorded in the middle latitudes between 40° and 50°. In the Early Cretaceous, the latitudes between 10° and latitudes between 50° and 60° equally yield the most abundant species, while the regions between them have fewer species. In the Late Cretaceous, latitudes between 10° and 20° have the highest species richness, where the speciose Kachin deposit mentioned above is located. This is also the epoch when berothids reached the highest latitudes. During the Paleogene, all reported species were distributed between 40° to 60°. In summary, the middle latitudes hold the highest species richness for the majority of berothid geological history.

(2) Statistical analyses on berothid-habitat environmental variables

For ecological niche modeling, the environmental variables of berothid record sites were collected according to their (paleo)coordinates in the corresponding time. To avoid over-fitting during the modeling, the correlation among the variables was tested firstly referring to the Pearson correlation coefficient. The results find high correlations between *H*-MAT and *H*-CMT; *H*-TS, *H*-CI, and *H*-CMT; *H*-MAP and *H*-WMP (*SI Appendix*, Table S7). Subsequently, Principal Component Analyses (PCA) of the habitat environmental variables were conducted. According to the contribution of the variables to berothid distribution, the variable of lower contribution between the correlated ones was excluded in the ecological niche modeling (*SI Appendix*, Table S8). Eventually, six environmental variables were used for the modeling. They are *H*-WMP, *H*-DMP, *H*-PS, *H*-EQ, *H*-WMT, *H*-CMT.

(3) Ecological niche modeling for extant and extinct berothids

The extant berothids have intensively and densely distributed records, consequently, the data were filtered to avoid over-fitting. Using ArcGIS, a 6-km buffer was established for each data point. For any data point dropped into the buffer zone of another data point, either of them was screened on the premise of retaining as many effective data points as possible for modeling, and no data point in any buffer zone.

Since the fossil data are relatively sparse but ranged over a long geological history, we selected 100 Ma as a representative for deep-time modeling to explore whether the niche of Berothidae has changed between the extinct and extant taxa. First, we extracted the paleoenvironmental variables of each fossil record according to their paleocoordinates and the site paleoenvironmental variables of the corresponding age as their true environmental values. For example, we extracted the environmental data of the paleocoordinates at 110 Ma for the species of *Cantabroberotha*. Then all fossil data were converted to paleocoordinates in 100 Ma according to their fossil deposit coordinates and corresponding age using the R package 'rgplates'. The paleoenvironmental variables of the converted fossil site in 100 Ma were replaced by the true environmental values of each fossil record, while the vacant no-fossil grids on the 100-Ma map remain their original paleoenvironmental values. In other words, we did these simulations using the age-matched paleoenvironmental data, and then converted their paleocoordinates to paleocoordinates at 100 Ma. Using this global distribution data of 100 Ma, the ecological niche modeling was performed for all the fossil species, as in Fig. 1*G*. In the same way, these true fossil environmental values were used to replace their modern site environmental variables, and the fossil ecological niche modeling was conducted and projected on the modern earth (Fig. 1*E*). Besides, we performed modeling with fossil taxa only from Mesozoic rather than all fossils for restricting their time span, using the same method (*SI Appendix*, Fig. S3). In contrast, since the extant species are all from the modern earth, their ecological niche modeling was performed using their exact modern environmental values of the collecting site (Fig. 1*A*). After modeling, using their habitable range of each environmental variable, their suitable habitats were projected on the map of 100 Ma on the basis of its paleoenvironmental conditions (Fig. 1*C*). The modeled results were mapped on the respective (paleo)geographic maps. The suitable habitats were outlined when $p > 0.5$, less suitable habitats

when $0.3 < p < 0.5$, and unsuitable areas when $p < 0.3$.

(4) Niche breadth and niche position quantification

To detect the ecological niches using more specific indices, we assessed the niche breadth and niche position by conducting Outlying Mean Index (OMI) analyses. The niche breadth measures the amplitude of the distribution of species over the multivariate climatic space, which is indicated by the tolerance ('Tol') in the analysis (1). Niche position could be described either by marginality or by the value of the OMI first niche axis (1–4). Marginality is an index measured as the distance of the mean habitat conditions used by a species to the origin of the niche hyperspace calculated by given environmental data (5), so reflects a more comprehensive niche condition of the species to a certain extent. However, as a scalar quantity, marginality is unable to reflect the niche direction change (1, 2). To interpret the evolutionary trend of the beaded lacewing niches in this study, we adopted the value of the OMI first niche axis ('li') to indicate niche position and referred to the marginality as well.

In the OMI analyses, we proceeded with two sets of experiments according to different age division schemes. In the first scheme, we compiled all the environmental data from the habitats of fossil and extant beaded lacewings, and divided them into geochronological intervals. They are Jurassic, Early Cretaceous, Late Cretaceous, Paleogene, and Recent (*SI Appendix*, Fig. S4). In the result, the first axis of OMI analysis accounted for 71.35% of the marginality, and the second axis accounted for 25.40%, accumulatively 96.75%. All the OMI resulting graphs used the first two axes (Fig. 2*A* and *SI Appendix*, Fig. S4). Subsequently, to investigate the niche variation in finer time intervals, we extracted and supplemented the environmental variables from all the inner nodes according to the results of ancestral-state reconstruction. The combined environmental variables were divided into time bins of 10 Myr. But few fossil deposits were reported in the period from 105 Ma to 65 Ma. The time bins in this period were divided for every 20 Myr. In total, 16 bins were in the second scheme (Fig. 2*B*).

Both sets of OMI analyses find extensive variation in niche position and comparable changing trends through their evolutionary history. Comparing the niche breadth and position results between the coarser and finer time interval schemes, both sets of analyses demonstrate that niche breadth around 130 Ma, corresponding to the Early Cretaceous, is distinctly higher

than that in other epochs (Fig. 2*B* and *SI Appendix*, Fig. S4). This similarity suggests that the adaptability of berothids in the Early Cretaceous was better compared with that of other intervals. Niche breadth in the Jurassic was generally consistent with the breadth from 170 Ma to 150 Ma (Fig. 2*B*). Notably, the niche breadth from 100Ma to 80Ma is lower than that in the Late Cretaceous. This difference may be attributed to the concentrated fossil distribution in fine-scale time intervals, but varied significantly across the epoch, although the influence of the node data cannot be disregarded.

We also examined the results of species marginality, *i.e.*, distance to the average niche, as a supplementary index of niche position. It reveals a larger marginality for the fossil species than the extant ones, especially for the Jurassic and Cretaceous berothids. The marginality changed faster in the Cretaceous and slower from the Paleogene to the present. A downward trend could be detected generally (*SI Appendix*, Tables S9 and S10). Considering the vast abundance of extant species compared to the fossil ones, the average niche calculated from the dataset could be influenced by the extant species to a certain extent when we divide the dataset according to time intervals. Consequently, the results indicate a greater discrepancy of berothid niche positions present as time extends deeper (6, 7).

(5) Causal inference for niche changes

To study which environmental factor could cause biological variation involving niche breadth, niche position, lineage through time and net diversification rate, we used convergent cross-mapping (CCM) analysis to explore the causality between biological variation of beaded lacewings and the changes of global environmental conditions. We test the causality of both directions between these variable sets using a Python package ‘causal-ccm’. In this research, the niche data of Berothidae are derived from the OMI analyses on 10/20-myr time bin, and the environmental data are the global average value of each time bin calculated by NCO.

In this method, two main parameters were set: embedding dimension (E) and embedding lag step (τ). The embedding dimension, signifying the number used to construct the embedding data of the system, is set to 2 for the shadow manifold. The embedding lag step, denoting the time lags implied to the embedding, is set to 1. During the part of checking convergence, the interval of test length was set as 1, on account of the amount of data. The test length and prediction skill are used to signify the results. The test length represents the amount of data

used in each causal analysis test, relating to the sample size and the interval. The prediction skill indicates the accuracy of causality in different directions in the test. The CCM result shows the prediction skill (ρ) of the causality between two variables in either direction (a to b, or b to a) as the test length increases. As the test length increases, if the prediction skill of one direction (e.g., a to b) is positive and getting close to +1, and significantly higher than the prediction skill of the opposite direction (b to a) between the two variables, it manifests that the causality of the former direction is strong. Besides, the *p*-value was calculated to indicate the significance of each test. It indicates a strong significance if the *p*-value is < 0.05. The results were evaluated by comparing the prediction skills through test length and *p*-values between the two directions. Thus, only the direction with mostly higher prediction skill as well as lower and significant *p*-value indicates the significant and more probable causality direction between the two variables, while the other direction or both directions would be denied when the *p*-value indicates non-significant. Furthermore, causalities of global climate change with lineage through time (LTT) and net diversity rate (NDR) were tested too (*SI Appendix*, Fig. S5).

In addition to the results shown in the main text, we also tested the causality of other bioclimatic variables. In general, climate fluctuation (*G*-TS and *G*-CI) and average climatic conditions (*G*-MAT and *G*-MAP) have less influence on the niche variation, while extreme conditions have more effect to the niche changes (*G*-DMP and *G*-WMT, *SI Appendix*, Figs. S5 *A–C*, S25 and S26). For the climate fluctuation, as the habitat *H*-TS show highest phylogenetic niche conservatism ($\lambda = 1.02$, $p = 0.0001$; $K = 1.38$, $p = 0.0001$), the global *G*-TS might have less impact on the niche position or breadth change (Fig. 3*E*). For the effect of extreme climate on berothid niches, it could refer to the effect of extreme high temperature (EHT) on insects, as it has been reported for many insect groups (8, 9). Insects' distributions could be affected by EHT, because their tolerance can reflect the adaptation of species to climate, which will further affect changes in the niche breadth. This could be a potential reason that explains the result that *G*-WMT change affects the niche breadth of Berothidae (*SI Appendix*, Fig. S5*B*). Additionally, *G*-WMT is found significantly and negatively correlated with the LTT of beaded lacewings, and could be a causal factor for both LTT and NDR, which further verifies the EHT effect on beaded lacewings (*SI Appendix*, Fig. S5).

2. Phylogeny

(1) Phylogenetic analyses and divergence time estimation

Bayesian tip-dating analyses were performed to recover the phylogenetic relationships of Berothidae based on 50 ingroups and three outgroups, totally 53 genera including 28 extant and 25 extinct genera, combined with 103 morphological characters. The divergent time on the phylogeny was estimated by the uncorrelated independent gamma rates (IGR) clock model and the gamma-distributed rate model with fossil ages as tip calibrations in MrBayes 3.2.7. Two Triassic berothid species were described recently, *Ferganoberotha minutissima* from the Middle-Late Triassic Madygen Formation (Kyrgyzstan) and *Triassoberotha japonica* from the Late Triassic Momonoki Formation (Japan) (10, 11), which are important for the systematics of the Berothidae clade, but unfortunately, we were not able to include these two genera in our analyses. We did a simple phylogenetic test including the genera afterwards. The results indicate *Triassoberotha* sister to the clade including Berothidae, Rhachiberothidae, and Mantispidae, and then *Ferganoberotha* sister to all the taxa above (Dataset S9). Considering that these two genera are preserved only by partial wings and provide limited morphological characters, we suggest their phylogenetic relationships to Berothidae and related families need further study with more and better-preserved fossil specimens. As most fossil specimens have limited preserved characters and thus provide poor resolution of the basal nodes, ingroups were constrained in the final analyses.

Due to the limited molecular sequences available from only nine genera and the limited fossil characters, it is challenging to construct a robust phylogenetic tree for the family Berothidae. We retrieved all available molecular sequences for extant berothids and the outgroups, and conducted phylogenetic analyses with *i*. combined matrix of morphological characters and molecular sequences and *ii*. morphology only matrix. Both parsimony analyses and Bayesian inferences were applied to different matrices. The molecular sequences include 16S, 18S, *CO1*, and *CAD* genes from nine extant genera (*Berotha*, *Isoscelipteron*, *Lomamyia*, *Naizema*, *Ormiscocerus*, *Podallea*, *Quasispermophorella*, *Spiroberotha*, and *Stenobiella*) of Berothidae and three outgroups. The sequences were downloaded in FASTA format. Sequences alignment was performed using MEGA X and concatenated into a matrix using PhyloSuite v. 1.2.2 (12). The morphological data matrix and the molecular sequence matrix were concatenated using the 'Concatenate Matrices' function in Mesquite ver. 3.70, resulting in a

combined matrix.

Parsimony analyses were carried out on TNT v1.6 to recover the phylogenetic relationships of Berothidae based on 50 ingroups and one outgroup (*Plega*), totally 51 genera, including 26 extant and 25 extinct genera, combining 103 morphological characters and 5910 nucleotide loci. An additional analysis was conducted using the parsimony method based on 50 ingroups and one outgroup (*Plega*), with 103 morphological characters.

Among all analyses, the phylogenetic tree recovered from Bayesian inference with morphological matrix shows the highest resolution and the highest support. We labeled the common clade nodes found in the different analyses on the phylogenetic tree from Bayesian inference with morphological matrix (*SI Appendix*, Fig. S6).

The result indicates a divergence onset at approximately 178.7 Ma between the outgroups and the berothid clade. All the Jurassic and Early Cretaceous genera are recovered as stem-groups of the family or subclades. The Late Jurassic genus *Berothone* is recovered as sister to all other berothids, and the divergence time of these two clades was estimated to be ~176.5 Ma. The Early Cretaceous *Oloberotha* from China was sister to *Mesithone* with relatively high support, and these spanned from the late Middle Jurassic to Early Cretaceous and are distributed in Russia and Kazakhstan. The phylogeny also indicates a sister relationship between the Early Cretaceous *Epimesoberotha* from the UK and *Sinosmylites* from the Middle and Late Jurassic of China, Mongolia, and Kazakhstan (*SI Appendix*, Fig. S6). All remaining berothids constitute two major clades. One clade consists of extinct genera spanning from the Jurassic to Paleogene and one extant genus (Clade A). The other clade contains most extant and Cretaceous genera (Clade B).

In Clade A, the Late Jurassic *Krokhathone* from Kazakhstan is recovered as sister to the remaining genera of younger ages. The phylogeny indicates the Early Cretaceous *Pseudosisyra* from Russia and *Sibelliberotha* from Lebanon successively as stem-groups to the remaining Late Cretaceous to Recent taxa. The Late Cretaceous genera, mostly from Burmese ambers, are recovered as stem-groups to the Cenozoic genera. Notably, *Speleoberotha* from South America (Brazil) is the only extant genus grouped in Clade A. The genus is recovered as sister to the Eocene *Microberotha* from North America (Canada). Together, they are sister to the Eocene *Xenoberotha* from North America (USA). The Cenozoic subclade diverged from the

Mesozoic genera at approximately 69.2 Ma, which also shows significant differentiation biogeographically. In this clade, the basal nodes are mostly weakly supported, while the posterior probabilities of nodes close to the crown tips increase.

In Clade B, the Early Cretaceous genera, *i.e.*, *Araripeberotha*, *Caririberotha*, and *Cantabroberotha* constitute a subclade, and as sister to the remaining Late Cretaceous and Cenozoic berothids. Most of the Late Cretaceous genera from Burmese ambers form a subclade. The sister relationship between *Ansoberotha* and *Cornoberotha* was well supported (Posterior Probability: 70%). Additionally, the Late Cretaceous *Nascimberotha* and *Plesiorobius* from North America and Europe are sister groups. All Late Cretaceous genera are recovered as stem-groups to the Cenozoic genera. The Cenozoic subclade was inferred to diverge from their sister at ~75 Ma. In this Cenozoic subclade, the Eocene *Elektroberotha* from Baltic amber is recovered as sister to all extant genera. Among the extant berothids, ((*Nyrma* + *Ormiscocerus*) + *Brothimerobius*) + *Tanzanberotha* are grouped with a posterior probability of 74%. (((*Austroberothella* + *Protobiella*) + (*Cyrenoberotha* + *Manselliberotha*)) + *Spiroberotha*) + (*Naizema* + (*Trichoberotha* + *Trichoma*)) are clustered with a posterior probability of 60%. *Nosybus* is sister to *Stenobiella*, which is supported by a posterior probability of 81%. *Berlekrumyia* and *Lekrugeria* are sister groups with a posterior probability of 100%, the two of which are sister to ((*Asadeteva* + *Isoscelipteron*) + *Nodalla*) + (*Berotha* + ((*Lomamyia* + *Spermophorella*) + *Podallea*)), supported by a posterior probability of 75%. Generally, in this clade, the basal nodes show generally low support, while the Cenozoic subclade has higher support, especially for the crown sister groups.

(2) Diversification assessment

To gain insights into the evolutionary history of beaded lacewings, we conducted a series of analyses based on the phylogeny and assessed the lineage diversity, net diversification rate, speciation rate and extinction rate changes through time, by applying the R package ‘phytools’ and BAMM v2.6.

The results of Lineage Through Time (LTT) reflect the diversity patterns of the family (Fig. 2*F*). Berothidae underwent two phases of diversification. The first phase happened from the Jurassic to the Early Cretaceous (178–100 Ma). The genera gradually accumulated during this phase and reached their first peak (13 genera) at the end Late Cretaceous. During this phase,

genera also became extinct from time to time. Most of the genera did not get through the Late Cretaceous, which resulted in a sharp drop of the LTT plot in the early Late Cretaceous, losing approximately 60% of genera. This is followed by a long-term slow accumulation during the Late Cretaceous and Paleogene. The beaded lacewings experienced their second phase of diversification during the Neogene and Quaternary.

Comparatively, the net diversification rate (NDR) generally maintained a low level before 80 Ma (Fig. 2*F*). The NDR rapidly increased after 80 Ma, especially during the Paleogene. From the Neogene, the NDR stayed at a high level. The low rate of net diversification in the early phases of their evolutionary history can be attributed to the interplay between a high rate of speciation and a high rate of extinction (*SI Appendix*, Fig. S21). This phenomenon is in accordance with the results of the phylogenetic tree and LTT plot.

3. Phylogenetic patterns evaluation

(1) Ecological niche changes

In order to obtain the evolutionary pattern of the ecological niche of beaded lacewings and the potential impact of phylogeny on the ecological niche variation, we tracked the physiological adaptation of the family by integrating ancestral-state reconstruction, phylogenetic signal, and evolutionary rate analyses. The relevant climate niche variables are all numerical continuous data. We used the R package 'phytools' to reconstruct their ancestral states based on the phylogeny. Phylogenetic signal of each niche variable was evaluated by R package 'phylosignal', referring to Pagel's λ and Blomberg's K. In order to detect the evolutionary patterns of different niche variables, we tested the evolutionary model fitness of each variable by the R package 'geiger'. The niche evolutionary rates were calculated using BAMM v2.6. Because most genera have more than one site or specimen, all the available data from their sites and specimens were collected and compiled. The tip value adopted the average value of each genus for each niche variable in the analyses.

The results show significant differences between the relevant temperature niche variables and the relevant precipitation niche variables. The phylogenetic signals of temperature factors are generally stronger than those of precipitation factors (*SI Appendix*, Table S2). Similar phenomena were also found in other insect groups (13, 14). No significant phylogenetic signal is observed in *H*-EL, *H*-DMP, *H*-EQ. Particularly, *H*-TS and *H*-CI show significant and strong

phylogenetic signals referring to both *K* and *λ*, suggesting phylogenetic niche conservatism (PNC). Besides, the results of ancestral-state reconstruction and evolutionary rate also reveal different variation trends and patterns among the relevant temperature niche variables and the relevant precipitation niche variables (*SI Appendix*, Figs. S7–S9).

The changes of habitat annual mean temperature (*H*-MAT) and the habitat mean temperature of the coldest month (*H*-CMT) through time show high similarities. *H*-MAT had been continuously increasing from its divergence (~ 12.33 °C) until 100 Ma (~ 29.7 °C). Subsequently, it decreased and stabilized around 18.94 °C. Likewise, *H*-CMT started from the initial ~ -2.78 °C, to the highest ~ 25.52 °C at 100 Ma, till stably maintaining around 13.41 °C. When projecting the habitable regions according to each ecological factor threshold on successive time bins, the results also show similar patterns and changes based on *H*-MAT and *H*-CMT (*SI Appendix*, Figs. S15 and S22). Unlike the two variables above, the habitat temperature seasonality variables, *i.e.*, *H*-TS and *H*-CI, show highly similar trends. They started from a high value (12.33 °C and 33.23 °C), then decreased and fluctuated until stabilized around 3.87 °C and 1.65 °C. The habitat mean temperature of the warmest month (*H*-WMT) also shows a rising trend from the initial 30.45 °C to 35.36 °C at 100 Ma, and then continuously decreasing until 24.07 °C. All the relevant temperature variables have high evolutionary rates in the Jurassic, and then gradually declined through time. Our analyses find that the early burst model (EB) best described the data of *H*-MAT, *H*-TS, *H*-CI, *H*-CMT, indicating that the evolutionary rate was initially high and decreased exponentially over time. And the Ornstein-Uhlenbeck model (OU) best described the data of *H*-WMT. Indeed, *H*-WMT shows a different evolutionary pattern from the others. After reaching a peak, the overall value of *H*-WMT steadily decreased, rather than stabilized, as the other variables. This indicates that beaded lacewings are likely intolerant to extremely high temperature, which is compatible with the inference from the causality concerning EHT.

Unlike the relevant temperature factors, the relevant precipitation factors show different evolutionary patterns. The habitat annual precipitation factor (*H*-MAP) has two peaks respectively during the Early Cretaceous (1647 mm) and K-Pg boundary (1417 mm), with their initial and modern values as 747 mm and 870 mm. The changes in habitat precipitation of the wettest month (*H*-WMP) show a similar process to that of *H*-MAT, starting from 105 mm,

reaching the top of 314 mm at 100 Ma, and staying around 166 mm in the recent periods. The habitat precipitation of the driest month (*H*-DMP) started from 20.28 mm, reached its peak of 52.53 mm at 54 Ma, later than the other factors, and then decreased gradually to 16.88 mm. The habitat precipitation seasonality (*H*-PS) started from 45.42 mm, reached the peak after 50 Ma of 81.29 mm, then fluctuated to 75.90 mm. The habitat Ellenberg quotient (*H*-EQ) shows generally increasing from the initial 27.14 °C/mm to 163.45 °C/mm. The weak and non-significant phylogenetic signals and relatively frequent changes of habitat relevant precipitation factors within clades imply rapid adaptability or resilience to precipitation conditions of the beaded lacewings. This is also agreeable with the habitable region mapping according to *H*-DMP, which reveals significantly larger regions than those based on the relevant temperature factors, although with similar changing trend (*SI Appendix*, Fig. S23). In the Mesozoic, most habitable regions were located in the mid to high latitudes, with a few isolated regions at low latitudes. The regions gradually expanded during the Cenozoic, but were still mainly distributed in the mid to high latitudes. The mapping on the modern earth covers all the continents today, suggesting that the extant beaded lacewings are suitable for large ranges of precipitation. The evolutionary rates of all relevant precipitation variables did not change as extensively as the relevant temperature variables. Our analyses find the Ornstein-Uhlenbeck model (OU) fit best the data of *H*-MAP, *H*-DMP, *H*-PS. This suggests that these variables are likely to evolve in a fluctuating pattern around an optimal threshold. In contrast, the Delta (DT) model is the best-fitting model for the datasets *H*-WMP and *H*-EQ. This suggests that these two variables have evolved more rapidly in recent times.

Additionally, the habitat elevation (*H*-EL) changed extensively. It started from 430 m, reached two peaks during the Early Cretaceous (605 m) and the Paleogene (765 m), and stably ended at a relatively high level of 615 m. Different from the other factors, the *H*-EL dropped to the lowest of 374 m at 100 Ma. Its evolutionary rate reveals high rates at the initial, the Early Cretaceous, the Paleogene, and the final phase. The White Noise model (WN), *i.e.*, the non-phylogenetic model is found to best fit the data of *H*-EL, which agrees with its non-significant phylogenetic signal.

Indeed, mountains provide rich ecological opportunities and are hailed as the cradle of biodiversity. The LTT plot of beaded lacewing unveils a sharp increase of their diversity in the

last 25 Myr, namely the second radiation (Fig. 2*F*), which is coincident with the orogeny of the Himalayas (15). The analyses of ancestral-state reconstruction and the evolutionary rates of habitat elevation of berothids (*H*-EL) jointly demonstrated the association of the adaptation and biogeography of beaded lacewings with elevation, particularly exhibited by several living genera (*SI Appendix*, Figs. S7–S9). These beaded lacewings are endemic to the Himalayan region. *Asadeteva* with the highest elevation at 3567 m and *Nodalla* at 4042 m, are distributed in the western region of the Himalayas, while *Berotha* (1756 m) and *Lekrugeria* (963 m) in the southern region. Among them, the habitat elevation evolution rate of *Asadeteva* is higher, while the other three genera are close. According to previous paleoaltimetry results, the Himalayan - India Asian suture's elevation during the early Miocene (23 Ma) was approximately 2200 m (16–18), while in the late Miocene (7 Ma), its elevation was approximately 5550 m (19–21). These four genera adapted to the mid to high-elevation regions of the Himalayas have increased the evolution rate of the habitat elevation of beaded lacewings since 25 Ma. At the same time, it is also a manifestation of Berothidae adapting to new environments along with diversification. The uplift of the Himalayas region resulted in similar climate conditions to those in mid latitudes (*SI Appendix*, Figs. S15 and S22: 30 Ma). Consequently, the Himalayan region provided ecological opportunities in lower latitudes for beaded lacewings that prefer cooler environments (Fig. 2 *D* and *E*). Accordingly, the formation of mountainous terrains offers abundant climatic conditions, enabling the berothid genera to adapt to high-elevation environments in low latitudes. The results elucidate biodiversity stimulation by ecological niche differentiation along vertical stratification.

It is noticeable that the phylogenetic relationships of Berothidae exhibit some nodes with low support. This may give rise to intricate evolutionary issues. The evolutionary pattern of the clades with low support nodes may affect the interpretation of their ancestral-state reconstruction results. Two low-supported nodes were identified as posing challenges to state variation history. They are the divergence of *Pseudosisyra* from its sister group at 150.77 Ma and the divergence of *Xiaoberotha* from its sister group at 110.9 Ma. These two nodes exhibited an evolutionary pattern shift in nearly all habitat environments. It is noteworthy that if these two nodes turn to polytomous nodes due to low support, it may result in a change in the evolutionary pattern from a single peak (trough) to a monotonically changing pattern. This may

similarly arise in biogeographic analyses, resulting in higher uncertainty on the dispersal time of the corresponding events. However, the aforementioned problems have a minimal impact on other clades because their states are more conservative around the nodes.

(2) Wing morphological divergence

Both wing length and wing shape show significant phylogenetic signals, of which the wing length has a relatively stronger signal (*SI Appendix*, Table S2). The OU model is found to best describe the data of wing length, suggesting that wing length is likely to evolve in a fluctuating pattern around 6.24 mm. In contrast, the DT model provides the best fit for that of wing shape, indicating that wing shape has evolved more rapidly in recent times. The ancestral-state reconstruction reveals that the wing length of Berothidae, although fluctuating all the time, generally increased throughout their evolutionary history. The continuous elongation of wing length has the potential effect of enhancing flight efficiency (22). The rate of wing length change displays generally stable change. Overall, the beginning phase shows a higher rate than the latter time, with an exceptionally rapid rate stage during the J-K boundary. Since the late Early Cretaceous, the rate of wing length change retained a lower level, but increased slightly through time.

The ancestral-state reconstruction of wing shape revealed the primary state of berothids wings to be oval-shaped. The earliest falcate wing was derived before the mid-Cretaceous but the majority of falcate wings burst onto the scene in the Cenozoic, with the earlier representative, the Paleogene *Xenoberotha* and the latter extant ones mainly clustered in two lineages, which account for 50% of extant beaded lacewings.

To investigate the relevant differentiation of wing shape divergence, we compiled the wing length, habitat climate factors and atmospheric conditions data of all living berothid records and performed statistical analyses. The results find significant differences in all the variables between the two wing shape types, including wing length, *H*-MAT, *H*-MAP, airflow velocity, air density and air viscosity. The falcate wings are mostly longer than the oval-shaped wings. The habitats of berothids with falcate wings are characterized by lower *H*-MAT, airflow velocity, air viscosity, and higher *H*-MAP, air density. Consequently, we infer that the wing shape of berothids has evolved in tandem with wing length, plausibly jointly driven by aerodynamic efficiency.

Phylogenetic generalized least squares analyses (PGLS) were conducted using R package 'caper' on wing shape (*SI Appendix*, Fig. S11)/wing length (*SI Appendix,* Fig. S12) to habitat environmental variables, yet the results did not demonstrate a significant correlation. This may be attributed to the data limitation as PGLS were analyzed on the terminal taxa of the tree, *i.e.*, 52 genera, compared to the individual specimen data, which consists of 1174 data points. Otherwise, it is plausible because that the environmental differences between wing shapes reveal independent adaptation among the insects, other than shared evolutionary history.

4. Computational fluid dynamics

(1) Transient analyses

To investigate the differences in the flight performance of berothids across diverse atmospheric conditions, we executed transient simulations under different environmental features. For the fluid domain, we established boundary conditions by combining the maximum or minimum of each atmospheric variable with the mean values of the other two, resulting in six comparable field setting scenarios (Fig. 4*A* and *SI Appendix*, Table S3). During the modeling, one entire cycle of stroke was performed on one forewing of each wing shape, while the body kept static and the body-to-wing ratio remained constant. The median length of each wing shape type was used. Additionally, one oval-shaped longer wing, equal to the median length of falcate wings was modeled too, considering the larger wing length range of the oval-shaped wings. Consequently, totally three forewings were modeled. Pre-experiments were conducted in which the time step size was set to 0.001 and 0.01 seconds separately. The results demonstrated no significant difference between them. For consideration of computational efficiency, during the final modeling, a sufficiently small time step size (0.01 s) was set to ensure convergence at each time step. The number of max iterations within each time step was kept at 20. The maximum lift-to-drag ratio over an entire stroke cycle of each wing under each field scenario was extracted (*SI Appendix*, Table S4). The results find severe influence of airflow velocity among the atmospheric conditions. Both oval-shaped and falcate longer wings show distinctly higher maximum lift-to-drag ratios, with only slight differences between them. This suggests that, within the spectrum of berothid wing lengths, elongated wings confer a significant advantage in lift-to-drag ratio during flight performance.

Furthermore, the number of dispersal events over time was extracted from the BSM

analyses and combined with the results of ancestral-state reconstruction related to wing length to perform hidden Markov modeling and logistic regression analysis. The results indicated a significantly positive correlation between wing length and the number of dispersal events per million years (Fig. 5 *A* and *B* and *SI Appendix*, Fig. S16), with a turning point, particularly when wing length exceeded 6.69 mm. This indicates that the population would undergo a greater number of dispersal events as wing length increased, which is likely attributed to the enhanced flight performance resulting from the elongation in wing length.

(2) Steady analyses

To test the aerodynamic efficiency differences at explicit angles-of-attack, we performed steady analyses between the two wing shapes regarding the lift-to-drag ratios and drag coefficient. For the fluid domain condition, we used the mean values of airflow velocity, air viscosity and the minimum value of air density for a relative adverse circumstance. A series of modelings were performed at angles-of-attack ranging from 2° to 36° for the wings of the two shapes. We modeled one forewing as representative of each wing shape, with an equal length of 9 mm. When the analyses achieved convergent, the results show that the lift-to-drag ratios of the oval-shaped wings are slightly higher than that of falcate wings (Fig. 4*F*). However, the drag coefficient shows noteworthy differences, which is consistently lower for the falcate wing than the oval-shaped wing across all angles-of-attack (Fig. 4*G*). Compared to the percentage difference, the drag coefficients are much more significant than the lift-to-drag ratios (*SI Appendix*, Fig. S13 and Table S5). The results suggest that the evolution of the falcate wing likely represents a strategic adaptation during the late phase of beaded lacewing evolution. Benefiting from a stably lower drag profile, the berothids with falcate wings potentially have advanced flight capability for long-distance flights, from an aerodynamic perspective. Subsequently, we added energy equations to construct fields of different air temperatures. We used the median value of habitat MAT of both wing shapes and their corresponding atmospheric variable values as boundary conditions. Steady analyses were performed for both wing shapes under the series of angles-of-attack. The results show that the falcate wing exhibits a more pronounced low-drag effect at relatively low temperatures (*SI Appendix*, Fig. S14 and Table S6). This is evidenced by the result that at relatively lower temperatures, the disadvantage of the lower lift-to-drag ratio of falcate wings is effectively minimized as the angle-of-attack is

varied. Whereas the advantage in terms of energy consumption at low drag consistently increases. Additionally, the berothids with oval-shaped wings reveal an evolutionary trend of increasing wing length, from which they gradually acquired advantages in lift-to-drag ratio and stronger flight capabilities compared to the falcate wings.

Figures and Tables

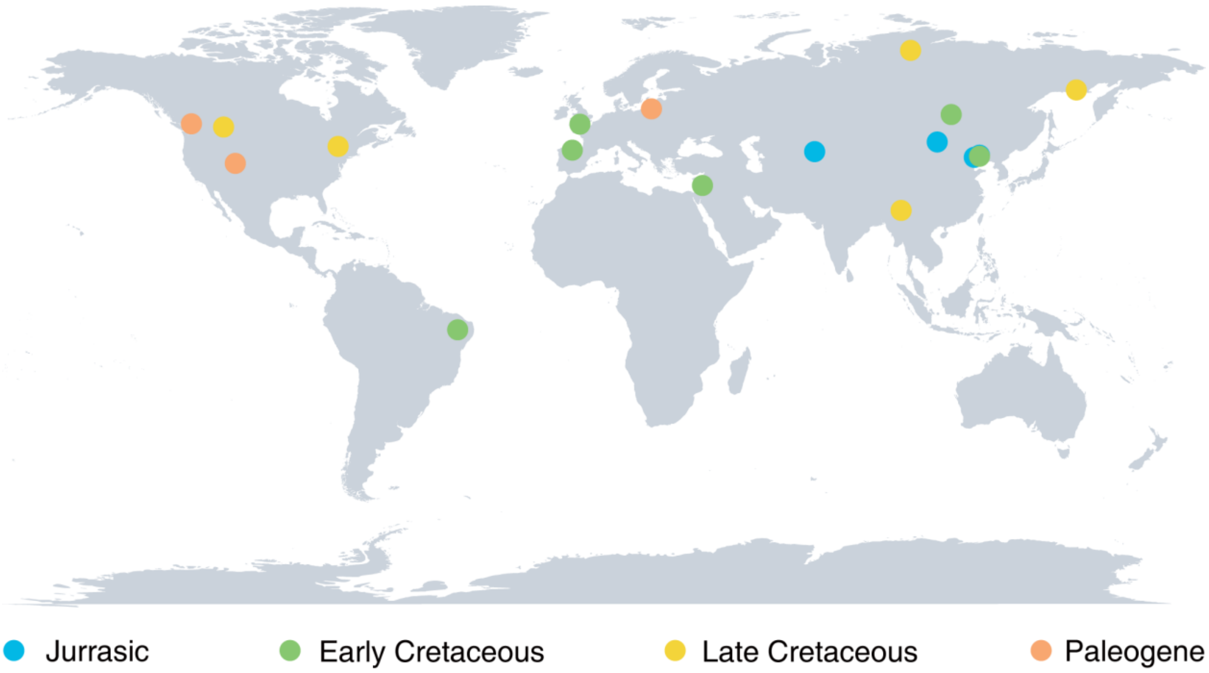

Fig. S1 Distribution of fossil berothids on the present geographic map. Colors represent different periods.

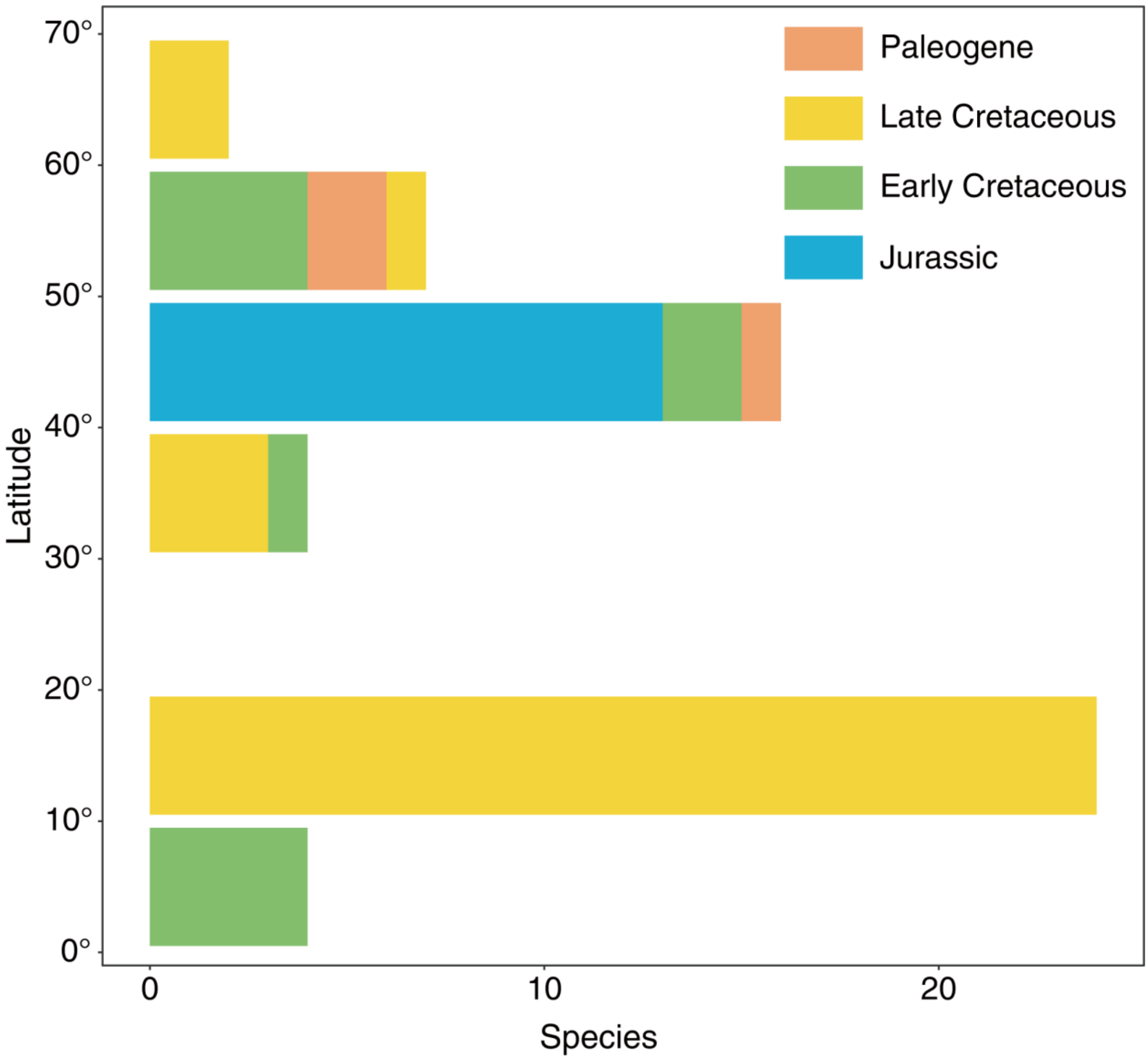

Fig. S2 Latitudinal distribution of fossil berothids according to their paleocoordinates.

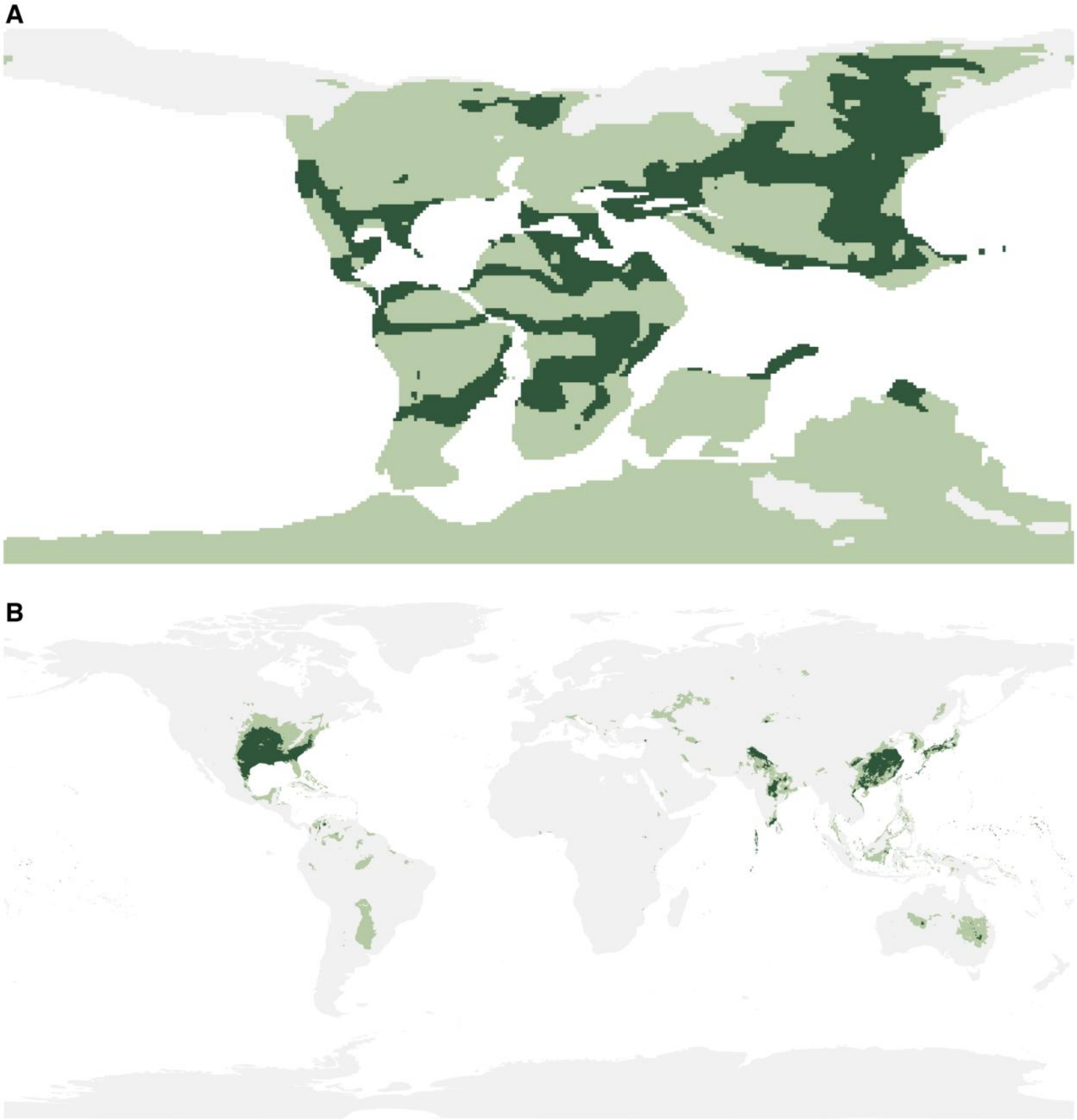

Fig. S3 Ecological niche modeling of Mesozoic berothids (A) projected on paleogeography and paleoclimate at 100 Ma, and **(B)** projected on modern earth and climate conditions. Dark green regions indicate suitable habitats ($p > 0.5$); light green regions indicate less suitable habitats ($0.3 < p < 0.5$); grey regions indicate unsuitable areas ($p < 0.3$).

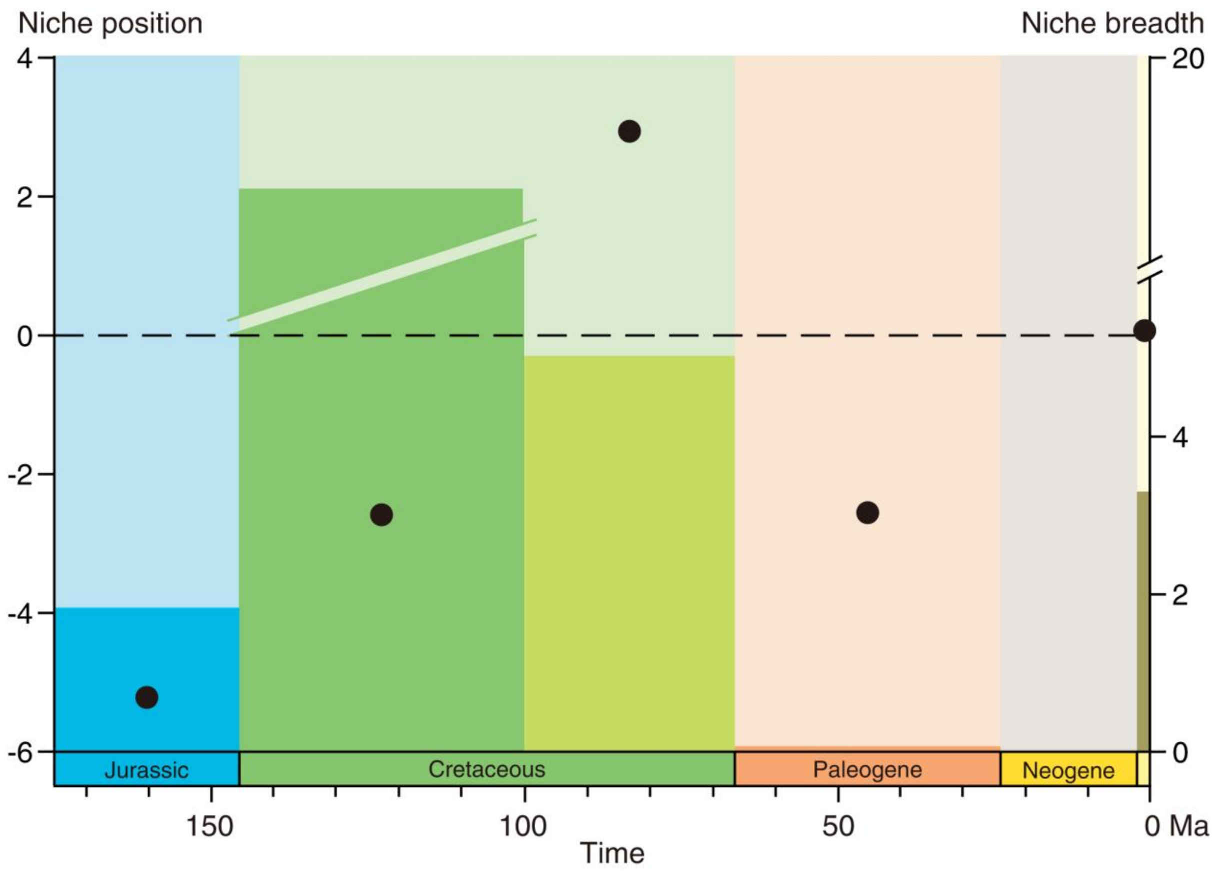

Fig. S4 Niche breadths and positions of Berothidae in five geochronological intervals based on fossil and extant species data. Bar chart illustrates niche breadth (referring to right vertical axis) while the scatter chart represents niche position (referring to left vertical axis). Grey area indicates periods with no fossil data available.

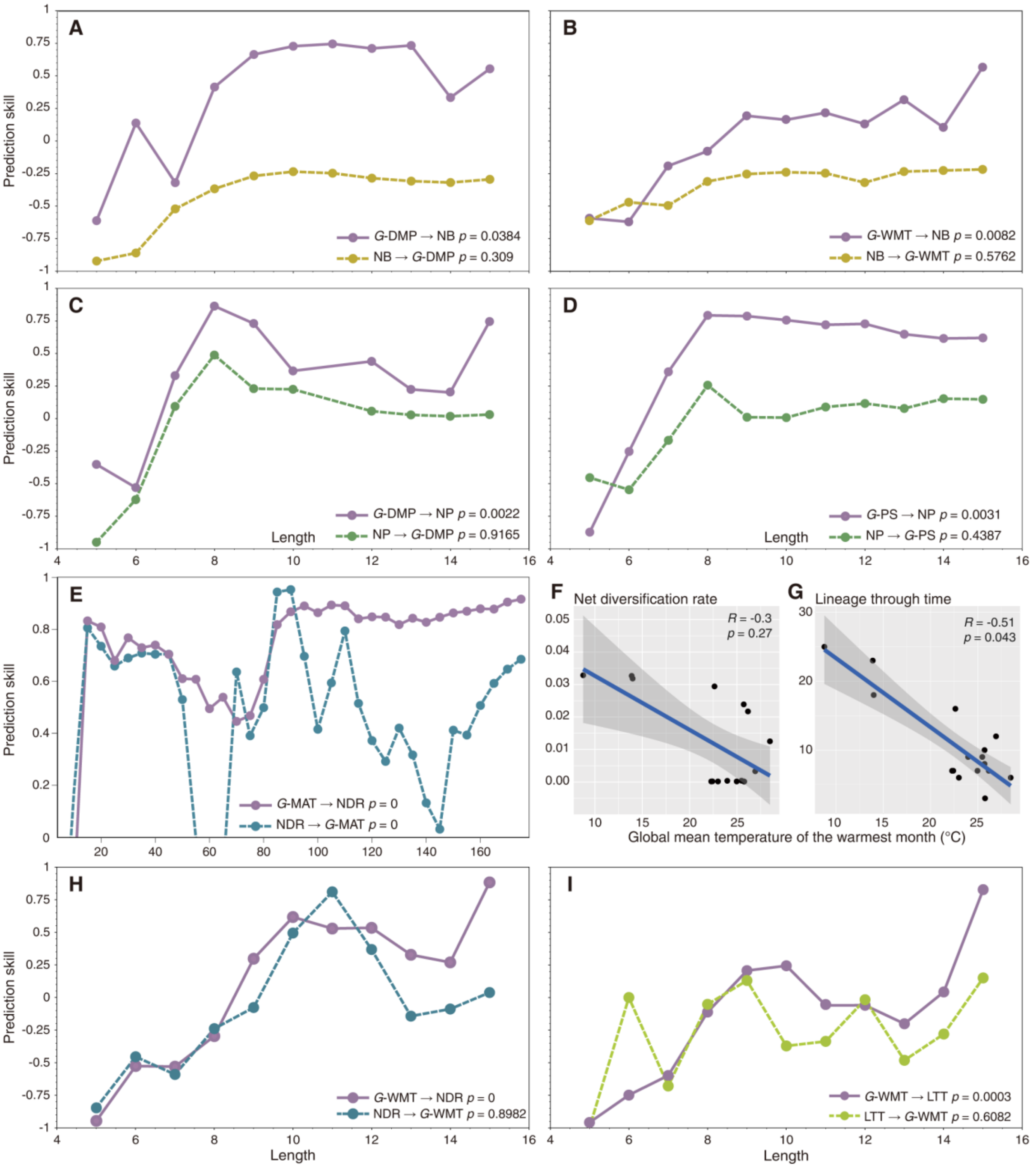

Fig. S5 Causal inference and correlation analyses between global climate variables and niche changes/diversification of Berothidae. Causal inference **(A)** between global precipitation of driest month (*G*-DMP) and niche breadth (NB); **(B)** between global mean temperature of warmest month (*G*-WMT) and NB; **(C)** *G*-DMP and niche position (NP); **(D)** between global precipitation seasonality (*G*-PS) and NP; **(E)** between global annual mean temperature (*G*-MAT) and net diversification rate (NDR). Spearman correlation coefficient **(F)** between *G*-WMT and NDR; **(G)** between *G*-WMT and lineage through time (LTT). Causal inference **(H)** between *G*-WMT and NDR; **(I)** between *G*-WMT and LTT.

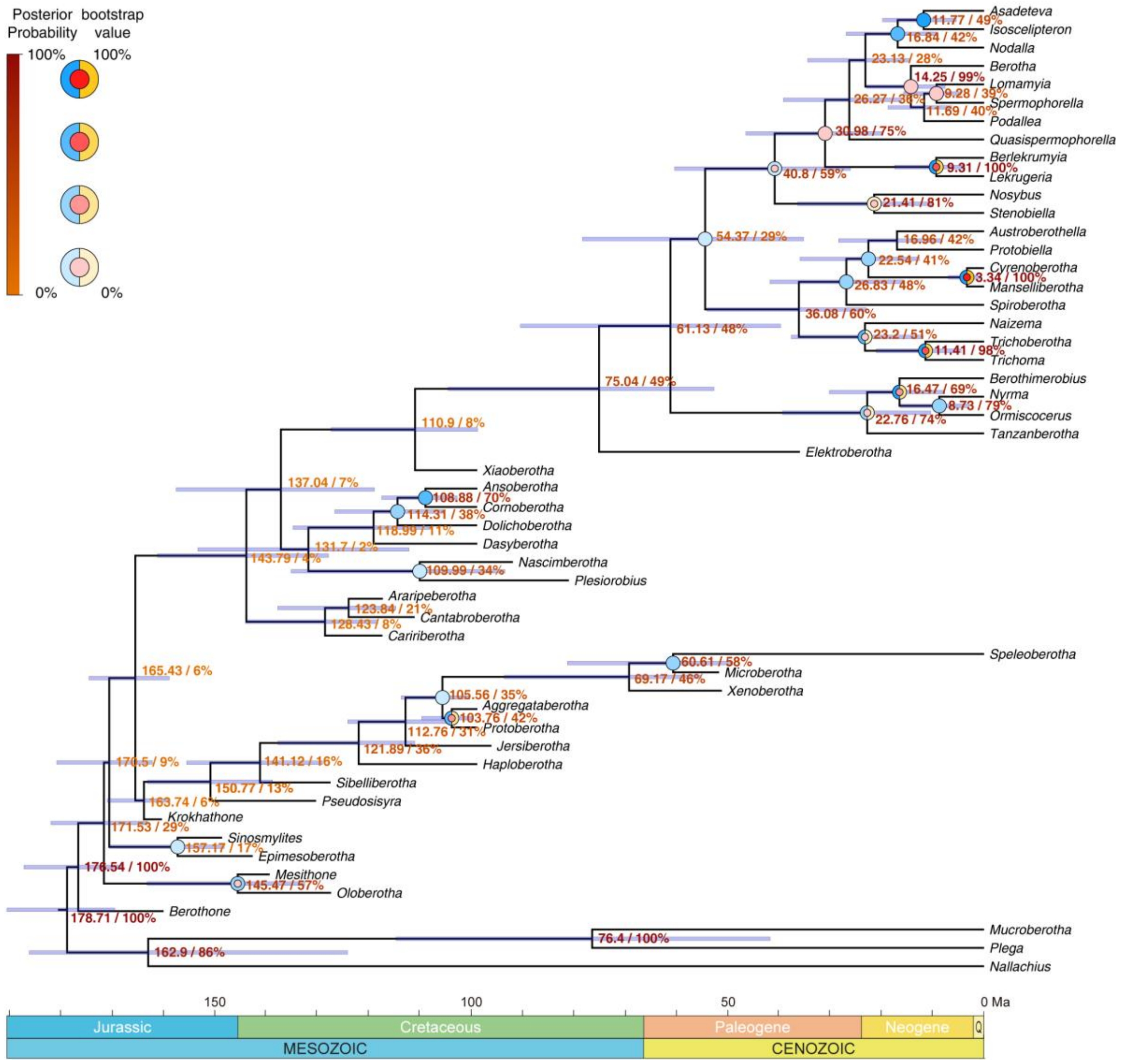

Fig. S6 Time-calibrated phylogeny of Berothidae. Maximum compatible tree from Bayesian phylogenetic tip-dating analysis using morphological matrix. The nodes are labeled by the median estimates of divergent times (Ma) and the posterior probabilities, which are separated by "/", with color intensity indicating the magnitude of posterior probabilities. The node bars are age intervals of 95% highest posterior density. Blue (semi)circles: nodes supported by Bayesian inference using combined matrix of morphological and molecular data, with their saturation representing posterior probability; yellow (semi)circles: nodes supported by the maximum parsimony analysis with combined matrix; red circles: nodes supported by the maximum parsimony analysis with morphological data. The saturation of yellow and red circles represents the bootstrap value of the nodes.

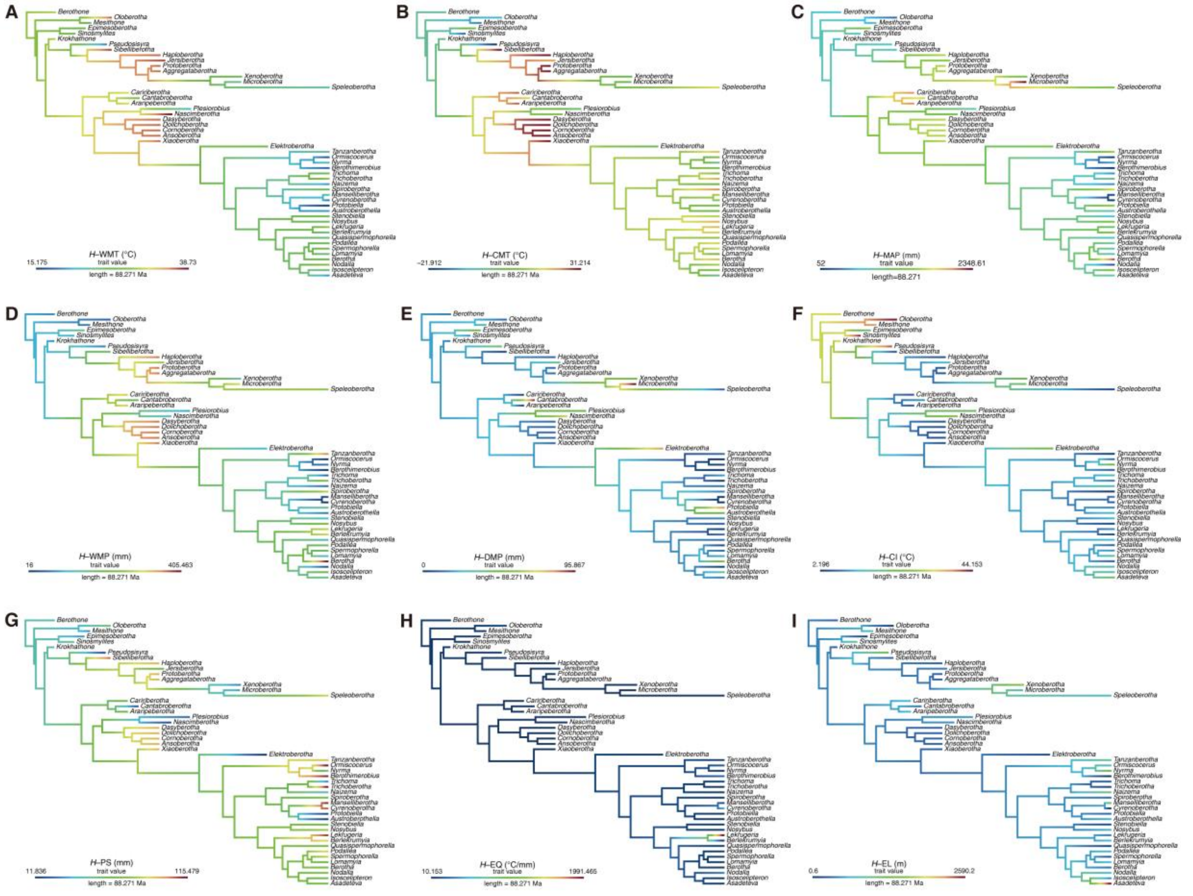

Fig. S7 Ancestral-state reconstruction of ecological niche factors. Colors indicate state of different ecological niche factors.

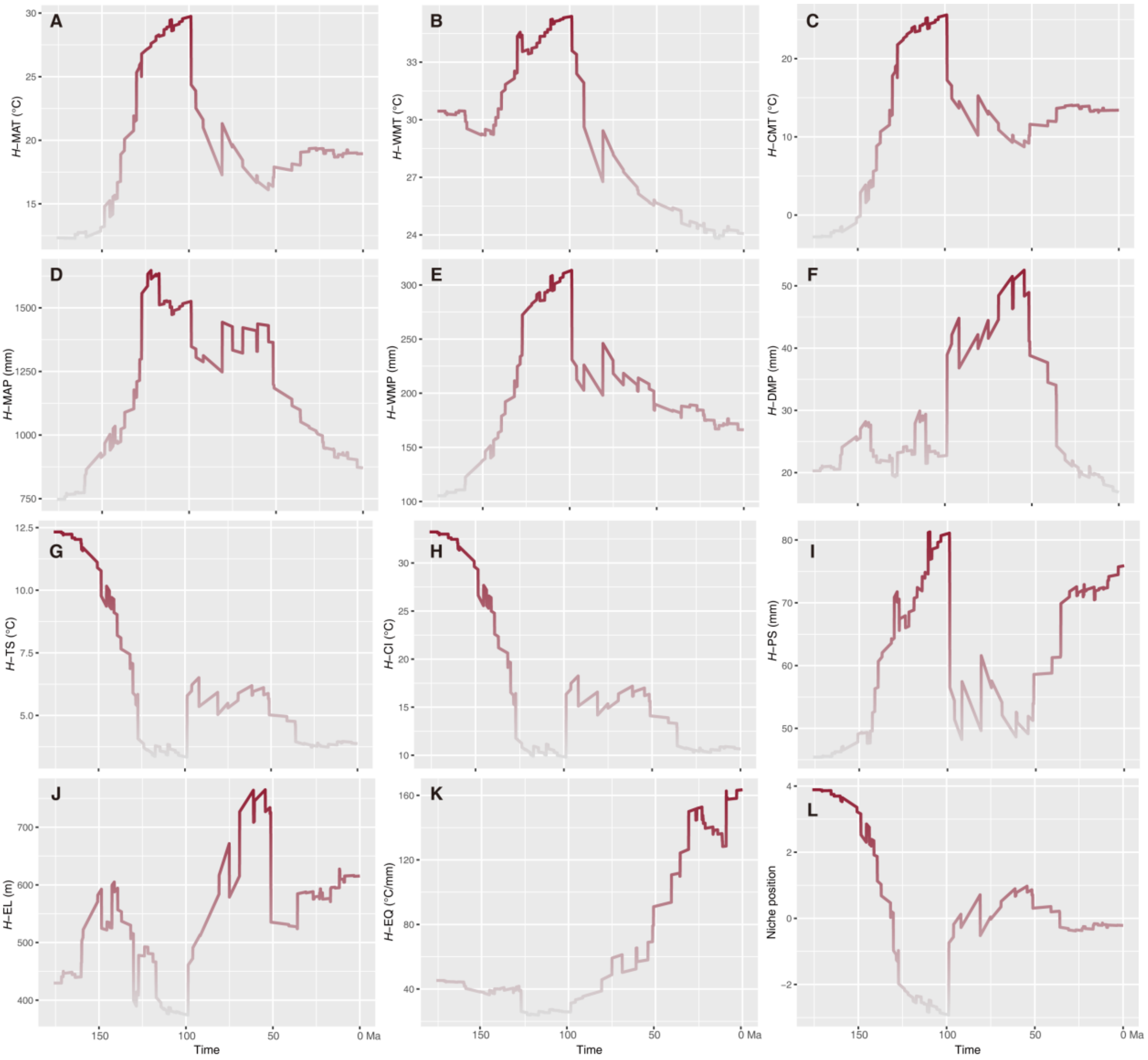

Fig. S8 Ecological niche factors' average state changes through time based on ancestral-state reconstruction. The horizontal axis indicates geological time (Ma). The vertical axis represents the mean values of ecological niche factors.

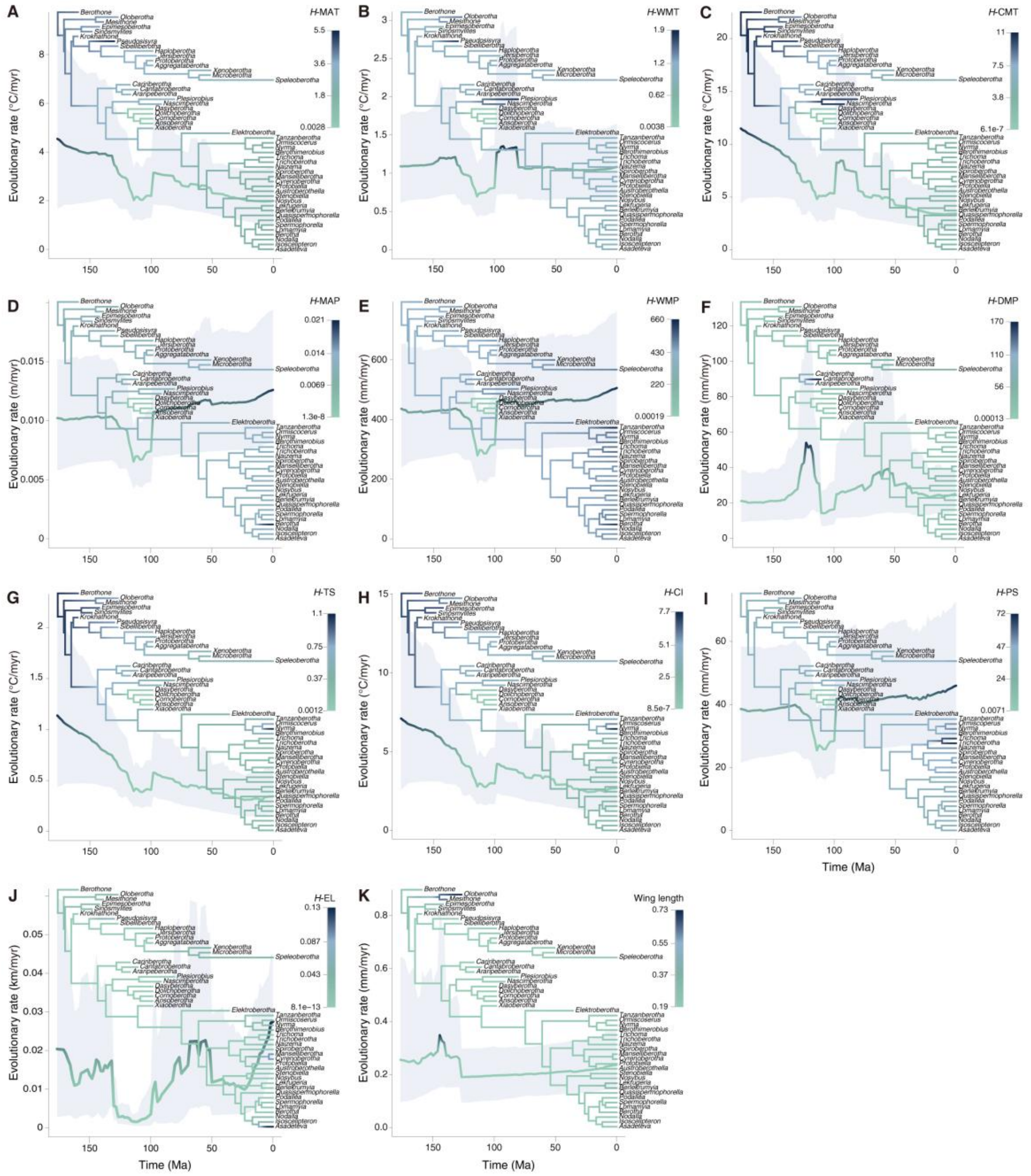

Fig. S9 Evolutionary rate variation of different ecological niche factors. The horizontal axis indicates geological time (Ma). The vertical axis represents the evolutionary rate of different ecological niche factors. Colors within phylogenetic relationships indicate evolutionary rates on the branches. Curve represents the average evolutionary rate per million years, with the grey area indicating the 95% confidence interval.

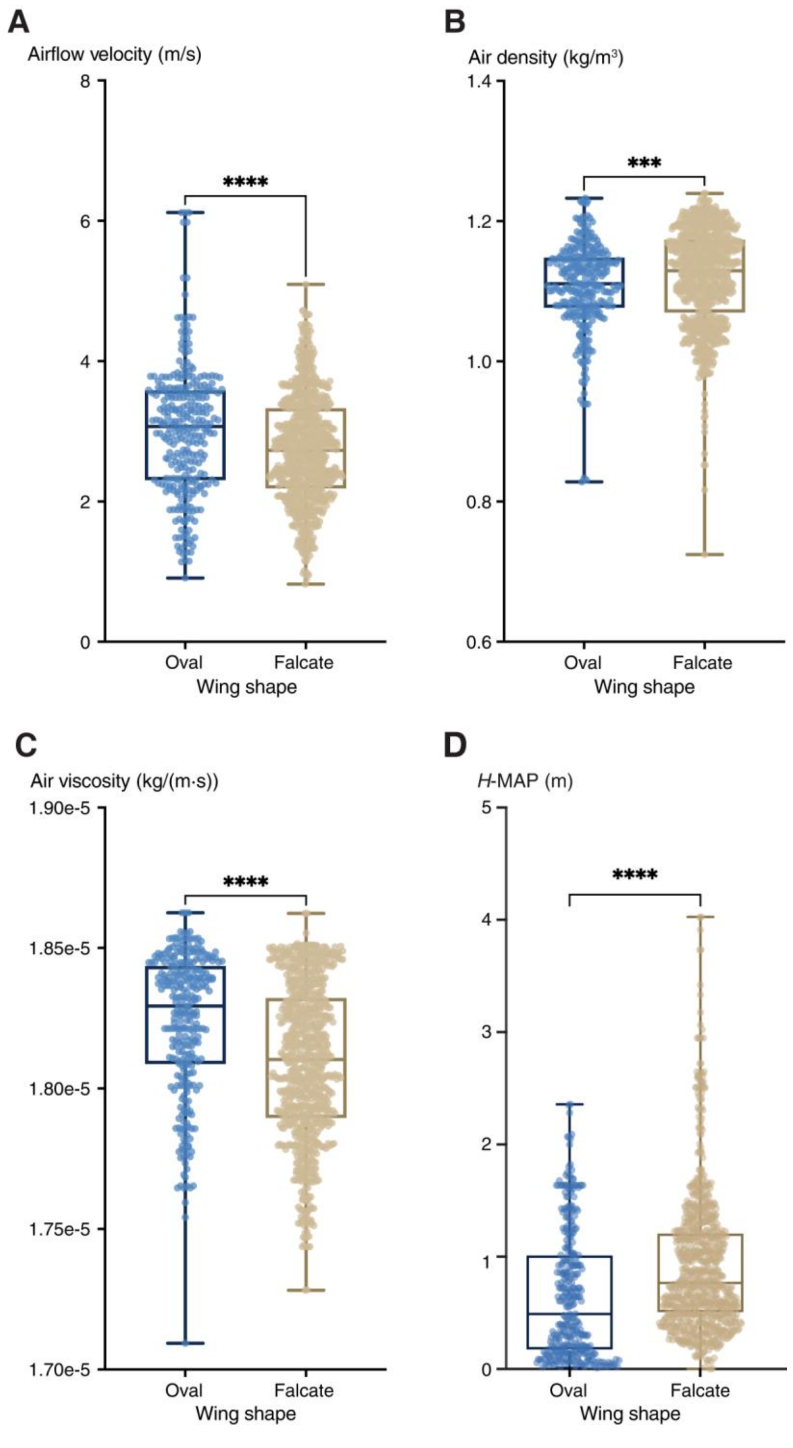

Fig. S10 Mann-Whitney tests of habitat environmental variables between two wing shapes. Mann-Whitney tests of **(A)** airflow velocity (m/s); **(B)** air density (kg/m^3); **(C)** air viscosity (kg/(m·s)) and **(D)** *H*-MAP (m) between oval-shaped wings and falcate wings berothids. (*** $p < 0.001$; **** $p < 0.0001$).

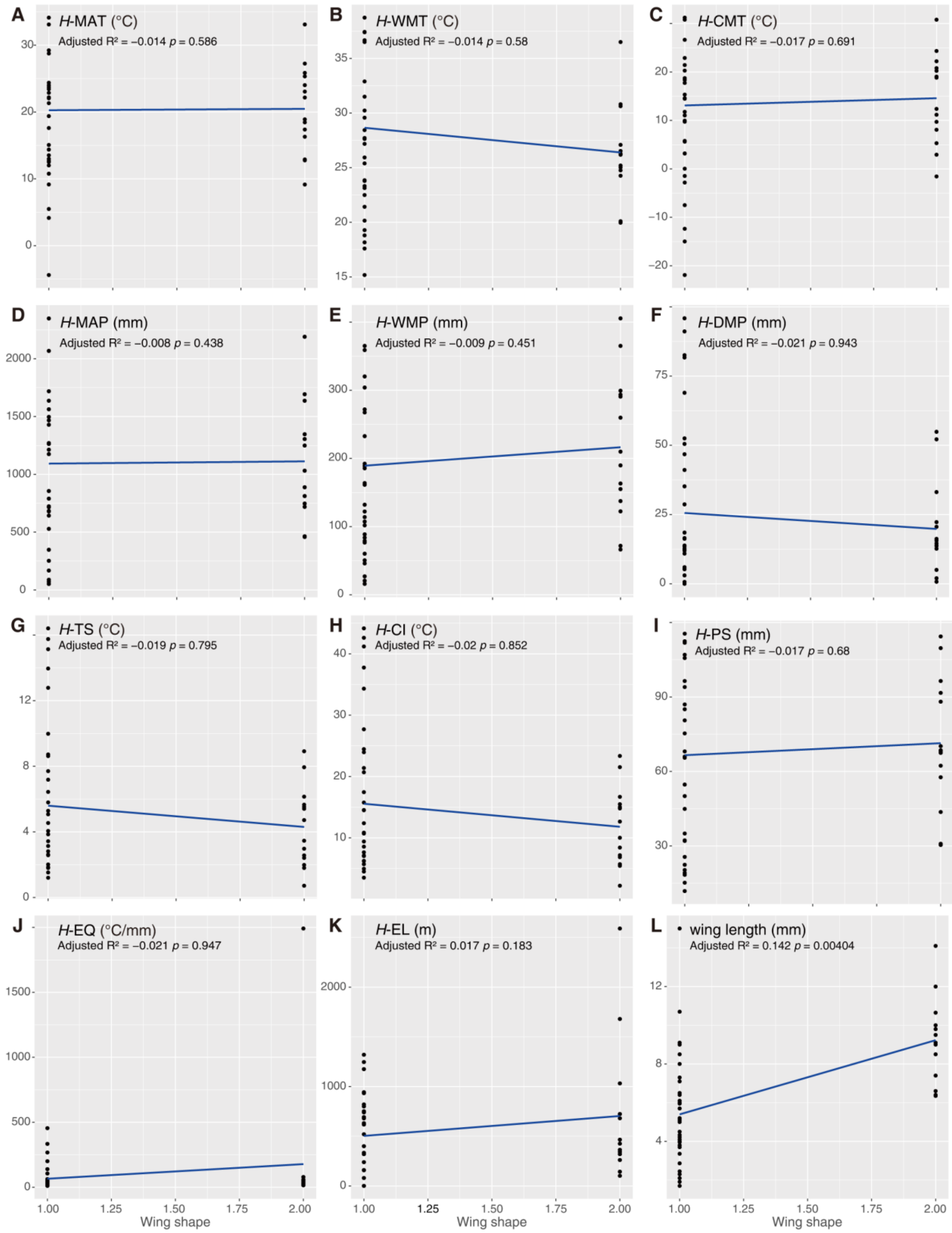

Fig. S11 Phylogenetic generalized least squares analyses of wing shape and environmental variables. The horizontal axis represents the different wing shapes. Oval-shaped wings valued as 1, falcate wings valued as 2. The vertical axis indicates ecological niche factors or wing length. The adjusted R^2 penalizes the addition of irrelevant predictors. *p* means significance.

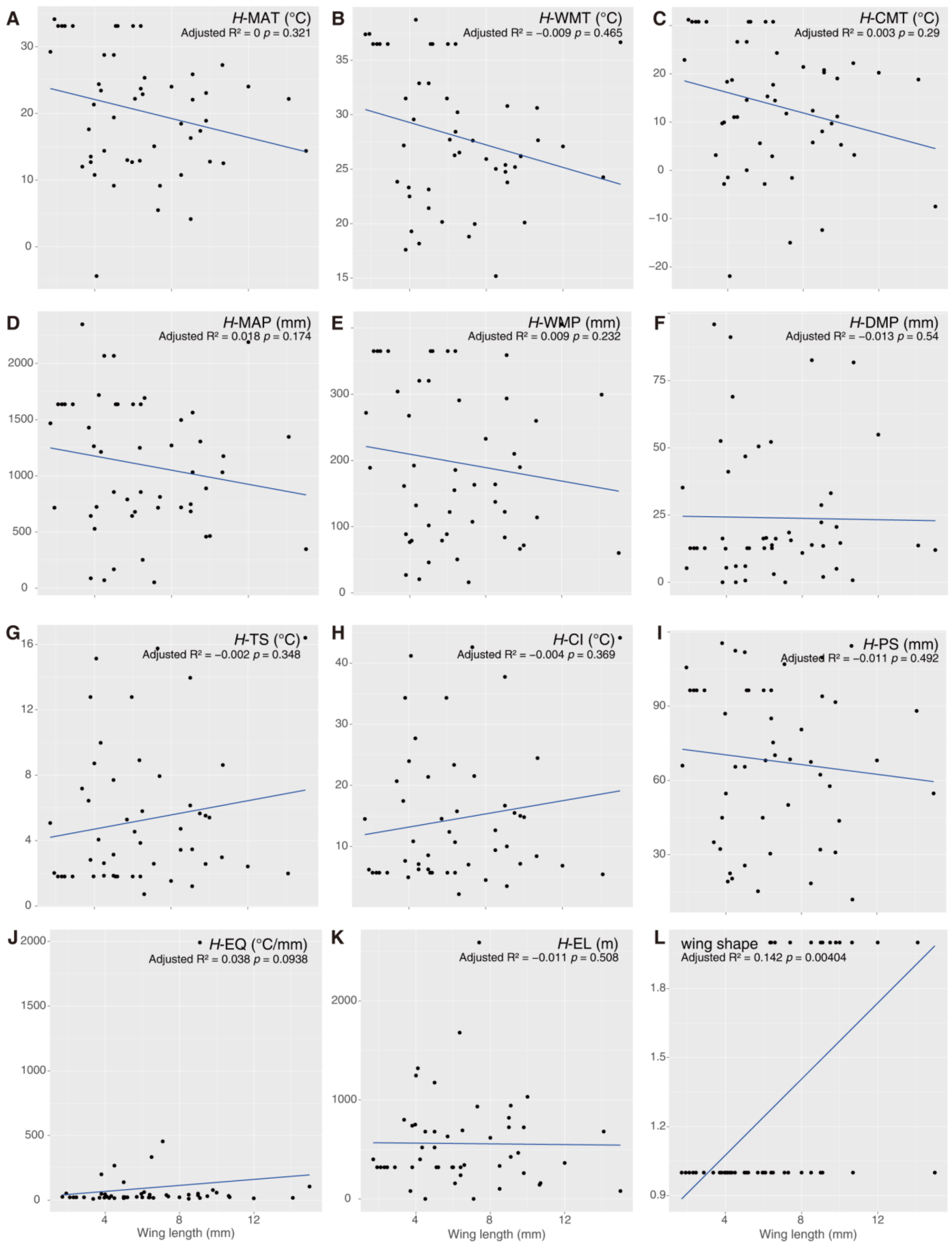

Fig. S12 Phylogenetic generalized least squares analyses of wing length and habitat environmental variables. The horizontal axis indicates wing length (mm). The vertical axis indicates ecological niche factors or wing shape. The adjusted R^2 penalizes the addition of irrelevant predictors. p means significance.

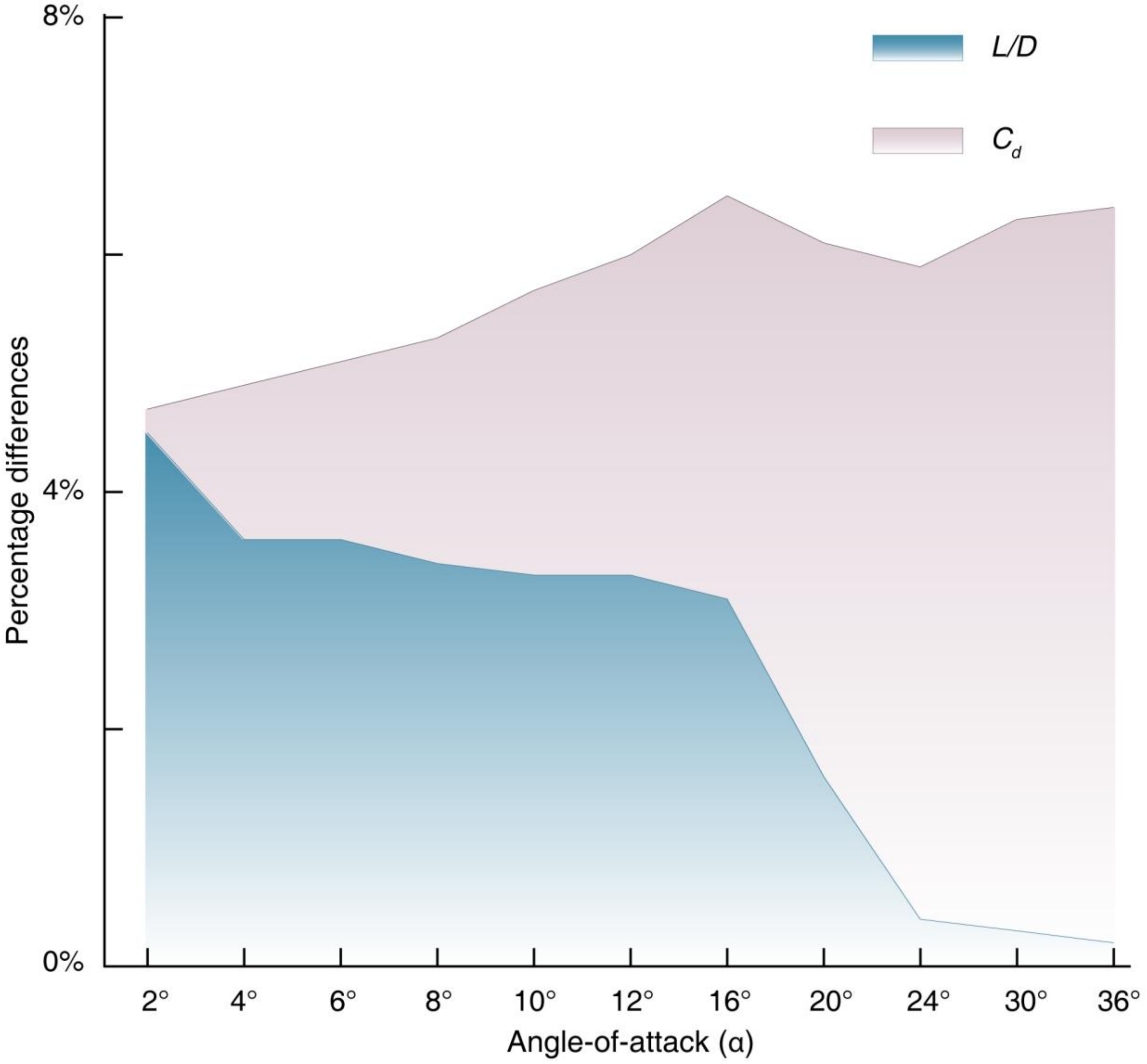

Fig. S13 Percentage differences of lift-to-drag ratios (*L/D*) and drag coefficients (C_d) between two wing shapes at various angles-of-attack. The horizontal axis represents eleven angles-of-attack, ranging from 2° to 36°. The vertical axis represents the percentage difference values between the two wing shapes, with light blue representing *L/D* and light pink representing C_d.

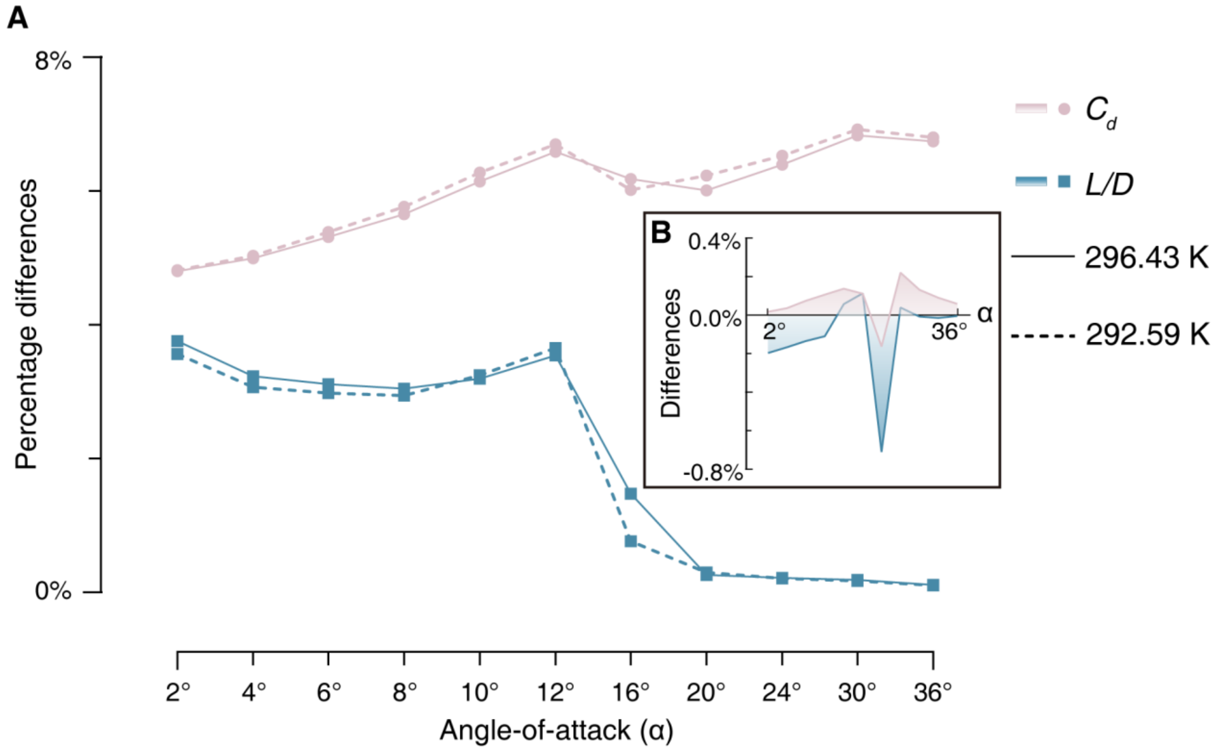

Fig. S14 (A) The percentage differences of lift-to-drag ratios (*L/D*) and drag coefficients (C_d) between two wing shapes at various angles-of-attack in certain temperature field. The horizontal axis indicates eleven angles-of-attack, ranging from 2° to 36°. The vertical axis indicates the percentage difference values between the two wing shapes, with blue line representing *L/D* and pink line representing C_d. The solid line indicates the temperature of 296.43 K and the dashed line is 292.59 K. **(B) Differences enlargement in drag coefficients and lessening in lift-to-drag ratios between falcate and oval-shaped wings in the lower temperature field (292.59 K) compared to higher temperature (296.43 K) through varied angles-of-attack.** The vertical axis indicates the value of the dashed line minus the value of the solid line of each aerodynamic index in (A). The horizontal axis represents angles-of-attack ranging from 2° to 36°. Blue shade represents the difference of *L/D* in (A), and pink shade represents the difference of C_d in (A).

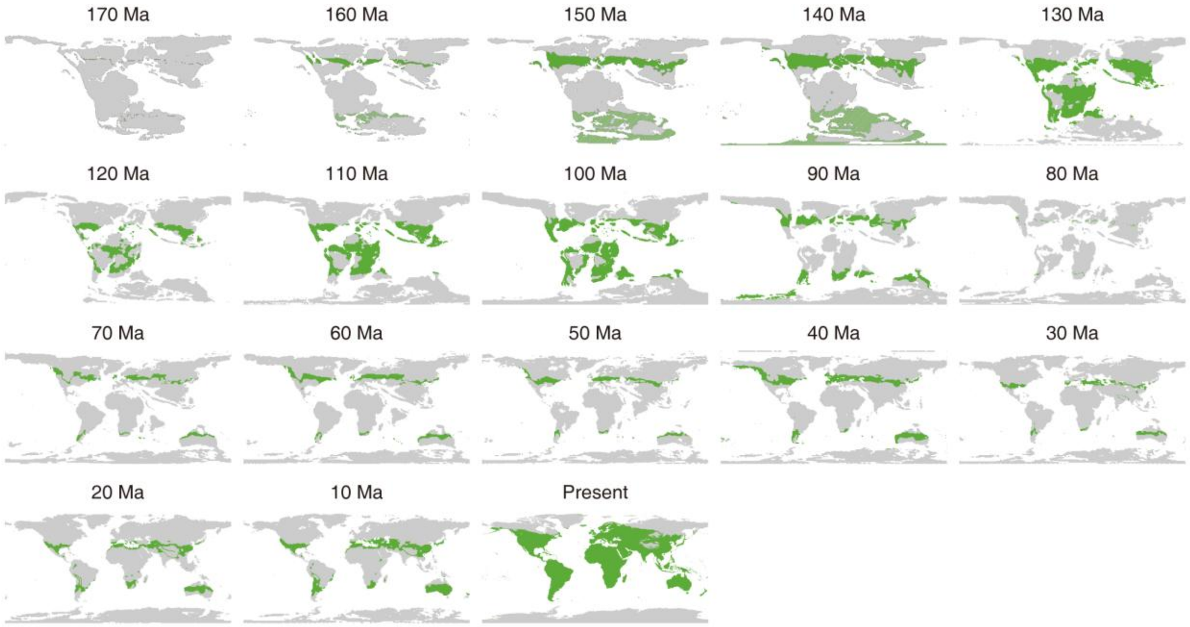

Fig. S15 Projection of potential habitable area (in green) for Berothidae based solely on *H*-MAT threshold through time. The masked green area indicates area with suitable *H*-MAT but not reachable at the time bin.

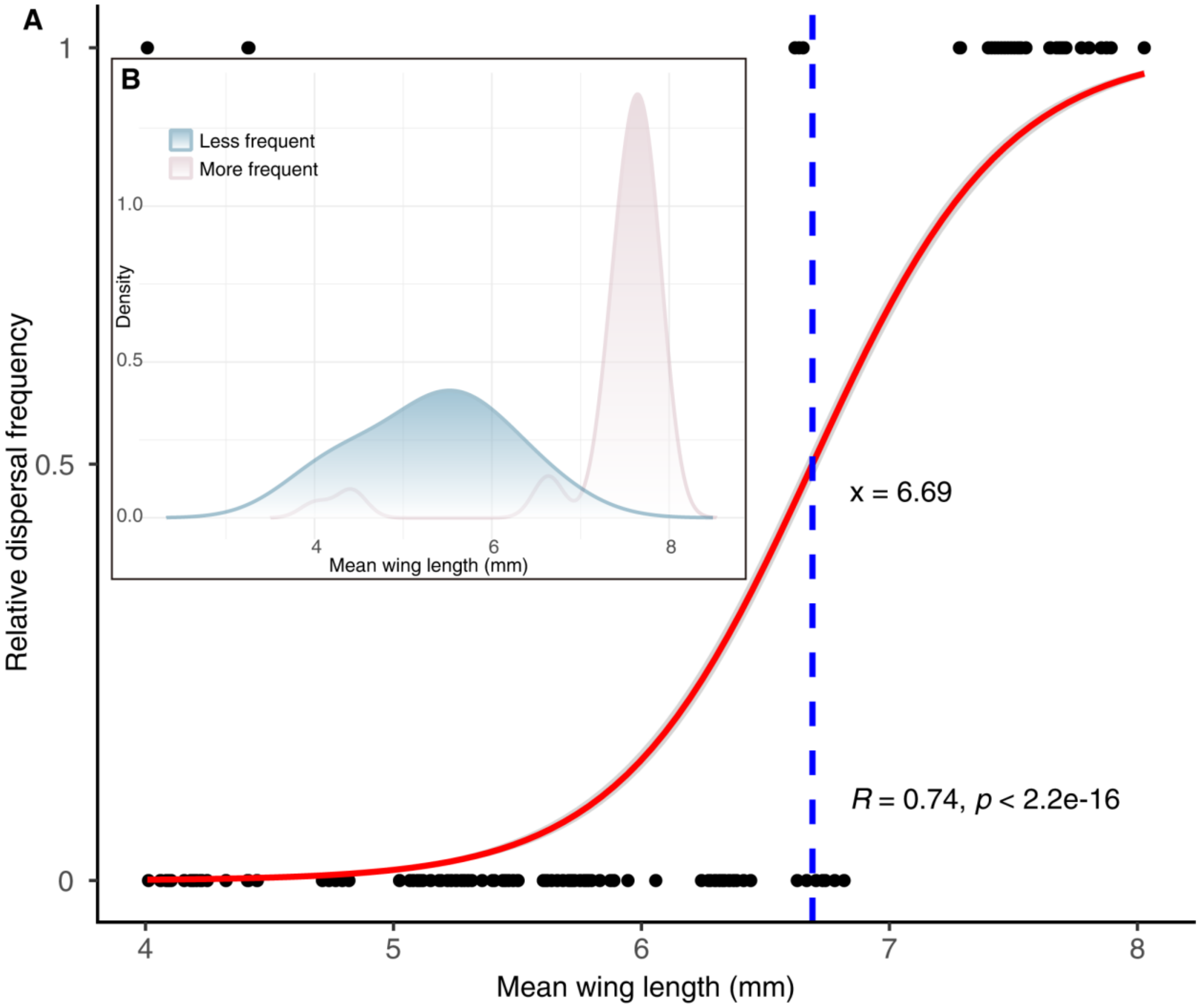

Fig. S16 (A) Logistic regression analysis of mean wing length by relative dispersal events per million years. The vertical axis represents the relative number of dispersal events per million years. 1 means more frequent and 0 means less frequent. Wing length of 6.69 mm is the turning point of the state (from 0 to 1). The grey area indicates the 95% confidence interval. *R* represents the Spearman correlation coefficient. *p* means significance. **(B) Probability density distribution of mean wing length by relative dispersal events per million years.** The data for the two plots are derived from the fitted hidden Markov model.

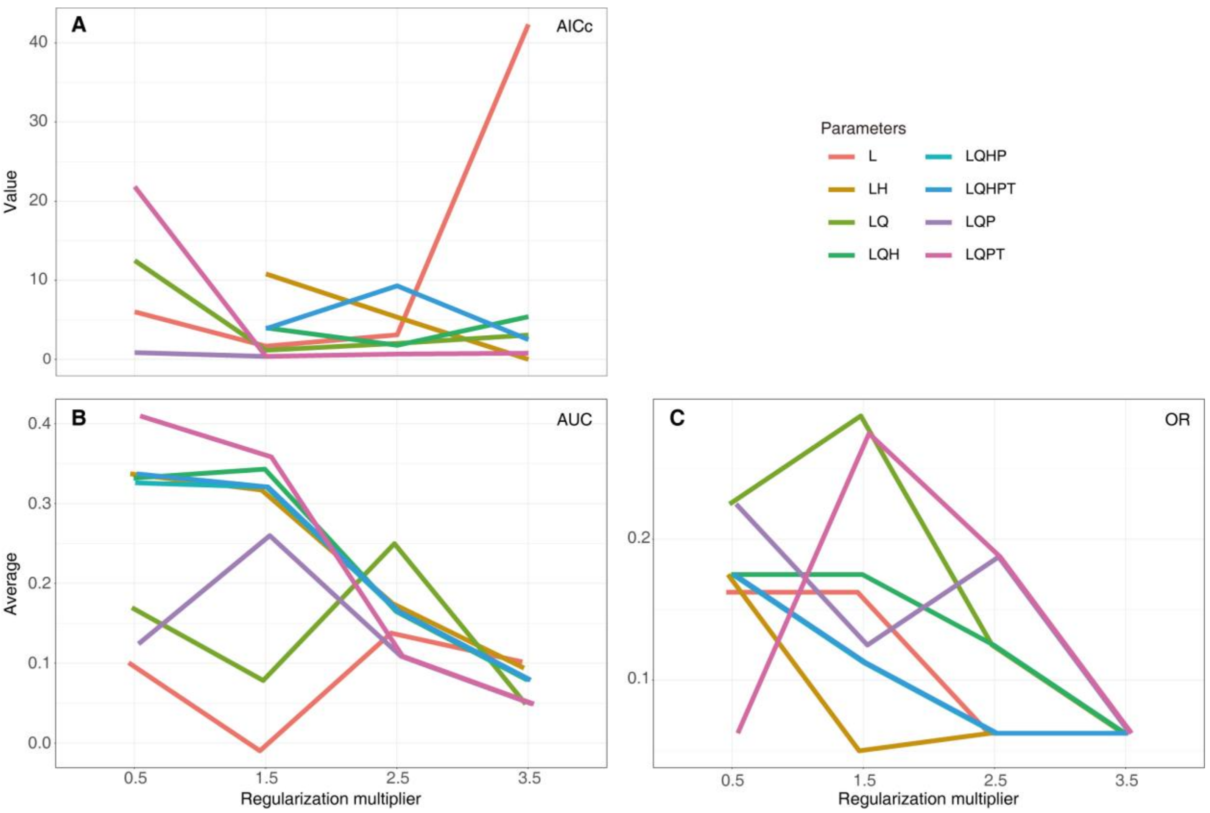

Fig. S17 Evaluation metrics of MaxEnt models for fossil berothids in the climatic conditions of 100 Ma across feature class combinations and regularization multipliers. (A) Akaike information criterion corrected (AICc) curve. Horizontal axis means different regularization multipliers, and vertical axis indicates the AICc value. **(B)** Area Under the Curve (AUC). Horizontal axis is the same as A, and vertical axis indicates average AUC value. **(C)** Omission Rate (OR) curve. Vertical axis means omission value. Colors indicate different sets of parameters. Abbreviation: L – linear feature; H – hinge feature; Q – quadratic feature; P – product feature; T – threshold feature.

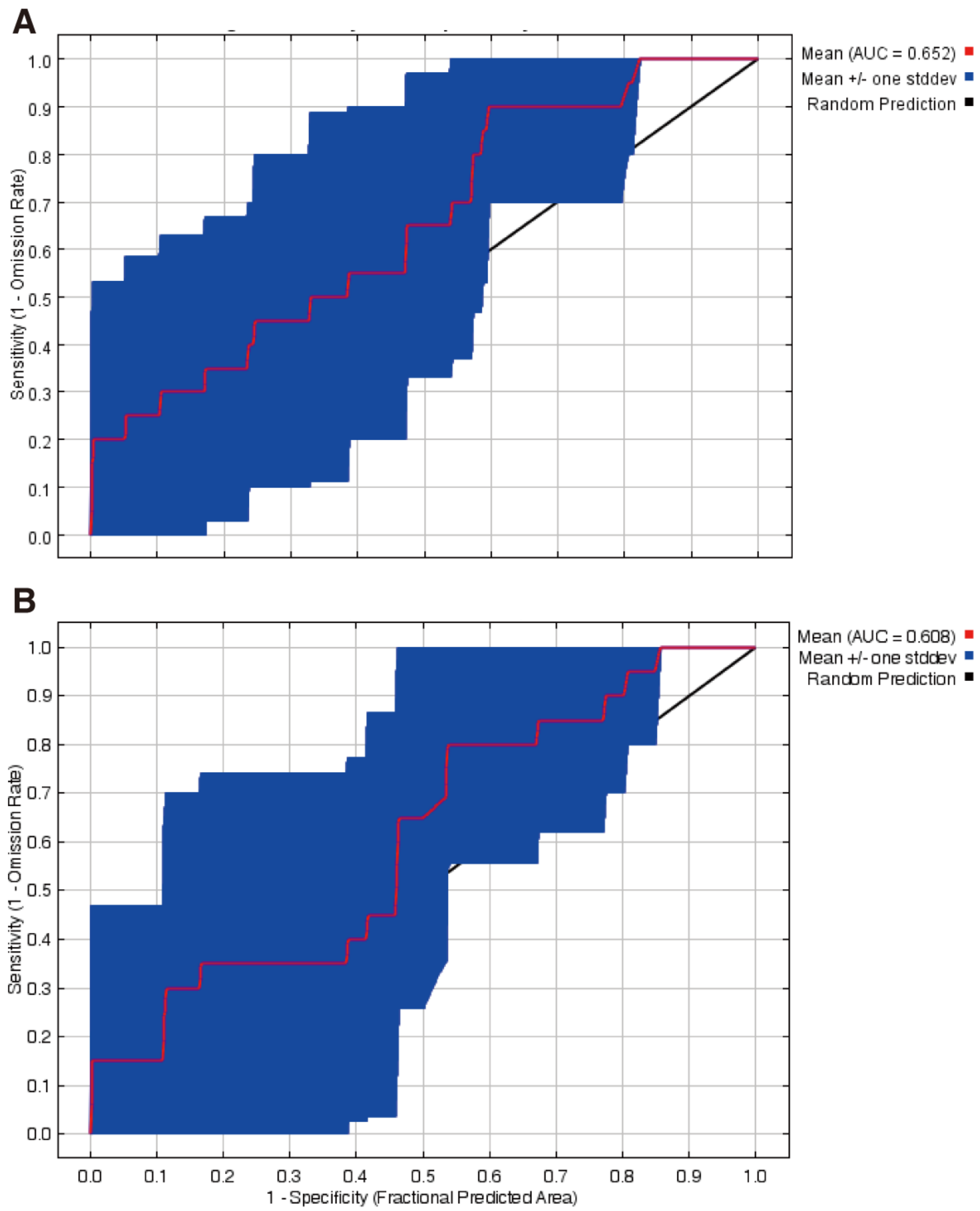

Fig. S18 Receiver operating characteristic (ROC) curve for modeling of fossil data with environment variables at 100 Ma. (A) ROC curve of all fossils data in MaxEnt. **(B)** ROC curve of Mesozoic-only fossils data in this model.

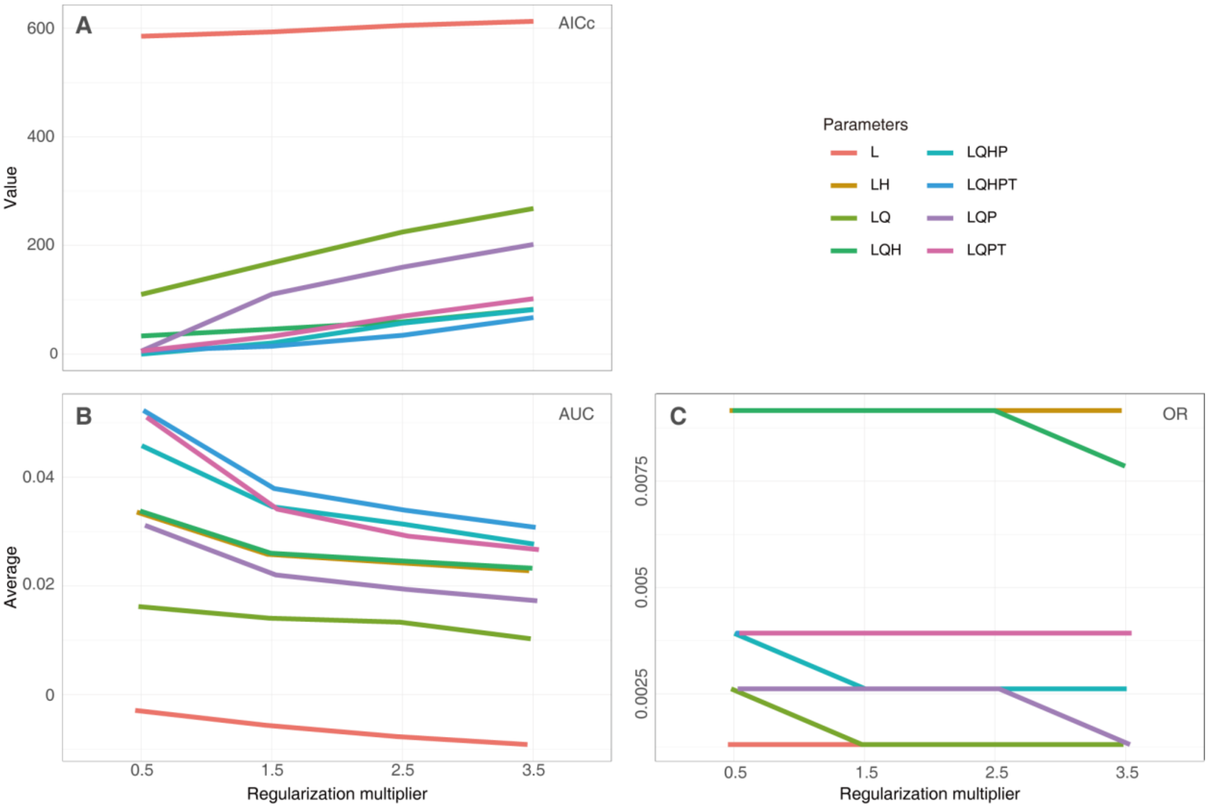

Fig. S19 Evaluation metrics of MaxEnt models for extant berothids in the present climatic conditions across feature class combinations and regularization multipliers. (A) Akaike information criterion corrected (AICc) curve. Horizontal axis means different regularization multipliers, and vertical axis indicates the AICc value. **(B)** Area Under the Curve (AUC). Horizontal axis is the same as A, and vertical axis indicates average AUC value. **(C)** Omission Rate (OR) curve. Vertical axis means omission value. Colors indicate different sets of parameters. Abbreviation: L – linear feature; H – hinge feature; Q – quadratic feature; P – product feature; T – threshold feature.

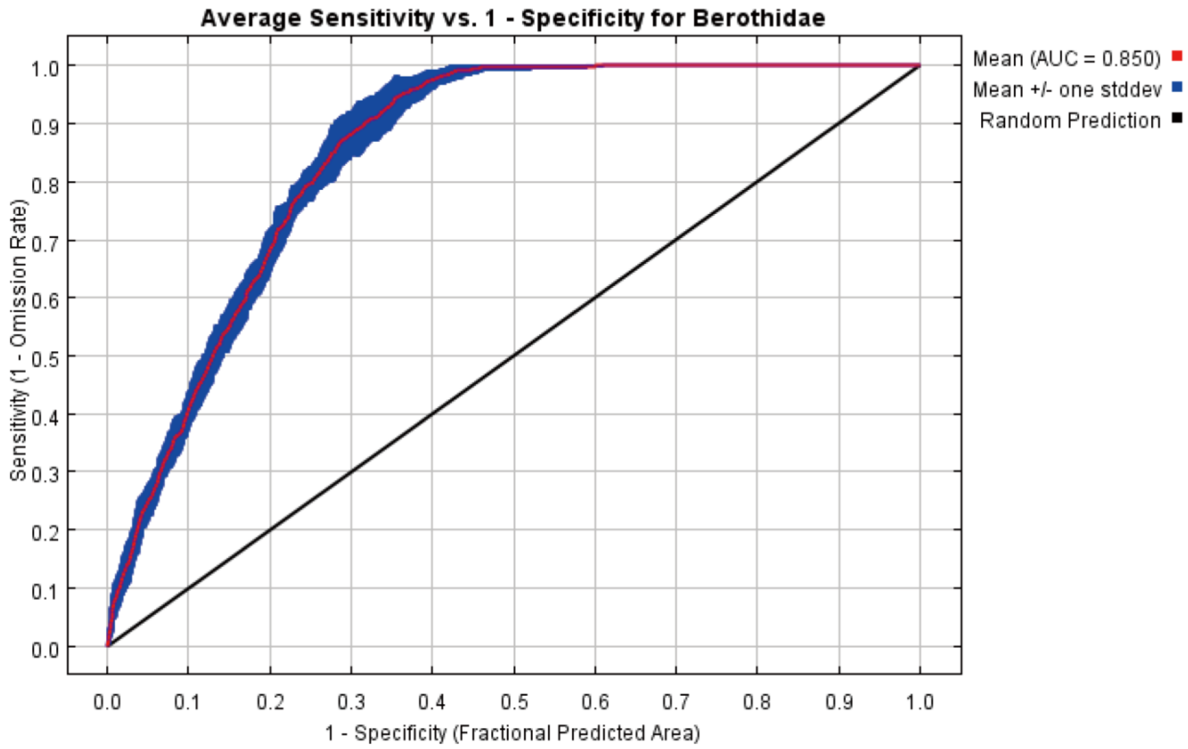

Fig. S20 Receiver operating characteristic (ROC) curve for modeling of extant species.

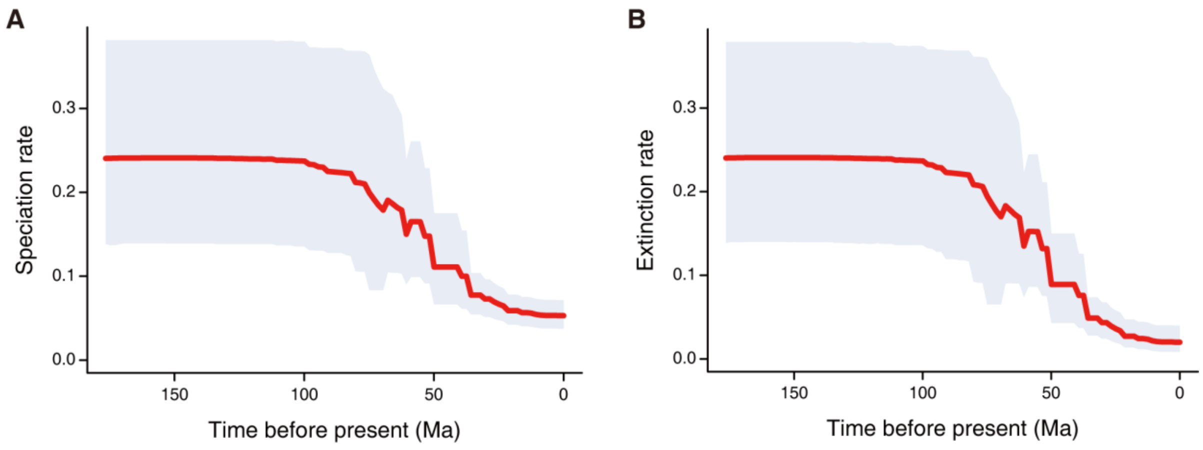

Fig. S21 Speciation (A) and extinction (B) rate of Berothidae through time. The grey area indicates the 95% confidence interval. The horizontal axis indicates geological time (Ma). The vertical axis indicates speciation rate or extinction rate.

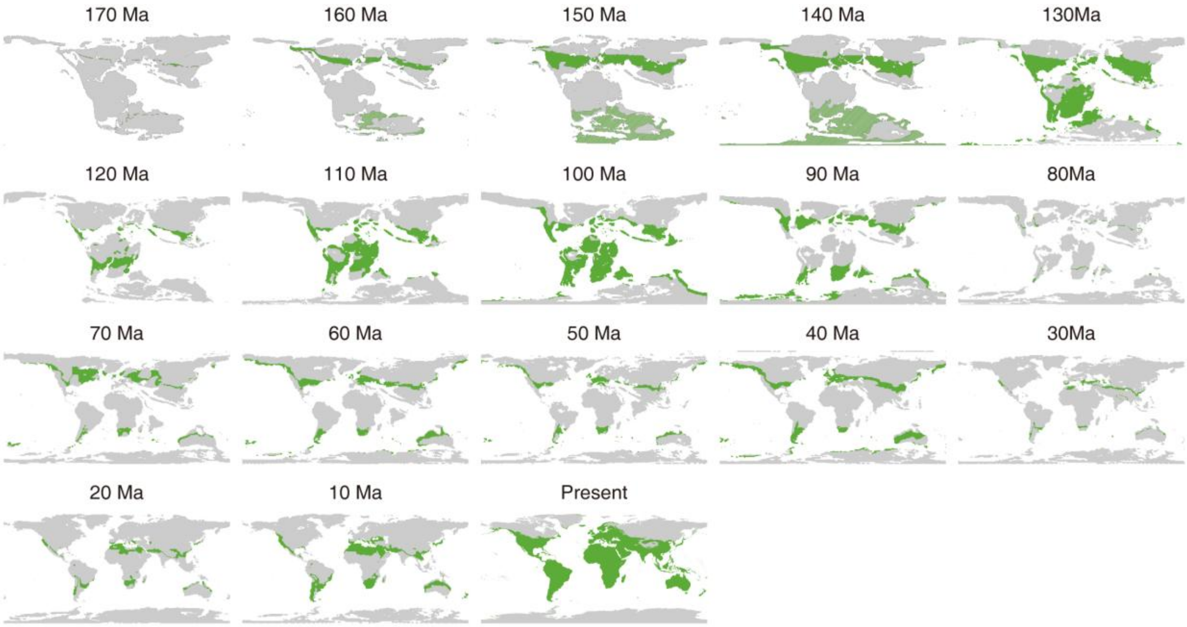

Fig. S22 Projection of potential habitable area (in green) for Berothidae based solely on *H*-CMT threshold through time. The masked green area indicates area with suitable *H*-CMT but not reachable at the time bin.

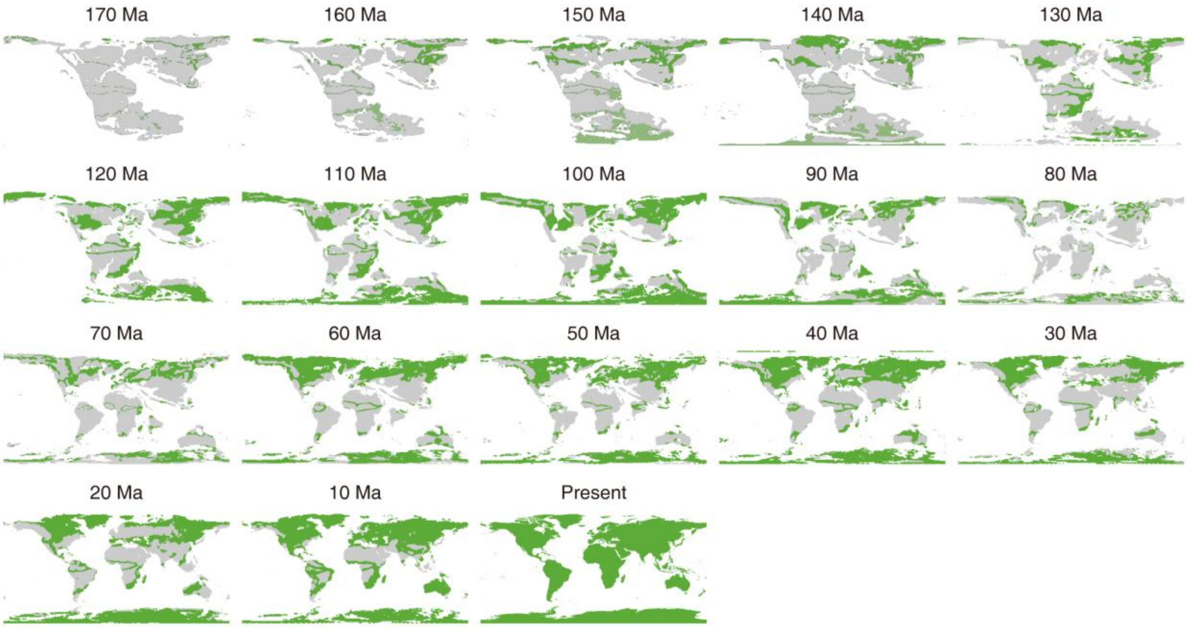

Fig. S23 Projection of potential habitable area (in green) for Berothidae based solely on *H*-DMP threshold through time. The masked green area indicates area with suitable *H*-DMP but not reachable at the time bin.

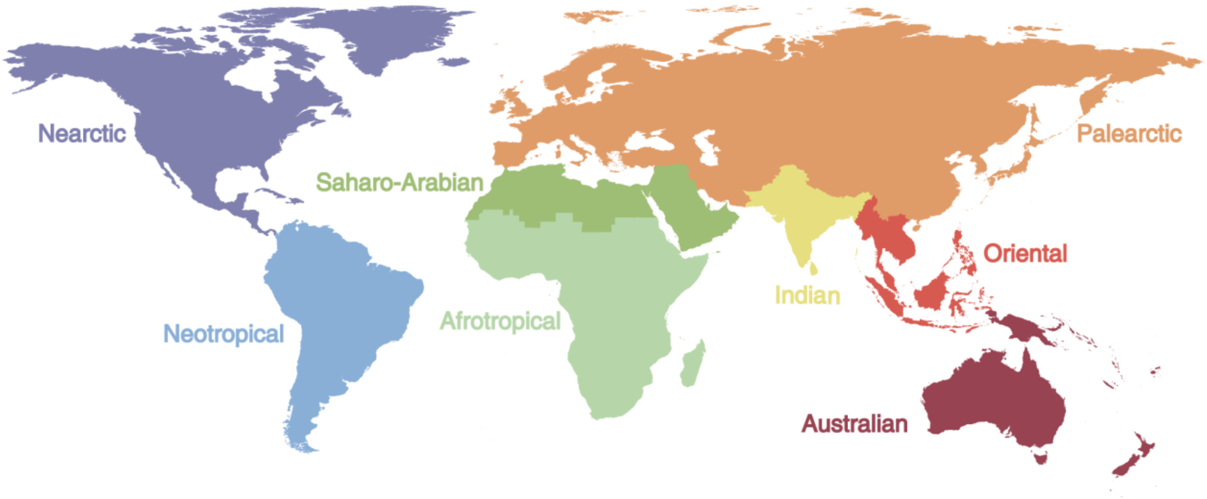

Fig. S24 Biogeographic realms division scenario applied in historical biogeographic study.

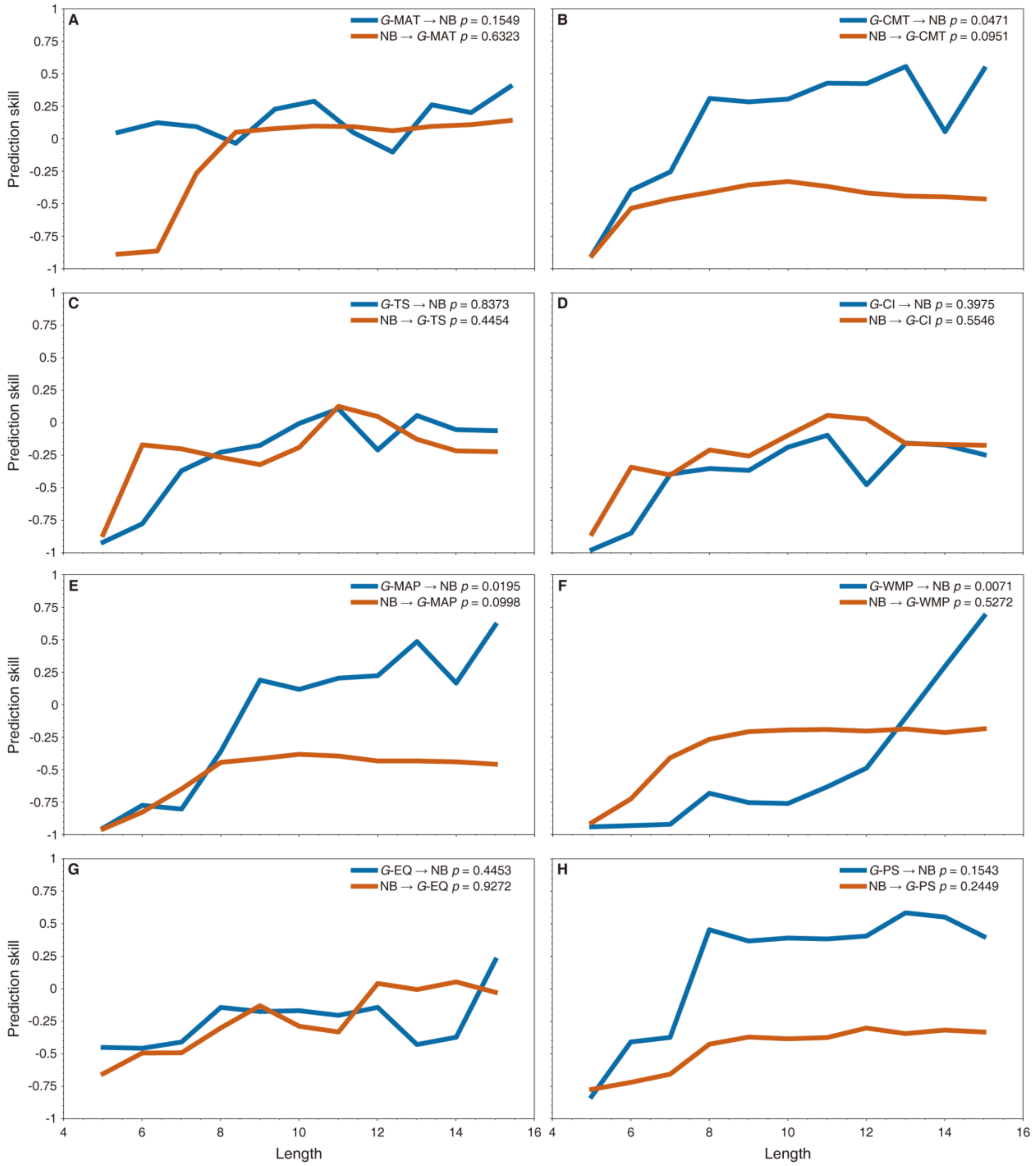

Fig. S25 Causal inference between global environmental variables and niche breadth (NB) of Berothidae. **(A)** Causal inference between *G*-MAT and NB, **(B)** between *G*-CMT and NB, **(C)** between *G*-TS and NB, **(D)** between *G*-CI and NB, **(E)** between *G*-MAP and NB, **(F)** between *G*-WMP and NB, **(G)** between *G*-EQ and NB and **(H)** between *G*-PS and NB. Blue line means the direction from global environmental variables to NB, while orange line indicates the opposite direction. *p* means significance.

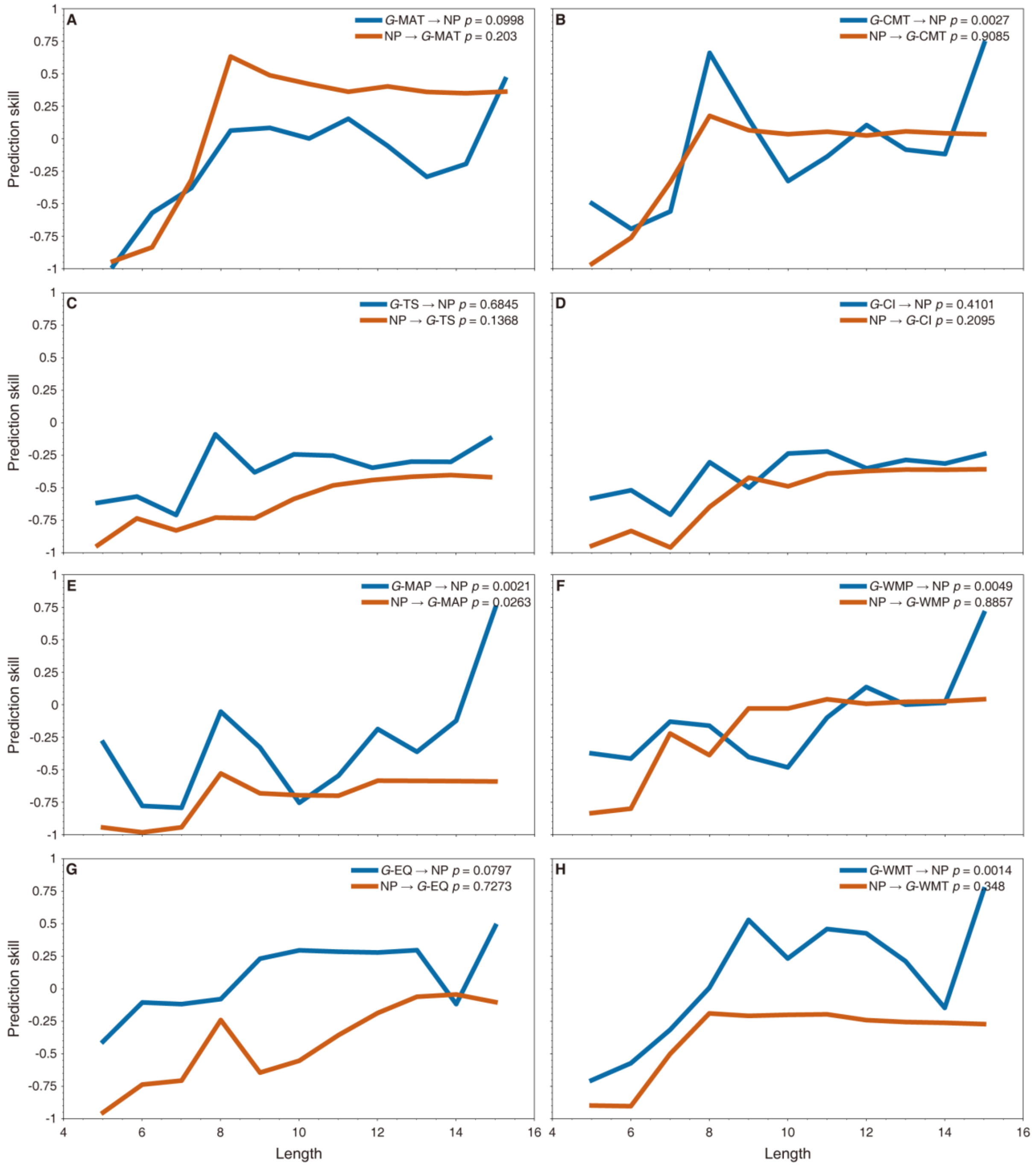

Fig. S26 Causal inference between global environmental variables and niche position (NP) of Berothidae. (A) Causal inference between *G*-MAT and NP, **(B)** between *G*-CMT and NP, **(C)** between *G*-TS and NP, **(D)** between *G*-CI and NP, **(E)** between *G*-MAP and NP, **(F)** between *G*-WMP and NP, **(G)** between *G*-EQ and NP and **(H)** between *G*-WMT and NP. Blue line means the direction from global environmental variables to NP, while orange line indicates the opposite direction. *p* means significance.

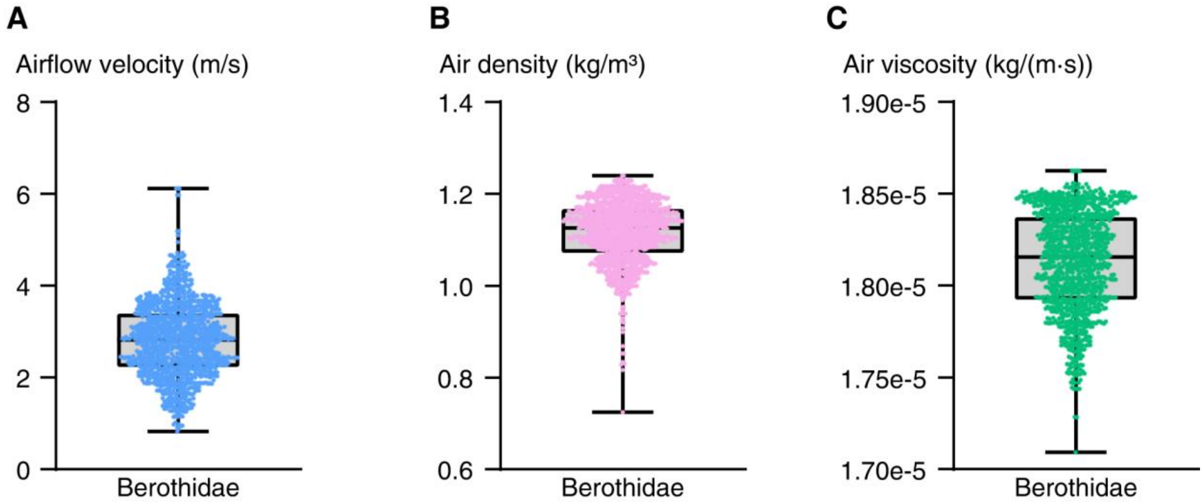

Fig. S27 Raw data for each of habitat atmospheric variables. The vertical axis represents the magnitude of the value of the habitat atmospheric variables. **(A)** airflow velocity, **(B)** air density, **(C)** air viscosity.

Table S1. Environmental variables explanation and abbreviation.

Bioclimatic variables	Explanation	Unit	Global background variables	Berothidae Habitat variables
Bio1	Annual mean temperature	°C	***G*-MAT**	***H*-MAT**
Bio4	Temperature seasonality	°C	***G*-TS**	***H*-TS**
Bio12	Annual precipitation	mm	***G*-MAP**	***H*-MAP**
Bio13	Precipitation of wettest month	mm	***G*-WMP**	***H*-WMP**
Bio14	Precipitation of driest month	mm	***G*-DMP**	***H*-DMP**
Bio15	Precipitation seasonality	mm	***G*-PS**	***H*-PS**
Bio20	Ellenberg quotient	°C/mm	***G*-EQ**	***H*-EQ**
Bio27	Simplified continentality index	°C	***G*-CI**	***H*-CI**
Bio28	Mean temperature of warmest month	°C	***G*-WMT**	***H*-WMT**
Bio29	Mean temperature of coldest month	°C	***G*-CMT**	***H*-CMT**
Elevation	Elevation	m	***G*-EL**	***H*-EL**

Table S2. Phylogenetic signals and trait evolution models.

Factor	*K*	*p* value	*λ*	*p* value	Fitted model
***H*-MAT**	0.5610439	0.0020	1.006783	0.000100	EB
***H*-EMT**	0.4307829	0.0054	0.980043	0.000100	OU
***H*-CMT**	0.8261142	0.0002	1.015315	0.000100	EB
***H*-TS**	1.3783733	0.0001	1.017086	0.000100	EB
***H*-CI**	1.3567661	0.0001	1.016860	0.000100	EB
***H*-MAP**	0.4145048	0.0097	0.833028	0.000100	OU
***H*-WMP**	0.5104507	0.0013	0.905551	0.000100	DT
***H*-DMP**	0.2164876	0.2430	0.000067	1.000000	OU
***H*-PS**	0.3728160	0.0136	0.832760	0.005372	OU
***H*-EQ**	0.1346374	0.7964	0.011443	0.862101	DT
***H*-EL**	0.1359096	0.8539	0.000067	1.000000	WN
**Niche Position**	0.8555934	0.0001	1.017166	0.000100	EB
**Wing Length**	0.4086708	0.0151	0.947774	0.000100	OU
**Wing Shape**	0.4350039	0.0077	0.417549	0.000857	DT

WN = white-noise (non-phylogenetic) model; DT = delta model; LB = lambda model; KP = kappa model; EB = Early-burst model; OU = Ornstein-Uhlenbeck model.

Table S3. Fluid domain conditions in transient analyses.

Case	Airflow velocity (m/s)	Air density (kg/m^3)	Air viscosity (kg/(m*s))
v_{max}<br>v_{min}	**6.117**<br>**0.820**	1.117	1.813e-5
ρ_{max}<br>ρ_{min}	2.815	**1.239**<br>**0.724**	1.813e-5
μ_{max}<br>μ_{min}	2.815	1.117	**1.863e-5**<br>**1.709e-5**

Table S4. Maximum lift-to-drag ratio of different wing length/shape under varying atmospheric conditions.

Case	Maximum lift-to-drag ratio (L/D_{max})		
	Falcate wing	Long oval-shaped wing	Short oval-shaped wing
v_{max}	0.1995	0.2143	0.0578
v_{min}	0.5185	0.5604	0.1754
ρ_{max}	0.3158	0.3401	0.1003
ρ_{min}	0.2259	0.2440	0.0724
μ_{max}	0.2913	0.3138	0.0924
μ_{min}	0.3072	0.3309	0.0975

Table S5. Aerodynamic index values in the steady analyses of two wing shapes.

Angle-of-attack (α)	Falcate wing		Oval-shaped wing	
	L/D	C_d (*10^{-5})	*L/D*	C_d (*10^{-5})
2°	0.493	2.035	0.515	2.134
4°	0.966	2.086	1.002	2.191
6°	1.380	2.174	1.430	2.288
8°	1.723	2.298	1.783	2.424
10°	1.977	2.458	2.043	2.602
12°	2.133	2.653	2.204	2.818
16°	2.145	3.120	2.212	3.330
20°	1.918	3.626	1.948	3.856
24°	1.680	4.206	1.687	4.461
30°	1.381	5.199	1.385	5.536
36°	1.146	6.280	1.148	6.698

Table S6. Aerodynamic index values in the steady analyses of two wing shapes in the temperature field.

Tempe-rature	Wing Shape	Value	Angle-of-attack (α)										
			**2°**	**4°**	**6°**	**8°**	**10°**	**12°**	**16°**	**20°**	**24°**	**30°**	**36°**
**296.43 K**	falcate	C_l	2.998e-5	6.023e-5	8.986e-5	1.187e-4	1.449e-4	1.636e-4	1.779e-4	1.849e-4	1.916e-4	2.004e-4	2.039e-4
		C_d	3.911e-5	4.057e-5	4.308e-5	4.671e-5	5.154e-5	5.736e-5	7.101e-5	8.724e-5	1.056e-4	1.361e-4	1.686e-4
		L/D	0.766	1.484	2.086	2.540	2.811	2.853	2.505	2.120	1.815	1.472	1.210
	oval	C_l	3.266e-5	6.540e-5	9.777e-5	1.295e-4	1.591e-4	1.811e-4	1.921e-4	1.969e-4	2.048e-4	2.149e-4	2.184e-4
		C_d	4.104e-5	4.265e-5	4.543e-5	4.943e-5	5.480e-5	6.127e-5	7.555e-5	9.264e-5	1.126e-4	1.457e-4	1.804e-4
		L/D	0.796	1.533	2.152	2.619	2.903	2.956	2.542	2.125	1.819	1.475	1.211
**Percentage difference**		C_d	4.80%	5.00%	5.32%	5.66%	6.14%	6.59%	6.18%	6.01%	6.40%	6.83%	6.74%
		L/D	3.76%	3.24%	3.12%	3.05%	3.19%	3.54%	1.48%	0.26%	0.22%	0.19%	0.12%
**292.59 K**	falcate	C_l	4.093e-5	8.217e-5	1.227e-4	1.621e-4	1.971e-4	2.176e-4	2.370e-4	2.465e-4	2.573e-4	2.697e-4	2.748e-4
		C_d	4.703e-5	4.900e-5	5.237e-5	5.734e-5	6.402e-5	7.192e-5	9.138e-5	1.137e-4	1.389e-4	1.804e-4	2.243e-4
		L/D	0.870	1.677	2.342	2.827	3.080	3.026	2.594	2.168	1.852	1.495	1.225
	oval	C_l	4.451e-5	8.910e-5	1.334e-4	1.769e-4	2.169e-4	2.414e-4	2.537e-4	2.631e-4	2.752e-4	2.896e-4	2.945e-4
		C_d	4.935e-5	5.153e-5	5.527e-5	6.075e-5	6.817e-5	7.690e-5	9.705e-5	1.210e-4	1.483e-4	1.933e-4	2.401e-4
		L/D	0.902	1.729	2.413	2.911	3.181	3.139	2.614	2.174	1.855	1.498	1.226
**Percentage difference**		C_d	4.82%	5.03%	5.39%	5.77%	6.28%	6.70%	6.02%	6.23%	6.53%	6.92%	6.80%
		L/D	3.57%	3.07%	2.98%	2.94%	3.25%	3.66%	0.77%	0.30%	0.21%	0.17%	0.11%

Table S7. Pearson correlation coefficient of habitat environmental variables.

	***H*-MAT**	***H*-TS**	***H*-MAP**	***H*-WMP**	***H*-DMP**	***H*-PS**	***H*-EQ**	***H*-CI**	***H*-WMT**	***H*-CMT**
***H*-MAT**	1	-0.676	0.190	0.331	-0.156	0.483	0.053	-0.672	0.763	**0.945**
***H*-TS**		1	-0.403	-0.486	0.078	-0.461	0.048	**0.998**	-0.054	**-0.874**
***H*-MAP**			1	**0.867**	0.562	-0.063	-0.118	-0.392	-0.071	0.298
***H*-WMP**				1	0.181	0.312	-0.103	-0.475	0.050	0.427
***H*-DMP**					1	-0.630	-0.057	0.084	-0.147	-0.144
***H*-PS**						1	-0.048	-0.464	0.254	0.518
***H*-EQ**							1	0.042	0.095	0.011
***H*-CI**								1	-0.047	**-0.871**
***H*-WMT**									1	0.531
***H*-CMT**										1

Table S8. Contribution of habitat environmental variables in the first three principal components. Variables in bold are used in ecological niche modeling.

Environmental variables	PC1	PC2	PC3
H-EL	4.971343	0.031304	25.94695
H-MAT	16.90807	4.041402	5.517525
H-TS	16.98221	0.408299	6.636536
H-MAP	5.284953	28.92921	0.045613
***H*-WMP**	8.698446	12.35173	1.445225
***H*-DMP**	0.011268	29.26321	11.2965
***H*-PS**	6.120063	11.97835	10.72284
***H*-EQ**	0.023369	1.583002	3.59636
H-CI	16.83844	0.348288	6.788062
***H*-WMT**	3.837313	9.933807	27.85283
***H*-CMT**	20.32453	1.131389	0.151554

Table S9. Niche breadth, niche position and marginality of berothids in five geochronological intervals.

Geochronological interval	Niche breadth	Niche position	Marginality
**Jurassic**	1.873	-5.21627	32.927
**Early Cretaceous**	18.757	-2.58545	8.583
**Late Cretaceous**	5.067	2.941136	14.15
**Paleogene**	0.115	-2.556	14.008
**Recent**	3.344	0.06942	0.007

Table S10. Niche breadth, niche position and marginality of berothids in 16-time bins.

Time bin	Niche breadth	Niche position	Marginality
**170 Ma**	3.473	5.26	34.748
**160 Ma**	0.038	4.012	23.641
**150 Ma**	4.747	5.005	30.963
**140 Ma**	0.043	-0.208	3.126
**130 Ma**	23.163	3.423	14.224
**120 Ma**	0.123	-2.958	12.107
**110 Ma**	1.141	-2.399	9.87
**100–90 Ma**	1.136	-3.731	18.885
**80–70 Ma**	1.172	1.638	4.804
**60 Ma**	1.75	0.59	4.329
**50 Ma**	3.458	1.341	6.575
**40 Ma**	4.172	1.146	3.957
**30 Ma**	0.064	-0.43	0.513
**20 Ma**	0.525	0.053	0.743
**10 Ma**	0.328	-0.323	0.267
**0 Ma**	1.998	-0.048	0.009

Table S11. Maximum, minimum and mean values of the habitat atmospheric variables.

Value	Airflow velocity (m/s)	Air density (kg/m^3)	Air viscosity (kg/(m*s))
Maximum	6.117	1.239	1.863e-5
Mean	2.815	1.117	1.813e-5
Minimum	0.820	0.724	1.709e-5

Datasets list

S1. Distribution and corresponding environment variables of extant species of Berothidae.

S2. Distribution and corresponding paleoenvironment variables of fossil species of Berothidae.

S3. Niche breadth and position through time and corresponding environment variables.

S4. Lineage number, net diversification rate, and global mean annual temperature (*G*-MAT) through time.

S5. Environmental indicators data.

S6. Morphological matrix.

S7. Combined matrix of morphological and molecular data.

S8. Molecular data information of extant species used in phylogenetic analyses.

S9. Posterior consensus tree including *Ferganoberotha* and *Triassoberotha* as a 50% majority-rule.

S10. Dispersal multipliers applied in historical biogeographic analysis.

SI References

1. F. P. Peixoto, F. Villalobos, M. V. Cianciaruso, Phylogenetic conservatism of climatic niche in bats. *Glob. Ecol. Biogeogr.* **26**, 1055–1065 (2017).
2. S. F. Gouveia *et al.*, Climatic niche at physiological and macroecological scales: The thermal tolerance-geographical range interface and niche dimensionality. *Glob. Ecol. Biogeogr.* **23**, 446–456 (2014).
3. J. Heino, M. Grönroos, Untangling the relationships among regional occupancy, species traits, and niche characteristics in stream invertebrates. *Ecol. Evol.* **4**, 1931–1942 (2014).
4. K. A. Carscadden *et al.*, Niche breadth: Causes and consequences for ecology, evolution, and conservation. *Q. Rev. Biol.* **95**, 179–214 (2020).
5. J. Hausser, *Säugetiere der Schweiz / Mammifères de la Suisse / Mammiferi della Svizzera* (Birkhäuser Basel, 1995).
6. M. P. Rocha *et al.*, Predicting occupancy and abundance by niche position, niche breadth and body size in stream organisms. *Oecologia* **186**, 205–216 (2018).
7. D. M. Vela Díaz *et al.*, Untangling the importance of niche breadth and niche position as drivers of tree species abundance and occupancy across biogeographic regions. *Glob. Ecol. Biogeogr.* **29**, 1542–1553 (2020).
8. J. Heino, Positive relationship between regional distribution and local abundance in stream insects: A consequence of niche breadth or niche position? *Ecography* **28**, 345–354 (2005).
9. J. D. Carballo-Morales, R. A. Saldaña-Vázquez, F. Villalobos, L. Herrera-Alsina, Thermal niche breadth and their relationship with sturnira bat species diversification. *J. Therm. Biol.* **117**, 103697 (2023).
10. A. V. Khramov, The first Triassic beaded lacewing (Neuroptera: Berothidae) from Central Asia, with redescription of *Mesoberotha superba* (Riek, 1955). *Zootaxa* **5330**, 287–294 (2023).
11. A. V. Khramov, N. Oyama, S. Kenji, H. Takahashi, Late Triassic lacewings (Insecta: Neuroptera) from Japan. *Hist. Biol.* 1–9 (2023).

12. D. Zhang *et al.*, PhyloSuite: An integrated and scalable desktop platform for streamlined molecular sequence data management and evolutionary phylogenetics studies. *Mol. Ecol. Resour.* **20**, 348–355 (2020).

13. V. Kellermann *et al.*, Phylogenetic constraints in key functional traits behind species' climate niches: Patterns of desiccation and cold resistance across 95 *Drosophila* species. *Evolution* **66**, 3377–3389 (2012).

14. X. Arnan *et al.*, Introduced ant species occupy empty climatic niches in Europe. *Sci. Rep.* **11**, 3280 (2021).

15. J.-P. Burg, M. Ford, Orogeny through time: An overview. *Geol. Soc. Lond. Spec. Publ.* **121**, 1–17 (1997).

16. P. Molnar, P. Tapponnier, A possible dependence of tectonic strength on the age of the crust in Asia. *Earth Planet. Sci. Lett.* **52**, 107–114 (1981).

17. D. B. Rowley, B. S. Currie, Palaeo-altimetry of the late Eocene to Miocene Lunpola basin, central Tibet. *Nature* **439**, 677–681 (2006).

18. L. Ding *et al.*, The Andean-type Gangdese Mountains: Paleoelevation record from the Paleocene–Eocene Linzhou Basin. *Earth Planet. Sci. Lett.* **392**, 250–264 (2014).

19. F. Cai, L. Ding, Y. Yue, Provenance analysis of upper Cretaceous strata in the Tethys Himalaya, southern Tibet: Implications for timing of India-Asia collision. *Earth Planet. Sci. Lett.* **305**, 195–206 (2011).

20. D. J. J. van Hinsbergen *et al.*, Greater India Basin hypothesis and a two-stage Cenozoic collision between India and Asia. *Proc. Natl Acad. Sci.* **109**, 7659–7664 (2012).

21. P. Kapp, P. G. DeCelles, Mesozoic–Cenozoic geological evolution of the Himalayan-Tibetan orogen and working tectonic hypotheses. *Am. J. Sci.* **319**, 159–254 (2019).

22. T. N. Sullivan, M. A. Meyers, E. Arzt, Scaling of bird wings and feathers for efficient flight. *Sci. Adv.* **5**, eaat4269 (2019).
