# Supplementary material for: Dynamic interplay between niche variation and flight adaptability drove a hundred million years’ dispersion in iconic lacewings
Source: Proc Natl Acad Sci U S A. 2025 May 2;122(19):e2414549122. doi: 10.1073/pnas.2414549122 (PMC12087969; doi:10.1073/pnas.2414549122)
Supplement: Supplementary file 7 — Dataset S06 (RTF) [file pnas.2414549122.sd06.rtf]

#NEXUS[written Sun Oct 15 15:08:15 CST 2023 by Mesquite  version 3.70 (build 940) at scfs-iMac.local/127.0.0.1]BEGIN TAXA;	TITLE Taxa;	DIMENSIONS NTAX=53;	TAXLABELS		Mucroberotha Nallachius Plega Asadeteva Austroberothella Berlekrumyia Berotha Berothimerobius Cyrenoberotha Isoscelipteron Lekrugeria Lomamyia Manselliberotha Naizema Nodalla Nosybus Nyrma Ormiscocerus Podallea Protobiella Quasispermophorella Speleoberotha Spermophorella Spiroberotha Stenobiella Tanzanberotha Trichoberotha Trichoma Sinosmylites Berothone Krokhathone Mesithone Epimesoberotha Pseudosisyra Oloberotha Sibelliberotha Araripeberotha Caririberotha Cantabroberotha Aggregataberotha Ansoberotha Cornoberotha Dasyberotha Dolichoberotha Haploberotha Protoberotha Xiaoberotha Jersiberotha Nascimberotha Plesiorobius Microberotha Xenoberotha Elektroberotha 	;END;BEGIN CHARACTERS;	TITLE  Character_Matrix;	DIMENSIONS  NCHAR=103;	FORMAT DATATYPE = STANDARD RESPECTCASE GAP = - MISSING = ? SYMBOLS = "  0 1 2 3 4";	CHARSTATELABELS 		1 head_vertex /  domed flat,		2 postocular_margin_width /  '<1/5 of eye width' '>1/5 of eye width',		3 antennae /  pectinate moniliform,		4 antennal_scape_length_comparing_to_1st_flagellomere_length /  not_longer_than_4_times longer_than_4_times,		5 'frons + clypeus length' /  wider_than_long longer_than_wid,		6 scale_on_protonum_ /  absent present,		7 pronotum_suture /  absent present,		8 pronotum_with_fore_coxa /  elongate_anterior_to_forecoxa elongate_and_extended_ventrally_posterior_to_forecoxa,		9 forelegs /  cursorial raptorial,		10 forefemur_length_comparing_to_foretibia_and_tarsus_together /  'shorter than 4/5 length' 'longer than 4/5 length',		11 forefemur_width_comparing_to_length /  '<1/5' '>1/5',		12 forefemur_width_comparing_to_forecoxa /  thinner_than_1.2_times_of_coxa_width wider_than_1.2_times_of_coxa_width,		13 forefemur /  with_thin_setae with_spine,		14 foretibia /  without_a_lateroventral_ridge with_a_lateroventral_ridge,		15 foretibia_ventral_edge /  thin_setae with_short_prostrate_setae,		16 foretibia_spurs /  absent present,		17 foretarsus /  'five-segmented' 'four-segmented',		18 'foretarsomere 1 length comparing to tarsomeres 2-4 combined' /  less_than_1.2_times longer_than_1.2_times,		19 foretarsomere_1_apex /  thin_setae spine,		20 foretarsomere_2_length_to_tarsomere_3 /  shorter_than_2_times longer_than_2_times,		21 foretarsi_ventral_face /  short_thin_setae 'spine-like setae',		22 mid_and_hind_leg_spurs /  present absent,		23 mid_and_hind_tarsomere_1_length_to_tarsomere_2 /  '<2 times' '>2 times',		24 mid_and_hind_tarsomere_2_length_to_width /  more_than_2_times_longer_than_wide less_than_2_times_longer_than_wide,		25 mid_and_hind_tarsi_ventral_bristles /  absent one_pair_on_each_tarsomere two_or_more_pairs_on_each_tarsomere,		26 scale_on_wings /  absent present,		27 trichosors_number /  1 more_than_1,		28 forewing_length_to_width /  '<2.8' '>2.8',		29 forewing_humeral_vein /  not_recurrent recurrent,		30 forewing_costal_space_width /  '< 1/6 of wing width' '1/6 - 1/5 of wing width' '> 1/5 of wing width',		31 forewing_costal_crossveins /  most_simple most_forked,		32 forewing_subcostal_space_width_comparing_to_radial_space_width /  '< 7/10' '> 7/10',		33 'forewing sc-r number' /  one_or_none more_than_one,		34 forewing_Sc_termination /  R C,		35 forewing_pterostigma /  conspicuous faint,		36 forewing_pterostigma_position_with_Sc_termination /  after_pterostigma within_or_before_pterostigma,		37 forewing_pterostigma_connection_with_R1 /  touching separated,		38 forewing_crossveins_in_stigmal_area /  complete_and_distinct incomplete_or_faint,		39 'forewing r1-rs number' /  three_or_more two one_or_none,		40 forewing_Rs_branches_number /  less_than_7 more_than_7,		41 forewing_MP_and_R_base /  separated fused,		42 'forewing basal ma-mp position' /  distal_to_Rs_separation proximal_to_Rs_separation,		43 forewing_gradate_series /  reticulate_crossveins one_series_or_few_crossveins two_or_more_series,		44 forewing_iMP_cell_length /  not_longer_than_MP_stem_length longer_than_MP_stem_length,		45 'forewing mp-cua number' /  three_or_more one_to_two,		46 forewing_CuA_fork_position /  'distal 3/5 of wing length' 'basal 2/5 of wing length',		47 forewing_CuA_branched /  pectinate dichotomously_or_simple,		48 forewing_CuP_branched /  pectinate dichotomous_or_simple,		49 forewing_CuP_basal /  not_close_to_1A touching_or_very_closely_approximating_1A,		50 'forewing cua-cup' /  present absent,		51 'forewing cup-1a number' /  present absent,		52 forewing_2A_and_3A_basally /  free fused,		53 forewing_2A_and_3A_termination /  free fused_to_form_a_loop,		54 forewing_jugal_lobe /  reduced well_developed,		55 vesicae /  absent present,		56 nygma /  present absent,		57 hind_wing_humeral_plate /  beset_with_stout_setae with_frenulum,		58 hind_wing_costal_space_width_comparing_to_wing_width /  'narrower than 1/13 of wing width' 'wider than 1/13 of wing width',		59 hind_wing_Sc_in_the_midway /  distinctly_separated_from_C very_close_to_C_not_fused fused_with_C_and_separated_afterwards,		60 hind_wing_Sc_termination /  R1 C,		61 'hind wing sc-r1 number' /  absent present,		62 'hind wing r1-rs number' /  '<=1' 2 '>3',		63 hind_wing_Rs_branches /  no_more_than_six more_than_six,		64 'hind wing basal ma-mp' /  sinuous_and_longitudinal_oriented straight_and_oblique_or_vertical absent,		65 hind_wing_width_between_CuA_and_posterior_margin /  'narrower than 1/5 of wing width' 'wider than 1/5 of wing width',		66 hind_wing_CuA_termination /  distal_half_of_wing basal_half_of_wing,		67 hind_wing_CuA_branches_number /  '>4' '<3',		68 hind_wing_CuA_branches /  forked simple,		69 hind_wing_CuP_separated_from_CuA /  basally distally absent,		70 hind_wing_CuP /  simply_forked creeping fused_with_CuA absent,		71 hind_wing_CuP_and_1A /  'by cup-1a' touching not_touching_or_connected,		72 'hind wing cua-cup' /  absent present,		73 'hind wing cua-1a' /  absent present,		74 hind_wing_2A /  bifid simple with_multi_marginal_twigs,		75 male_9th_tergite_and_ectoproct /  separated fused,		76 male_callus_cerci /  present absent,		77 torulus /  absent present,		78 male_9th_sternite /  not_longer_than_ectoproct extending_posterior_to_ectoproct,		79 male_gonarcus_sclerization /  well_sclerized formed_or_covered_by_setose_membrane,		80 male_gornarcus_and_9th_gonocoxites_basally /  articulated fused separated,		81 male_9th_gonocoxite_length_comparing_to_9S /  distinctly_longer_than_9S not_longer_than_9S,		82 'male paramere and mediuncus/pseudopenis position' /  associated_or_separated partially_fused,		83 male_paramere /  paired one_piece undetected,		84 male_mediuncus_and_pseudopenis /  articulated_or_separated fused,		85 male_pseudopenis /  short_and_straight long_and_curved extremely_long_and_coiled,		86 male_pseudopenis /  without_bristles with_bristle,		87 female_7th_sternite /  one_piece paired,		88 female_7th_gonapophyses /  absent present,		89 female_8th_sternite /  absent one_piece one_piece_with_median_process one_piece_with_paired_processes,		90 female_8th_gonocoxite /  absent single_piece paired,		91 female_9th_tergite_and_ectoproct /  separated fused,		92 female_callus_cerci /  present absent,		93 female_9th_tergite_ventrally /  not_detached detached,		94 female_9th_tergite_pseudohypocauda /  absent present,		95 female_9th_tergite_and_9th_gonocoxite /  separated fused,		96 female_9th_gonocoxite_hypocauda /  absent short long,		97 female_9th_gonocoxite_position /  posterior_to_9th_tergite ventral_to_9th_tergite,		98 female_9th_gonocoxite /  forming_an_ovipositor no_modification,		99 female_9th_gonapophysis /  absent present,		100 female_spermatheca /  simple_coiled with_bulb with_difitiform_lobe,		101 female_bursa_copulatrix /  without_anterior_extension with_a_distinct_anterior_extension,		102 female_fertilization_canal /  within_bursa_copulatrix between_spermatheca_and_common_oviduct,		103 female_spermatheca /  paired unpaired ; 	MATRIX	Mucroberotha         0010100011011101(0 1)0(0 1)000002001000011001000101001011000111100011200000002100000001111002000001011011102111	Nallachius           01(0 1)0000000000000010001100000000011001010011000100100100001010101001000200000000001010000000000000001000	Plega                0010001111111110101100112010110100010111101001011010010110200000101000010100000000002000011000000002011	Asadeteva            0110000000??000?0?000????1010111000100011111110100000001?1100111000010101211000201111110211100020101011	Austroberothella     001000000000000000001110100100010001011000101011000000010000100100011100001000011-211100210011001112011	Berlekrumyia         0110000000??000?0?000????0001111111--1001111010100011001?10012110000100112110002011111102110001111?1011	Berotha              011100000000000001001110100100100001010001110101000100011010010100011120121000020-111111311000021101011	Berothimerobius      011000000000000000000????0001101111--0001100010100000001?110120100010101110000??11????001110011011?0011	Cyrenoberotha        0110100000000000000011001000101000010110001000110000010100000101000023-0100001001-211000020010001110011	Isoscelipteron       0110?00000??000?0?000??0?1000111000110011111010100010001?0100111000010101110101201112011321000020101011	Lekrugeria           0111000000??000?0?000????0011210111--0001111010100010001?1101211000010011211000201111110311000021100011	Lomamyia             111000000000000001001110110000110001001000110011000000011000010100001120121000120-211111311000021101011	Manselliberotha      0010100000000000000001001000101000010110001010110000010100000101000023-0100001000-211000010010001110011	Naizema              0110000000000000000011102000101100011120101010110010000101001001000010000210000100012100111000011111011	Nodalla              01110000000?0000000001???10100100000010011110101001?0001?0100101000010111110000201111110211000020101011	Nosybus              011?0000000000000100?1???00001101100110011111011000?0?010101000100001120121100021??121002110000001?1011	Nyrma                011010000???000?00001????0000101111--00000000101000?0?010101110100010001011000001-2110001010000011?1011	Ormiscocerus         0110?000000000000000??????000211111--000?000010100000?01?101110100010020020000001-2???002??0000011?0011	Podallea             1111010000000000010011101101001000010100011101010001010100100101000011201210001201112010211000011100011	Protobiella          ?110?0000???000?0000??10?0000110000100000011011100000001?0000101000011000110000?1??111002110110011?0011	Quasispermophorella  0110000000??000?0?000????0010210001--0101011010100110001?1100101000010001211101201112011311000020101011	Speleoberotha        01101000000000000100010000001011110011200011111101000001?1011000000112201101000011011000000100001110011	Spermophorella       1110000000000000010010101100001000010100011001010010000100100101000011201210000201112011311000021101011	Spiroberotha         0111010000000000000011101101001000010120101000110000000100100100000011000210000110012011?11011021111011	Stenobiella          0111010000000000000010101001011101011020011110110011100101110001000111211210001200011100211000011101011	Tanzanberotha        0110000000??000?0000??????000211111--00010110111000?0001?10110010001?????111000211?111??????????????011	Trichoberotha        ??1??0000???00??0?0??????00110100100?0001-100100000?0?01?101010100001000021010?10??11100111?000001?0011	Trichoma             011000000000000000001110100100101100110011100111000000010101000100001000021010110-211100321?000111?1011	Sinosmylites         ?????0000???0000?0?00?01?0000101001--10110111101001?0001?10012110000???1???????????????????????????????	Berothone            ?????????????????????????0001211101--010?0200011000?0?01???????????????????????????????????????????????	Krokhathone          ??????????0??????????????000000100???020?01111110???0?01???????????????????????????????????????????????	Mesithone            ???????????????????????????0111110???011(0 1)0110101000?0001???????????????????????????????????????????????	Epimesoberotha       ?????????????????????????0000101?0?--01(0 1)?010?101??????01???????????????????????????????????????????????	Pseudosisyra         ??????????????????????????001111111--0201011101101000001???????????????????????????????????????????????	Oloberotha           ?????0??0????????????????0?0121?00???00110110101000?0001?00001010000102000?????????????????????????????	Sibelliberotha       0?1010000?0?00000?0?010100000001000101201011100001000001?0100001000110200?????????????002????0?0???????	Araripeberotha       ?0????????????????????????0???0100???0101?1???????????0??000?10?0001???????????????????????????????????	Caririberotha        00?0??????????????????????0???11?0???010??1???????????0???00?00?0??????????????????????????????????????	Cantabroberotha      001000000?0?0000?????????0000101001--01110111011?0???001?00000000001???????????????????????????1???????	Aggregataberotha     001010000000000000000110?000000100001020101110(0 1)1011??001?10110020001?1????????????????001?01100011?????	Ansoberotha          0011?000000000000100001??0010000001--00010111100001??001?1000(1 2)01010101210?????????????1???10?0?201?????	Cornoberotha         00110000000?00000000010100000000001--00000111101000??001?1000202010(0 1)01210(1 3)0??0??0?????10??10?10201?????	Dasyberotha          0?1??000000?00?0?????????0010001001--010?0110111001??001?100??0???????????????????????????1??1??01?????	Dolichoberotha       001100000000000000000101?001011000010000?011110(0 1)000??001?1000(1 2)0100000(1 2)200101?0????????????10?00201?????	Haploberotha         011000000000000001000110?0000001001--1200011111101000001?0000002000100210111?0?????????????????????????	Protoberotha         001010000000000000000100?0000001001--0201011100101100?01?0011002000123-002????????????????0??0?011?????	Xiaoberotha          001000000000000000000111?0001001001--0100011001000000001?0000101000001010200?1??1?????????0?100001?????	Jersiberotha         0010?0000000000000000110?0010001001--010101110110(0 1)0?0001?0000002000123--0?????????????00?????1???1?????	Nascimberotha        0????0000?0?0??0?0?0?????0001100001--00000201001000??001?100020?1010?1?????????????????????????????????	Plesiorobius         1?1010??0000000000000?00?0001201001--120?0210101001???01?100??0???????????00?1?????????????????????????	Microberotha         011000000000000000000110?00100001101?02010111100010??001???11001000123--1011?0??????10?????????????????	Xenoberotha          0??0?0?00???0????????????001?0?0?0???0100011110?0?????01???????????????????????????????????????????????	Elektroberotha       ?0?0?100000000000100011?10001111001--00100110111000?00011000?201?000010102????????????102?1??10211?????;END;BEGIN ASSUMPTIONS;	TYPESET * UNTITLED   =  unord:  1- 103;END;BEGIN MESQUITECHARMODELS;	ProbModelSet * UNTITLED   =  'Mk1 (est.)':  1- 103;END;BEGIN NOTES;	SUT   TAXON = 26 NAME = color INTEGER = 1;	SU  T = 30 C = 1 N = color I = 15;	SU  T = 31 C = 1 N = color I = 13;	SU  T = 33 C = 1 N = color I = 13;	SU  T = 35 C = 1 N = color I = 13;	SU  T = 37 C = 1 N = color I = 15;	SU  T = 38 C = 1 N = color I = 15;	SU  T = 49 C = 1 N = color I = 15;	SU  T = 51 C = 1 N = color I = 4;	SU  T = 4 C = 2 N = color I = 13;	SU  T = 36 C = 2 N = color I = 15;	SU  T = 37 C = 2 N = color I = 13;	SU  T = 38 C = 2 N = color I = 15;	SU  T = 41 C = 2 N = color I = 13;	SU  T = 42 C = 2 N = color I = 13;	SU  T = 44 C = 2 N = color I = 13;	SU  T = 46 C = 2 N = color I = 7;	SU  T = 51 C = 2 N = color I = 4;	SU  C = 3 N = color I = 3;	SU  T = 1 C = 3 N = color I = 3;	SU  T = 2 C = 3 N = color I = 3;	SU  T = 3 C = 3 N = color I = 3;	SU  T = 4 C = 3 N = color I = 3;	SU  T = 5 C = 3 N = color I = 3;	SU  T = 6 C = 3 N = color I = 3;	SU  T = 7 C = 3 N = color I = 3;	SU  T = 8 C = 3 N = color I = 3;	SU  T = 9 C = 3 N = color I = 3;	SU  T = 10 C = 3 N = color I = 3;	SU  T = 11 C = 3 N = color I = 3;	SU  T = 12 C = 3 N = color I = 3;	SU  T = 13 C = 3 N = color I = 3;	SU  T = 14 C = 3 N = color I = 3;	SU  T = 15 C = 3 N = color I = 3;	SU  T = 16 C = 3 N = color I = 3;	SU  T = 17 C = 3 N = color I = 3;	SU  T = 18 C = 3 N = color I = 3;	SU  T = 19 C = 3 N = color I = 3;	SU  T = 20 C = 3 N = color I = 3;	SU  T = 21 C = 3 N = color I = 3;	SU  T = 22 C = 3 N = color I = 3;	SU  T = 23 C = 3 N = color I = 3;	SU  T = 24 C = 3 N = color I = 3;	SU  T = 25 C = 3 N = color I = 3;	SU  T = 26 C = 3 N = color I = 3;	SU  T = 27 C = 3 N = color I = 3;	SU  T = 28 C = 3 N = color I = 3;	SU  T = 29 C = 3 N = color I = 3;	SU  T = 30 C = 3 N = color I = 3;	SU  T = 31 C = 3 N = color I = 3;	SU  T = 32 C = 3 N = color I = 3;	SU  T = 33 C = 3 N = color I = 3;	SU  T = 34 C = 3 N = color I = 3;	SU  T = 35 C = 3 N = color I = 3;	SU  T = 36 C = 3 N = color I = 3;	SU  T = 37 C = 3 N = color I = 3;	SU  T = 38 C = 3 N = color I = 3;	SU  T = 39 C = 3 N = color I = 3;	SU  T = 40 C = 3 N = color I = 3;	SU  T = 41 C = 3 N = color I = 3;	SU  T = 42 C = 3 N = color I = 3;	SU  T = 43 C = 3 N = color I = 3;	SU  T = 44 C = 3 N = color I = 3;	SU  T = 45 C = 3 N = color I = 3;	SU  T = 46 C = 3 N = color I = 3;	SU  T = 47 C = 3 N = color I = 3;	SU  T = 48 C = 3 N = color I = 3;	SU  T = 49 C = 3 N = color I = 3;	SU  T = 50 C = 3 N = color I = 3;	SU  T = 51 C = 3 N = color I = 3;	SU  T = 52 C = 3 N = color I = 3;	SU  T = 53 C = 3 N = color I = 3;	SU  T = 4 C = 4 N = color I = 13;	SU  T = 6 C = 4 N = color I = 13;	SU  T = 10 C = 4 N = color I = 13;	SU  T = 37 C = 4 N = color I = 15;	SU  T = 38 C = 4 N = color I = 15;	SU  T = 40 C = 4 N = color I = 13;	SU  T = 41 C = 4 N = color I = 13;	SU  T = 42 C = 4 N = color I = 13;	SU  T = 51 C = 4 N = color I = 4;	SU  T = 8 C = 5 N = color I = 13;	SU  T = 10 C = 5 N = color I = 15;	SU  T = 41 C = 5 N = color I = 15;	SU  T = 48 C = 5 N = color I = 15;	SU  T = 51 C = 5 N = color I = 4;	SU  C = 6 N = color I = 5;	SU  T = 26 C = 6 N = color I = 7;	SU  T = 51 C = 6 N = color I = 4;	SU  C = 7 N = color I = 3;	SU  T = 1 C = 7 N = color I = 3;	SU  T = 2 C = 7 N = color I = 3;	SU  T = 3 C = 7 N = color I = 3;	SU  T = 4 C = 7 N = color I = 3;	SU  T = 5 C = 7 N = color I = 3;	SU  T = 6 C = 7 N = color I = 3;	SU  T = 7 C = 7 N = color I = 3;	SU  T = 8 C = 7 N = color I = 3;	SU  T = 9 C = 7 N = color I = 3;	SU  T = 10 C = 7 N = color I = 3;	SU  T = 11 C = 7 N = color I = 3;	SU  T = 12 C = 7 N = color I = 3;	SU  T = 13 C = 7 N = color I = 3;	SU  T = 14 C = 7 N = color I = 3;	SU  T = 15 C = 7 N = color I = 3;	SU  T = 16 C = 7 N = color I = 3;	SU  T = 17 C = 7 N = color I = 3;	SU  T = 18 C = 7 N = color I = 3;	SU  T = 19 C = 7 N = color I = 3;	SU  T = 20 C = 7 N = color I = 3;	SU  T = 21 C = 7 N = color I = 3;	SU  T = 22 C = 7 N = color I = 3;	SU  T = 23 C = 7 N = color I = 3;	SU  T = 24 C = 7 N = color I = 3;	SU  T = 25 C = 7 N = color I = 3;	SU  T = 26 C = 7 N = color I = 3;	SU  T = 27 C = 7 N = color I = 3;	SU  T = 28 C = 7 N = color I = 3;	SU  T = 29 C = 7 N = color I = 3;	SU  T = 30 C = 7 N = color I = 3;	SU  T = 31 C = 7 N = color I = 3;	SU  T = 32 C = 7 N = color I = 3;	SU  T = 33 C = 7 N = color I = 3;	SU  T = 34 C = 7 N = color I = 3;	SU  T = 35 C = 7 N = color I = 3;	SU  T = 36 C = 7 N = color I = 3;	SU  T = 37 C = 7 N = color I = 3;	SU  T = 38 C = 7 N = color I = 3;	SU  T = 39 C = 7 N = color I = 3;	SU  T = 40 C = 7 N = color I = 3;	SU  T = 41 C = 7 N = color I = 3;	SU  T = 42 C = 7 N = color I = 3;	SU  T = 43 C = 7 N = color I = 3;	SU  T = 44 C = 7 N = color I = 3;	SU  T = 45 C = 7 N = color I = 3;	SU  T = 46 C = 7 N = color I = 3;	SU  T = 47 C = 7 N = color I = 3;	SU  T = 48 C = 7 N = color I = 3;	SU  T = 49 C = 7 N = color I = 3;	SU  T = 50 C = 7 N = color I = 3;	SU  T = 51 C = 7 N = color I = 3;	SU  T = 52 C = 7 N = color I = 3;	SU  T = 53 C = 7 N = color I = 3;	SU  C = 8 N = color I = 3;	SU  T = 1 C = 8 N = color I = 3;	SU  T = 2 C = 8 N = color I = 3;	SU  T = 3 C = 8 N = color I = 3;	SU  T = 4 C = 8 N = color I = 3;	SU  T = 5 C = 8 N = color I = 3;	SU  T = 6 C = 8 N = color I = 3;	SU  T = 7 C = 8 N = color I = 3;	SU  T = 8 C = 8 N = color I = 3;	SU  T = 9 C = 8 N = color I = 3;	SU  T = 10 C = 8 N = color I = 3;	SU  T = 11 C = 8 N = color I = 3;	SU  T = 12 C = 8 N = color I = 3;	SU  T = 13 C = 8 N = color I = 3;	SU  T = 14 C = 8 N = color I = 3;	SU  T = 15 C = 8 N = color I = 3;	SU  T = 16 C = 8 N = color I = 3;	SU  T = 17 C = 8 N = color I = 3;	SU  T = 18 C = 8 N = color I = 3;	SU  T = 19 C = 8 N = color I = 3;	SU  T = 20 C = 8 N = color I = 3;	SU  T = 21 C = 8 N = color I = 3;	SU  T = 22 C = 8 N = color I = 3;	SU  T = 23 C = 8 N = color I = 3;	SU  T = 24 C = 8 N = color I = 3;	SU  T = 25 C = 8 N = color I = 3;	SU  T = 26 C = 8 N = color I = 3;	SU  T = 27 C = 8 N = color I = 3;	SU  T = 28 C = 8 N = color I = 3;	SU  T = 29 C = 8 N = color I = 3;	SU  T = 30 C = 8 N = color I = 3;	SU  T = 31 C = 8 N = color I = 3;	SU  T = 32 C = 8 N = color I = 3;	SU  T = 33 C = 8 N = color I = 3;	SU  T = 34 C = 8 N = color I = 3;	SU  T = 35 C = 8 N = color I = 3;	SU  T = 36 C = 8 N = color I = 3;	SU  T = 37 C = 8 N = color I = 3;	SU  T = 38 C = 8 N = color I = 3;	SU  T = 39 C = 8 N = color I = 3;	SU  T = 40 C = 8 N = color I = 3;	SU  T = 41 C = 8 N = color I = 3;	SU  T = 42 C = 8 N = color I = 3;	SU  T = 43 C = 8 N = color I = 3;	SU  T = 44 C = 8 N = color I = 3;	SU  T = 45 C = 8 N = color I = 3;	SU  T = 46 C = 8 N = color I = 3;	SU  T = 47 C = 8 N = color I = 3;	SU  T = 48 C = 8 N = color I = 3;	SU  T = 49 C = 8 N = color I = 3;	SU  T = 50 C = 8 N = color I = 3;	SU  T = 51 C = 8 N = color I = 3;	SU  T = 52 C = 8 N = color I = 3;	SU  T = 53 C = 8 N = color I = 3;	SU  T = 30 C = 9 N = color I = 15;	SU  T = 31 C = 9 N = color I = 15;	SU  T = 33 C = 9 N = color I = 13;	SU  T = 37 C = 9 N = color I = 15;	SU  T = 38 C = 9 N = color I = 15;	SU  T = 51 C = 9 N = color I = 4;	SU  T = 52 C = 9 N = color I = 15;	SU  T = 36 C = 10 N = color I = 15;	SU  T = 43 C = 10 N = color I = 13;	SU  T = 49 C = 10 N = color I = 15;	SU  T = 51 C = 10 N = color I = 4;	SU  T = 2 C = 11 N = color I = 17;	SU  T = 25 C = 11 N = color I = 7;	SU  T = 31 C = 11 N = color I = 17;	SU  T = 36 C = 11 N = color I = 17;	SU  T = 39 C = 11 N = color I = 15;	SU  T = 40 C = 11 N = color I = 17;	SU  T = 42 C = 11 N = color I = 13;	SU  T = 43 C = 11 N = color I = 13;	SU  T = 45 C = 11 N = color I = 17;	SU  T = 46 C = 11 N = color I = 7;	SU  T = 49 C = 11 N = color I = 17;	SU  T = 51 C = 11 N = color I = 4;	SU  T = 40 C = 12 N = color I = 13;	SU  T = 46 C = 12 N = color I = 7;	SU  T = 51 C = 12 N = color I = 4;	SU  T = 51 C = 13 N = color I = 4;	SU  T = 43 C = 14 N = color I = 13;	SU  T = 46 C = 14 N = color I = 7;	SU  T = 49 C = 14 N = color I = 15;	SU  T = 51 C = 14 N = color I = 4;	SU  C = 15 N = color I = 3;	SU  T = 1 C = 15 N = color I = 3;	SU  T = 2 C = 15 N = color I = 3;	SU  T = 3 C = 15 N = color I = 3;	SU  T = 4 C = 15 N = color I = 3;	SU  T = 5 C = 15 N = color I = 3;	SU  T = 6 C = 15 N = color I = 3;	SU  T = 7 C = 15 N = color I = 3;	SU  T = 8 C = 15 N = color I = 3;	SU  T = 9 C = 15 N = color I = 3;	SU  T = 10 C = 15 N = color I = 3;	SU  T = 11 C = 15 N = color I = 3;	SU  T = 12 C = 15 N = color I = 3;	SU  T = 13 C = 15 N = color I = 3;	SU  T = 14 C = 15 N = color I = 3;	SU  T = 15 C = 15 N = color I = 3;	SU  T = 16 C = 15 N = color I = 3;	SU  T = 17 C = 15 N = color I = 3;	SU  T = 18 C = 15 N = color I = 3;	SU  T = 19 C = 15 N = color I = 3;	SU  T = 20 C = 15 N = color I = 3;	SU  T = 21 C = 15 N = color I = 3;	SU  T = 22 C = 15 N = color I = 3;	SU  T = 23 C = 15 N = color I = 3;	SU  T = 24 C = 15 N = color I = 3;	SU  T = 25 C = 15 N = color I = 3;	SU  T = 26 C = 15 N = color I = 3;	SU  T = 27 C = 15 N = color I = 3;	SU  T = 28 C = 15 N = color I = 3;	SU  T = 29 C = 15 N = color I = 3;	SU  T = 30 C = 15 N = color I = 3;	SU  T = 31 C = 15 N = color I = 3;	SU  T = 32 C = 15 N = color I = 3;	SU  T = 33 C = 15 N = color I = 3;	SU  T = 34 C = 15 N = color I = 3;	SU  T = 35 C = 15 N = color I = 3;	SU  T = 36 C = 15 N = color I = 3;	SU  T = 37 C = 15 N = color I = 3;	SU  T = 38 C = 15 N = color I = 3;	SU  T = 39 C = 15 N = color I = 3;	SU  T = 40 C = 15 N = color I = 3;	SU  T = 41 C = 15 N = color I = 3;	SU  T = 42 C = 15 N = color I = 3;	SU  T = 43 C = 15 N = color I = 3;	SU  T = 44 C = 15 N = color I = 3;	SU  T = 45 C = 15 N = color I = 3;	SU  T = 46 C = 15 N = color I = 3;	SU  T = 47 C = 15 N = color I = 3;	SU  T = 48 C = 15 N = color I = 3;	SU  T = 49 C = 15 N = color I = 3;	SU  T = 50 C = 15 N = color I = 3;	SU  T = 51 C = 15 N = color I = 3;	SU  T = 52 C = 15 N = color I = 3;	SU  T = 53 C = 15 N = color I = 3;	SU  C = 16 N = color I = 3;	SU  T = 1 C = 16 N = color I = 3;	SU  T = 2 C = 16 N = color I = 3;	SU  T = 3 C = 16 N = color I = 3;	SU  T = 4 C = 16 N = color I = 3;	SU  T = 5 C = 16 N = color I = 3;	SU  T = 6 C = 16 N = color I = 3;	SU  T = 7 C = 16 N = color I = 3;	SU  T = 8 C = 16 N = color I = 3;	SU  T = 9 C = 16 N = color I = 3;	SU  T = 10 C = 16 N = color I = 3;	SU  T = 11 C = 16 N = color I = 3;	SU  T = 12 C = 16 N = color I = 3;	SU  T = 13 C = 16 N = color I = 3;	SU  T = 14 C = 16 N = color I = 3;	SU  T = 15 C = 16 N = color I = 3;	SU  T = 16 C = 16 N = color I = 3;	SU  T = 17 C = 16 N = color I = 3;	SU  T = 18 C = 16 N = color I = 3;	SU  T = 19 C = 16 N = color I = 3;	SU  T = 20 C = 16 N = color I = 3;	SU  T = 21 C = 16 N = color I = 3;	SU  T = 22 C = 16 N = color I = 3;	SU  T = 23 C = 16 N = color I = 3;	SU  T = 24 C = 16 N = color I = 3;	SU  T = 25 C = 16 N = color I = 3;	SU  T = 26 C = 16 N = color I = 3;	SU  T = 27 C = 16 N = color I = 3;	SU  T = 28 C = 16 N = color I = 3;	SU  T = 29 C = 16 N = color I = 3;	SU  T = 30 C = 16 N = color I = 3;	SU  T = 31 C = 16 N = color I = 3;	SU  T = 32 C = 16 N = color I = 3;	SU  T = 33 C = 16 N = color I = 3;	SU  T = 34 C = 16 N = color I = 3;	SU  T = 35 C = 16 N = color I = 3;	SU  T = 36 C = 16 N = color I = 3;	SU  T = 37 C = 16 N = color I = 3;	SU  T = 38 C = 16 N = color I = 3;	SU  T = 39 C = 16 N = color I = 3;	SU  T = 40 C = 16 N = color I = 3;	SU  T = 41 C = 16 N = color I = 3;	SU  T = 42 C = 16 N = color I = 3;	SU  T = 43 C = 16 N = color I = 3;	SU  T = 44 C = 16 N = color I = 3;	SU  T = 45 C = 16 N = color I = 3;	SU  T = 46 C = 16 N = color I = 3;	SU  T = 47 C = 16 N = color I = 3;	SU  T = 48 C = 16 N = color I = 3;	SU  T = 49 C = 16 N = color I = 3;	SU  T = 50 C = 16 N = color I = 3;	SU  T = 51 C = 16 N = color I = 3;	SU  T = 52 C = 16 N = color I = 3;	SU  T = 53 C = 16 N = color I = 3;	SU  T = 33 C = 17 N = color I = 4;	SU  T = 41 C = 17 N = color I = 14;	SU  T = 43 C = 17 N = color I = 15;	SU  T = 46 C = 17 N = color I = 7;	SU  T = 51 C = 17 N = color I = 4;	SU  T = 53 C = 17 N = color I = 14;	SU  T = 36 C = 18 N = color I = 15;	SU  T = 40 C = 18 N = color I = 13;	SU  T = 41 C = 18 N = color I = 15;	SU  T = 46 C = 18 N = color I = 7;	SU  T = 49 C = 18 N = color I = 13;	SU  T = 51 C = 18 N = color I = 15;	SU  T = 41 C = 19 N = color I = 14;	SU  T = 43 C = 19 N = color I = 13;	SU  T = 46 C = 19 N = color I = 7;	SU  T = 51 C = 19 N = color I = 4;	SU  T = 53 C = 19 N = color I = 14;	SU  T = 36 C = 20 N = color I = 15;	SU  T = 46 C = 20 N = color I = 7;	SU  T = 49 C = 20 N = color I = 13;	SU  T = 51 C = 20 N = color I = 15;	SU  T = 10 C = 21 N = color I = 4;	SU  T = 39 C = 21 N = color I = 13;	SU  T = 46 C = 21 N = color I = 7;	SU  T = 51 C = 21 N = color I = 4;	SU  T = 36 C = 22 N = color I = 13;	SU  T = 41 C = 22 N = color I = 15;	SU  T = 46 C = 22 N = color I = 7;	SU  T = 48 C = 22 N = color I = 13;	SU  T = 51 C = 22 N = color I = 4;	SU  T = 4 C = 23 N = color I = 13;	SU  T = 41 C = 23 N = color I = 13;	SU  T = 42 C = 23 N = color I = 15;	SU  T = 44 C = 23 N = color I = 15;	SU  T = 45 C = 23 N = color I = 13;	SU  T = 46 C = 23 N = color I = 7;	SU  T = 51 C = 23 N = color I = 15;	SU  T = 10 C = 24 N = color I = 13;	SU  T = 11 C = 24 N = color I = 4;	SU  T = 36 C = 24 N = color I = 15;	SU  T = 42 C = 24 N = color I = 15;	SU  T = 44 C = 24 N = color I = 15;	SU  T = 45 C = 24 N = color I = 13;	SU  T = 46 C = 24 N = color I = 7;	SU  T = 50 C = 24 N = color I = 15;	SU  T = 51 C = 24 N = color I = 4;	SU  T = 46 C = 25 N = color I = 7;	SU  T = 51 C = 25 N = color I = 4;	SU  C = 26 N = color I = 5;	SU  T = 10 C = 26 N = color I = 7;	SU  T = 15 C = 26 N = color I = 7;	SU  T = 26 C = 26 N = color I = 7;	SU  T = 51 C = 26 N = color I = 4;	SU  T = 4 C = 27 N = color I = 13;	SU  T = 6 C = 27 N = color I = 13;	SU  T = 8 C = 27 N = color I = 13;	SU  T = 10 C = 27 N = color I = 13;	SU  T = 11 C = 27 N = color I = 13;	SU  T = 15 C = 27 N = color I = 13;	SU  T = 21 C = 27 N = color I = 13;	SU  T = 22 C = 27 N = color I = 13;	SU  T = 26 C = 27 N = color I = 13;	SU  T = 30 C = 27 N = color I = 17;	SU  T = 31 C = 27 N = color I = 17;	SU  T = 32 C = 27 N = color I = 17;	SU  T = 33 C = 27 N = color I = 17;	SU  T = 34 C = 27 N = color I = 17;	SU  T = 35 C = 27 N = color I = 17;	SU  T = 36 C = 27 N = color I = 17;	SU  T = 39 C = 27 N = color I = 13;	SU  T = 40 C = 27 N = color I = 17;	SU  T = 41 C = 27 N = color I = 13;	SU  T = 42 C = 27 N = color I = 15;	SU  T = 43 C = 27 N = color I = 17;	SU  T = 44 C = 27 N = color I = 17;	SU  T = 45 C = 27 N = color I = 17;	SU  T = 46 C = 27 N = color I = 17;	SU  T = 47 C = 27 N = color I = 17;	SU  T = 48 C = 27 N = color I = 17;	SU  T = 49 C = 27 N = color I = 17;	SU  T = 50 C = 27 N = color I = 17;	SU  T = 51 C = 27 N = color I = 15;	SU  T = 52 C = 27 N = color I = 17;	SU  T = 51 C = 28 N = color I = 4;	SU  T = 30 C = 29 N = color I = 13;	SU  T = 45 C = 29 N = color I = 13;	SU  T = 51 C = 29 N = color I = 4;	SU  T = 51 C = 30 N = color I = 4;	SU  T = 10 C = 31 N = color I = 4;	SU  T = 30 C = 31 N = color I = 13;	SU  T = 37 C = 31 N = color I = 15;	SU  T = 38 C = 31 N = color I = 15;	SU  T = 51 C = 31 N = color I = 4;	SU  T = 35 C = 32 N = color I = 4;	SU  T = 37 C = 32 N = color I = 15;	SU  T = 38 C = 32 N = color I = 15;	SU  T = 51 C = 32 N = color I = 4;	SU  T = 33 C = 33 N = color I = 7;	SU  T = 35 C = 33 N = color I = 4;	SU  T = 37 C = 33 N = color I = 15;	SU  T = 38 C = 33 N = color I = 15;	SU  T = 41 C = 33 N = color I = 13;	SU  T = 43 C = 33 N = color I = 13;	SU  T = 44 C = 33 N = color I = 13;	SU  T = 50 C = 33 N = color I = 13;	SU  T = 51 C = 33 N = color I = 4;	SU  T = 51 C = 34 N = color I = 4;	SU  C = 35 N = color I = 4;	SU  T = 1 C = 35 N = color I = 4;	SU  T = 2 C = 35 N = color I = 4;	SU  T = 3 C = 35 N = color I = 4;	SU  T = 4 C = 35 N = color I = 4;	SU  T = 5 C = 35 N = color I = 4;	SU  T = 6 C = 35 N = color I = 13;	SU  T = 7 C = 35 N = color I = 4;	SU  T = 8 C = 35 N = color I = 4;	SU  T = 9 C = 35 N = color I = 15;	SU  T = 10 C = 35 N = color I = 4;	SU  T = 11 C = 35 N = color I = 13;	SU  T = 12 C = 35 N = color I = 4;	SU  T = 13 C = 35 N = color I = 15;	SU  T = 14 C = 35 N = color I = 13;	SU  T = 15 C = 35 N = color I = 4;	SU  T = 16 C = 35 N = color I = 4;	SU  T = 17 C = 35 N = color I = 4;	SU  T = 18 C = 35 N = color I = 4;	SU  T = 19 C = 35 N = color I = 4;	SU  T = 20 C = 35 N = color I = 4;	SU  T = 21 C = 35 N = color I = 13;	SU  T = 22 C = 35 N = color I = 4;	SU  T = 23 C = 35 N = color I = 4;	SU  T = 24 C = 35 N = color I = 4;	SU  T = 25 C = 35 N = color I = 4;	SU  T = 26 C = 35 N = color I = 13;	SU  T = 27 C = 35 N = color I = 4;	SU  T = 28 C = 35 N = color I = 4;	SU  T = 29 C = 35 N = color I = 4;	SU  T = 30 C = 35 N = color I = 15;	SU  T = 31 C = 35 N = color I = 15;	SU  T = 32 C = 35 N = color I = 15;	SU  T = 33 C = 35 N = color I = 4;	SU  T = 34 C = 35 N = color I = 15;	SU  T = 35 C = 35 N = color I = 13;	SU  T = 36 C = 35 N = color I = 4;	SU  T = 37 C = 35 N = color I = 4;	SU  T = 39 C = 35 N = color I = 15;	SU  T = 40 C = 35 N = color I = 4;	SU  T = 41 C = 35 N = color I = 4;	SU  T = 42 C = 35 N = color I = 15;	SU  T = 43 C = 35 N = color I = 4;	SU  T = 44 C = 35 N = color I = 4;	SU  T = 46 C = 35 N = color I = 7;	SU  T = 47 C = 35 N = color I = 15;	SU  T = 48 C = 35 N = color I = 15;	SU  T = 49 C = 35 N = color I = 4;	SU  T = 50 C = 35 N = color I = 4;	SU  T = 51 C = 35 N = color I = 4;	SU  T = 52 C = 35 N = color I = 13;	SU  T = 6 C = 36 N = color I = 15;	SU  T = 8 C = 36 N = color I = 15;	SU  T = 10 C = 36 N = color I = 13;	SU  T = 11 C = 36 N = color I = 15;	SU  T = 15 C = 36 N = color I = 13;	SU  T = 21 C = 36 N = color I = 15;	SU  T = 22 C = 36 N = color I = 13;	SU  T = 26 C = 36 N = color I = 15;	SU  T = 29 C = 36 N = color I = 4;	SU  T = 30 C = 36 N = color I = 15;	SU  T = 31 C = 36 N = color I = 15;	SU  T = 32 C = 36 N = color I = 15;	SU  T = 33 C = 36 N = color I = 13;	SU  T = 34 C = 36 N = color I = 15;	SU  T = 35 C = 36 N = color I = 13;	SU  T = 39 C = 36 N = color I = 15;	SU  T = 41 C = 36 N = color I = 13;	SU  T = 42 C = 36 N = color I = 15;	SU  T = 46 C = 36 N = color I = 7;	SU  T = 47 C = 36 N = color I = 15;	SU  T = 48 C = 36 N = color I = 15;	SU  T = 49 C = 36 N = color I = 13;	SU  T = 51 C = 36 N = color I = 4;	SU  T = 52 C = 36 N = color I = 13;	SU  T = 4 C = 37 N = color I = 13;	SU  T = 6 C = 37 N = color I = 15;	SU  T = 8 C = 37 N = color I = 15;	SU  T = 10 C = 37 N = color I = 15;	SU  T = 11 C = 37 N = color I = 15;	SU  T = 15 C = 37 N = color I = 13;	SU  T = 21 C = 37 N = color I = 15;	SU  T = 26 C = 37 N = color I = 15;	SU  T = 29 C = 37 N = color I = 4;	SU  T = 30 C = 37 N = color I = 15;	SU  T = 31 C = 37 N = color I = 15;	SU  T = 32 C = 37 N = color I = 15;	SU  T = 34 C = 37 N = color I = 15;	SU  T = 39 C = 37 N = color I = 15;	SU  T = 41 C = 37 N = color I = 13;	SU  T = 42 C = 37 N = color I = 15;	SU  T = 46 C = 37 N = color I = 7;	SU  T = 47 C = 37 N = color I = 15;	SU  T = 48 C = 37 N = color I = 15;	SU  T = 49 C = 37 N = color I = 4;	SU  T = 51 C = 37 N = color I = 4;	SU  T = 6 C = 38 N = color I = 4;	SU  T = 29 C = 38 N = color I = 4;	SU  T = 32 C = 38 N = color I = 13;	SU  T = 37 C = 38 N = color I = 15;	SU  T = 38 C = 38 N = color I = 15;	SU  T = 48 C = 38 N = color I = 13;	SU  T = 50 C = 38 N = color I = 13;	SU  T = 51 C = 38 N = color I = 4;	SU  T = 31 C = 39 N = color I = 13;	SU  T = 32 C = 39 N = color I = 13;	SU  T = 33 C = 39 N = color I = 13;	SU  T = 37 C = 39 N = color I = 15;	SU  T = 38 C = 39 N = color I = 13;	SU  T = 46 C = 39 N = color I = 15;	SU  T = 47 C = 39 N = color I = 4;	SU  T = 48 C = 39 N = color I = 13;	SU  T = 50 C = 39 N = color I = 4;	SU  T = 51 C = 39 N = color I = 4;	SU  T = 31 C = 40 N = color I = 13;	SU  T = 33 C = 40 N = color I = 15;	SU  T = 35 C = 40 N = color I = 15;	SU  T = 38 C = 40 N = color I = 13;	SU  T = 41 C = 40 N = color I = 4;	SU  T = 44 C = 40 N = color I = 15;	SU  T = 49 C = 40 N = color I = 15;	SU  T = 51 C = 40 N = color I = 4;	SU  T = 31 C = 41 N = color I = 4;	SU  T = 32 C = 41 N = color I = 15;	SU  T = 33 C = 41 N = color I = 15;	SU  T = 38 C = 41 N = color I = 15;	SU  T = 41 C = 41 N = color I = 15;	SU  T = 47 C = 41 N = color I = 13;	SU  T = 51 C = 41 N = color I = 4;	SU  T = 11 C = 42 N = color I = 4;	SU  T = 51 C = 42 N = color I = 4;	SU  T = 11 C = 43 N = color I = 13;	SU  T = 15 C = 43 N = color I = 13;	SU  T = 21 C = 43 N = color I = 13;	SU  T = 31 C = 43 N = color I = 13;	SU  T = 33 C = 43 N = color I = 13;	SU  T = 37 C = 43 N = color I = 15;	SU  T = 38 C = 43 N = color I = 15;	SU  T = 43 C = 43 N = color I = 13;	SU  T = 46 C = 43 N = color I = 13;	SU  T = 47 C = 43 N = color I = 13;	SU  T = 51 C = 43 N = color I = 4;	SU  C = 44 N = color I = 4;	SU  T = 1 C = 44 N = color I = 4;	SU  T = 2 C = 44 N = color I = 4;	SU  T = 3 C = 44 N = color I = 4;	SU  T = 4 C = 44 N = color I = 4;	SU  T = 5 C = 44 N = color I = 4;	SU  T = 6 C = 44 N = color I = 4;	SU  T = 7 C = 44 N = color I = 4;	SU  T = 8 C = 44 N = color I = 13;	SU  T = 9 C = 44 N = color I = 4;	SU  T = 10 C = 44 N = color I = 4;	SU  T = 11 C = 44 N = color I = 4;	SU  T = 12 C = 44 N = color I = 4;	SU  T = 13 C = 44 N = color I = 4;	SU  T = 14 C = 44 N = color I = 4;	SU  T = 15 C = 44 N = color I = 4;	SU  T = 16 C = 44 N = color I = 4;	SU  T = 17 C = 44 N = color I = 4;	SU  T = 18 C = 44 N = color I = 4;	SU  T = 19 C = 44 N = color I = 4;	SU  T = 20 C = 44 N = color I = 4;	SU  T = 21 C = 44 N = color I = 4;	SU  T = 22 C = 44 N = color I = 4;	SU  T = 23 C = 44 N = color I = 4;	SU  T = 24 C = 44 N = color I = 4;	SU  T = 25 C = 44 N = color I = 4;	SU  T = 27 C = 44 N = color I = 4;	SU  T = 28 C = 44 N = color I = 4;	SU  T = 29 C = 44 N = color I = 4;	SU  T = 30 C = 44 N = color I = 13;	SU  T = 31 C = 44 N = color I = 4;	SU  T = 32 C = 44 N = color I = 4;	SU  T = 33 C = 44 N = color I = 13;	SU  T = 35 C = 44 N = color I = 4;	SU  T = 36 C = 44 N = color I = 13;	SU  T = 37 C = 44 N = color I = 4;	SU  T = 39 C = 44 N = color I = 4;	SU  T = 40 C = 44 N = color I = 4;	SU  T = 41 C = 44 N = color I = 4;	SU  T = 42 C = 44 N = color I = 13;	SU  T = 43 C = 44 N = color I = 4;	SU  T = 44 C = 44 N = color I = 4;	SU  T = 46 C = 44 N = color I = 4;	SU  T = 47 C = 44 N = color I = 13;	SU  T = 48 C = 44 N = color I = 4;	SU  T = 49 C = 44 N = color I = 13;	SU  T = 50 C = 44 N = color I = 15;	SU  T = 51 C = 44 N = color I = 15;	SU  T = 52 C = 44 N = color I = 4;	SU  T = 31 C = 45 N = color I = 4;	SU  T = 46 C = 45 N = color I = 13;	SU  T = 49 C = 45 N = color I = 13;	SU  T = 51 C = 45 N = color I = 4;	SU  T = 11 C = 46 N = color I = 4;	SU  T = 31 C = 46 N = color I = 15;	SU  T = 46 C = 46 N = color I = 7;	SU  T = 51 C = 46 N = color I = 4;	SU  T = 8 C = 47 N = color I = 13;	SU  T = 11 C = 47 N = color I = 13;	SU  T = 31 C = 47 N = color I = 13;	SU  T = 33 C = 47 N = color I = 13;	SU  T = 41 C = 47 N = color I = 13;	SU  T = 43 C = 47 N = color I = 15;	SU  T = 46 C = 47 N = color I = 13;	SU  T = 47 C = 47 N = color I = 4;	SU  T = 49 C = 47 N = color I = 13;	SU  T = 50 C = 47 N = color I = 13;	SU  T = 51 C = 47 N = color I = 4;	SU  T = 52 C = 47 N = color I = 15;	SU  T = 11 C = 48 N = color I = 15;	SU  T = 31 C = 48 N = color I = 13;	SU  T = 36 C = 48 N = color I = 15;	SU  T = 44 C = 48 N = color I = 13;	SU  T = 46 C = 48 N = color I = 13;	SU  T = 51 C = 48 N = color I = 13;	SU  T = 52 C = 48 N = color I = 13;	SU  T = 31 C = 49 N = color I = 4;	SU  T = 47 C = 49 N = color I = 13;	SU  T = 51 C = 49 N = color I = 4;	SU  T = 46 C = 50 N = color I = 13;	SU  T = 47 C = 50 N = color I = 13;	SU  T = 48 C = 50 N = color I = 15;	SU  T = 51 C = 50 N = color I = 4;	SU  T = 40 C = 51 N = color I = 13;	SU  T = 46 C = 51 N = color I = 15;	SU  T = 51 C = 51 N = color I = 4;	SU  T = 15 C = 52 N = color I = 4;	SU  T = 31 C = 52 N = color I = 4;	SU  T = 40 C = 52 N = color I = 13;	SU  T = 46 C = 52 N = color I = 13;	SU  T = 51 C = 52 N = color I = 4;	SU  T = 31 C = 53 N = color I = 13;	SU  T = 40 C = 53 N = color I = 13;	SU  T = 46 C = 53 N = color I = 13;	SU  T = 51 C = 53 N = color I = 4;	SU  T = 21 C = 54 N = color I = 4;	SU  T = 46 C = 54 N = color I = 7;	SU  T = 51 C = 54 N = color I = 4;	SU  T = 30 C = 55 N = color I = 17;	SU  T = 51 C = 55 N = color I = 4;	SU  C = 56 N = color I = 3;	SU  T = 1 C = 56 N = color I = 3;	SU  T = 2 C = 56 N = color I = 3;	SU  T = 3 C = 56 N = color I = 3;	SU  T = 4 C = 56 N = color I = 3;	SU  T = 5 C = 56 N = color I = 3;	SU  T = 6 C = 56 N = color I = 3;	SU  T = 7 C = 56 N = color I = 3;	SU  T = 8 C = 56 N = color I = 3;	SU  T = 9 C = 56 N = color I = 3;	SU  T = 10 C = 56 N = color I = 3;	SU  T = 11 C = 56 N = color I = 3;	SU  T = 12 C = 56 N = color I = 3;	SU  T = 13 C = 56 N = color I = 3;	SU  T = 14 C = 56 N = color I = 3;	SU  T = 15 C = 56 N = color I = 3;	SU  T = 16 C = 56 N = color I = 3;	SU  T = 17 C = 56 N = color I = 3;	SU  T = 18 C = 56 N = color I = 3;	SU  T = 19 C = 56 N = color I = 3;	SU  T = 20 C = 56 N = color I = 3;	SU  T = 21 C = 56 N = color I = 3;	SU  T = 22 C = 56 N = color I = 3;	SU  T = 23 C = 56 N = color I = 3;	SU  T = 24 C = 56 N = color I = 3;	SU  T = 25 C = 56 N = color I = 3;	SU  T = 26 C = 56 N = color I = 3;	SU  T = 27 C = 56 N = color I = 3;	SU  T = 28 C = 56 N = color I = 3;	SU  T = 29 C = 56 N = color I = 3;	SU  T = 30 C = 56 N = color I = 3;	SU  T = 31 C = 56 N = color I = 3;	SU  T = 32 C = 56 N = color I = 3;	SU  T = 33 C = 56 N = color I = 3;	SU  T = 34 C = 56 N = color I = 3;	SU  T = 35 C = 56 N = color I = 3;	SU  T = 36 C = 56 N = color I = 3;	SU  T = 37 C = 56 N = color I = 3;	SU  T = 38 C = 56 N = color I = 3;	SU  T = 39 C = 56 N = color I = 3;	SU  T = 40 C = 56 N = color I = 3;	SU  T = 41 C = 56 N = color I = 3;	SU  T = 42 C = 56 N = color I = 3;	SU  T = 43 C = 56 N = color I = 3;	SU  T = 44 C = 56 N = color I = 3;	SU  T = 45 C = 56 N = color I = 3;	SU  T = 46 C = 56 N = color I = 3;	SU  T = 47 C = 56 N = color I = 3;	SU  T = 48 C = 56 N = color I = 3;	SU  T = 49 C = 56 N = color I = 3;	SU  T = 50 C = 56 N = color I = 3;	SU  T = 51 C = 56 N = color I = 3;	SU  T = 52 C = 56 N = color I = 3;	SU  T = 53 C = 56 N = color I = 3;	SU  T = 46 C = 57 N = color I = 7;	SU  T = 51 C = 57 N = color I = 4;	SU  T = 37 C = 58 N = color I = 15;	SU  T = 38 C = 58 N = color I = 15;	SU  T = 46 C = 58 N = color I = 7;	SU  T = 51 C = 58 N = color I = 4;	SU  T = 37 C = 59 N = color I = 15;	SU  T = 38 C = 59 N = color I = 15;	SU  T = 46 C = 59 N = color I = 7;	SU  T = 51 C = 59 N = color I = 4;	SU  T = 1 C = 60 N = color I = 7;	SU  T = 2 C = 60 N = color I = 7;	SU  T = 3 C = 60 N = color I = 7;	SU  T = 4 C = 60 N = color I = 7;	SU  T = 5 C = 60 N = color I = 7;	SU  T = 6 C = 60 N = color I = 7;	SU  T = 7 C = 60 N = color I = 12;	SU  T = 8 C = 60 N = color I = 7;	SU  T = 9 C = 60 N = color I = 7;	SU  T = 10 C = 60 N = color I = 7;	SU  T = 11 C = 60 N = color I = 7;	SU  T = 12 C = 60 N = color I = 7;	SU  T = 13 C = 60 N = color I = 7;	SU  T = 14 C = 60 N = color I = 7;	SU  T = 15 C = 60 N = color I = 7;	SU  T = 16 C = 60 N = color I = 7;	SU  T = 17 C = 60 N = color I = 7;	SU  T = 18 C = 60 N = color I = 7;	SU  T = 19 C = 60 N = color I = 7;	SU  T = 20 C = 60 N = color I = 7;	SU  T = 21 C = 60 N = color I = 7;	SU  T = 22 C = 60 N = color I = 7;	SU  T = 23 C = 60 N = color I = 7;	SU  T = 24 C = 60 N = color I = 7;	SU  T = 25 C = 60 N = color I = 7;	SU  T = 26 C = 60 N = color I = 7;	SU  T = 27 C = 60 N = color I = 7;	SU  T = 28 C = 60 N = color I = 7;	SU  T = 29 C = 60 N = color I = 7;	SU  T = 30 C = 60 N = color I = 7;	SU  T = 31 C = 60 N = color I = 7;	SU  T = 32 C = 60 N = color I = 7;	SU  T = 33 C = 60 N = color I = 7;	SU  T = 34 C = 60 N = color I = 7;	SU  T = 35 C = 60 N = color I = 7;	SU  T = 36 C = 60 N = color I = 7;	SU  T = 37 C = 60 N = color I = 15;	SU  T = 38 C = 60 N = color I = 15;	SU  T = 39 C = 60 N = color I = 7;	SU  T = 40 C = 60 N = color I = 13;	SU  T = 41 C = 60 N = color I = 7;	SU  T = 42 C = 60 N = color I = 7;	SU  T = 43 C = 60 N = color I = 7;	SU  T = 44 C = 60 N = color I = 7;	SU  T = 45 C = 60 N = color I = 7;	SU  T = 46 C = 60 N = color I = 7;	SU  T = 47 C = 60 N = color I = 7;	SU  T = 48 C = 60 N = color I = 7;	SU  T = 49 C = 60 N = color I = 7;	SU  T = 50 C = 60 N = color I = 7;	SU  T = 51 C = 60 N = color I = 15;	SU  T = 52 C = 60 N = color I = 7;	SU  T = 53 C = 60 N = color I = 7;	SU  T = 1 C = 61 N = color I = 7;	SU  T = 2 C = 61 N = color I = 12;	SU  T = 3 C = 61 N = color I = 7;	SU  T = 4 C = 61 N = color I = 7;	SU  T = 5 C = 61 N = color I = 7;	SU  T = 6 C = 61 N = color I = 7;	SU  T = 7 C = 61 N = color I = 7;	SU  T = 8 C = 61 N = color I = 7;	SU  T = 9 C = 61 N = color I = 7;	SU  T = 10 C = 61 N = color I = 7;	SU  T = 11 C = 61 N = color I = 7;	SU  T = 12 C = 61 N = color I = 7;	SU  T = 13 C = 61 N = color I = 7;	SU  T = 14 C = 61 N = color I = 7;	SU  T = 15 C = 61 N = color I = 7;	SU  T = 16 C = 61 N = color I = 7;	SU  T = 17 C = 61 N = color I = 7;	SU  T = 18 C = 61 N = color I = 7;	SU  T = 19 C = 61 N = color I = 7;	SU  T = 20 C = 61 N = color I = 7;	SU  T = 21 C = 61 N = color I = 7;	SU  T = 22 C = 61 N = color I = 7;	SU  T = 23 C = 61 N = color I = 7;	SU  T = 24 C = 61 N = color I = 12;	SU  T = 25 C = 61 N = color I = 7;	SU  T = 26 C = 61 N = color I = 7;	SU  T = 27 C = 61 N = color I = 7;	SU  T = 28 C = 61 N = color I = 7;	SU  T = 29 C = 61 N = color I = 15;	SU  T = 30 C = 61 N = color I = 7;	SU  T = 31 C = 61 N = color I = 7;	SU  T = 32 C = 61 N = color I = 7;	SU  T = 33 C = 61 N = color I = 7;	SU  T = 34 C = 61 N = color I = 7;	SU  T = 35 C = 61 N = color I = 7;	SU  T = 36 C = 61 N = color I = 7;	SU  T = 37 C = 61 N = color I = 7;	SU  T = 38 C = 61 N = color I = 7;	SU  T = 39 C = 61 N = color I = 15;	SU  T = 40 C = 61 N = color I = 13;	SU  T = 41 C = 61 N = color I = 7;	SU  T = 42 C = 61 N = color I = 7;	SU  T = 43 C = 61 N = color I = 7;	SU  T = 44 C = 61 N = color I = 7;	SU  T = 45 C = 61 N = color I = 7;	SU  T = 46 C = 61 N = color I = 7;	SU  T = 47 C = 61 N = color I = 7;	SU  T = 48 C = 61 N = color I = 7;	SU  T = 49 C = 61 N = color I = 7;	SU  T = 50 C = 61 N = color I = 7;	SU  T = 51 C = 61 N = color I = 7;	SU  T = 52 C = 61 N = color I = 7;	SU  T = 53 C = 61 N = color I = 7;	SU  T = 38 C = 62 N = color I = 15;	SU  T = 41 C = 62 N = color I = 13;	SU  T = 43 C = 62 N = color I = 13;	SU  T = 44 C = 62 N = color I = 13;	SU  T = 46 C = 62 N = color I = 15;	SU  T = 51 C = 62 N = color I = 4;	SU  T = 38 C = 63 N = color I = 15;	SU  T = 46 C = 63 N = color I = 13;	SU  T = 49 C = 63 N = color I = 13;	SU  T = 51 C = 63 N = color I = 4;	SU  T = 29 C = 64 N = color I = 4;	SU  T = 40 C = 64 N = color I = 13;	SU  T = 45 C = 64 N = color I = 13;	SU  T = 46 C = 64 N = color I = 13;	SU  T = 51 C = 64 N = color I = 4;	SU  C = 65 N = color I = 3;	SU  T = 1 C = 65 N = color I = 3;	SU  T = 2 C = 65 N = color I = 3;	SU  T = 3 C = 65 N = color I = 3;	SU  T = 4 C = 65 N = color I = 3;	SU  T = 5 C = 65 N = color I = 3;	SU  T = 6 C = 65 N = color I = 3;	SU  T = 7 C = 65 N = color I = 3;	SU  T = 8 C = 65 N = color I = 3;	SU  T = 9 C = 65 N = color I = 3;	SU  T = 10 C = 65 N = color I = 3;	SU  T = 11 C = 65 N = color I = 3;	SU  T = 12 C = 65 N = color I = 3;	SU  T = 13 C = 65 N = color I = 3;	SU  T = 14 C = 65 N = color I = 3;	SU  T = 15 C = 65 N = color I = 3;	SU  T = 16 C = 65 N = color I = 3;	SU  T = 17 C = 65 N = color I = 3;	SU  T = 18 C = 65 N = color I = 3;	SU  T = 19 C = 65 N = color I = 3;	SU  T = 20 C = 65 N = color I = 3;	SU  T = 21 C = 65 N = color I = 3;	SU  T = 22 C = 65 N = color I = 3;	SU  T = 23 C = 65 N = color I = 3;	SU  T = 24 C = 65 N = color I = 3;	SU  T = 25 C = 65 N = color I = 3;	SU  T = 26 C = 65 N = color I = 3;	SU  T = 27 C = 65 N = color I = 3;	SU  T = 28 C = 65 N = color I = 3;	SU  T = 29 C = 65 N = color I = 3;	SU  T = 30 C = 65 N = color I = 3;	SU  T = 31 C = 65 N = color I = 3;	SU  T = 32 C = 65 N = color I = 3;	SU  T = 33 C = 65 N = color I = 3;	SU  T = 34 C = 65 N = color I = 3;	SU  T = 35 C = 65 N = color I = 3;	SU  T = 36 C = 65 N = color I = 3;	SU  T = 37 C = 65 N = color I = 3;	SU  T = 38 C = 65 N = color I = 3;	SU  T = 39 C = 65 N = color I = 3;	SU  T = 40 C = 65 N = color I = 3;	SU  T = 41 C = 65 N = color I = 3;	SU  T = 42 C = 65 N = color I = 3;	SU  T = 43 C = 65 N = color I = 3;	SU  T = 44 C = 65 N = color I = 3;	SU  T = 45 C = 65 N = color I = 3;	SU  T = 46 C = 65 N = color I = 3;	SU  T = 47 C = 65 N = color I = 3;	SU  T = 48 C = 65 N = color I = 3;	SU  T = 49 C = 65 N = color I = 3;	SU  T = 50 C = 65 N = color I = 3;	SU  T = 51 C = 65 N = color I = 3;	SU  T = 52 C = 65 N = color I = 3;	SU  T = 53 C = 65 N = color I = 3;	SU  T = 37 C = 66 N = color I = 15;	SU  T = 38 C = 66 N = color I = 15;	SU  T = 46 C = 66 N = color I = 7;	SU  T = 51 C = 66 N = color I = 4;	SU  T = 37 C = 67 N = color I = 4;	SU  T = 49 C = 67 N = color I = 13;	SU  T = 51 C = 67 N = color I = 4;	SU  T = 37 C = 68 N = color I = 15;	SU  T = 38 C = 68 N = color I = 15;	SU  T = 42 C = 68 N = color I = 13;	SU  T = 46 C = 68 N = color I = 13;	SU  T = 51 C = 68 N = color I = 4;	SU  T = 44 C = 69 N = color I = 13;	SU  T = 46 C = 69 N = color I = 7;	SU  T = 51 C = 69 N = color I = 4;	SU  T = 42 C = 70 N = color I = 4;	SU  T = 44 C = 70 N = color I = 15;	SU  T = 46 C = 70 N = color I = 15;	SU  T = 51 C = 70 N = color I = 4;	SU  T = 21 C = 71 N = color I = 4;	SU  T = 45 C = 71 N = color I = 13;	SU  T = 46 C = 71 N = color I = 13;	SU  T = 51 C = 71 N = color I = 4;	SU  T = 51 C = 72 N = color I = 4;	SU  T = 46 C = 73 N = color I = 13;	SU  T = 51 C = 73 N = color I = 13;	SU  T = 46 C = 74 N = color I = 13;	SU  T = 47 C = 74 N = color I = 15;	SU  T = 51 C = 74 N = color I = 13;	SU  T = 15 C = 75 N = color I = 4;	SU  T = 41 C = 75 N = color I = 13;	SU  T = 46 C = 75 N = color I = 7;	SU  T = 51 C = 75 N = color I = 4;	SU  T = 10 C = 76 N = color I = 15;	SU  T = 16 C = 76 N = color I = 15;	SU  T = 41 C = 76 N = color I = 13;	SU  T = 46 C = 76 N = color I = 7;	SU  T = 47 C = 76 N = color I = 4;	SU  T = 51 C = 76 N = color I = 4;	SU  T = 4 C = 77 N = color I = 13;	SU  T = 6 C = 77 N = color I = 7;	SU  T = 8 C = 77 N = color I = 7;	SU  T = 10 C = 77 N = color I = 13;	SU  T = 11 C = 77 N = color I = 13;	SU  T = 15 C = 77 N = color I = 13;	SU  T = 21 C = 77 N = color I = 15;	SU  T = 22 C = 77 N = color I = 13;	SU  T = 26 C = 77 N = color I = 7;	SU  T = 44 C = 77 N = color I = 15;	SU  T = 51 C = 77 N = color I = 4;	SU  T = 44 C = 78 N = color I = 7;	SU  T = 45 C = 78 N = color I = 15;	SU  T = 47 C = 78 N = color I = 7;	SU  T = 51 C = 78 N = color I = 7;	SU  T = 4 C = 79 N = color I = 13;	SU  T = 6 C = 79 N = color I = 13;	SU  T = 8 C = 79 N = color I = 7;	SU  T = 10 C = 79 N = color I = 15;	SU  T = 11 C = 79 N = color I = 13;	SU  T = 15 C = 79 N = color I = 15;	SU  T = 21 C = 79 N = color I = 15;	SU  T = 22 C = 79 N = color I = 13;	SU  T = 26 C = 79 N = color I = 13;	SU  T = 51 C = 79 N = color I = 13;	SU  T = 4 C = 80 N = color I = 7;	SU  T = 6 C = 80 N = color I = 7;	SU  T = 8 C = 80 N = color I = 7;	SU  T = 10 C = 80 N = color I = 15;	SU  T = 11 C = 80 N = color I = 7;	SU  T = 15 C = 80 N = color I = 15;	SU  T = 21 C = 80 N = color I = 7;	SU  T = 22 C = 80 N = color I = 7;	SU  T = 26 C = 80 N = color I = 7;	SU  T = 51 C = 80 N = color I = 4;	SU  T = 4 C = 81 N = color I = 7;	SU  T = 6 C = 81 N = color I = 7;	SU  T = 8 C = 81 N = color I = 7;	SU  T = 10 C = 81 N = color I = 15;	SU  T = 11 C = 81 N = color I = 7;	SU  T = 15 C = 81 N = color I = 7;	SU  T = 21 C = 81 N = color I = 15;	SU  T = 22 C = 81 N = color I = 7;	SU  T = 26 C = 81 N = color I = 15;	SU  T = 42 C = 81 N = color I = 15;	SU  T = 44 C = 81 N = color I = 15;	SU  T = 47 C = 81 N = color I = 7;	SU  T = 51 C = 81 N = color I = 4;	SU  T = 4 C = 82 N = color I = 7;	SU  T = 6 C = 82 N = color I = 7;	SU  T = 8 C = 82 N = color I = 7;	SU  T = 10 C = 82 N = color I = 7;	SU  T = 11 C = 82 N = color I = 7;	SU  T = 15 C = 82 N = color I = 7;	SU  T = 21 C = 82 N = color I = 7;	SU  T = 22 C = 82 N = color I = 7;	SU  T = 26 C = 82 N = color I = 7;	SU  T = 42 C = 82 N = color I = 15;	SU  T = 44 C = 82 N = color I = 15;	SU  T = 50 C = 82 N = color I = 13;	SU  T = 51 C = 82 N = color I = 4;	SU  T = 4 C = 83 N = color I = 7;	SU  T = 6 C = 83 N = color I = 15;	SU  T = 7 C = 83 N = color I = 15;	SU  T = 8 C = 83 N = color I = 7;	SU  T = 10 C = 83 N = color I = 15;	SU  T = 11 C = 83 N = color I = 15;	SU  T = 15 C = 83 N = color I = 15;	SU  T = 21 C = 83 N = color I = 15;	SU  T = 22 C = 83 N = color I = 15;	SU  T = 26 C = 83 N = color I = 15;	SU  T = 42 C = 83 N = color I = 13;	SU  T = 44 C = 83 N = color I = 13;	SU  T = 46 C = 83 N = color I = 4;	SU  T = 51 C = 83 N = color I = 4;	SU  T = 4 C = 84 N = color I = 7;	SU  T = 6 C = 84 N = color I = 7;	SU  T = 8 C = 84 N = color I = 7;	SU  T = 10 C = 84 N = color I = 7;	SU  T = 11 C = 84 N = color I = 7;	SU  T = 15 C = 84 N = color I = 7;	SU  T = 21 C = 84 N = color I = 7;	SU  T = 22 C = 84 N = color I = 7;	SU  T = 26 C = 84 N = color I = 7;	SU  T = 42 C = 84 N = color I = 15;	SU  T = 44 C = 84 N = color I = 15;	SU  T = 51 C = 84 N = color I = 15;	SU  T = 4 C = 85 N = color I = 7;	SU  T = 6 C = 85 N = color I = 7;	SU  T = 8 C = 85 N = color I = 7;	SU  T = 10 C = 85 N = color I = 7;	SU  T = 11 C = 85 N = color I = 7;	SU  T = 15 C = 85 N = color I = 7;	SU  T = 21 C = 85 N = color I = 7;	SU  T = 22 C = 85 N = color I = 7;	SU  T = 26 C = 85 N = color I = 7;	SU  T = 39 C = 85 N = color I = 4;	SU  T = 42 C = 85 N = color I = 13;	SU  T = 44 C = 85 N = color I = 13;	SU  T = 51 C = 85 N = color I = 13;	SU  T = 10 C = 86 N = color I = 15;	SU  T = 42 C = 86 N = color I = 15;	SU  T = 44 C = 86 N = color I = 15;	SU  T = 51 C = 86 N = color I = 13;	SU  T = 4 C = 87 N = color I = 15;	SU  T = 6 C = 87 N = color I = 7;	SU  T = 8 C = 87 N = color I = 7;	SU  T = 10 C = 87 N = color I = 15;	SU  T = 11 C = 87 N = color I = 7;	SU  T = 15 C = 87 N = color I = 15;	SU  T = 21 C = 87 N = color I = 15;	SU  T = 22 C = 87 N = color I = 7;	SU  T = 26 C = 87 N = color I = 15;	SU  T = 36 C = 87 N = color I = 7;	SU  T = 40 C = 87 N = color I = 7;	SU  T = 41 C = 87 N = color I = 7;	SU  T = 42 C = 87 N = color I = 7;	SU  T = 44 C = 87 N = color I = 7;	SU  T = 47 C = 87 N = color I = 7;	SU  T = 48 C = 87 N = color I = 13;	SU  T = 51 C = 87 N = color I = 4;	SU  T = 53 C = 87 N = color I = 7;	SU  T = 4 C = 88 N = color I = 7;	SU  T = 6 C = 88 N = color I = 7;	SU  T = 8 C = 88 N = color I = 7;	SU  T = 10 C = 88 N = color I = 15;	SU  T = 11 C = 88 N = color I = 7;	SU  T = 15 C = 88 N = color I = 15;	SU  T = 21 C = 88 N = color I = 7;	SU  T = 22 C = 88 N = color I = 7;	SU  T = 24 C = 88 N = color I = 15;	SU  T = 26 C = 88 N = color I = 7;	SU  T = 33 C = 88 N = color I = 13;	SU  T = 36 C = 88 N = color I = 7;	SU  T = 40 C = 88 N = color I = 7;	SU  T = 41 C = 88 N = color I = 7;	SU  T = 42 C = 88 N = color I = 7;	SU  T = 44 C = 88 N = color I = 7;	SU  T = 47 C = 88 N = color I = 7;	SU  T = 48 C = 88 N = color I = 7;	SU  T = 51 C = 88 N = color I = 4;	SU  T = 53 C = 88 N = color I = 13;	SU  T = 4 C = 89 N = color I = 15;	SU  T = 6 C = 89 N = color I = 15;	SU  T = 8 C = 89 N = color I = 15;	SU  T = 10 C = 89 N = color I = 15;	SU  T = 11 C = 89 N = color I = 15;	SU  T = 15 C = 89 N = color I = 15;	SU  T = 21 C = 89 N = color I = 15;	SU  T = 22 C = 89 N = color I = 7;	SU  T = 26 C = 89 N = color I = 7;	SU  T = 36 C = 89 N = color I = 15;	SU  T = 40 C = 89 N = color I = 15;	SU  T = 41 C = 89 N = color I = 7;	SU  T = 42 C = 89 N = color I = 15;	SU  T = 44 C = 89 N = color I = 7;	SU  T = 47 C = 89 N = color I = 7;	SU  T = 48 C = 89 N = color I = 15;	SU  T = 51 C = 89 N = color I = 4;	SU  T = 53 C = 89 N = color I = 7;	SU  T = 4 C = 90 N = color I = 7;	SU  T = 6 C = 90 N = color I = 15;	SU  T = 8 C = 90 N = color I = 15;	SU  T = 10 C = 90 N = color I = 15;	SU  T = 11 C = 90 N = color I = 15;	SU  T = 15 C = 90 N = color I = 7;	SU  T = 21 C = 90 N = color I = 15;	SU  T = 22 C = 90 N = color I = 7;	SU  T = 26 C = 90 N = color I = 7;	SU  T = 36 C = 90 N = color I = 15;	SU  T = 40 C = 90 N = color I = 15;	SU  T = 41 C = 90 N = color I = 15;	SU  T = 42 C = 90 N = color I = 15;	SU  T = 44 C = 90 N = color I = 7;	SU  T = 47 C = 90 N = color I = 15;	SU  T = 48 C = 90 N = color I = 15;	SU  T = 51 C = 90 N = color I = 4;	SU  T = 53 C = 90 N = color I = 15;	SU  T = 4 C = 91 N = color I = 7;	SU  T = 6 C = 91 N = color I = 7;	SU  T = 8 C = 91 N = color I = 7;	SU  T = 10 C = 91 N = color I = 7;	SU  T = 11 C = 91 N = color I = 7;	SU  T = 15 C = 91 N = color I = 7;	SU  T = 21 C = 91 N = color I = 7;	SU  T = 22 C = 91 N = color I = 7;	SU  T = 26 C = 91 N = color I = 7;	SU  T = 36 C = 91 N = color I = 15;	SU  T = 40 C = 91 N = color I = 7;	SU  T = 41 C = 91 N = color I = 7;	SU  T = 42 C = 91 N = color I = 7;	SU  T = 43 C = 91 N = color I = 15;	SU  T = 44 C = 91 N = color I = 7;	SU  T = 46 C = 91 N = color I = 7;	SU  T = 47 C = 91 N = color I = 7;	SU  T = 48 C = 91 N = color I = 15;	SU  T = 51 C = 91 N = color I = 4;	SU  T = 53 C = 91 N = color I = 15;	SU  T = 4 C = 92 N = color I = 7;	SU  T = 6 C = 92 N = color I = 15;	SU  T = 8 C = 92 N = color I = 13;	SU  T = 10 C = 92 N = color I = 7;	SU  T = 11 C = 92 N = color I = 15;	SU  T = 15 C = 92 N = color I = 15;	SU  T = 21 C = 92 N = color I = 15;	SU  T = 22 C = 92 N = color I = 15;	SU  T = 26 C = 92 N = color I = 7;	SU  T = 36 C = 92 N = color I = 7;	SU  T = 40 C = 92 N = color I = 13;	SU  T = 41 C = 92 N = color I = 7;	SU  T = 42 C = 92 N = color I = 7;	SU  T = 43 C = 92 N = color I = 13;	SU  T = 44 C = 92 N = color I = 7;	SU  T = 46 C = 92 N = color I = 7;	SU  T = 47 C = 92 N = color I = 7;	SU  T = 48 C = 92 N = color I = 7;	SU  T = 51 C = 92 N = color I = 4;	SU  T = 52 C = 92 N = color I = 13;	SU  T = 53 C = 92 N = color I = 7;	SU  T = 4 C = 93 N = color I = 7;	SU  T = 6 C = 93 N = color I = 7;	SU  T = 8 C = 93 N = color I = 7;	SU  T = 10 C = 93 N = color I = 7;	SU  T = 11 C = 93 N = color I = 7;	SU  T = 15 C = 93 N = color I = 7;	SU  T = 21 C = 93 N = color I = 7;	SU  T = 22 C = 93 N = color I = 7;	SU  T = 26 C = 93 N = color I = 7;	SU  T = 36 C = 93 N = color I = 15;	SU  T = 40 C = 93 N = color I = 15;	SU  T = 41 C = 93 N = color I = 13;	SU  T = 42 C = 93 N = color I = 15;	SU  T = 44 C = 93 N = color I = 15;	SU  T = 47 C = 93 N = color I = 15;	SU  T = 48 C = 93 N = color I = 13;	SU  T = 51 C = 93 N = color I = 4;	SU  T = 53 C = 93 N = color I = 7;	SU  T = 4 C = 94 N = color I = 7;	SU  T = 6 C = 94 N = color I = 7;	SU  T = 8 C = 94 N = color I = 7;	SU  T = 10 C = 94 N = color I = 7;	SU  T = 11 C = 94 N = color I = 7;	SU  T = 15 C = 94 N = color I = 7;	SU  T = 21 C = 94 N = color I = 7;	SU  T = 22 C = 94 N = color I = 7;	SU  T = 26 C = 94 N = color I = 7;	SU  T = 36 C = 94 N = color I = 7;	SU  T = 40 C = 94 N = color I = 7;	SU  T = 41 C = 94 N = color I = 7;	SU  T = 42 C = 94 N = color I = 7;	SU  T = 43 C = 94 N = color I = 15;	SU  T = 44 C = 94 N = color I = 7;	SU  T = 46 C = 94 N = color I = 7;	SU  T = 47 C = 94 N = color I = 15;	SU  T = 48 C = 94 N = color I = 7;	SU  T = 51 C = 94 N = color I = 4;	SU  T = 53 C = 94 N = color I = 7;	SU  T = 4 C = 95 N = color I = 7;	SU  T = 6 C = 95 N = color I = 7;	SU  T = 8 C = 95 N = color I = 7;	SU  T = 10 C = 95 N = color I = 7;	SU  T = 11 C = 95 N = color I = 7;	SU  T = 15 C = 95 N = color I = 7;	SU  T = 21 C = 95 N = color I = 7;	SU  T = 22 C = 95 N = color I = 7;	SU  T = 26 C = 95 N = color I = 7;	SU  T = 36 C = 95 N = color I = 15;	SU  T = 39 C = 95 N = color I = 13;	SU  T = 40 C = 95 N = color I = 7;	SU  T = 41 C = 95 N = color I = 13;	SU  T = 42 C = 95 N = color I = 13;	SU  T = 44 C = 95 N = color I = 13;	SU  T = 47 C = 95 N = color I = 7;	SU  T = 48 C = 95 N = color I = 13;	SU  T = 51 C = 95 N = color I = 4;	SU  T = 53 C = 95 N = color I = 15;	SU  C = 96 N = color I = 4;	SU  T = 1 C = 96 N = color I = 4;	SU  T = 2 C = 96 N = color I = 4;	SU  T = 3 C = 96 N = color I = 4;	SU  T = 4 C = 96 N = color I = 7;	SU  T = 5 C = 96 N = color I = 4;	SU  T = 6 C = 96 N = color I = 15;	SU  T = 7 C = 96 N = color I = 4;	SU  T = 8 C = 96 N = color I = 7;	SU  T = 9 C = 96 N = color I = 4;	SU  T = 10 C = 96 N = color I = 7;	SU  T = 11 C = 96 N = color I = 7;	SU  T = 12 C = 96 N = color I = 4;	SU  T = 13 C = 96 N = color I = 4;	SU  T = 14 C = 96 N = color I = 4;	SU  T = 15 C = 96 N = color I = 7;	SU  T = 16 C = 96 N = color I = 4;	SU  T = 17 C = 96 N = color I = 4;	SU  T = 18 C = 96 N = color I = 4;	SU  T = 19 C = 96 N = color I = 4;	SU  T = 20 C = 96 N = color I = 4;	SU  T = 21 C = 96 N = color I = 7;	SU  T = 22 C = 96 N = color I = 7;	SU  T = 23 C = 96 N = color I = 4;	SU  T = 24 C = 96 N = color I = 4;	SU  T = 25 C = 96 N = color I = 4;	SU  T = 26 C = 96 N = color I = 7;	SU  T = 27 C = 96 N = color I = 4;	SU  T = 28 C = 96 N = color I = 4;	SU  T = 29 C = 96 N = color I = 4;	SU  T = 30 C = 96 N = color I = 4;	SU  T = 31 C = 96 N = color I = 4;	SU  T = 32 C = 96 N = color I = 4;	SU  T = 33 C = 96 N = color I = 4;	SU  T = 34 C = 96 N = color I = 4;	SU  T = 35 C = 96 N = color I = 4;	SU  T = 36 C = 96 N = color I = 7;	SU  T = 37 C = 96 N = color I = 4;	SU  T = 38 C = 96 N = color I = 4;	SU  T = 39 C = 96 N = color I = 13;	SU  T = 40 C = 96 N = color I = 7;	SU  T = 41 C = 96 N = color I = 7;	SU  T = 42 C = 96 N = color I = 7;	SU  T = 43 C = 96 N = color I = 4;	SU  T = 44 C = 96 N = color I = 7;	SU  T = 45 C = 96 N = color I = 4;	SU  T = 46 C = 96 N = color I = 7;	SU  T = 47 C = 96 N = color I = 7;	SU  T = 48 C = 96 N = color I = 13;	SU  T = 49 C = 96 N = color I = 4;	SU  T = 50 C = 96 N = color I = 4;	SU  T = 51 C = 96 N = color I = 4;	SU  T = 52 C = 96 N = color I = 4;	SU  T = 53 C = 96 N = color I = 7;	SU  T = 4 C = 97 N = color I = 7;	SU  T = 6 C = 97 N = color I = 15;	SU  T = 8 C = 97 N = color I = 7;	SU  T = 10 C = 97 N = color I = 7;	SU  T = 11 C = 97 N = color I = 15;	SU  T = 15 C = 97 N = color I = 7;	SU  T = 21 C = 97 N = color I = 7;	SU  T = 22 C = 97 N = color I = 7;	SU  T = 26 C = 97 N = color I = 15;	SU  T = 36 C = 97 N = color I = 7;	SU  T = 40 C = 97 N = color I = 15;	SU  T = 41 C = 97 N = color I = 15;	SU  T = 42 C = 97 N = color I = 7;	SU  T = 43 C = 97 N = color I = 15;	SU  T = 44 C = 97 N = color I = 7;	SU  T = 46 C = 97 N = color I = 7;	SU  T = 47 C = 97 N = color I = 13;	SU  T = 48 C = 97 N = color I = 15;	SU  T = 51 C = 97 N = color I = 4;	SU  T = 53 C = 97 N = color I = 7;	SU  T = 4 C = 98 N = color I = 7;	SU  T = 6 C = 98 N = color I = 7;	SU  T = 8 C = 98 N = color I = 7;	SU  T = 10 C = 98 N = color I = 7;	SU  T = 11 C = 98 N = color I = 7;	SU  T = 15 C = 98 N = color I = 7;	SU  T = 21 C = 98 N = color I = 7;	SU  T = 22 C = 98 N = color I = 7;	SU  T = 26 C = 98 N = color I = 15;	SU  T = 36 C = 98 N = color I = 15;	SU  T = 40 C = 98 N = color I = 13;	SU  T = 41 C = 98 N = color I = 7;	SU  T = 42 C = 98 N = color I = 7;	SU  T = 43 C = 98 N = color I = 13;	SU  T = 44 C = 98 N = color I = 7;	SU  T = 46 C = 98 N = color I = 7;	SU  T = 47 C = 98 N = color I = 13;	SU  T = 48 C = 98 N = color I = 13;	SU  T = 51 C = 98 N = color I = 4;	SU  T = 53 C = 98 N = color I = 7;	SU  T = 4 C = 99 N = color I = 7;	SU  T = 6 C = 99 N = color I = 7;	SU  T = 8 C = 99 N = color I = 7;	SU  T = 10 C = 99 N = color I = 7;	SU  T = 11 C = 99 N = color I = 7;	SU  T = 15 C = 99 N = color I = 13;	SU  T = 21 C = 99 N = color I = 7;	SU  T = 22 C = 99 N = color I = 7;	SU  T = 26 C = 99 N = color I = 7;	SU  T = 40 C = 99 N = color I = 13;	SU  T = 41 C = 99 N = color I = 15;	SU  T = 51 C = 99 N = color I = 4;	SU  T = 53 C = 99 N = color I = 7;	SU  T = 4 C = 100 N = color I = 7;	SU  T = 6 C = 100 N = color I = 7;	SU  T = 8 C = 100 N = color I = 15;	SU  T = 10 C = 100 N = color I = 7;	SU  T = 11 C = 100 N = color I = 7;	SU  T = 15 C = 100 N = color I = 7;	SU  T = 21 C = 100 N = color I = 7;	SU  T = 22 C = 100 N = color I = 7;	SU  T = 26 C = 100 N = color I = 15;	SU  T = 51 C = 100 N = color I = 4;	SU  C = 101 N = color I = 3;	SU  T = 1 C = 101 N = color I = 3;	SU  T = 2 C = 101 N = color I = 3;	SU  T = 3 C = 101 N = color I = 3;	SU  T = 4 C = 101 N = color I = 3;	SU  T = 5 C = 101 N = color I = 3;	SU  T = 6 C = 101 N = color I = 3;	SU  T = 7 C = 101 N = color I = 3;	SU  T = 8 C = 101 N = color I = 3;	SU  T = 9 C = 101 N = color I = 3;	SU  T = 10 C = 101 N = color I = 3;	SU  T = 11 C = 101 N = color I = 3;	SU  T = 12 C = 101 N = color I = 3;	SU  T = 13 C = 101 N = color I = 3;	SU  T = 14 C = 101 N = color I = 3;	SU  T = 15 C = 101 N = color I = 3;	SU  T = 16 C = 101 N = color I = 3;	SU  T = 17 C = 101 N = color I = 3;	SU  T = 18 C = 101 N = color I = 3;	SU  T = 19 C = 101 N = color I = 3;	SU  T = 20 C = 101 N = color I = 3;	SU  T = 21 C = 101 N = color I = 3;	SU  T = 22 C = 101 N = color I = 3;	SU  T = 23 C = 101 N = color I = 3;	SU  T = 24 C = 101 N = color I = 3;	SU  T = 25 C = 101 N = color I = 3;	SU  T = 26 C = 101 N = color I = 3;	SU  T = 27 C = 101 N = color I = 3;	SU  T = 28 C = 101 N = color I = 3;	SU  T = 29 C = 101 N = color I = 3;	SU  T = 30 C = 101 N = color I = 3;	SU  T = 31 C = 101 N = color I = 3;	SU  T = 32 C = 101 N = color I = 3;	SU  T = 33 C = 101 N = color I = 3;	SU  T = 34 C = 101 N = color I = 3;	SU  T = 35 C = 101 N = color I = 3;	SU  T = 36 C = 101 N = color I = 3;	SU  T = 37 C = 101 N = color I = 3;	SU  T = 38 C = 101 N = color I = 3;	SU  T = 39 C = 101 N = color I = 3;	SU  T = 40 C = 101 N = color I = 3;	SU  T = 41 C = 101 N = color I = 3;	SU  T = 42 C = 101 N = color I = 3;	SU  T = 43 C = 101 N = color I = 3;	SU  T = 44 C = 101 N = color I = 3;	SU  T = 45 C = 101 N = color I = 3;	SU  T = 46 C = 101 N = color I = 3;	SU  T = 47 C = 101 N = color I = 3;	SU  T = 48 C = 101 N = color I = 3;	SU  T = 49 C = 101 N = color I = 3;	SU  T = 50 C = 101 N = color I = 3;	SU  T = 51 C = 101 N = color I = 3;	SU  T = 52 C = 101 N = color I = 3;	SU  T = 53 C = 101 N = color I = 3;	SU  C = 102 N = color I = 3;	SU  T = 1 C = 102 N = color I = 3;	SU  T = 2 C = 102 N = color I = 3;	SU  T = 3 C = 102 N = color I = 3;	SU  T = 4 C = 102 N = color I = 3;	SU  T = 5 C = 102 N = color I = 3;	SU  T = 6 C = 102 N = color I = 3;	SU  T = 7 C = 102 N = color I = 3;	SU  T = 8 C = 102 N = color I = 3;	SU  T = 9 C = 102 N = color I = 3;	SU  T = 10 C = 102 N = color I = 3;	SU  T = 11 C = 102 N = color I = 3;	SU  T = 12 C = 102 N = color I = 3;	SU  T = 13 C = 102 N = color I = 3;	SU  T = 14 C = 102 N = color I = 3;	SU  T = 15 C = 102 N = color I = 3;	SU  T = 16 C = 102 N = color I = 3;	SU  T = 17 C = 102 N = color I = 3;	SU  T = 18 C = 102 N = color I = 3;	SU  T = 19 C = 102 N = color I = 3;	SU  T = 20 C = 102 N = color I = 3;	SU  T = 21 C = 102 N = color I = 3;	SU  T = 22 C = 102 N = color I = 3;	SU  T = 23 C = 102 N = color I = 3;	SU  T = 24 C = 102 N = color I = 3;	SU  T = 25 C = 102 N = color I = 3;	SU  T = 26 C = 102 N = color I = 3;	SU  T = 27 C = 102 N = color I = 3;	SU  T = 28 C = 102 N = color I = 3;	SU  T = 29 C = 102 N = color I = 3;	SU  T = 30 C = 102 N = color I = 3;	SU  T = 31 C = 102 N = color I = 3;	SU  T = 32 C = 102 N = color I = 3;	SU  T = 33 C = 102 N = color I = 3;	SU  T = 34 C = 102 N = color I = 3;	SU  T = 35 C = 102 N = color I = 3;	SU  T = 36 C = 102 N = color I = 3;	SU  T = 37 C = 102 N = color I = 3;	SU  T = 38 C = 102 N = color I = 3;	SU  T = 39 C = 102 N = color I = 3;	SU  T = 40 C = 102 N = color I = 3;	SU  T = 41 C = 102 N = color I = 3;	SU  T = 42 C = 102 N = color I = 3;	SU  T = 43 C = 102 N = color I = 3;	SU  T = 44 C = 102 N = color I = 3;	SU  T = 45 C = 102 N = color I = 3;	SU  T = 46 C = 102 N = color I = 3;	SU  T = 47 C = 102 N = color I = 3;	SU  T = 48 C = 102 N = color I = 3;	SU  T = 49 C = 102 N = color I = 3;	SU  T = 50 C = 102 N = color I = 3;	SU  T = 51 C = 102 N = color I = 3;	SU  T = 52 C = 102 N = color I = 3;	SU  T = 53 C = 102 N = color I = 3;	SU  C = 103 N = color I = 3;	SU  T = 1 C = 103 N = color I = 3;	SU  T = 2 C = 103 N = color I = 3;	SU  T = 3 C = 103 N = color I = 3;	SU  T = 4 C = 103 N = color I = 3;	SU  T = 5 C = 103 N = color I = 3;	SU  T = 6 C = 103 N = color I = 3;	SU  T = 7 C = 103 N = color I = 3;	SU  T = 8 C = 103 N = color I = 3;	SU  T = 9 C = 103 N = color I = 3;	SU  T = 10 C = 103 N = color I = 3;	SU  T = 11 C = 103 N = color I = 3;	SU  T = 12 C = 103 N = color I = 3;	SU  T = 13 C = 103 N = color I = 3;	SU  T = 14 C = 103 N = color I = 3;	SU  T = 15 C = 103 N = color I = 3;	SU  T = 16 C = 103 N = color I = 3;	SU  T = 17 C = 103 N = color I = 3;	SU  T = 18 C = 103 N = color I = 3;	SU  T = 19 C = 103 N = color I = 3;	SU  T = 20 C = 103 N = color I = 3;	SU  T = 21 C = 103 N = color I = 3;	SU  T = 22 C = 103 N = color I = 3;	SU  T = 23 C = 103 N = color I = 3;	SU  T = 24 C = 103 N = color I = 3;	SU  T = 25 C = 103 N = color I = 3;	SU  T = 26 C = 103 N = color I = 3;	SU  T = 27 C = 103 N = color I = 3;	SU  T = 28 C = 103 N = color I = 3;	SU  T = 29 C = 103 N = color I = 3;	SU  T = 30 C = 103 N = color I = 3;	SU  T = 31 C = 103 N = color I = 3;	SU  T = 32 C = 103 N = color I = 3;	SU  T = 33 C = 103 N = color I = 3;	SU  T = 34 C = 103 N = color I = 3;	SU  T = 35 C = 103 N = color I = 3;	SU  T = 36 C = 103 N = color I = 3;	SU  T = 37 C = 103 N = color I = 3;	SU  T = 38 C = 103 N = color I = 3;	SU  T = 39 C = 103 N = color I = 3;	SU  T = 40 C = 103 N = color I = 3;	SU  T = 41 C = 103 N = color I = 3;	SU  T = 42 C = 103 N = color I = 3;	SU  T = 43 C = 103 N = color I = 3;	SU  T = 44 C = 103 N = color I = 3;	SU  T = 45 C = 103 N = color I = 3;	SU  T = 46 C = 103 N = color I = 3;	SU  T = 47 C = 103 N = color I = 3;	SU  T = 48 C = 103 N = color I = 3;	SU  T = 49 C = 103 N = color I = 3;	SU  T = 50 C = 103 N = color I = 3;	SU  T = 51 C = 103 N = color I = 3;	SU  T = 52 C = 103 N = color I = 3;	SU  T = 53 C = 103 N = color I = 3;END;Begin MESQUITE;		MESQUITESCRIPTVERSION 2;		TITLE AUTO;		tell ProjectCoordinator;		timeSaved 1697353696123;		getEmployee #mesquite.minimal.ManageTaxa.ManageTaxa;		tell It;			setID 0 2509909606420158246;			tell It;				setDefaultOrder  1 0 2 24 7 22 16 19 3 26 23 17 4 11 25 10 5 6 15 8 27 20 18 9 14 21 13 12 28 52 38 48 36 53 41 44 31 49 32 47 30 33 35 34 54 43 46 37 40 42 39 45 29;				attachments ;			endTell;		endTell;		getEmployee #mesquite.charMatrices.ManageCharacters.ManageCharacters;		tell It;			setID 0 3267967362074950588;			tell It;				setDefaultOrder  0 2 1 4 3 128 8 7 11 13 14 15 17 19 20 21 26 24 27 25 22 33 34 35 36 129 40 38 43 44 45 47 48 49 51 52 53 54 56 57 58 59 61 63 64 65 66 67 68 69 70 71 72 73 42 41 75 76 77 78 79 80 81 82 84 85 86 87 88 89 90 91 92 93 95 96 98 99 100 101 103 104 105 106 107 108 109 111 112 113 114 115 116 117 118 119 121 122 123 125 124 127 126;				attachments ;			endTell;			mqVersion 370;			checksumv 0 3 1428219467 null  getNumChars 103 numChars 103 getNumTaxa 53 numTaxa 53   short true   bits 15   states 15   sumSquaresStatesOnly 11157.0 sumSquares 11157.0 longCompressibleToShort false usingShortMatrix true   NumFiles 1 NumMatrices 1;			mqVersion;		endTell;		getWindow;		tell It;			suppress;			setResourcesState false true 70;			setPopoutState 300;			setExplanationSize 0;			setAnnotationSize 0;			setFontIncAnnot 0;			setFontIncExp 0;			setSize 1503 1086;			setLocation 423 25;			setFont SanSerif;			setFontSize 10;			getToolPalette;			tell It;			endTell;			desuppress;		endTell;		getEmployee  #mesquite.minimal.ManageTaxa.ManageTaxa;		tell It;			showTaxa #2509909606420158246 #mesquite.lists.TaxonList.TaxonList;			tell It;				setTaxa #2509909606420158246;				getWindow;				tell It;					useTargetValue off;					setTargetValue ;					newAssistant  #mesquite.lists.TaxonListCurrPartition.TaxonListCurrPartition;					setExplanationSize 30;					setAnnotationSize 20;					setFontIncAnnot 0;					setFontIncExp 0;					setSize 1503 1014;					setLocation 423 25;					setFont SanSerif;					setFontSize 10;					getToolPalette;					tell It;						setTool mesquite.lists.TaxonList.TaxonListWindow.ibeam;					endTell;					setActive;				endTell;				showWindow;				getEmployee #mesquite.lists.ColorTaxon.ColorTaxon;				tell It;					setColor Dark Gray;					removeColor on;				endTell;				getEmployee #mesquite.lists.TaxonListAnnotPanel.TaxonListAnnotPanel;				tell It;					togglePanel off;				endTell;			endTell;		endTell;		getEmployee  #mesquite.charMatrices.BasicDataWindowCoord.BasicDataWindowCoord;		tell It;			showDataWindow #3267967362074950588 #mesquite.charMatrices.BasicDataWindowMaker.BasicDataWindowMaker;			tell It;				getWindow;				tell It;					getTable;					tell It;						rowNamesWidth 110;					endTell;					setExplanationSize 30;					setAnnotationSize 20;					setFontIncAnnot 0;					setFontIncExp 0;					setSize 1503 1014;					setLocation 423 25;					setFont SanSerif;					setFontSize 10;					getToolPalette;					tell It;						setTool mesquite.charMatrices.BasicDataWindowMaker.BasicDataWindow.ibeam;					endTell;					setTool mesquite.charMatrices.BasicDataWindowMaker.BasicDataWindow.ibeam;					colorCells  #mesquite.charMatrices.ColorByState.ColorByState;				tell It;					setStateLimit 9;					toggleUniformMaximum on;				endTell;					colorRowNames  #mesquite.charMatrices.TaxonGroupColor.TaxonGroupColor;					colorColumnNames  #mesquite.charMatrices.CharGroupColor.CharGroupColor;					colorText  #mesquite.charMatrices.NoColor.NoColor;					setBackground White;					toggleShowNames off;					toggleShowTaxonNames on;					toggleTight off;					toggleThinRows off;					toggleShowChanges on;					toggleSeparateLines off;					toggleShowStates on;					toggleReduceCellBorders off;					toggleAutoWCharNames on;					toggleAutoTaxonNames off;					toggleShowDefaultCharNames off;					toggleConstrainCW on;					toggleBirdsEye off;					toggleColorOnlyTaxonNames off;					toggleShowPaleGrid off;					toggleShowPaleCellColors off;					toggleShowPaleExcluded off;					togglePaleInapplicable on;					togglePaleMissing off;					toggleShowBoldCellText off;					toggleAllowAutosize on;					toggleColorsPanel on;					toggleDiagonal on;					setDiagonalHeight 80;					toggleLinkedScrolling on;					toggleScrollLinkedTables off;					getInfoPanel;					tell It;						btspOpen true;						apOpen true;						fpOpen true;					endTell;					toggleInfoPanel off;				endTell;				showWindow;				getWindow;				tell It;					forceAutosize;				endTell;				getEmployee #mesquite.charMatrices.AlterData.AlterData;				tell It;					toggleBySubmenus off;				endTell;				getEmployee #mesquite.charMatrices.ColorCells.ColorCells;				tell It;					setColor Yellow;					removeColor off;				endTell;				getEmployee #mesquite.categ.StateNamesStrip.StateNamesStrip;				tell It;					showStrip off;				endTell;				getEmployee #mesquite.charMatrices.AnnotPanel.AnnotPanel;				tell It;					togglePanel off;				endTell;				getEmployee #mesquite.charMatrices.CharReferenceStrip.CharReferenceStrip;				tell It;					showStrip off;				endTell;				getEmployee #mesquite.charMatrices.QuickKeySelector.QuickKeySelector;				tell It;					autotabOff;				endTell;				getEmployee #mesquite.charMatrices.SelSummaryStrip.SelSummaryStrip;				tell It;					showStrip off;				endTell;				getEmployee #mesquite.categ.SmallStateNamesEditor.SmallStateNamesEditor;				tell It;					panelOpen true;				endTell;			endTell;		endTell;		getEmployee  #mesquite.charMatrices.ManageCharacters.ManageCharacters;		tell It;			showCharacters #3267967362074950588 #mesquite.lists.CharacterList.CharacterList;			tell It;				setData 0;				getWindow;				tell It;					useTargetValue off;					setTargetValue ;					newAssistant  #mesquite.lists.DefaultCharOrder.DefaultCharOrder;					newAssistant  #mesquite.lists.CharListInclusion.CharListInclusion;					newAssistant  #mesquite.lists.CharListPartition.CharListPartition;					newAssistant  #mesquite.parsimony.CharListParsModels.CharListParsModels;					newAssistant  #mesquite.lists.CharacterStats.CharacterStats;				tell It;					toggleSelectedOnly off;				endTell;					setExplanationSize 30;					setAnnotationSize 20;					setFontIncAnnot 0;					setFontIncExp 0;					setSize 1503 1014;					setLocation 423 25;					setFont SanSerif;					setFontSize 10;					getToolPalette;					tell It;						setTool mesquite.lists.CharacterList.CharacterListWindow.ibeam;					endTell;				endTell;				showWindow;				getEmployee #mesquite.lists.CharListAnnotPanel.CharListAnnotPanel;				tell It;					togglePanel off;				endTell;			endTell;		endTell;		endTell;end;
