# Supplementary material for: Dynamic interplay between niche variation and flight adaptability drove a hundred million years’ dispersion in iconic lacewings
Source: Proc Natl Acad Sci U S A. 2025 May 2;122(19):e2414549122. doi: 10.1073/pnas.2414549122 (PMC12087969; doi:10.1073/pnas.2414549122)
Supplement: Supplementary file 8 — Dataset S07 (RTF) [file pnas.2414549122.sd07.rtf]

#NEXUSbegin data;dimensions ntax=53 nchar=6013;format datatype = mixed( standard: 1-103, dna: 104-6013) gap = - missing =?;matrixMucroberotha   0010100011011101(0 1)0(0 1)000002001000011001000101001011000111100011200000002100000001111002000001011011102111CAGCCATTTTACCAGTACGCGACAATGATTATTCTCAACTAACCATAAAGATATTGGAG-TATTATATTTTATTTTTGGAATTTGATCTGGATTAGTAGGTACAAGTTTAAGATTATTAATTCGAGCAGAATTAGGTCAACCAGGTTCATTAATTGGAGATGATCAAATTTACAAT-GTTATTGTAACTGCACATGCATTTATCATAATTTTTTTTATAGTTATACCAATCATAATTGGAGGATTCGGAAATTGACTAGTGCCTTTAATATTAGCAGCACCAGATATAGCTTTTCCACGTATAAACAATATAAGTTTTTGACTTTTACCACCATCTTTAACTTTATTATTAGCTTCATCTTTAGTAGAAAGAGGAGCTGGAACAGGATGAACAGTTTATCCTCCTTTAGCTTCAGGAATTGCTCATGCTGGAGCTTCTGTAGATTTAGCTATTTTTAGTTTACACCTTGCAGGAGTTTCTTCAATTTTAGGGGCAGTAAATTTCATTACTACAGTAATTAATATACGATTAAATTATATAACTTTAGACCGTATACCTTTATTTTGTTGAGCTGTTGTAATTACAGCTTTACTTTTATTATTATCATTACCAGTATTAGCTGGAGCTATTACAATATTATTAACTGATCGTAATTTAAATACATCTTTCTTTGATCCGGCAGGAGGAGGAGACCCAATCTTATATCAACATTTATTTTGATGTTTTGGTCACCCTGAAGTTTATATATT--TCGCCTGTTTATCAAAAACATGTCTTTTTGAA-TATAATATAAAGTCTAACCTGCCCACTGAAAAT---TTTAAAGGGCCGCGGTATATTGACTGTGCAAAGGTAGCATAATCATTTGTCTTTTAATTGAAGGCTAGAATGAATGGTTGAATGAAGTATTAACTGTCTCATTTTAATAAATTTAAAAATTTAATTTTTTAGTCAAAAAGCTAAAATTTATTTAAAAGACGAGAAGACCCTATAGATCTTTATAAAT-TTTTTTATAAAAATATTTAGAATAAAAAAT-TATTTTTATATAATTA--TTTATTTTATTGGGGTGATAGGAAAATTTATTTAACTTTTCATA--ATTTAAT-CCATTGATTTATGATTTATTGATCCAATTTTATTGATTATAAATTTAAGTTACCTTAGGGATAACAGCGTA-ATTTTTTTAGAGAGTTCTTATCGATAAAAAAGATTGCGACCTCGATGTTGAATTAAAGGTTATTTTTAGATGTAGAAGTTTAA-AGTTTAGGTCTGTTCGACCTTTGAATCT------------------------------TCTGGTTGATCCTGCCAGTAGTCATATGCTTGTCTCAAAGATTAAGCCATGCATGTCTCAGTACATACCAAAATAAGGTGAAACCGCGAAAGGCTCATTATATCAGTTATGGTTCCTTAGATCGTACACACATTTACTTGGATAACTGTGGT-----AATTCTAGAGCTAATACATG---CAAACAG--AGTCC-CGACCAGAGATGGAAGGGATGCTTTTATTAGATCAAAACCAATCGGTGTT---------------------------GCATTTTATTT---ATTTACATAGATA----------------------------------------------------------TTATTGTA------------GCATCGTTTCTTTTGGTGACTCTGAATAACTTTAAGCTGATCGCACGGTCTC------GTACCGGCGACGCATCTTTCAAAT---------GTCTGCCTTATCAACTTTCGATGGTAGGTTCTGCGCCTACCATGG-----TTGTAACGGGTAACGGGGAATCAGGGTTCG-------ATTCCGGAGAGGGAGCCTGAGAAACGGCTACCACATCCAA------GGAAGGCAGCAGGCGCGCAAATTACCCACTCCCGGCACGGGGAGGTA--------GTGACG--AAAAATAACGATACGGGACTCATCCGAGGCCCCGTAATCGGAATGAGTACACTTTAAATCCTTTAACA-ATTAACAATTGGAG--GGCAAGTCTGGTGCCAGCAG---CCGCGGTAATTCCAGCTCCAATAGCGTATATTAAAGTTGTTGCGGTTAAAAAGCTCGTAGTCGAATTTGTGCCCCACACTGTCGGTTCACCGCTCGCGGTGTTCAACTGGCATGTTAT-----------------------------------------------------------------------------------------------------------------------------------------------------------------------------------------------------------------------------------------------------------------------------------------------------------------------------------------------------------------------------------------------------------------------------------------------------------------------------------------------------------------------------------------------------------------------------------------------------------------------------------------------------------------------------------------------------------------------------------------------------------------------------------------------------------------------------------------------------------------------------------------------------------------------------------------------------------------------------------------------------------------------------------------------------------------------------------------------------------------------------------------------------------------------------------------------------------------------------------------------------------------------------------------------------------------------------------------------------------------------------------------------------------------------------------------------------------------------------------------------------------------------------GGAACAAATCGCTGCTATATGACGTCACAAAATCATGGATACGCCGTAAGTACTGAATCATTTCCAAACGAATGGGAAGCCCTATTTACAAATGCTAATGACAAAACTAATGAAGGAACTATCCATAAAACATTACCATACTTCAGTGTACAGTTTCATCCAGAACATACAGCTGGACCCGAAGACCTGGAAAATCTTTTTGATGTATTTCTTGATTCAGTTAAAAATAATATTGAAAATAAATTGTCAAATATTAAAGAAATTTTAACAGATCGTTTAACATATAAGC---CAAAACATTATTATTCAGTTACTCATCCGAAAAAGGTTCTTATAATTGGATCTGGCGGCTTATCGATTGGTCAAGCTGGTGAATTTGATTATTCTGGATCGCAAGCAATAAAAGCTCTGAAAGAGGAAAATATTCAAACAGTTTTAATAAATCCAAATATCGCCACAGTACAAACATCTAAAGGATTAGCAGACAAGGTTTATTTTCTACCTTTAGTACCCGAATATGTTGAGCAAGTAATTCGTTCAGAACGTCCCAATGGTGTTTTGCTAACTTTCGGTGGACAAACAGCATTAAATTGTGGTGTGGAATTAGAAAAAGCGAATGTTTTTGAAAAATATGGCTGTAAAATTTTAGGCACACCAATTCAATCTATAATTGAAACAGAAGATCGAAAAATATTTGCTGAAAAAATTCATGAGATAGGTGAAAAAGTTGCCCCCAGTGCTGCTGTGCATTCGGTGCAGGAAGCCTTAGAAGCTGCTGAAAATATTGGATATCCTATTTTATGTCGAGCGGCTTTTTCACTTGGTGGTTTAGGTTCTGGATTTGCACATAATAAAGAGCAATTAGTCAAGCTAGCAACGCAAGCTTTTGCTAATTCAAATCAGTTGATTATTGACAAATCTTTAAAAGGATGGAAGGAAGTTGAGTATGAAGTTGTTCGCGATGCTTTTGATAATTGTATAACCGTTTGTAATATGGAAAATGTTGATCCACTTGGAATACATACGGGAGAGTCTATTGTCGTTGCACCGAGTCAAACTTTATCAAATCGTGAATACAATATGCTTAGAACAACAGCCATAAGTGTAATCCGCCATTTTGGTGTTGTAGGTGAATGTAACATTCAGTACGCATTGAGTCCAAATTCCGAAGAATATTATATAATCGAAGTCAATGCTAGATTATCACGCAGTTCTGCATTAGCTAGTAAAGCTACCGGATACCCTCTAGCGTATGTCGCAGCAAAATTAGCCCTTGGTACATCTTTACTCGATATTAAAAATTCAGTTACTGGTGAAACAACTGCCTGTTTTGAACCAAGTTTAGATTATTGTGTTGTGAAAATTCCTCGCTGGGATCTGAGTAAATTTAATCGTGTTAGCTCGAAAATAGGCAGTTCAA-TGAAAAGTGTGGGTGAAGTTATGGCCGTTGGTAGAAAGTTTGAAGAAGCATTTCAAAAAGCGTTACGTATGGTTGATGAAAATGTAATGGGTT-------TTGATCCAAATTTGCAGAAAATTAGTGATGAAGATTTAAAAGAACCAACTGAC----AAACGCATGTTTGTTATTGCGGCATCGCTTATGGCTGGTTATTCAATTGAGAAACTTAACAATTTAACTAAAATTGATCCTTGGTTTTTGCAGAAAATGAAGAATATTATTGATATTCATTGTAAATTAACAACACT----CACTCAACATGACG---TTTCGCATGAACATTTGCGTGAAGCAAAACGTTACGGATTTTCAGACAAACAAATTGCAAGTGCAATTCAAAGTACCGAATTAGCCGTACGAAAGCAGAGACAAGAATGTGGAATTTTGCCGTTTGTAAAGCAAATTGATACTGTTGCCGCCGAGTGGCCAGCATGTACTAATTACTTATATCTTACGTA------TAATG-CTCTCTCACACGATTTAGAATTTCCTGGAGGATATACAATGGTTATCGGGTCGGGT--GTATATCGAATCGGTAGTTCAGTTGAATTTGA--TTGGTGT-----GCTGTCG-GTTGTTTAAGAGAGTTACGAAAATTAGGCAAG--------AAAACAGTTATGGTTAACTACAACCCGGAAACCGTCAGTACAGATTATGATATGTGCGACCGTT-TATATTTCGAAGAAATTTCTTTCGAGGTAGT-TATGG-ATATTTATAATGCTGAAAATCCTGAAGGTATC-Nallachius   01(0 1)0000000000000010001100000000011001010011000100100100001010101001000200000000001010000000000000001000--TCAGCCATTTTACCATGCAACGTTGACTTTTTTCAACTAATCATAAAGATATTGGAA-CTTTATACTTCATCTTTGGAATTTGAGCAGGGCTTGTAGGTATAAATCTAAGTATAATCATTCGAGCAGAACTTGCACATCCAGGAGCTTTAATTGGGGATGACCAAATTTATAAT-GTCATTGTTACAGCTCATGCCTTTATCATAATTTTCTTTATAGTTATACCTATTGTTATTGGAGGGTTTGGAAATTGACTTGTTCCATTAATACTTGCTGCACCTGATATAGCTTTCCCTCGAATAAATAATATAAGTTTTTGACTTCTGCCACCATCATTAACCTTACTTTTGGCCTCAGGAATAGTAGAAAGAGGAGCTGGAACAGGATGAACTGTGTATCCTCCTTTAGCCTCTGCTGTTGCCCATGCTGGAGCATCAGTTGACCTAACAATTTTCAGATTACACCTTGCTGGGGTATCTTCAATTCTTGGAGCTGTAAATTTTATTACCACTGTAATTAATATACGTCTTCCATCAATAACCTTAGACCGAATGCCCCTATTTGTTTGATCTGTAGTAATTACAGCAATTTTATTACTCTTATCCCTACCTGTTCTTGCAGGTGCAATTACTATACTCCTTACTGACCGTAACTTAAATACATCATTCTTTGACCCAGCAGGAGGTGGAGATCCCATCCTATACCAACATCTATTTTGATTTTTTGGTCATCCTGAAGTTAT----------------------------CAGGCTTGAAGAGTATATTTTTAAGTCTAATCTGCCCACTGAATTTA--TTTAAAGGGCCGCAGTATTTTGACTGTGCAAAGGTAGCATAATTATTTGTTTCTTAATTAGGGACTAGTATGAATGATTGAATGAAATTAGTACTGTCTCATTATAAGTGATTATAGAATTTAATTTTTAAGTAAAAAAGCTTAAATTAAATTAAAAGACGAGAAGACCCTATAGAGTTTAATATTTAAAAATAATA---TTATATATGGGGTAATATAGATAGTTTTATTAAAA---ATATTTAATTGGGGTGATTAGAAAATTAAATTAACTTTTCTTT--AGTTTAA--TATTG-TTAATAGATATATGATCCTTTATTATAGATTAAAAGATTAAATTACCTTAGGGATAACAGCGTACATTTATTTGGAGAGTTCATATTGAAAAAAAAGTTTGCGACCTCGATGTTGAAATAAAATATAATT-TGAGTGCAGAAGCTC-------------------------------------------------------------TCTGGTTGATCCTGCCAGTAGTCATATGCTTGTCTCAAAGATTAAGCCATGCATGTCTCAGTACAAGCCATATTAAGGTGAAACCGCGAAAGGCTCATTATATCAGTTATGGTTCCTTAGATCGTACCCACATTTACTTGGATAACTGTGGT-----AATTCTAGAGCTAATACATG---CAAACAG--AGTTC-TGACCAGAGATGGAAGGAATGCTTTTATTAGATCAAAACCAATCGGTATTTATTATATGGGTTTATATTTTTATTCGATATTTTGATTTATATTTATATTGATAACTTTTGTTTGATATATTTAAATATAATGATAATTGAGAATTGAATATATATTACTCATTAATATA----TTATATATGTACCGTATTATTTGGTGACTCTGAATAACTTTTAGCTGATCGCACGGTCTTTATTTAGTACCGGCGACGTATCTTTCAAAT---------GTCTGCCTTATCAACTGTCGATGGTAGGTTCTGTGCCTACCATGG-----TTGTAACGGGTAACGGGGAATCAGGGTTCG-------ATTCCGGAGAGGGAGCCTGAGAAACGGCTACCACATCCAA------GGAAGGCAGCAGGCGCGCAAATTACCCACTCCCAGCACGGGGAGGTA--------GTGACG--AAAAATAACGATACGGGACTCATCCGAGGCCCCGTAATCGGAATGAGTACACTTTAAATCCTTTAACG-AGGATCCATTGGAG--GGCAAGTCTGGTGCCAGCGG---CCGCGGTAATT-------------------------------------------------------------------------------------------------------------------------------------------------------------------------------------------------------------------------------------------------------------------------------------------------------------------------------------------------------------------------------------------------------------------------------------------------------------------------------------------------------------------------------------------------------------------------------------------------------------------------------------------------------------------------------------------------------------------------------------------------------------------------------------------------------------------------------------------------------------------------------------------------------------------------------------------------------------------------------------------------------------------------------------------------------------------------------------------------------------------------------------------------------------------------------------------------------------------------------------------------------------------------------------------------------------------------------------------------------------------------------------------------------------------------------------------------------------------------------------------------------------------------------------------------------------------------------------------------------------------------------------------ACAAATCGTTGCTTTATGACATCACAAAATCATGGATATGCCGTTGATTCAAAAGCACCACCTACACAGTGGCGTTCACTATTTATAAATGCAAATGATCAAAGTAACGAAGGAATTCAACATTCAACGTTGCCATATTTTAGTGTACAATTTCATCCGGAACATACAGCTGGACCAGAAGATTTAGAGTGTCTCTTCGATGTGTTCCTTGACACAGTAAAGCAATATAAAGCAAATGTCAAAGTAAAACTAAATGATATTTTAAACAAATGTTTACAATATGTAC---CTAAGGAGATTTACAACACAAACATTCCAAAGAAAGTGTTGATTATCGGCTCTGGGGGATTGTCAATTGGACAAGCTGGTGAATTCGATTACTCCGGTTCACAAGCAATAAAAGCATTACGTGAGGAGGGTATACAAACAATTTTAATTAATCCAAATATTGCAACAGTGCAAACATCAAAAGGACTAGCTGACAAAGTGTATTATTTACCACTACTACCGGAGTATATTGAACAGGTGATTAAATCCGAGCGTCCTGGCGGTGTTTTGCTTACATTCGGCGGACAAACAGCACTGAATTGTGGTGTACAATTAGAAAAGGCGAATGTATTTAAAAAGTATGGATGCCGAATATTAGGCACACCAATTGAGTCCATTATCGAAACAGAGGATCGTAAAATGTTTGCAGAGAAAATTAGTGAAATTAATGAACGTGTAGCACCTAGTGAGGCTGTGTATTCGATTGAAGAAGCATTGAATGCAGCTGATAAAATTGGATATCCAGTTATGACACGTGCTGCATTTTCACTAGGTGGTTTAGGTTCCGGTTTTGCTAACAATAGTGACGAATTACGATTGCTAGCGGCTCAAGCATTAGCACATTCATCACAAGTTATTATTGATAAATCGTTAAAAGGTTGGAAGGAGGTGGAATATGAGGTAGTTCGTGATGCGTATGATAACTGTATAACGGTATGTAATATGGAGAATCTTGATCCATTAGGAATACATACCGGTGAATCAATAGTAGTAGCACCAAGTCAAACATTATCAAATCGCGAATATAATTTATTGAGAAGTACAGCCATAAAAGTAATTCGTCATTTTGGTATTGTCGGTGAATGCAACATACAATATGCATTCAATCCATATAGTGAAGAATATTATATCATTGAAGTGAATGCACGTTTATCGCGAAGTTCAGCACTTGCTAGCAAAGCAACTGGTTATCCATTAGCGTATGTAGCTGCTAAATTAGCACTAGGTACATCGTTGTTAAATATAAAGAATACGGTAACTGGTGAAACAACAGCATGCTTCGAGCCTAGTTTAGATTATTGTGTGGTAAAAATACCACGGTGGGATTTAAGTAAATTTTTACGAGTTAGCACTAAAATTGGCAGTTCAA-TGAAAAGTGTCGGTGAAGTGATGGCTATCGGTAGAAAATTCGAGGAGGCATTTCAAAAAGCATTACGTATGGTTGATGAAAATGTAAAAGGTT-------TTGATCCCAACATACAAAAAATCAACGATGACAATTTAACAGAGCCTACCGAC----AAACGTATGTTTGTAATAGCGGCATCATTAAAAGCTGGTTACACTGTGGATCGTTTGCATGATCTCACAAAAATTGATCGTTGGTTTTTGCAAAAAATGAAAAATATCATTGAC-TTCAATAAATATTTAGAGGATTTGAGCACTAAACCGGATACAATAACACGTGACATTTTATTGCATGCAAAACGTTATGGATTTTCAGATAAACAAATTGCTGCATCAATTAGAAGTACCGAACTAGCGGTGCGGAAACAACGAGAAGAGCACAATATATTACCGTTTGTAAAACAAATTGATACAGTTGCTGCTGAATGGCCAGCGAGCACAAATTATCTATATCTCACATA------CAACG-CTTCATCACACGACTTAGAATTTCCAGGCAATCACATTATGGTAATTGGTTCAGGT--GTTTATCGTATTGGTAGTTCTGTTGAATTTGA--TTGGTGT-----GCAGTAG-GATGTTTACGCGAATTACGGAAATTGAATAAA--------AAAACAATTATGGTCAATTACAATCCAGAAACAGTGAGCACCGATTATGATATGTGCGATCGTT-TATATTTTGAAGAGATATCATTTGAAGTCGT-TATGG-ATATATATAATATAGTGAATCCGGAT-------Plega   0010001111111110101100112010110100010111101001011010010110200000101000010100000000002000011000000002011---------------------------------------------------------AG-TTTTATATTTTATTTTTGGAATTTGATCAGGACTTGTAGGAACTAGTTTAAGTTTATTAATTCGTGCAGAATTAGGTCAACCAGGTTCATTAATTGGAGATGATCAAATTTATAAT-GTTATCGTTACAGCCCATGCTTTTATTATAATTTTTTTTATAGTTATACCTATTATAATTGGAGGATTTGGAAATTGATTAGTACCTTTAATATTAGCAGCTCCAGATATAGCATTTCCTCGAATAAATAATATAAGTTTTTGATTTTTACCTCCATCTTTAACATTACTTTTAGCATCATCAATAGTTGAAAGAGGAGCTGGTACCGGATGAACAGTTTATCCTCCATTAGCTGCTAGAATTGCTCATGCAGGAGCATCAGTAGATTTAGCAATTTTTAGTTTACATTTAGCCGGTATTTCATCTATTTTAGGAGCAGTAAATTTTATTACTACAGTAATTAATATACGATTAAATTATATAACACTAGATCGAATACCTTTATTTTGTTGAGCTGTTGTTATTACAGCACTTTTACTATTATTATCTTTACCAGTATTAGCTGGAGCTATTACTATATTATTAACAGATCGTAACTTAAATACTTCCTTTTTTGATCCAGCAGGAGGTGGAGATCCTATTTTATACCAACATTTATTT----------------------------------TCGCTTGTTTATCAAAAACATGTCTTTTTTGAATATAATTTAAAGTCTGACCTGCCCACTGAAAAAT--TTTGAAGGGCCGCGGTATATTGACTGTGCAAAGGTAGCATAATCATTTGTCTTTTAATTGAAGGCTAGAATGAAAGGTTGAATGAAGTATTAACTGTCTCATTTTAAAAATGTTAAAAATTTAATTTTTAAGTCAAAAAGCTTAAATTAAATTAGAGGACGAGAAGACCCTATAGATCTTTATAATTAATGTTTTTA---TTAGGAGTAGAAAATAATTTTTATTAAAAATGTAA--ATTATTTTATTGGGGTGATAGGAAAATTAATTAAACTTTTTTTATAATTTAAA--CATTGATTTATGATTAATTGATCCATTATTTATGATTAAAAATTTAAGTTACCTTAGGGATAACAGCGTA-ATTTTTTTAGAGAGTTCTTATCGATAAAAAAGATTGCGACCTCGATGTTGAATTAAAGGTTATTTTTAGATGCAGAAGTTTAA-AGTTTAGGTCTGTTCCGCCTTTGAATCTAATTACCCACTCCCGGCACGGGGAGGTAGTGACGAAAAATAACGATACGGGACTCATCCGAGGCCCCGTAATCGGAATGAGTACACTTTAAATCCTTTAACAAGGATCCATTGGAGGGCAAGTCTGGTGCCAGCAGCCGCGGTAATTCCAGCTCCAATAGCGTATATTAAAGTTGTTGCGGTTAAAAAGCTCGTAGTCGAATCTGTGTCTCACACTGTTGGTTCACCGCTCGCGGTGTTCAACTGGCATGTTATGTGAGACGTCCTACCGGTGGGTGGTGTGAATTATTGTTATTGTTTTATATATAATTTTATGTTTATATTTATATTATATATTACAATATTGTAAGTGTTAAAATTTATTTTTTAATAACTGCGCTTGTATATATTTCTATTTATATATTATGTATATTATATTG-GCAATTTACATTAATAATTTGCCG-TAAGTGTTTTGCCGTGAAG-AACGGCGACAGCCCCCAATTGCAATCCCGTCGCGGTGCTCTTAACTGAGTGTCGAGGTGGGCCGGTACGTTTACTTTGAACAAATTAGAGTGCTTAAGGCAGGCTCAAATTTTGCCTGAATATTGTGTGCATGGAATAATGGAATAGGACCTCGGTTCTATTTTGTTGGTTTTCGGAACTCCGAGGTAATGATTAATACGGACAGATGGGGGCATTCGTATTGCGACGTTAGAGGTGAAATTCTTGGATCGTCGCAAGACGGACAGAAGCGAAAGCATTTGCCAAAAATGTTTTGATTGATCAAGAACGAAAGTTAGATGTTCGAAGGCGATCAGATACCGCCCTAGTTCTAACCATAAACGATGCCAGCTAGCGATCCGCCGAAGTTCCTCCGATGACTCGGCGGGCAGCTT---CCGGGAAACCAAAGCTTTTGGGTTCCGGGGGAAGTATGGTTGCAAAGCTGAAACTTAAAGGAATTGACGGAAGGGCACCACCAGGAGTGGAGCCTGCGGCTTAATTTGACTCAACACGGGAAACCTCACCAGGCCCGGACACCGGAAGGATTGACAGATTGAGAGCTCTTTCTTGATTCGGTGGGTGGTGGTGCATGGCCGTTCTTAGTTGGTGGAGCGATTTGTCTGGTTAATTCCGATAACGAACGAGACTCTAGCCTGCTAAATAGACGTTTCTCCGGCATCTCAAGGCCCACCGGCTGTTTGTTTTCCGTTATGTTGTTGGCATGATTTAATATTTTAATATGATATATGTGTATATTGAAAATATTTTTATTTTGTTTTGGTATTTCAGTGTACTTATACAGGCTAAATTGTTATATATTTTGTTTTCCGCATATATATATGACAATCTGTATTTAGTGTACTGGGTCCATTCAAATAAAATATCGTAGATATATATGTGTTTATGTTTTATGTTTGCATGTTGACACATGCGGGCTACAGTTATTTGGTGTGGTTTTTACTGCCGGCGTACATTTAAAACTTCTTAGAGGGACAGGCGGCTTCTAGCCGCACGAGATTGAGCAATAACAGGTCTGTGATGCCCTTAGATGTTCTGGGCCGCACGCGCGCTACACTGAAGGAATCAGCGTGTCCTCCCTGGCCGAAAGGTCCGGGTAACCCGCTGAACCTCCTTCGTGCTAGGGATTGGGGCTTGCAATTGTTCCCCATGAACGAGGAATTCCCAGTAAGCGCGAGTCATAAGCTCGCGTTGATTACGTCCCTGCCCTTTGTACACACCGCCCGTCGCTACTACCGATTGAATGATTTAGTGAGGTCTTCGGACTGGTACGCGGTAATGTTTCTGACCTTACCGATGTTGCTGGGAAGATGACCAAACTTGATCATTTAGAGGAAGTAAAAGTCGTAACAAGGTTTCCGTTAG--------------------------------------------------------------------------------------------------------------------------------------------------------------------------------------------------------------------------------------------------------------------------------------------------------------------------------------------------------------------------------------------------------------------------------------------------------------------------------------------------------------------------------------ACGGACTCAAAACGGTGCTACATGACATCACAAAACCACGGTTTCGCCGTCGACACAGAAAAACAGAATCGCGATTGGGAGCCTTTATTCACCAACGCTAACGACAAAACAAACGAGGGAATCGTCCACAAAACGTTGCCTTACTTCAGCGTCCAGTTCCACCCCGAACACACCGCCGGTCCAGAAGACTTGGAGTGCCTTTTCGACGTGTTCCTCGACTCCGTGAAGA---ACAACGCCAGAAA---------CATTAAGGACTTAATTAACGAACGACTAACGTACAAATCTGATATACATTCCAAATTACCCGACATTCCAAAAAAGGTGCTCATCATAGGCTCCGGGGGCCTGTCCATCGGACAAGCGGGCGAATTCGACTACTCAGGCTCGCAGGCCATTAAGGCGCTTAAAGAGCAGAACGTCCAAACGGTGCTGATGAACCCAAACATCGCTACAGTGCAAACTTCCAAGGGCCTAGCGGATAAGGTCTACTTCCTGCCGTTGATTCCGGAGTACGTCGAGCAAGTGATACGATCAGAAAGACCCGGCGGAGTCTTGTTGACTTTCGGCGGACAGACGGCGTTGAACTGCGGCGTTGAATTGGAGAAAGCCGGCGTTTTCGCAAAATATGGATGCAAAATTTTGGGCACGCCCATCCAGTCGATCATAGACACGGAAGATCGAAAGAAATTCGCGGAGAAAATCAACGAAATCGGCGAGAAAGTCGCGCCCAGCGCCGCTGTTTACTCGATTCAGGAAGCCCTGGACGCCGCAGACAAGATTGGGTACCCGGTCATGGCCCGCGCTGCGTTCTCGCTCGGCGGTTTGGGCTCCGGGTTCGCTCACAACAAAGAGCAGCTGCAGAACTTAGCTTCGCAAGCGCTAGCGCACTCCAACCAGTTGATCATCGACAAGTCGTTGAAAGGATGGAAGGAAGTGGAGTACGAGGTCGTCCGGGACGCTTACGACAACTGCATAACGGTGTGCAACATGGAGAACGTGGATCCCTTGGGAATACACACCGGAGAGTCGATAGTCGTGGCTCCGAGTCAAACCCTGTCCAATCAGGAGTACAACATGCTGCGAACGACCGCGATCAAAGTCATCCGCCACTTCCAAGTCGTCGGCGAGTGCAACATCCAGTACGCCCTGAACCCCCAGTCCGCCGAGTACTACATAATCGAGGTGAACGCCCGCTTATCCCGGAGCTCTGCCCTGGCGAGCAAAGCGACGGGGTACCCGCTAGCCTACGTGGCGGCGAAACTCGCTCTGGGATCGGCGCTGCCAGACATCAAAAACTCCGTGACCGGCGAGACAACGGCCTGTTTCGAGCCCAGCTTGGACTACTGCGTCGTGAAGATCCCCCGCTGGGATCTGAGCAAATTCACGCGGGTCAGTTCCAAGATCGGCAGCTCCA-TGAAGAGCGTCGGCGAAGTGATGGCAATCGGGCGGAAGTTCGAGGAGGCGTTCCAGAAAGCGCTGCGCATGGTCGACGAGAACGTCATGGGCT-------TTGATCCGAACTTGCAGAAGATCAGCGACGAGGATTTGAAGGAGCCCACGGAC----AAGCGGATGTTTGTGATCGCGGCCTCTCTCCAGGCCGGTTACACGGTCGATCGTCTTCACGAGCTAACCAAGATCGATCGCTGGTTTCTGCAGAAGATGAAGAATATCATCGAACTCTACTCGACGCTTACTAATTT----CACGTCGCAAGCGC---TGACACGGGATATGTTGCTGCAAGCAAAGCGGTACGGGTTTTCCGATAAGCAAGTGGCAAGCGCGGTGAAAAGTACGGAACTAGCTATCCGGAAACAACGGGAAGAATACGATATCACGCCGTTCGTGAAGCAGATCGACACTGTGGCCGCGGAATGGCCCGCATGCACAAACTATCTGTATATGACTTA------CAACG-CGCTGTCGCATGACCTGGTGTTCCCAGGCGGCTACACGATGGTCATTGGCTCCGGT--GTCTATCGAATAGGCAGCTCCGTGGAGTTCGA--CTGGTGC-----GCCGTCG-GTTGTCTACGCGAGTTGCGCAAATTGGGCAAG--------AAAACCGTGATGGTCAACTACAACCCGGAGACGGTCAGCACCGATTACGATATGTGCGACCGTC-TGTACTTCGAGGAGATTTCGTTTGAGGTCGT-GATGG-ATATCTACAACG---------------------Asadeteva   0110000000??000?0?000????1010111000100011111110100000001?1100111000010101211000201111110211100020101011------------------------------------------------------------------------------------------------------------------------------------------------------------------------------------------------------------------------------------------------------------------------------------------------------------------------------------------------------------------------------------------------------------------------------------------------------------------------------------------------------------------------------------------------------------------------------------------------------------------------------------------------------------------------------------------------------------------------------------------------------------------------------------------------------------------------------------------------------------------------------------------------------------------------------------------------------------------------------------------------------------------------------------------------------------------------------------------------------------------------------------------------------------------------------------------------------------------------------------------------------------------------------------------------------------------------------------------------------------------------------------------------------------------------------------------------------------------------------------------------------------------------------------------------------------------------------------------------------------------------------------------------------------------------------------------------------------------------------------------------------------------------------------------------------------------------------------------------------------------------------------------------------------------------------------------------------------------------------------------------------------------------------------------------------------------------------------------------------------------------------------------------------------------------------------------------------------------------------------------------------------------------------------------------------------------------------------------------------------------------------------------------------------------------------------------------------------------------------------------------------------------------------------------------------------------------------------------------------------------------------------------------------------------------------------------------------------------------------------------------------------------------------------------------------------------------------------------------------------------------------------------------------------------------------------------------------------------------------------------------------------------------------------------------------------------------------------------------------------------------------------------------------------------------------------------------------------------------------------------------------------------------------------------------------------------------------------------------------------------------------------------------------------------------------------------------------------------------------------------------------------------------------------------------------------------------------------------------------------------------------------------------------------------------------------------------------------------------------------------------------------------------------------------------------------------------------------------------------------------------------------------------------------------------------------------------------------------------------------------------------------------------------------------------------------------------------------------------------------------------------------------------------------------------------------------------------------------------------------------------------------------------------------------------------------------------------------------------------------------------------------------------------------------------------------------------------------------------------------------------------------------------------------------------------------------------------------------------------------------------------------------------------------------------------------------------------------------------------------------------------------------------------------------------------------------------------------------------------------------------------------------------------------------------------------------------------------------------------------------------------------------------------------------------------------------------------------------------------------------------------------------------------------------------------------------------------------------------------------------------------------------------------------------------------------------------------------------------------------------------------------------------------------------------------------------------------------------------------------------------------------------------------------------------------------------------------------------------------------------------------------------------------------------------------------------------------------------------------------------------------------------------------------------------------------------------------------------------------------------------------------------------------------------------------------------------------------------------------------------------------------------------------------------------------------------------------------------------------------------------------------------------------Austroberothella   001000000000000000001110100100010001011000101011000000010000100100011100001000011-211100210011001112011------------------------------------------------------------------------------------------------------------------------------------------------------------------------------------------------------------------------------------------------------------------------------------------------------------------------------------------------------------------------------------------------------------------------------------------------------------------------------------------------------------------------------------------------------------------------------------------------------------------------------------------------------------------------------------------------------------------------------------------------------------------------------------------------------------------------------------------------------------------------------------------------------------------------------------------------------------------------------------------------------------------------------------------------------------------------------------------------------------------------------------------------------------------------------------------------------------------------------------------------------------------------------------------------------------------------------------------------------------------------------------------------------------------------------------------------------------------------------------------------------------------------------------------------------------------------------------------------------------------------------------------------------------------------------------------------------------------------------------------------------------------------------------------------------------------------------------------------------------------------------------------------------------------------------------------------------------------------------------------------------------------------------------------------------------------------------------------------------------------------------------------------------------------------------------------------------------------------------------------------------------------------------------------------------------------------------------------------------------------------------------------------------------------------------------------------------------------------------------------------------------------------------------------------------------------------------------------------------------------------------------------------------------------------------------------------------------------------------------------------------------------------------------------------------------------------------------------------------------------------------------------------------------------------------------------------------------------------------------------------------------------------------------------------------------------------------------------------------------------------------------------------------------------------------------------------------------------------------------------------------------------------------------------------------------------------------------------------------------------------------------------------------------------------------------------------------------------------------------------------------------------------------------------------------------------------------------------------------------------------------------------------------------------------------------------------------------------------------------------------------------------------------------------------------------------------------------------------------------------------------------------------------------------------------------------------------------------------------------------------------------------------------------------------------------------------------------------------------------------------------------------------------------------------------------------------------------------------------------------------------------------------------------------------------------------------------------------------------------------------------------------------------------------------------------------------------------------------------------------------------------------------------------------------------------------------------------------------------------------------------------------------------------------------------------------------------------------------------------------------------------------------------------------------------------------------------------------------------------------------------------------------------------------------------------------------------------------------------------------------------------------------------------------------------------------------------------------------------------------------------------------------------------------------------------------------------------------------------------------------------------------------------------------------------------------------------------------------------------------------------------------------------------------------------------------------------------------------------------------------------------------------------------------------------------------------------------------------------------------------------------------------------------------------------------------------------------------------------------------------------------------------------------------------------------------------------------------------------------------------------------------------------------------------------------------------------------------------------------------------------------------------------------------------------------------------------------------------------------------------------------------------------Berlekrumyia   0110000000??000?0?000????0001111111--1001111010100011001?10012110000100112110002011111102110001111?1011------------------------------------------------------------------------------------------------------------------------------------------------------------------------------------------------------------------------------------------------------------------------------------------------------------------------------------------------------------------------------------------------------------------------------------------------------------------------------------------------------------------------------------------------------------------------------------------------------------------------------------------------------------------------------------------------------------------------------------------------------------------------------------------------------------------------------------------------------------------------------------------------------------------------------------------------------------------------------------------------------------------------------------------------------------------------------------------------------------------------------------------------------------------------------------------------------------------------------------------------------------------------------------------------------------------------------------------------------------------------------------------------------------------------------------------------------------------------------------------------------------------------------------------------------------------------------------------------------------------------------------------------------------------------------------------------------------------------------------------------------------------------------------------------------------------------------------------------------------------------------------------------------------------------------------------------------------------------------------------------------------------------------------------------------------------------------------------------------------------------------------------------------------------------------------------------------------------------------------------------------------------------------------------------------------------------------------------------------------------------------------------------------------------------------------------------------------------------------------------------------------------------------------------------------------------------------------------------------------------------------------------------------------------------------------------------------------------------------------------------------------------------------------------------------------------------------------------------------------------------------------------------------------------------------------------------------------------------------------------------------------------------------------------------------------------------------------------------------------------------------------------------------------------------------------------------------------------------------------------------------------------------------------------------------------------------------------------------------------------------------------------------------------------------------------------------------------------------------------------------------------------------------------------------------------------------------------------------------------------------------------------------------------------------------------------------------------------------------------------------------------------------------------------------------------------------------------------------------------------------------------------------------------------------------------------------------------------------------------------------------------------------------------------------------------------------------------------------------------------------------------------------------------------------------------------------------------------------------------------------------------------------------------------------------------------------------------------------------------------------------------------------------------------------------------------------------------------------------------------------------------------------------------------------------------------------------------------------------------------------------------------------------------------------------------------------------------------------------------------------------------------------------------------------------------------------------------------------------------------------------------------------------------------------------------------------------------------------------------------------------------------------------------------------------------------------------------------------------------------------------------------------------------------------------------------------------------------------------------------------------------------------------------------------------------------------------------------------------------------------------------------------------------------------------------------------------------------------------------------------------------------------------------------------------------------------------------------------------------------------------------------------------------------------------------------------------------------------------------------------------------------------------------------------------------------------------------------------------------------------------------------------------------------------------------------------------------------------------------------------------------------------------------------------------------------------------------------------------------------------------------------------------Berotha   011100000000000001001110100100100001010001110101000100011010010100011120121000020-111111311000021101011???????????????????????????????????????????????????????????????????????????????????????????????????????????????????????????????????????????????????????????????????????????????????????????????????????????????????????????????????????????????????????????????????????????????????????????????????????????????????????????????????????????????????????????????????????????????????????????????????????????????????????????????????????????????????????????????????????????????????????????????????????????????????????????????????????????????????????????????????????????????????????????????????????????????????????????????????????????????????????????????????????????????????????????????????????????????????????????????????????????????????????????????????????????????------------------------TTTTT-GAATTTAATAAAAAGTCTGACCTGCCCACTGAATAAAT-TTTAAAGGGCCGCGGTATTTTGACTGTGCAAAGGTAGCATAATCATTTGTCTTTTAATTGGAGGCTAGAATGAATGGTTAAATGAAGTATTAACTGTCTCATTTTAATAAGTATAAAAATTTAATTTTTTAGTTAAAAAGCTAAAATGTTATTAAAGGACGAGAAGACCCTATAAATCTTTAT--TT-ATTTATATAAATTTAATATTAGATTATAATTATTAATTATTTATAAA--TAAATTTTATTGGGGTGATAGGAAAATTAATCAAACTTTTTTTT--ATTTTAAACTATTGATTTATAAATTATTGATCCATTATTAATGATTATAAGATTAAGATACTTTAGGGATAACAGCGTA-ATTTTTTTAGAGAGTTCTTATCGATAAAAAAGATTGCGACCTCGATGTTGAATTAAAATTAAATTTTAAATGTAGCAGTTTAAGATATTAGGTCTGTTCGACCTTTAAATTT??????????????????????????????????????????????????????????????????????????????????????????????????????????????????????????????????????????????????????????????????????????????????????????????????????????????????????????????????????????????????????????????????????????????????????????????????????????????????????????????????????????????????????????????????????????????????????????????????????????????????????????????????????????????????????????????????????????????????????????????????????????????????????????????????????????????????????????????????????????????????????????????????????????????????????????????????????????????????????????????????????????????????????????????????????????????????????????????????????????????????????????????????????????????????????????????????????????????????????????????????????????????????????????????????????????????????????????????????????????????????????????????????????????????????????????????????????????????????????????????????????????????????????????????????????????????????????????????????????????????????????????????????????????????????????????????????????????????????????????????????????????????????????????????????????????????????????????????????????????????????????????????????????????????????????????????????????????????????????????????????????????????????????????????????????????????????????????????????????????????????????????????????????????????????????????????????????????????????????????????????????????????????????????????????????????????????????????????????????????????????????????????????????????????????????????????????????????????????????????????????????????????????????????????????????????????????????????????????????????????????????????????????????????????????????????????????????????????????????????????????????????????????????????????????????????????????????????????????????????????????????????????????????????????????????????????????????????????????????????????????????????????????????????????????????????????????????????????????????????????????????????????????????????????????????????????????????????????????????????????????????????????????????????????????????????????????????????????????????????????????????????????????????????????????????????????????????????????????????????????????????????????????????????????????????????????????????????????????????????????????????????????????????????????????????????????????????????????????????????????????????????????????????????????????????????????????????????????????????????????????????????????????????????????????????????????????????????????????????????????????????????????????????????????????????????????????????????????????????????????????????????????????????????????????????????????????????????????????????????????????????????????????????????????????????????????????????????????????????????????????????????????????????????????????????????????????????????????????????????????????????????????????????????????????????????????????????????????????????????????????????????????????????????????????????????????????????????????????????????????????????????????????????????????????????????????????????????????????????????????????????????????????????????????????????????????????????????????????????????????????????????????????????????????????????????????????????????????????????????????????????????????????????????????????????????????????????????????????????????????????????????????????????????????????????????????????????????????????????????????????????????????????????????????????????????????????????????????????????????????????????????????????????????????????????????????????????????????????????????????????????????????????????????????????????????????????????????????????????????????????????????????????????????????????????????????????????????????????????????????????????????????????????????????????????????????????????????????????????????????????????????????????????????????????????????????????????????????????????????????????????????????????????????????????????????????????????????????????????????????????????????????????????????????????????????????????????????????????????????????????????????????????????????????????????????????????????????????????????????????????????????????????????????????????????????????????????????????????????????????????????????????????????????????????????????????????????????????????????????????????????????????????????????????????????????????????????????????????????????????????????????????????????????????????????????????????????????????????????????????????????????????????????????????????????????????????????????????????????????????????????????????????????????????????????????????????????-Berothimerobius   011000000000000000000????0001101111--0001100010100000001?110120100010101110000??11????001110011011?0011------------------------------------------------------------------------------------------------------------------------------------------------------------------------------------------------------------------------------------------------------------------------------------------------------------------------------------------------------------------------------------------------------------------------------------------------------------------------------------------------------------------------------------------------------------------------------------------------------------------------------------------------------------------------------------------------------------------------------------------------------------------------------------------------------------------------------------------------------------------------------------------------------------------------------------------------------------------------------------------------------------------------------------------------------------------------------------------------------------------------------------------------------------------------------------------------------------------------------------------------------------------------------------------------------------------------------------------------------------------------------------------------------------------------------------------------------------------------------------------------------------------------------------------------------------------------------------------------------------------------------------------------------------------------------------------------------------------------------------------------------------------------------------------------------------------------------------------------------------------------------------------------------------------------------------------------------------------------------------------------------------------------------------------------------------------------------------------------------------------------------------------------------------------------------------------------------------------------------------------------------------------------------------------------------------------------------------------------------------------------------------------------------------------------------------------------------------------------------------------------------------------------------------------------------------------------------------------------------------------------------------------------------------------------------------------------------------------------------------------------------------------------------------------------------------------------------------------------------------------------------------------------------------------------------------------------------------------------------------------------------------------------------------------------------------------------------------------------------------------------------------------------------------------------------------------------------------------------------------------------------------------------------------------------------------------------------------------------------------------------------------------------------------------------------------------------------------------------------------------------------------------------------------------------------------------------------------------------------------------------------------------------------------------------------------------------------------------------------------------------------------------------------------------------------------------------------------------------------------------------------------------------------------------------------------------------------------------------------------------------------------------------------------------------------------------------------------------------------------------------------------------------------------------------------------------------------------------------------------------------------------------------------------------------------------------------------------------------------------------------------------------------------------------------------------------------------------------------------------------------------------------------------------------------------------------------------------------------------------------------------------------------------------------------------------------------------------------------------------------------------------------------------------------------------------------------------------------------------------------------------------------------------------------------------------------------------------------------------------------------------------------------------------------------------------------------------------------------------------------------------------------------------------------------------------------------------------------------------------------------------------------------------------------------------------------------------------------------------------------------------------------------------------------------------------------------------------------------------------------------------------------------------------------------------------------------------------------------------------------------------------------------------------------------------------------------------------------------------------------------------------------------------------------------------------------------------------------------------------------------------------------------------------------------------------------------------------------------------------------------------------------------------------------------------------------------------------------------------------------------------------------------------Cyrenoberotha   0110100000000000000011001000101000010110001000110000010100000101000023-0100001001-211000020010001110011------------------------------------------------------------------------------------------------------------------------------------------------------------------------------------------------------------------------------------------------------------------------------------------------------------------------------------------------------------------------------------------------------------------------------------------------------------------------------------------------------------------------------------------------------------------------------------------------------------------------------------------------------------------------------------------------------------------------------------------------------------------------------------------------------------------------------------------------------------------------------------------------------------------------------------------------------------------------------------------------------------------------------------------------------------------------------------------------------------------------------------------------------------------------------------------------------------------------------------------------------------------------------------------------------------------------------------------------------------------------------------------------------------------------------------------------------------------------------------------------------------------------------------------------------------------------------------------------------------------------------------------------------------------------------------------------------------------------------------------------------------------------------------------------------------------------------------------------------------------------------------------------------------------------------------------------------------------------------------------------------------------------------------------------------------------------------------------------------------------------------------------------------------------------------------------------------------------------------------------------------------------------------------------------------------------------------------------------------------------------------------------------------------------------------------------------------------------------------------------------------------------------------------------------------------------------------------------------------------------------------------------------------------------------------------------------------------------------------------------------------------------------------------------------------------------------------------------------------------------------------------------------------------------------------------------------------------------------------------------------------------------------------------------------------------------------------------------------------------------------------------------------------------------------------------------------------------------------------------------------------------------------------------------------------------------------------------------------------------------------------------------------------------------------------------------------------------------------------------------------------------------------------------------------------------------------------------------------------------------------------------------------------------------------------------------------------------------------------------------------------------------------------------------------------------------------------------------------------------------------------------------------------------------------------------------------------------------------------------------------------------------------------------------------------------------------------------------------------------------------------------------------------------------------------------------------------------------------------------------------------------------------------------------------------------------------------------------------------------------------------------------------------------------------------------------------------------------------------------------------------------------------------------------------------------------------------------------------------------------------------------------------------------------------------------------------------------------------------------------------------------------------------------------------------------------------------------------------------------------------------------------------------------------------------------------------------------------------------------------------------------------------------------------------------------------------------------------------------------------------------------------------------------------------------------------------------------------------------------------------------------------------------------------------------------------------------------------------------------------------------------------------------------------------------------------------------------------------------------------------------------------------------------------------------------------------------------------------------------------------------------------------------------------------------------------------------------------------------------------------------------------------------------------------------------------------------------------------------------------------------------------------------------------------------------------------------------------------------------------------------------------------------------------------------------------------------------------------------------------------------------------------------Isoscelipteron   0110?00000??000?0?000??0?1000111000110011111010100010001?0100111000010101110101201112011321000020101011---------------------------------------------------------AA-CTTTATACTTTATTTTCGGAATTTGATCTGGATTAGTAGGAACAAGCCTTAGTTTATTAATTCGTGCTGAATTAGGTCAACCAGGGTCTCTAATTGGAGATGATCAAATTTATAAT-GTTATTGTAACAGCCCATGCTTTTATTATAATTTTTTTTATAGTAATACCAATTGTTATTGGAGGGTTTGGTAATTGATTAGTTCCTTTAATACTAGCAGCTCCTGATATAGCTTTTCCTCGAATAAATAATATAAGCTTTTGATTTCTTCCTCCTTCATTAACACTTCTTCTAGCTTCATCTATAGTAGAAAGTGGGGCAGGAACAGGATGAACAGTGTATCCTCCTCTAGCTTCTGGAATTGCTCATGCAGGTGCATCAGTTGATTTAGCAATTTTTAGTTTACATTTAGCAGGTGTTTCTTCAATTTTAGGGGCTGTAAATTTTATTACAACTGTAATTAATATACGTCTACCTTATATAACATTAGATCGTATGCCTTTATTCTGTTGAGCTGTTGTAATTACAGCTATTCTTCTATTACTATCTTTACCAGTATTAGCAGGTGCTATTACTATACTTTTAACAGATCGTAATTTAAATACTTCTTTTTTTGACCCAGCTGGAGGAGGAGACCCTATTCTTTATCAACATTTATTT----------------------------------??????????????????????????????????????????????????????????????????????????????????????????????????????????????????????????????????????????????????????????????????????????????????????????????????????????????????????????????????????????????????????????????????????????????????????????????????????????????????????????????????????????????????????????????????????????????????????????????????????????????????????????????????????????????????????????????????????????????????????????????????????????????????????????????????????????????????????????????????????????????????????????????????????????????????????????????????????????????????????????????????????????????????????????????????????????????????????????????????????????????????????????????????????????????????????????????????????????????????????????????????????????????????????????????????????????????????????????????????????????????????????????????????????????????????????????????????????????????????????????????????????????????????????????????????????????????????????????????????????????????????????????????????????????????????????????????????????????????????????????????????????????????????????????????????????????????????????????????????????????????????????????????????????????????????????????????????????????????????????????????????????????????????????????????????????????????????????????????????????????????????????????????????????????????????????????????????????????????????????????????????????????????????????????????????????????????????????????????????????????????????????????????????????????????????????????????????????????????????????????????????????????????????????????????????????????????????????????????????????????????????????????????????????????????????????????????????????????????????????????????????????????????????????????????????????????????????????????????????????????????????????????????????????????????????????????????????????????????????????????????????????????????????????????????????????????????????????????????????????????????????????????????????????????????????????????????????????????????????????????????????????????????????????????????????????????????????????????????????????????????????????????????????????????????????????????????????????????????????????????????????????????????????????????????????????????????????????????????????????????????????????????????????????????????????????????????????????????????????????????????????????????????????????????????????????????????????????????????????????????????????????????????????????????????????????????????????????????????????????????????????????????????????????????????????????????????????????????????????????????????????????????????????????????????????????????????????????????????????????????????????????????????????????????????????????????????????????????????????????????????????????????????????????????????????????????????????????????????????????????????????????????????????????????????????????????????????????????????????????????????????????????????????????????????????????????????????????????????????????????????????????????????????????????????????????????????????????????????????????????????????????????????????????????????????????????????????????????????????????????????????????????????????????????????????????????????????????????????????????????????????????????????????????????????????????????????????????????????????????????????????????????????????????????????????????????????????????????????????????????????????????????????????????????????????????????????????????????????????????????????????????????????????????????????????????????????????????????????????????????????????????????????????????????????????????????????????????????????????????????????????????????????????????????????????????????????????????????????????????????????????????????????????????????????????????????????????????????????????????????????????????????????????????????????????????????????????????????????????????????????????????????????????????????????????????????????????????????????????????????????????????????????????????????????????????????????????????????????????????????????????????????????????????????????????????????????????????????????????????????????????????????????????????????????????????????????????????????????????????????????????????????????????????????????????????????????????????????????????????????????????????????????????????????????????????????????????????????????????????????????????????????????????????????????????????????????????????????????????????????????????????????????????????????????????????????????????????????????????????????????????????????????????????????????????????????????????????????????????????????????????????????????????????????????????????????????????????????????????????????????????????????????????????????????????????????????????????????????????????????????????????????????????????????????????????????????????????????????????????????????????????????????????????????????????????????????????????????????????????????????????????????????????????????????????????????????????????????????????????????????????????????????????????????????????????????????????????????????????????????????????????????????????????????????????????-Lekrugeria   0111000000??000?0?000????0011210111--0001111010100010001?1101211000010011211000201111110311000021100011------------------------------------------------------------------------------------------------------------------------------------------------------------------------------------------------------------------------------------------------------------------------------------------------------------------------------------------------------------------------------------------------------------------------------------------------------------------------------------------------------------------------------------------------------------------------------------------------------------------------------------------------------------------------------------------------------------------------------------------------------------------------------------------------------------------------------------------------------------------------------------------------------------------------------------------------------------------------------------------------------------------------------------------------------------------------------------------------------------------------------------------------------------------------------------------------------------------------------------------------------------------------------------------------------------------------------------------------------------------------------------------------------------------------------------------------------------------------------------------------------------------------------------------------------------------------------------------------------------------------------------------------------------------------------------------------------------------------------------------------------------------------------------------------------------------------------------------------------------------------------------------------------------------------------------------------------------------------------------------------------------------------------------------------------------------------------------------------------------------------------------------------------------------------------------------------------------------------------------------------------------------------------------------------------------------------------------------------------------------------------------------------------------------------------------------------------------------------------------------------------------------------------------------------------------------------------------------------------------------------------------------------------------------------------------------------------------------------------------------------------------------------------------------------------------------------------------------------------------------------------------------------------------------------------------------------------------------------------------------------------------------------------------------------------------------------------------------------------------------------------------------------------------------------------------------------------------------------------------------------------------------------------------------------------------------------------------------------------------------------------------------------------------------------------------------------------------------------------------------------------------------------------------------------------------------------------------------------------------------------------------------------------------------------------------------------------------------------------------------------------------------------------------------------------------------------------------------------------------------------------------------------------------------------------------------------------------------------------------------------------------------------------------------------------------------------------------------------------------------------------------------------------------------------------------------------------------------------------------------------------------------------------------------------------------------------------------------------------------------------------------------------------------------------------------------------------------------------------------------------------------------------------------------------------------------------------------------------------------------------------------------------------------------------------------------------------------------------------------------------------------------------------------------------------------------------------------------------------------------------------------------------------------------------------------------------------------------------------------------------------------------------------------------------------------------------------------------------------------------------------------------------------------------------------------------------------------------------------------------------------------------------------------------------------------------------------------------------------------------------------------------------------------------------------------------------------------------------------------------------------------------------------------------------------------------------------------------------------------------------------------------------------------------------------------------------------------------------------------------------------------------------------------------------------------------------------------------------------------------------------------------------------------------------------------------------------------------------------------------------------------------------------------------------------------------------------------------------------------------------------------------------------Lomamyia   111000000000000001001110110000110001001000110011000000011000010100001120121000120-211111311000021101011---------------------ACAATGATTATTTTCAACTAACCATAAAGATATTGGAA-CTTTATATTTTATTTTCGGGATTTGATCTGGACTAGTAGGAACTAGTTTAAGTTTATTAATTCGAGCTGAATTAGGACAGCCAGGATCATTAATTGGTGATGACCAGATTTATAAT-GTAATTGTTACAGCACATGCATTTATTATAATTTTTTTTATAGTAATACCTATTGTAATTGGAGGATTTGGAAATTGATTAGTACCTTTAATATTAGCAGCACCAGATATAGCATTTCCTCGTATAAATAATATAAGTTTTTGATTCCTCCCTCCTTCTTTAACTCTATTATTAGCTTCATCAATAGTAGAAAGAGGAGCTGGAACAGGATGAACAGTTTACCCTCCTCTAGCTTCAGGAATTGCTCATGCAGGAGCTTCAGTTGATTTAGCCATTTTTAGTTTACACCTTGCCGGAGTTTCTTCAATTTTAGGGGCAGTAAATTTTATTACAACTGTTATTAATATACGGTTATCTTATATAACTCTTGACCGTATACCTTTATTCTGTTGAGCAGTTGTTATTACTGCTTTATTATTATTATTATCTTTACCAGTATTAGCAGGAGCTATTACAATATTATTAACTGATCGTAATTTAAATACTTCTTTCTTTGATCCAGCAGGAGGAGGAGATCCTATTTTATACCAACATTTATTT----------------------------------CCGCTTGTTTATCAAAAACATGTCTTTTT-GAAAATAATATAAAGTCTGACCTGCCCACTGAAAGT---TTTGAAGGGCCGCGGTATATTGACTGTGCAAAGGTAGCATAATCATTTGTCTCTTAATTGGAGGCTAGAATGAATGGTTGAATGAGGTATTAACTGTCTCATTTTAATAAATTTAAAAATTTAATTTTTTAGTCAAAAAGCTAAAATTTTATTGGAGGACGAGAAGACCCTATAAATCTTTAT--TTAATTAGAATAT-TTTAATAGTAGAAAATAATAATTAAATTAATTTAAT--TAAATTTTATTGGGGTGATAGGAAAATTAAATAAACTTTTTTTA--ATTTAAA--CATTGATTTATGATAATTTGATCCATTATGAGTGATTAAAAGTTTAAGATACTTTAGGGATAACAGCGTA-ATTTTTTTAGAGAGTTCATATCGATAAAAAAGATTGCGACCTCGATGTTGAATTAAAGATAAATTTTAAATGCAGAAGTTTAA-AAATTAGGTCTGTTCGACCTTTGAATCC---------------------------------------------------------------CTCAAAGATTAAGCCATGCATGTCTCAGTACAAGCCAAATTAAGGTGAAACCGCGAAAGGCTCATTATATCAGTTATGGTTCCTTAGATCGTACCCACATTTACTTGGATAACTGTGGT-----AATTCTAGAGCTAATACATG---CAAACAG--AGTTC-CGACCAGAGATGGAAGGAATGCTTTTATTAGATCAAAACCAATCGATGTT-------------------------TCATATTTGTACATTAATTT------GTAT---------------------------------------------------------TATTTATG----TT------GCATCGTTCTTTTTGGTGACTCTGAATAACTTTAAGCTGATCGCACGGTCTC------GTACCGGCGACGCATCTTTCAAAT---------GTCTGCCTTATCAACTGTCGATGGTAGGTTTTGCGCCTACCATGG-----TTGTAACGGGTAACGGGGAATCAGGGTTCG-------ATTCCGGAGAGGGAGCCTGAGAAACGGCTACCACATCCAA------GGAAGGCAGCAGGCGCGCAAATTACCCACTCCCGGCACGGGGAGGTA--------GTGACG--AAAAATAACGATACGGGACTCATCCGAGGCCCCGTAATCGGAATGAGTACACTTTAAATCCTTTAACG-AGGATCCATTAGAGAGGGCAATTCTGGTGCCAACCG---CCGCGGTAATTCCAGCTCTAATAGCGTATATTAAAGTTGTTGCGGTTAAAAAGCTCGTAGCCGAATCTGTGTCTCACACTGTCGGTTCACCGCTCGCGGTGTTCAACTGGCATGTTATGTGAGACGTCCTACCGGTGGGTGGTGTAAATTATT-----------------------------------------------------------------------------------------------------------------------------------------------------------------------------------------------------------------------------------------------------------------------------------------------------------------------------------------------------------------------------------------------------------------------------------------------------------------------------------------------------------------------------------------------------------------------------------------------------------------------------------------------------------------------------------------------------------------------------------------------------------------------------------------------------------------------------------------------------------------------------------------------------------------------------------------------------------------------------------------------------------------------------------------------------------------------------------------------------------------------------------------------------------------------------------------------------------------------------------------------------------------------------------------------------------------------------------------------------------------------------------------------------------------------------------------------------------------------------TGCATACATCATGG-AACAGGTCGTTGCTATATGACATCACAGAATCACGGTTTTGCGGTAGATGTCAAAACTTTACCCGATGATTGGGAAGCGCTATTTACTAACGCTAATGACAAAACCAATGAAGGAATAATTCATAAAGAATTGCCATACTTCAGTGTGCAATTTCATCCAGAACATACAGCTGGCCCAGAAGACTTGGAATGTCTCTTTGATGTCTTCTTAAAAACCGTTAAGTTCTACACTGAACATAAATCTATTTGCATTAAAGATGAACTTACAAAGCGACTAAGTTATGAAC---GTCCAGTTTCCACTCAAATCGACATTCCAAAAAAAGTTCTCATCATCGGTTCTGGCGGGTTATCTATCGGTCAAGCGGGCGAATTCGATTATTCTGGATCACAAGCAATTAAAGCATTGAAAGAGGAAAATATACAAACGATTTTAATGAACCCAAATATTGCAACAGTACAAACATCGAAAGGTCTCGCTGATAAGGTCTACTTTTTACCGTTGGTTCCCGAATATGTAGAACAAGTGATTCGCTCTGAGCGTCCAGATGGAGTTCTTTTAACTTTCGGTGGCCAAACAGCTCTAAATTGTGGAGTAGAACTCGAAAAAGCTGGTGTATTCAAAAAGTATGATGTTCGAATCTTGGGCACACCAATAAAATCGATAATCGAAACGGAAGATAGGAAGATATTTGCTGATCGTGTAAATGAAATTGGTGAGAAAGTTGCGCCTAGTGCAGCAGTTTACTCTGTCCAAGAAGCTTTGAACGCCGCTGAAACAATTGGGTATCCAGTAATGGCTCGCGCTGCATTCTCCCTAGGAGGTTTAGGATCTGGTTTTGCAAATAATTCCGACGAATTAAAGATACTCGCAACACAGGCATTGGCACATTCAAGTCAATTGATCATCGATAAGTCGTTGAGAGGTTGGAAAGAAGTTGAATATGAAATAGTTCGCGATGCTTTTGATAATTGCATTACCGTTTGCAACATGGAGAATGTTGATCCATTGGGCATTCATACCGGTGAATCGATTGTTGTTGCGCCAAGTCAAACTCTTTCGAATCGTGAATATAATATGTTGCGAACAACTGCGATTAACGTGATTCGTCACTTCGGTGTCGTTGGAGAATGTAACATCCAGTATGCTTTGAATCCCCATTCAGAAGAATATTACATAATTGAAGTGAATGCTCGTCTATCACGAAGTTCAGCTTTGGCGAGTAAAGCGACAGGTTATCCATTAGCTTACGTTGCTGCCAAGTTAGCGTTAGGCTCGTCTCTTCTTGATATCAAGAATTCTGTAACAGGGGAAACCACAGCGTGTTTCGAGCCTAGTTTAGACTACTGCGTTGTAAAAATTCCACGTTGGGATTTAAGTAAATTCAGTCGCGTTAGTTCAAAAATTGGAAGCTCAA-TGAAAAGTGTTGGTGAAGTAATGGCAATCGGCCGCAAATTCGAGGAGGCATTTCAAAAAGCGTTGCGTATGGTTGATGAAAACGTTTGTGGTT-------TCGATCCTGATCTACAAAAGATTAATGACGAATGCTTGAAAGAGCCAACCGAT----AAGAGAATGTTTGTTATTGCAGCGTCTTTGAAAGCAGGTTATAGTGTTGACAAACTTCACCAATTAACCAACATTGATCCATGGTTCTTGCAAAAAATGAAGAATATCATTGACCTGCATACAATGCTAACTGCATT----GGACCAACAGGGTA---TTACACGTTCCATCCTCCATCAAGCTAAGACCTATGGATTCTCTGATAAGCAAATAGCCAATGCGGTGGCAAGCACAGAATTAGCTATTCGCAAACACCGAGAAGAATGTGGACTCCGGCCTTTCGTGAAGCAGATTGATACAGTTGCAGCAGAGTGGCCAGCATGTACAAACTACCTGTATTTGACGTA------CAACG-CTGATTCACACGACATTGATTACCCGGGTGGTTATATTATGGTCATCGGTTCTGGT--GTGTATCGAATCGGAAGTTCGGTGGAATTCGA--TTGGTGC-----GCTGTAG-GATGTTTAAGAGAATTACGCAAACTGGGAAAG--------AAAACCATTATGGTCAATTATAATCCAGAAACCGTTAGTACAGATTACGATATGTGCGATCGTT-TGTATTTTGAAGAGATTTCCTTTGAAGTGGT-TATGG-ATATCTACAATCTAGTAAATCCCGAGGGTGTT-Manselliberotha   0010100000000000000001001000101000010110001010110000010100000101000023-0100001000-211000010010001110011------------------------------------------------------------------------------------------------------------------------------------------------------------------------------------------------------------------------------------------------------------------------------------------------------------------------------------------------------------------------------------------------------------------------------------------------------------------------------------------------------------------------------------------------------------------------------------------------------------------------------------------------------------------------------------------------------------------------------------------------------------------------------------------------------------------------------------------------------------------------------------------------------------------------------------------------------------------------------------------------------------------------------------------------------------------------------------------------------------------------------------------------------------------------------------------------------------------------------------------------------------------------------------------------------------------------------------------------------------------------------------------------------------------------------------------------------------------------------------------------------------------------------------------------------------------------------------------------------------------------------------------------------------------------------------------------------------------------------------------------------------------------------------------------------------------------------------------------------------------------------------------------------------------------------------------------------------------------------------------------------------------------------------------------------------------------------------------------------------------------------------------------------------------------------------------------------------------------------------------------------------------------------------------------------------------------------------------------------------------------------------------------------------------------------------------------------------------------------------------------------------------------------------------------------------------------------------------------------------------------------------------------------------------------------------------------------------------------------------------------------------------------------------------------------------------------------------------------------------------------------------------------------------------------------------------------------------------------------------------------------------------------------------------------------------------------------------------------------------------------------------------------------------------------------------------------------------------------------------------------------------------------------------------------------------------------------------------------------------------------------------------------------------------------------------------------------------------------------------------------------------------------------------------------------------------------------------------------------------------------------------------------------------------------------------------------------------------------------------------------------------------------------------------------------------------------------------------------------------------------------------------------------------------------------------------------------------------------------------------------------------------------------------------------------------------------------------------------------------------------------------------------------------------------------------------------------------------------------------------------------------------------------------------------------------------------------------------------------------------------------------------------------------------------------------------------------------------------------------------------------------------------------------------------------------------------------------------------------------------------------------------------------------------------------------------------------------------------------------------------------------------------------------------------------------------------------------------------------------------------------------------------------------------------------------------------------------------------------------------------------------------------------------------------------------------------------------------------------------------------------------------------------------------------------------------------------------------------------------------------------------------------------------------------------------------------------------------------------------------------------------------------------------------------------------------------------------------------------------------------------------------------------------------------------------------------------------------------------------------------------------------------------------------------------------------------------------------------------------------------------------------------------------------------------------------------------------------------------------------------------------------------------------------------------------------------------------------------------------------------------------------------------------------------------------------------------------------------------------------------------------------------------Naizema   0110000000000000000011102000101100011120101010110010000101001001000010000210000100012100111000011111011---------------------------------------------------------AA-CTCTATATTTTATTTTTGGAATTTGATCAGGGCTAGTAGGAACTAGATTAAGTTTATTAATTCGTGCTGAATTAGGTCAACCAGGTTCATTAATTGGTGATGATCAGATTTATAAT-GTAATTGTTACAGCACATGCTTTTATTATAATCTTTTTTATAGTAATACCCATTGTTATTGGAGGATTTGGGAATTGATTAATTCCATTAATATTAGGAGCCCCTGATATAGCATTTCCTCGAATAAATAATATAAGATTTTGATTTTTACCACCTTCATTAACTTTATTAATAGCTTCATCAATAGTAGAAAGAGGAGCTGGTACAGGATGAACAGTTTACCCTCCTCTTGCAGCTAATATTGCTCATACTGGAGCTTCAGTAGATTTAACCATTTTTAGATTACATTTAGCAGGAATTTCTTCAATTTTGGGGGCAGTAAATTTTATTACTACTATAATTAATATACGATTATCATATATAACTTTTGAAAAAATACCATTATTTTGTTGAGCAGTTATAATTACTGCTTTATTATTATTACTTTCATTACCTGTTTTAGCAGGGGCTATTACTATATTATTAACTGATCGTAATTTAAATACATCTTTTTTTGACCCAGCTGGAGGAGGAGATCCCATTTTATATCAACATTTATTT----------------------------------??????????????????????????????????????????????????????????????????????????????????????????????????????????????????????????????????????????????????????????????????????????????????????????????????????????????????????????????????????????????????????????????????????????????????????????????????????????????????????????????????????????????????????????????????????????????????????????????????????????????????????????????????????????????????????????????????????????????????????????????????????????????????????????????????????????????????????????????????????????????????????????????????????????????????????????????????????????????????????????????????????????????????????????????????????????????????????????????????????????????????????????????????????????????????????????????????????????????????????????????????????????????????????????????????????????????????????????????????????????????????????????????????????????????????????????????????????????????????????????????????????????????????????????????????????????????????????????????????????????????????????????????????????????????????????????????????????????????????????????????????????????????????????????????????????????????????????????????????????????????????????????????????????????????????????????????????????????????????????????????????????????????????????????????????????????????????????????????????????????????????????????????????????????????????????????????????????????????????????????????????????????????????????????????????????????????????????????????????????????????????????????????????????????????????????????????????????????????????????????????????????????????????????????????????????????????????????????????????????????????????????????????????????????????????????????????????????????????????????????????????????????????????????????????????????????????????????????????????????????????????????????????????????????????????????????????????????????????????????????????????????????????????????????????????????????????????????????????????????????????????????????????????????????????????????????????????????????????????????????????????????????????????????????????????????????????????????????????????????????????????????????????????????????????????????????????????????????????????????????????????????????????????????????????????????????????????????????????????????????????????????????????????????????????????????????????????????????????????????????????????????????????????????????????????????????????????????????????????????????????????????????????????????????????????????????????????????????????????????????????????????????????????????????????????????????????????????????????????????????????????????????????????????????????????????????????????????????????????????????????????????????????????????????????????????????????????????????????????????????????????????????????????????????????????????????????????????????????????????????????????????????????????????????????????????????????????????????????????????????????????????????????????????????????????????????????????????????????????????????????????????????????????????????????????????????????????????????????????????????????????????????????????????????????????????????????????????????????????????????????????????????????????????????????????????????????????????????????????????????????????????????????????????????????????????????????????????????????????????????????????????????????????????????????????????????????????????????????????????????????????????????????????????????????????????????????????????????????????????????????????????????????????????????????????????????????????????????????????????????????????????????????????????????????????????????????????????????????????????????????????????????????????????????????????????????????????????????????????????????????????????????????????????????????????????????????????????????????????????????????????????????????????????????????????????????????????????????????????????????????????????????????????????????????????????????????????????????????????????????????????????????????????????????????????????????????????????????????????????????????????????????????????????????????????????????????????????????????????????????????????????????????????????????????????????????????????????????????????????????????????????????????????????????????????????????????????????????????????????????????????????????????????????????????????????????????????????????????????????????????????????????????????????????????????????????????????????????????????????????????????????????????????????????????????????????????????????????????????????????????????????????????????????????????????????????????????????????????????????????????????????????????????????????????????????????????????????????????????????????????????????????????????????????????????????????????????????????????????????????????????????????????????????????????????????????????????????????????????????????????????????????????????????????????????????????????????????????????????????????????????????????????????????????????????????????????????????????????????????????????????????????????????????????????????????????????????????????????????????????????????????????????????????????????????????-Nodalla   01110000000?0000000001???10100100000010011110101001?0001?0100101000010111110000201111110211000020101011------------------------------------------------------------------------------------------------------------------------------------------------------------------------------------------------------------------------------------------------------------------------------------------------------------------------------------------------------------------------------------------------------------------------------------------------------------------------------------------------------------------------------------------------------------------------------------------------------------------------------------------------------------------------------------------------------------------------------------------------------------------------------------------------------------------------------------------------------------------------------------------------------------------------------------------------------------------------------------------------------------------------------------------------------------------------------------------------------------------------------------------------------------------------------------------------------------------------------------------------------------------------------------------------------------------------------------------------------------------------------------------------------------------------------------------------------------------------------------------------------------------------------------------------------------------------------------------------------------------------------------------------------------------------------------------------------------------------------------------------------------------------------------------------------------------------------------------------------------------------------------------------------------------------------------------------------------------------------------------------------------------------------------------------------------------------------------------------------------------------------------------------------------------------------------------------------------------------------------------------------------------------------------------------------------------------------------------------------------------------------------------------------------------------------------------------------------------------------------------------------------------------------------------------------------------------------------------------------------------------------------------------------------------------------------------------------------------------------------------------------------------------------------------------------------------------------------------------------------------------------------------------------------------------------------------------------------------------------------------------------------------------------------------------------------------------------------------------------------------------------------------------------------------------------------------------------------------------------------------------------------------------------------------------------------------------------------------------------------------------------------------------------------------------------------------------------------------------------------------------------------------------------------------------------------------------------------------------------------------------------------------------------------------------------------------------------------------------------------------------------------------------------------------------------------------------------------------------------------------------------------------------------------------------------------------------------------------------------------------------------------------------------------------------------------------------------------------------------------------------------------------------------------------------------------------------------------------------------------------------------------------------------------------------------------------------------------------------------------------------------------------------------------------------------------------------------------------------------------------------------------------------------------------------------------------------------------------------------------------------------------------------------------------------------------------------------------------------------------------------------------------------------------------------------------------------------------------------------------------------------------------------------------------------------------------------------------------------------------------------------------------------------------------------------------------------------------------------------------------------------------------------------------------------------------------------------------------------------------------------------------------------------------------------------------------------------------------------------------------------------------------------------------------------------------------------------------------------------------------------------------------------------------------------------------------------------------------------------------------------------------------------------------------------------------------------------------------------------------------------------------------------------------------------------------------------------------------------------------------------------------------------------------------------------------------------------------------------------------------------------------------------------------------------------------------------------------------------------------------------------------------------------Nosybus   011?0000000000000100?1???00001101100110011111011000?0?010101000100001120121100021??121002110000001?1011------------------------------------------------------------------------------------------------------------------------------------------------------------------------------------------------------------------------------------------------------------------------------------------------------------------------------------------------------------------------------------------------------------------------------------------------------------------------------------------------------------------------------------------------------------------------------------------------------------------------------------------------------------------------------------------------------------------------------------------------------------------------------------------------------------------------------------------------------------------------------------------------------------------------------------------------------------------------------------------------------------------------------------------------------------------------------------------------------------------------------------------------------------------------------------------------------------------------------------------------------------------------------------------------------------------------------------------------------------------------------------------------------------------------------------------------------------------------------------------------------------------------------------------------------------------------------------------------------------------------------------------------------------------------------------------------------------------------------------------------------------------------------------------------------------------------------------------------------------------------------------------------------------------------------------------------------------------------------------------------------------------------------------------------------------------------------------------------------------------------------------------------------------------------------------------------------------------------------------------------------------------------------------------------------------------------------------------------------------------------------------------------------------------------------------------------------------------------------------------------------------------------------------------------------------------------------------------------------------------------------------------------------------------------------------------------------------------------------------------------------------------------------------------------------------------------------------------------------------------------------------------------------------------------------------------------------------------------------------------------------------------------------------------------------------------------------------------------------------------------------------------------------------------------------------------------------------------------------------------------------------------------------------------------------------------------------------------------------------------------------------------------------------------------------------------------------------------------------------------------------------------------------------------------------------------------------------------------------------------------------------------------------------------------------------------------------------------------------------------------------------------------------------------------------------------------------------------------------------------------------------------------------------------------------------------------------------------------------------------------------------------------------------------------------------------------------------------------------------------------------------------------------------------------------------------------------------------------------------------------------------------------------------------------------------------------------------------------------------------------------------------------------------------------------------------------------------------------------------------------------------------------------------------------------------------------------------------------------------------------------------------------------------------------------------------------------------------------------------------------------------------------------------------------------------------------------------------------------------------------------------------------------------------------------------------------------------------------------------------------------------------------------------------------------------------------------------------------------------------------------------------------------------------------------------------------------------------------------------------------------------------------------------------------------------------------------------------------------------------------------------------------------------------------------------------------------------------------------------------------------------------------------------------------------------------------------------------------------------------------------------------------------------------------------------------------------------------------------------------------------------------------------------------------------------------------------------------------------------------------------------------------------------------------------------------------------------------------------------------------------------------------------------------------------------------------------------------------------------------------------------------------------Nyrma   011010000???000?00001????0000101111--00000000101000?0?010101110100010001011000001-2110001010000011?1011------------------------------------------------------------------------------------------------------------------------------------------------------------------------------------------------------------------------------------------------------------------------------------------------------------------------------------------------------------------------------------------------------------------------------------------------------------------------------------------------------------------------------------------------------------------------------------------------------------------------------------------------------------------------------------------------------------------------------------------------------------------------------------------------------------------------------------------------------------------------------------------------------------------------------------------------------------------------------------------------------------------------------------------------------------------------------------------------------------------------------------------------------------------------------------------------------------------------------------------------------------------------------------------------------------------------------------------------------------------------------------------------------------------------------------------------------------------------------------------------------------------------------------------------------------------------------------------------------------------------------------------------------------------------------------------------------------------------------------------------------------------------------------------------------------------------------------------------------------------------------------------------------------------------------------------------------------------------------------------------------------------------------------------------------------------------------------------------------------------------------------------------------------------------------------------------------------------------------------------------------------------------------------------------------------------------------------------------------------------------------------------------------------------------------------------------------------------------------------------------------------------------------------------------------------------------------------------------------------------------------------------------------------------------------------------------------------------------------------------------------------------------------------------------------------------------------------------------------------------------------------------------------------------------------------------------------------------------------------------------------------------------------------------------------------------------------------------------------------------------------------------------------------------------------------------------------------------------------------------------------------------------------------------------------------------------------------------------------------------------------------------------------------------------------------------------------------------------------------------------------------------------------------------------------------------------------------------------------------------------------------------------------------------------------------------------------------------------------------------------------------------------------------------------------------------------------------------------------------------------------------------------------------------------------------------------------------------------------------------------------------------------------------------------------------------------------------------------------------------------------------------------------------------------------------------------------------------------------------------------------------------------------------------------------------------------------------------------------------------------------------------------------------------------------------------------------------------------------------------------------------------------------------------------------------------------------------------------------------------------------------------------------------------------------------------------------------------------------------------------------------------------------------------------------------------------------------------------------------------------------------------------------------------------------------------------------------------------------------------------------------------------------------------------------------------------------------------------------------------------------------------------------------------------------------------------------------------------------------------------------------------------------------------------------------------------------------------------------------------------------------------------------------------------------------------------------------------------------------------------------------------------------------------------------------------------------------------------------------------------------------------------------------------------------------------------------------------------------------------------------------------------------------------------------------------------------------------------------------------------------------------------------------------------------------------------------------------------------------------------------------------------------------------------------------------------------------------------------------------------------------------------------Ormiscocerus   0110?000000000000000??????000211111--000?000010100000?01?101110100010020020000001-2???002??0000011?0011???????????????????????????????????????????????????????????????????????????????????????????????????????????????????????????????????????????????????????????????????????????????????????????????????????????????????????????????????????????????????????????????????????????????????????????????????????????????????????????????????????????????????????????????????????????????????????????????????????????????????????????????????????????????????????????????????????????????????????????????????????????????????????????????????????????????????????????????????????????????????????????????????????????????????????????????????????????????????????????????????????????????????????????????????????????????????????????????????????????????????????????????????????????????????????????????????????????????????????????????????????????????????????????????????????????????????????????????????????????????????????????????????????????????????????????????????????????????????????????????????????????????????????????????????????????????????????????????????????????????????????????????????????????????????????????????????????????????????????????????????????????????????????????????????????????????????????????????????????????????????????????????????????????????????????????????????????????????????????????????????????????????AATTACCCACTCCCGGCACGGGGAGGTAGTGACGAAAAATAACGATACGGGACTCATCCGAGGCCCCGTAATCGGAATGAGTACACTTTAAATCCTTTAACGAGGATCCATTGGAGGGCAAGTCTGGTGCCAGCAGCCGCGGTAATTCCAGCTCCAATAGCGTATATTAAAGTTGTTGCGGTTAAAAAGCTCGTAGTCGAATCTGTGTCCCACACTGTCGGTTCACCGCTCGCGGTGTTCAACTGGCATGTTATGTGGGACGTCCTACCGGTGGGTGGTGTGAATTATTGTTTTTGTTTC-TGTATGATATTGTGTGTGTATGTGTATTATAT--TACA------------TTAAA-----------------------TGTGTATTTTACTACT---GCATTATGCATATTGTATGGTGCAATTATATTTAATAATTTGCCG-TAAGTGTTTAGCCGTGAAG-AACGGCGGCAGCCCCCAATTGCAATCCCGTCGCGGTGCTCTTAACTGAGTGTCGAGGTGGGCCGGTACGTTTACTTTGAACAAATTAGAGTGCTTAAAGCAGGCTAAAATTTCGCCTGAATATTGTGTGCATGGAATAATAAAATAGGATCTCGGTTCTATTTTGTTGGTTTTCGGAACTCCGAGATAATGATTAATACGGACAGATGGGGGCATTCGTATTGCGACGTTAGAGGTGAAATTCTTGGATCGTCGCAAGACGGACAGAAGCGAAAGCATTTGCCAAAAATGTTTTGATTGATCAAGAACGAAAGTTAGAGGTTCGAAGGCGATCAGATACCGCCCTAGTTCTAACCATAAACGATGCCAGCTAGCGATCCGCCGAAGTTCCTCCGATGACTCGGCGGGCAGCTT---CCGGGAAACCAAAGCTTTTGGGTTCCGGGGGAAGTATGGTTGCAAAGCTGAAACTTAAAGGAATTGACGGAAGGGCACCACCAGGAGTGGAGCCTGCGGCTTAATTTGACTCAACACGGGAAACCTCACCAGGCCCGGACACCGTTAGGATTGACAGATTGAGAGCTCTTTCTTGATTCGGTGGGTGGTGGTGCATGGCCGT-CTTAG----------------------------------------------------------------------------------------------------------------------------------------------------------------------------------------------------------------------------------------------------------------------------------------------------------------------------------------------------------------------------------------------------------------------------------------------------------------------------------------------------------------------------------------------------------------------------------------------------------------------------------------------------------------------------------------------------------------------------------------------------------------------------------------------------------------------------------------------------------------------------------------------------------------------------------------------------------------------------------------------------------------------------------------------------------------------------------------------------------------------------------------------------------------------------------------------------------------------------------------------------------------------------------------------------------------------------------------??????????????????????????????????????????????????????????????????????????????????????????????????????????????????????????????????????????????????????????????????????????????????????????????????????????????????????????????????????????????????????????????????????????????????????????????????????????????????????????????????????????????????????????????????????????????????????????????????????????????????????????????????????????????????????????????????????????????????????????????????????????????????????????????????????????????????????????????????????????????????????????????????????????????????????????????????????????????????????????????????????????????????????????????????????????????????????????????????????????????????????????????????????????????????????????????????????????????????????????????????????????????????????????????????????????????????????????????????????????????????????????????????????????????????????????????????????????????????????????????????????????????????????????????????????????????????????????????????????????????????????????????????????????????????????????????????????????????????????????????????????????????????????????????????????????????????????????????????????????????????????????????????????????????????????????????????????????????????????????????????????????????????????????????????????????????????????????????????????????????????????????????????????????????????????????????????????????????????????????????????????????????????????????????????????????????????????????????????????????????????????????????????????????????????????????????????????????????????????????????????????????????????????????????????????????????????????????????????????????????????????????????????????????????????????????????????????????????????????????????????????????????????????????????????????????????????????????????????????????????????????????????????????????????????????????????????????????????????????????????????????????????????????????????????????????????????????????????????????????????????????????????????????????????????????????????????????????????????????????????????????????????????????????????????????????????????????????????????????????????????????????????????????????????????????????????????????????????????????????????-Podallea   1111010000000000010011101101001000010100011101010001010100100101000011201210001201112010211000011100011--CTACTTCAGCCATCTTACCAAAATGAATATTTTCAACTAACCATAAAGATATTGGAA-CTTTATATTTTTTATTTGGAATTTGATCAGGAATAGTAGGATCTTCTTTAAGTATTTTAATTCGTGCAGAATTAGGGCACTCAGGAGCTTTAATTGGAGACGACCAAATTTATAAT-GTAATTGTAACAGCTCACGCTTTTATTATAATTTTTTTTACTGTTATACCCATTTTAATTGGTGGATTTGGAAATTGACTAGTACCTTTAATATTAGGAGCCCCTGATATAGCATTTCCCCGAATAAATAATATAAGATTCTGATTATTACCCCCTTCATTATTATTATTATTAAGAAGATCTATCGTAGAAAGAGGGGCGGGGACAGGTTGAACTGTCTATCCTCCTTTATCAAGAAATATTGCCCACTCCGGCGCCTCCGTAGATTTAGCTATTTTTAGATTACATTTAGCAGGAGTTTCATCAATTTTAGGAGCAATTAATTTTATTACTACAGTATTAAATATACGCCCAGCAGGTATAACACTAGATCGAATACCTTTATTTGTATGATCTGTTGTAATTACAGCTCTTCTATTACTTCTTTCTTTACCTGTACTAGCTGGAGCTATTACTATACTATTAACTGACCGAAATTTTAATACTTCTTTTTTTGACCCCGCGGGTGGGGGAGACCCCTTACTTTATCAACATCTATTTTGAT-TTTTGGTCATCCCGAAGT-----------GCGCCTGTTTATCAAAAACATGTCTTTTT-GAAAATAATAAAAAGTCTAACCTGCCCACTGAAAATAT-TTTAAAGGGCCGCGGTATATTGACTGTGCAAAGGTAGCATAATCATTTGTCTCTTAATTGGAGGCTAGAATGAATGGTTGAATGAAGTATTAACTGTCTCATTTTAATAAATATAAAAATTTAATTTTT-AGTTAAAAATCTAAAATTTATTTAGAGGACGAGAAGACCCTATAAATCTTTAT--TTTAT-TAATTATTTTAAATTTTAGAATATAATTATTTATTATAATTGAT--AAAATTTTGTTGGGGTGACAGGAAAATTTAATAAACTTTTTTTA--ATATAAA--CATAAATTTATGATTAATTGATCCAATTTTATTGATTAAAAGATTAAGATACTTTAGGGATAACAGCGTA-ATTTTTTTAGAGAGTTCTTATCGATAAAAAAGATTGCGACCTCGATGTTGAATTAAAGAT-AGTTTTAGATGTAGCAGTTTAA-TAATTAGGTCTGTTCGACCTTTAAATCT----------------------------------------TCTGCCAGTAGTCATATGCTTGTCTCAAAGATTAAGCCATGCATGTCTCAGTACAAGCCAAATTAAGGTGAAACCGCGAAAGGCTCATTATATCAGTTATGGTTCCTTAGATCGTACCCACATTTACTTGGATAACTGTGGT-----AATTCTAGAGCTAATACATG---CAAACAG--AGTTC-CGACCAGAGATGGAAGGAATGCTTTTATTAGATCAAAACCAATCGATGTT--------------------------GTCATATTTTTTGACATTTAT-TTGTTAAT--------------------------------------------------------TAAATATG----TT------GCATCGTTCTATTTGGTGACTCTGAATAACTTTAAGCTGATCGCACGGTCTC------GTACCGGCGACGCATCTTTCAAAT---------GTCTGCCTTATCAACTGTCGATGGTAGGTTCTGCGCCTACCATGG-----TTGTAACGGGTAACGGGGAATCAGGGTTCG-------ATTCCGGAGAGGGAGCCTGAGAAACGGCTACCACATCCAA------GGAAGGCAGCAGGCGCGCAAATTACCCACTCCCGGCACGGGGAGGTA--------GTGACG--AAAAATAACGATACGGGACTCATCCGAGGCCCCGTAATCGGAATGAGTACACTTTAAATCCTTTAACA-AGGATCCATTAGAG--GGCAAGTCTGGTGCCAGCAG---CCGCGGTAATTCCAGCTCTAATAGCGTATATTAAAGTTGTTGCGGTTAAAAAGCTCGTAGTCGAATCTGTGTCTTACACTGTCGGTTCACCGCTCGCGGTGTCCAACTGGCATGTCA-----------------------------------------------------------------------------------------------------------------------------------------------------------------------------------------------------------------------------------------------------------------------------------------------------------------------------------------------------------------------------------------------------------------------------------------------------------------------------------------------------------------------------------------------------------------------------------------------------------------------------------------------------------------------------------------------------------------------------------------------------------------------------------------------------------------------------------------------------------------------------------------------------------------------------------------------------------------------------------------------------------------------------------------------------------------------------------------------------------------------------------------------------------------------------------------------------------------------------------------------------------------------------------------------------------------------------------------------------------------------------------------------------------------------------------------------------------------------------------------------------------------TGCATCCATCATGG-CACAAACCGTTGCTACATGACGTCACAGAACCATGGATTCGCCGTTGATCCAACTACTAAACCCAGAGATTGGGAAGTTTTGTTCACCAACGCAAACGATGGATCAAATGAAGGAATCGTTCATACGCAACAACCATACTTCAGTGTTCAATTCCATCCGGAACACACTGCTGGTCCAGAAGACCTTGAATGCCTCTTTGATATCTTCTTAGATTCCGTCAAAGCTTACAAAGCCAATAAATCGTATTGTGTTCGTGAACAGCTTACAAAATGCTTAACTTACAATC---GTCCAAATTCCACTAAAATAAACATTCCAAAGAAGGTGCTGATCATCGGTTCCGGTGGGTTATCCATCGGCCAAGCTGGTGAATTCGATTACTCAGGCTCACAAGCGATCAAAGCTTTAAAAGAAGAGCACATACAAACAATTTTAATGAATCCAAATATTGCAACGGTGCAAACTTCCAAAGGTCTCGCGGATAAAGTTTACTTTCTGCCTTTGATGCCGGAATACGTTGAGCAAGTGATTCGTTCTGAGCGTCCAGATGGCGTACTTCTAACATTCGGCGGTCAAACCGCATTAAATTGCGGCGTAGAACTCGAACAAGCGGGAATATTCAAAAAATATGGAGTTCAAATTTTAGGAACACCCATCAAATCGATCATCGAAACAGAAGATAGAAAAATATTCGCAGACCGCGTGAATGAGATTGGTGAGAAAGTGGCGCCCAGTGCTGCTGTTTACTCCGTGGAGGAAGCTTTGAAAGCTGCTGAGACAATTGGATATCCAGTAATGGCTCGTGCAGCGTTCTCCTTAGGAGGGTTAGGTTCTGGTTTCGCTAATAACTCCGACGAATTGAAAATTTTGGCGACACAAGCTTTGGCCCATTCAAGTCAGTTAATCATCGATAAGTCGTTGAGGGGTTGGAAAGAAGTTGAATATGAAGTTGTCCGCGATGCGTTTGATAACTGTATCACTGTTTGCAACATGGAGAATGTCGATCCTTTGGGAATTCACACTGGTGAGTCCATTGTCGTTGCGCCCAGTCAAACCCTTTCCAATCGGGAATACAACATGTTGCGAACAACCGCGATTAAAGTGATTCGTCACTTTGGTGTCGTTGGAGAATGCAACATCCAATACGCTTTGAATCCCCATTCCGAAGAATTTTACATAATTGAAGTCAACGCTCGTTTGTCACGAAGTTCAGCTTTAGCCAGTAAAGCAACAGGATACCCATTAGCTTACGTTGCTGCTAAATTAGCATTAGGAAACTCACTCCTTGATATCAAGAATTCCGTAACGGGCAAAACAACAGCATGTTTTGAGCCCAGTTTAGATTACTGCGTTGTAAAAATTCCACGCTGGGATTTGAGTAAATTCAGCCGTGTGAGTTCAAAAATTGGCAGTTCAA-TGAAAAGTGTTGGTGAAGTAATGGCGATTGGTCGTAAATTCGAGGAAGCGTTTCAAAAAGCTTTACGTATGGTGGATGAGAACGTTTCCGGCT-------TTAATCCCAATTTGCAGAAAATTAACGACGAGGATTTAAAAGAACCCACAGAT----AAACGAATGTTCGTTATTGCTACGTCATTGAAAGCGGGTTACTCCGTTGAAAAACTCCACGAGTTAACAAAAATTGATCCTTGGTTCTTGCAGAAGATGAAGAATATCATTGACTTGCATACAATGCTTACTGCTTT----AGATCAGCAGGGAA---TAACGCGTTCGATTCTCCTTCAAGCTAAATGCTATGGATTCTCAGATAAGCAGATCGCCAATGCTGTTGGGAGCACGGAATTAGCGATCCGCAAACATCGACAGGAATGCGGGGTTTTACCATTCGTGAAGCAGATTGATACAGTCGCCGCCGAATGGCCAGCATGCACCAACTACTTATACTTGACGTA------CAACG-CGAGTGCTCACGATATCGAATATCCTGGAGGTTACATCATGGTCATTGGCTCTGGG--GTCTATCGAATTGGAAGTTCCGTGGAGTTCGA--TTGGTGC-----GCTGTTG-GTTCTCTACGCGAACTGCAGAAACTTGGAAAG--------AAAACAATCATGGTGAACTACAATCCAGAAACTGTTAGTACGGATTACGATATGTGCGATCGTT-TGTATTTTGAAGAGATTTCCTTCGAAAGAGT-TAGGG-ATAGGTACAACT---------------------Protobiella   ?110?0000???000?0000??10?0000110000100000011011100000001?0000101000011000110000?1??111002110110011?0011------------------------------------------------------------------------------------------------------------------------------------------------------------------------------------------------------------------------------------------------------------------------------------------------------------------------------------------------------------------------------------------------------------------------------------------------------------------------------------------------------------------------------------------------------------------------------------------------------------------------------------------------------------------------------------------------------------------------------------------------------------------------------------------------------------------------------------------------------------------------------------------------------------------------------------------------------------------------------------------------------------------------------------------------------------------------------------------------------------------------------------------------------------------------------------------------------------------------------------------------------------------------------------------------------------------------------------------------------------------------------------------------------------------------------------------------------------------------------------------------------------------------------------------------------------------------------------------------------------------------------------------------------------------------------------------------------------------------------------------------------------------------------------------------------------------------------------------------------------------------------------------------------------------------------------------------------------------------------------------------------------------------------------------------------------------------------------------------------------------------------------------------------------------------------------------------------------------------------------------------------------------------------------------------------------------------------------------------------------------------------------------------------------------------------------------------------------------------------------------------------------------------------------------------------------------------------------------------------------------------------------------------------------------------------------------------------------------------------------------------------------------------------------------------------------------------------------------------------------------------------------------------------------------------------------------------------------------------------------------------------------------------------------------------------------------------------------------------------------------------------------------------------------------------------------------------------------------------------------------------------------------------------------------------------------------------------------------------------------------------------------------------------------------------------------------------------------------------------------------------------------------------------------------------------------------------------------------------------------------------------------------------------------------------------------------------------------------------------------------------------------------------------------------------------------------------------------------------------------------------------------------------------------------------------------------------------------------------------------------------------------------------------------------------------------------------------------------------------------------------------------------------------------------------------------------------------------------------------------------------------------------------------------------------------------------------------------------------------------------------------------------------------------------------------------------------------------------------------------------------------------------------------------------------------------------------------------------------------------------------------------------------------------------------------------------------------------------------------------------------------------------------------------------------------------------------------------------------------------------------------------------------------------------------------------------------------------------------------------------------------------------------------------------------------------------------------------------------------------------------------------------------------------------------------------------------------------------------------------------------------------------------------------------------------------------------------------------------------------------------------------------------------------------------------------------------------------------------------------------------------------------------------------------------------------------------------------------------------------------------------------------------------------------------------------------------------------------------------------------------------------------------------------------------------------------------------------------------------------------------------------------------------------------------------------------------------------------------------------------------------------------------------------------------------------------------------------------------------------------------------------------------------Quasispermophorella   0110000000??000?0?000????0010210001--0101011010100110001?1100101000010001211101201112011311000020101011---------------ACGCGACAATGTATTATTCTCAACTAACCATAAAGATATTGGAAACCTTATATTTTGTATTCGGTGTATGATCAGGCCTTATTGGAACAAGTTTAAGACTTCTAATTCGTGCTGAACTCGGTCAACCAGGATCACTTATTGGTGATGACCAAATTTACAAC-GTAATTGTTACAGCTCATGCTTTCGTAATAATTTTTTTCATAGTAATACCTATTATAATTGGAGGATTTGGTAACTGATTGGTACCATTAATATTAGCTGCACCAGATATAGCATTCCCTCGAATAAATAACATAAGATTTTGATTATTACCCCCATCATTAACCTTACTATTAGCTTCAAGAATTGTAGAAAGTGGAGCTGGGACAGGATGAACTGTTTATCCCCCCCTCTCTTCAGGTATTGCTCATGCTGGAGCATCAGTTGATCTAGCCATTTTTAGTCTTCACCTAGCGGGTGTATCATCAATTCTTGGTGCAGTAAATTTTATTACAACAGTAATTAATATACGCTTATCATACATAACACTTGACCGAATACCTTTATTTGTGTGATCTGTAGTTATTACAGCTATTCTTCTATTACTCTCTTTACCAGTATTAGCTGGAGCTATTACCATATTATTAACTGATCGAAACCTTAATACATCTTTCTTTGACCCCGCGGGAGGAGGAGACCCTATTTTATACCAACATTTATTCTGATTTTTTGGACATCCAGAAGTTATATTTAATT-----TGTTTATCAAAAACATGTCTTTAT-GAAAATAATATAAAGTCTAACCTGCCCACTGAAATTTTATTTAAAGGGCCGCGGTATTTTGACTGTGCAAAGGTAGCATAATCATTTGTCTTTTAATTGAAGGCTGGAATGAATGGTTGAATGAAGTATTAACTGTCTCATTTTAATAAATGTAAAAATTTAATTTTTTAGTCAAAAAGCTAAAATTTAATTGAAGGACGAGAAGACCCTATAAATCTTTAT--TTTATATTATTAAATTAAATTTTAGATAAAAATTATTTATTATAATATAT--AAAATTTTATTGGGGTGATAGGAAAATTTAATAAACTTTTTTT---ATTTTAA-CTATTGATTTATAAATATTTGATCCAATATTGTTGATTAAAAGATTAAGATACTTTAGGGATAACAGCGTA-ATTTTTTTAGAGAGTTCTTATCGATAAAAAAGATTGCGACCTCGATGTTGAATTAAAGATTAATATTAAATGTAGAAGTTTAA-ATATTAGGTCTGTTCGACCTTTAAATCT----------------------------------GTTGATCCTGCCAGTAGTCATATGCTTGTCTCAAAGATTAAGCCATGCATGTCTCAGTACAAGCCAAATTAAGGTGAAACCGCGAAAGGCTCATTATATCAGTTATGGTTCCTTAGATCGTACCCACATTTACTTGGATAACTGTGGT-----AATTCTAGAGCTAATACATG---CAAACAG--AGTTC-CAACCAGAGATGGAAGGAATGCTTTTATTAGATCAAAACCAATCGATGTTG------------------------TCATATTTGTATATTGGTTTTCCAATATAT---------------------------------------------------------TTGATATG----GT------GCATCGTTCTATTTGGTGACTCTGAATAACTTTAAGCTGATCGCACGGTCTC------GTACCGGCGACGCATCTTTCAAAT---------GTCTGCCTTATCAACTGTCGATGGTAGGTTCTGCGCCTACCATGG-----TTGTAACGGGTAACGGGGAATCAGGGTTCG-------ATTCCGGAGAGGGAGCCTGAGAAACGGCTACCACATCCAA------GGAAGGCAGCAGGCGCGCAAATTACCCACTCCCGGCACGGGGAGGTA--------GTGACG--AAAAATAACGATACGGGACTCATCCGAGGCCCCGTAATCGGAATGAGTACACTTTAAATCCTTTAACG-AGGATCCATTAGAG--GGCAAGTCTGGTGCCAGCAG---CCGCGGTATTTCCAGCTCTAATAGCGTATATTAAAGTTGTTGCGGTTAAAAAGCTCGTAGTCGAATCTGTGTCTTACACTGTCGGTTCACCGCTCGCGGTGTTCAACTGGCATGTTATGTGAGACGTCCTACCGGTGGGTGGTGTAAATTATTGTTATTATTTTTGCCCACTGTATATTGTTGGATATGCACCAATGCCAGTTATATATTTATCTTGGATTGTTATGTATGTTAATATGCTTTTCAGTATCGGCTTTGCACATCAAAAGGTGACAGTGTTCAAAATTTATATATCCATTTCTCTTTTCGGATCTCTATATATTGAAATTTTTTTTTTGATATATTTGGGATTTGATTGAGTTTTTTGGTTTATTTATTTTGCCTGTCGCTGTGTTTAAAGTGACAAGATTTGATAATGGCATATACATATTATATTCAGTCCGTTTATATGGGATGATATATTGACTGCTTGGATTAAAAATGTATATTTTTACGTATATATAGTGTTCTGGTTGGATAATTAACATAAAAATAATTTGCCGTAAGTGTTTAGCCGTGAAGAACGGCGACAGCCCCCAATTGCAATCCTGTCGCGGTGCTCTTAATTGAGTGTCGAGGTGGGCCGGTACGTTTACTTTGAACAAATTAGAGTGCTTAAGGCAGGTTAAAATTTCGCCTGAATATTGTGTGCATGGAATAATGGAATAGGACCTCGGTTCTATTTTGTTGGTTTTCGGAACTCCGAGGTAATGATTAATACGGACAGATGGGGGCATTCGTATTGCGACGTTAGAGGTGAAATTCTTGGATCGTCGCAAGACGGACAGAAGCGAAAGCATTTGCCAAAAATGTTTTGATTGATCAAGAACGAAAGTTAGAGGTTCGAAGGCGATCAGATACCGCCCTAGTTCTAACCATAAACGATGCCAGCTAGCGATCCGCCGAAGTTCTTCCGATGACTCGGCGGGCAGCTTCCGGGAAACCAAAGCTTTTGGGTTCCGGGGGAAGTATGGTTGCAAAGCTGAAACTTAAAGGAATTGACGGAAGGGCACCACCAGGAGTGGAGCCTGCGGCTTAATTTGACTCAACACGGGAAACCTCACCAGGCCCGGACACCGGAAGGATTGACAGATTGAGAGCTCTTTCTTGATTCGGTGGGTGGTGGTGCATGGCCGTTCTTAGTTGGTGGAGCGATTTGTCTGGTTAATTCCGATAACGAACGAGACTGTAGCCTGCTAAATAGACGTACCTTACTGGCATCTCAAGGCCCATCGGCTGTAATGTATCGTTACGTTTGTTGCTTACTTTTAAATAAATTTTACTCTTATTATTGTATGGGCCTCTCTATTTTACATATTCGCTATATATGTATATGCATATATGTTTCTGATGTATATGTGTGTGCGTGTGTCGCGGTGTGTATTTATAGGGATGTGTTTTATATGATGATAGGGTATTTTCTATTGGGGTTTGTTAACACGTGCGGTGTACAGTTTTTTGATGTGGTTTTTACTGCCGGCGTACATATTAATCTTCTTAGAGGGACAGGCGGGTTTTAGCCGCACGAAGATTGA-GTGTCCATCACGGTAACAAAACGTTGCTATATGACGTCACAGAATCACGGTTTTGCAGTGGACGTTGAAACATTACCTAAAGAGTGGGAGGCTTTATTCACCAATGCCAATGATAAAACAAATGAAGGAATCATTCACACAAAATTACCATATTTTAGTGTACAGTTTCATCCAGAACATACTGCTGGTCCGGAGGACTTAGAGTCTCTTTTTGACGTGTTTTTAAACTCCGTTAAATCATATATTTCCAAAGAACCTTTCTGTGTTAAAGACGAGCTTATTAAACGATTAACGTATGATT---GTTCACATTCCACACCAATAACCATTCCTAAGAAGGTTCTCATTATCGGTTCTGGTGGATTATCCATTGGTCAAGCTGGTGAATTTGATTACTCTGGATCACAAGCCATCAAAGCATTGAAAGAGGAAAATATACAAACGATTTTAATGAATCCAAATATCGCTACAGTTCAGACGTCTAAAGGGCTCGCGGATAAAGTCTATTTTCTTCCTTTAATTCCTGAATATGTAGAGCAAGTTATCCGCTCTGAGCGTCCAGACGGGGTACTGTTAACTTTTGGGGGTCAAACAGCGTTAAACTGTGGTGTTGAACTCGAACAAGCTGGTGTATTCAAAAAATATGGCGTTCAAATATTAGGTACGCCAATTAAATCGATAATCGAAACTGAAGATCGGAAAATATTCGCTGAACGTGTAAATGAGATTGGTGAGAAAGTTGCACCCAGTGCTGCTGTTTACTCAGTTCAGGAAGCTTTAAACGCCGCTGAGGTGATTGGATATCCCGTGATGGCCCGTGCTGCGTTTTCTCTCGGAGGTTTAGGTTCTGGTTTTGCTAATAACGCCGATGAATTGAAGATACTTGCGTCACAAGCTTTAGCACATTCGAATCAGTTGATTATTGATAAGTCGTTGCGTGGCTGGAAAGAGGTGGAGTATGAAGTAGTACGTGATGCTTTTGACAACTGCATCACCGTTTGTAATATGGAGAATGTTGATCCTTTGGGAATCCATACTGGTGAGTCGATTGTCGTTGCTCCTAGTCAAACGCTTTCAAACCGGGAATATAATATGCTGCGAACAACAGCGATTAATGTGATCCGACACTTTGGTGTCGTTGGTGAATGTAACATTCAATATGCTTTGAATCCTCACTCAGAGGAATACTACATTATTGAAGTGAATGCACGTTTATCAAGAAGTTCTGCGTTAGCAAGTAAAGCGACAGGTTATCCCTTGGCCTACGTTGCTGCTAAATTAGCATTAGGTTCATCCCTTCTCGACATCAAGAACTCCGTTACTGGTGAAACAACAGCTTGTTTCGAGCCTAGTTTAGATTATTGTGTCGTAAAAATACCACGTTGGGATTTAAGTAAATTCAGTCGTGTAAGTTCAAAAATTGGAAGTTCTA-TGAAAAGTGTTGGTGAAGTAATGGCGATTGGTCGTAAATTTGAAGAAGCGTTTCAAAAAGCGTTACGTATGGTCGATGAAAACGTTTCCGGTT-------TCGATCCTAATTTACAAAAGATCAATGATGAAGATCTAAAAGAACCAACCGAC----AAACGAATGTTTGTCATTGCCGCTTCATTAAAAGCCGGTTACACTGTTGAAAAACTTCACGAGCTAACAAAAATTGATCCATGGTTCTTACAGAAGATGAAAAATATCATTGATTTACATACGATGTTAACCGCGTT----GGATCAACAAGGTG---TCACACGGTCTATTCTCTATCAAGCGAAATGTTATGGTTTCTCAGATAAGCAAATCGCTAATGCTGTTGCGAGCACGGAGTTAGCTATACGGTTACACCGACAAGAGTGTGGTGTCTTACCGTACGTGAAACAGATTGACACGGTTGCAGCTGAATGGCCAGCTTGTACTAACTACTTGTACTTAACGTA------TAACG-CTAATTCTCATGATATAGAATACCCTGGTGGGTATATTATGGTGATTGGATCCGGT--GTTTATCGTATTGGAAGTTCAGTGGAGTTCGA--TTGGTGC-----GCTGTTG-GTTGTCTGAGAGAACTACGCAAACTTGGAAAG--------AAAACCATTATGGTTAACTATAACCCGGAAACCGTTAGTACTGATTACGATAAGAGTGATCGTC-TATATTTTGAAGAAATCTCCTTCGAAGTGGT-AATGG-ATATCTATAACCTCGAAAATCCA----------Speleoberotha   01101000000000000100010000001011110011200011111101000001?1011000000112201101000011011000000100001110011------------------------------------------------------------------------------------------------------------------------------------------------------------------------------------------------------------------------------------------------------------------------------------------------------------------------------------------------------------------------------------------------------------------------------------------------------------------------------------------------------------------------------------------------------------------------------------------------------------------------------------------------------------------------------------------------------------------------------------------------------------------------------------------------------------------------------------------------------------------------------------------------------------------------------------------------------------------------------------------------------------------------------------------------------------------------------------------------------------------------------------------------------------------------------------------------------------------------------------------------------------------------------------------------------------------------------------------------------------------------------------------------------------------------------------------------------------------------------------------------------------------------------------------------------------------------------------------------------------------------------------------------------------------------------------------------------------------------------------------------------------------------------------------------------------------------------------------------------------------------------------------------------------------------------------------------------------------------------------------------------------------------------------------------------------------------------------------------------------------------------------------------------------------------------------------------------------------------------------------------------------------------------------------------------------------------------------------------------------------------------------------------------------------------------------------------------------------------------------------------------------------------------------------------------------------------------------------------------------------------------------------------------------------------------------------------------------------------------------------------------------------------------------------------------------------------------------------------------------------------------------------------------------------------------------------------------------------------------------------------------------------------------------------------------------------------------------------------------------------------------------------------------------------------------------------------------------------------------------------------------------------------------------------------------------------------------------------------------------------------------------------------------------------------------------------------------------------------------------------------------------------------------------------------------------------------------------------------------------------------------------------------------------------------------------------------------------------------------------------------------------------------------------------------------------------------------------------------------------------------------------------------------------------------------------------------------------------------------------------------------------------------------------------------------------------------------------------------------------------------------------------------------------------------------------------------------------------------------------------------------------------------------------------------------------------------------------------------------------------------------------------------------------------------------------------------------------------------------------------------------------------------------------------------------------------------------------------------------------------------------------------------------------------------------------------------------------------------------------------------------------------------------------------------------------------------------------------------------------------------------------------------------------------------------------------------------------------------------------------------------------------------------------------------------------------------------------------------------------------------------------------------------------------------------------------------------------------------------------------------------------------------------------------------------------------------------------------------------------------------------------------------------------------------------------------------------------------------------------------------------------------------------------------------------------------------------------------------------------------------------------------------------------------------------------------------------------------------------------------------------------------------------------------------------------------------------------------------------------------------------------------------------------------------------------------------------------------------------------------------------------------------------------------------------------------------------------------------------------------------------------------------------Spermophorella   1110000000000000010010101100001000010100011001010010000100100101000011201210000201112011311000021101011------------------------------------------------------------------------------------------------------------------------------------------------------------------------------------------------------------------------------------------------------------------------------------------------------------------------------------------------------------------------------------------------------------------------------------------------------------------------------------------------------------------------------------------------------------------------------------------------------------------------------------------------------------------------------------------------------------------------------------------------------------------------------------------------------------------------------------------------------------------------------------------------------------------------------------------------------------------------------------------------------------------------------------------------------------------------------------------------------------------------------------------------------------------------------------------------------------------------------------------------------------------------------------------------------------------------------------------------------------------------------------------------------------------------------------------------------------------------------------------------------------------------------------------------------------------------------------------------------------------------------------------------------------------------------------------------------------------------------------------------------------------------------------------------------------------------------------------------------------------------------------------------------------------------------------------------------------------------------------------------------------------------------------------------------------------------------------------------------------------------------------------------------------------------------------------------------------------------------------------------------------------------------------------------------------------------------------------------------------------------------------------------------------------------------------------------------------------------------------------------------------------------------------------------------------------------------------------------------------------------------------------------------------------------------------------------------------------------------------------------------------------------------------------------------------------------------------------------------------------------------------------------------------------------------------------------------------------------------------------------------------------------------------------------------------------------------------------------------------------------------------------------------------------------------------------------------------------------------------------------------------------------------------------------------------------------------------------------------------------------------------------------------------------------------------------------------------------------------------------------------------------------------------------------------------------------------------------------------------------------------------------------------------------------------------------------------------------------------------------------------------------------------------------------------------------------------------------------------------------------------------------------------------------------------------------------------------------------------------------------------------------------------------------------------------------------------------------------------------------------------------------------------------------------------------------------------------------------------------------------------------------------------------------------------------------------------------------------------------------------------------------------------------------------------------------------------------------------------------------------------------------------------------------------------------------------------------------------------------------------------------------------------------------------------------------------------------------------------------------------------------------------------------------------------------------------------------------------------------------------------------------------------------------------------------------------------------------------------------------------------------------------------------------------------------------------------------------------------------------------------------------------------------------------------------------------------------------------------------------------------------------------------------------------------------------------------------------------------------------------------------------------------------------------------------------------------------------------------------------------------------------------------------------------------------------------------------------------------------------------------------------------------------------------------------------------------------------------------------------------------------------------------------------------------------------------------------------------------------------------------------------------------------------------------------------------------------------------------------------------------------------------------------------------------------------------------------------------------------------------------------------------------Spiroberotha   0111010000000000000011101101001000010120101000110000000100100100000011000210000110012011?11011021111011----------------------------------------------------------------------------------------------------------------------------------------------------------------------------------------------------------------------------------------------------------------------------------------------------------------------TAAGATTTTGATTTTTACCACCATCATTAACATTATTACTAGCTTCTTCCATAGTAGAAAGAGGAGCTGGAACAGGTTGAACTGTTTACCCACCTTTAGCTGCAGGAATTGCTCATGCAGGAGCTTCAGTTGATTTAGCTATTTTTAGTTTACATTTAGCAGGTGTATCTTCAATTCTTGGTGCTGTAAATTTCATTACCACAGTAATTAATATACGATTATCTTATATGACTCTAGATCGAATACCATTATTTTGTTGAGCTGTTGTAATTACAGCTTTATTACTTCTGCTTTCTTTACCTGTATTAGCTGGAGCAATTACAATACTTTTAACGGATCGTAATTTAAATACATCATTCTTTGACCCAGCAGGTGGGGGGGACCCAATTTTATACCAACATCTATTT----------------------------------??????????????????????????????????????????????????????????????????????????????????????????????????????????????????????????????????????????????????????????????????????????????????????????????????????????????????????????????????????????????????????????????????????????????????????????????????????????????????????????????????????????????????????????????????????????????????????????????????????????????????????????????????????????????????????????????????????????????????????????????????????????????????????????????????????????????????????????????????????????????????????????????????????????????????????????????????????????????????????????????????????????????????????????????????????????????????????????????????????????????????????????????????????????????????????????????????????????????????????????????????????????????????????????????????????????????????????????????????????????????????????????????????????????????????????????????????????????????????????????????????????????????????????????????????????????????????????????????????????????????????????????????????????????????????????????????????????????????????????????????????????????????????????????????????????????????????????????????????????????????????????????????????????????????????????????????????????????????????????????????????????????????????????????????????????????????????????????????????????????????????????????????????????????????????????????????????????????????????????????????????????????????????????????????????????????????????????????????????????????????????????????????????????????????????????????????????????????????????????????????????????????????????????????????????????????????????????????????????????????????????????????????????????????????????????????????????????????????????????????????????????????????????????????????????????????????????????????????????????????????????????????????????????????????????????????????????????????????????????????????????????????????????????????????????????????????????????????????????????????????????????????????????????????????????????????????????????????????????????????????????????????????????????????????????????????????????????????????????????????????????????????????????????????????????????????????????????????????????????????????????????????????????????????????????????????????????????????????????????????????????????????????????????????????????????????????????????????????????????????????????????????????????????????????????????????????????????????????????????????????????????????????????????????????????????????????????????????????????????????????????????????????????????????????????????????????????????????????????????????????????????????????????????????????????????????????????????????????????????????????????????????????????????????????????????????????????????????????????????????????????????????????????????????????????????????????????????????????????????????????????????????????????????????????????????????????????????????????????????????????????????????????????????????????????????????????????????????????????????????????????????????????????????????????????????????????????????????????????????????????????????????????????????????????????????????????????????????????????????????????????????????????????????????????????????????????????????????????????????????????????????????????????????????????????????????????????????????????????????????????????????????????????????????????????????????????????????????????????????????????????????????????????????????????????????????????????????????????????????????????????????????????????????????????????????????????????????????????????????????????????????????????????????????????????????????????????????????????????????????????????????????????????????????????????????????????????????????????????????????????????????????????????????????????????????????????????????????????????????????????????????????????????????????????????????????????????????????????????????????????????????????????????????????????????????????????????????????????????????????????????????????????????????????????????????????????????????????????????????????????????????????????????????????????????????????????????????????????????????????????????????????????????????????????????????????????????????????????????????????????????????????????????????????????????????????????????????????????????????????????????????????????????????????????????????????????????????????????????????????????????????????????????????????????????????????????????????????????????????????????????????????????????????????????????????????????????????????????????????????????????????????????????????????????????????????????????????????????????????????????????????????????????????????????????????????????????????????????????????????????????????????????????????????????????????????????????????????????????????????????????????????????????????????????????????????????????????????????????????????????????????????????????????????????????????????????????????????????????????????????????????????????????????????????????????????????????????????????????????????????????????????????????????????????????????????????????????????????????????????????????????????????????????????????????????-Stenobiella   0111010000000000000010101001011101011020011110110011100101110001000111211210001200011100211000011101011TAAACTTCAGCCATTTACCGCACAATGACTATTTTCAACTAATCATAAAAACATTGGTA-TCTTATATTTTATTTTCGGAATATGATCTGGAATAATCGGTACATCTTTAAGAATGCTAATTCGAACAGAATTAAGTCATTCAGGATTATTATTAGAAAACGAACAATTATATAATTGCAATTGTTACTACTCATGCTTTTGTTATAATTTT-TTTATAGTAATACCTATCATAATTGGAGGGTTTGGTAATTGACTAGTACCCCTAATATTGGGGGCCCCTGACATGGCTTTTCCTCGAATAAATAATATAAGATTTTGACTTTTGCCCCCATCATTAATATTAATACTCACTAGAAATATTTCAGAATTAGGAATTGGGACTGGTTGAACAGTATACCCCCCACTATCGACTAACCTATTTCATAGAAGAGCATCTGTAGATTTAGCAATTTTTTCCTTACATCTAGCTGGAATCTCATCTATTTTAGGGGCTATTAATTTTATTACAACAATTATCAATATACGTCCCTTAAATATAAGATTTGATCGAATTTCACTATTTGTGTGATCTGTATTTATCACTGCAATTTTATTATTATTATCCCTCCCAGTTTTAGCGGGAGCTATTACAATATTATTGTCAGACCGAAATTTTAATACATCCTTTTTTGACCCTGCTGGAGGAGGAGACCCTATTTTATATCAACACTTATTCTGATTTTTTGGTCATC-------------------------------------------------------ATAAAAAGTCTGACCTGCCC-CTGAAATAAT-TTTAAAGGGCCGCGGTATTTTGACTGTGCAAAGGTAGCATAATCATTTGTCTTTTAATTGAAGGCTGGAATGAATGGTTAAATGAAGTATTAACTGTCTCATTTTAAAAAATATAAAAATTTAATTTTTTAGTTAAAAAGCTAAAATTTATTTAGAGGACGAGAAGACCCTATAAATCTTTAT--TT-ATTTTATTAAATTAAATTTTAGATAAAAATTATTTATTATAATAAAA--TAAATTTTATTGGGGTGATAGGAAAATTTAATTAACTTTTTTT---ATTATAT-CTATTGATTTATAAATATTTGATCCATTATTAATGATTATAAGATTAAGATACTTTAGGGATAACAGCGTA-ATTTTTTTAGAGAGTTCTTATCGATAAAAAAGATTGCGACCTCGATGTTGAATTAAAGATTAATTTTAAATGCAGAAGTTTAA-TAATTAGGTCTGTTCGACCTTTAAATCT-----------------------------------TGATCCTTGCCAGTAGTCATATGCTTGTCTCAAAGATTAAGCCATGCATGTCTCAGTACAAGCCAAATTAAGGTGAAACCGCGAAAGGCTCATTATATCAGTTATGGTTCCTTAGATCGTACCCACATTTACTTGGATAACTGTGGT-----AATTCTAGAGCTAATACATG---CAAACAG--AGTTC-CAACCAGAGATGGAAGGAATGCTTTTATTAGATCAAAACCAATCGATGTT--------------------------GTCATATATTT----ATATTT-TTTATATT--------------------------------------------------------TATATATG----TT------GCATCGTTTTATTTGGTGACTCTGAATAACTTTAAGCTGATCGCACGGTCTC------GTACCGGCGACGCATCTTTCAAAT---------GTCTGCCTTATCAACTGTCGATGGTAGGTTCTGCGCCTACCATGG-----TTGTAACGGGTAACGGGGAATCAGGGTTCG-------ATTCCGGAGAGGGAGCCTGAGAAACGGCTACCACATCCAA------GGAAGGCAGCAGGCGCGCAAATTACCCACTCCCGGCACGGGGAGGTA--------GTGACG--AAAAATAACGATACGGGACTCATCCGAGGCCCCGTAATCGGAATGAGTACACTTTAAATCCTTTAACG-AGGATCCATTAGAG--GGCAAGTCTGGTGCCAGCAG---CCGCGGTAATTCCAGCTCTAATAGCGTATATTAAAGTTGTTGCGGTTAAAAAGCTCGTAGTCGAATCTGTGTCTTACACTGTCGGTTCACCGCTCGCGGTGTTCAACTGGCATGTTATG---------------------------------------------------------------------------------------------------------------------------------------------------------------------------------------------------------------------------------------------------------------------------------------------------------------------------------------------------------------------------------------------------------------------------------------------------------------------------------------------------------------------------------------------------------------------------------------------------------------------------------------------------------------------------------------------------------------------------------------------------------------------------------------------------------------------------------------------------------------------------------------------------------------------------------------------------------------------------------------------------------------------------------------------------------------------------------------------------------------------------------------------------------------------------------------------------------------------------------------------------------------------------------------------------------------------------------------------------------------------------------------------------------------------------------------------------------------------------------------------------------------TGTATTCATCACGG-AACAAATCGATGCTACATGACGTCACAGAATCACGGTTTTGCGGTGGATGTTGAAACATTACC?AAAGAGTGGGAAGCGTTATTCACAAATGCAAATGATAAAACAAATGAGGGAATAATTCACTCAAAATTACCGTATTTCAGTGTACAATTCCATCCGGAGCATACTGCTGGTCCGGAAGACTTGGAAAGCCTTTTCGATGTGTTTTTAAACTCCGTTAAGTCGTATATTTCCAACGAAACGTTCTGTGTTAAAGATGAACTTATAAAACTTTTGTCGTATGATC---GTCCACATTCCACACTAGTAAAAATACCAAAAAAGGTGCTAATTATCGGTTCCGGTGGGTTATCCATCGGTCAAGCTGGTGAATTTGATTATTCCGGGTCACAAGCCATTAAAGCGTTAAAAGAGGAGAATATACAAACGGTTTTGATGAATCCAAATATCGCGACAGTTCAGACGTCAAAAGGTCTCGCAGATAAAGTTTATTTCTTACCGTTAATTCCTGAATACGTGGAGCAGGTGATTCGCTCTGAGCGTCCAGATGGCGTTCTTTTAACATTCGGTGGTCAAACGGCGTTAAATTGTGGCGTGGAACTCGAACAAGCTGGTGTATTCAAAAAATATGGTGTTCGAATATTAGGCACGCCGATTAAATCGATTATAGAAACGGAAGATCGAAAGATATTCGCCGAACGTGTGAATGAGATTGGTGAGAAAGTTGCACCCAGTGCAGCTGTTTACTCCGTTCAAGAAGCGTTGAACGCCGCAGATGTGATTGGATATCCCGTGATGGCGCGTGCAGCATTTTCGTTAGGTGGTTTAGGATCAGGTTTTGCTAATAACGCAGACGAGTTGAAAATTCTTGCTTCACAAGCTTTAGCGCATTCCAATCAGTTAATCATTGATAAATCGTTACGCGGTTGGAAAGAAGTTGAATACGAAGTTGTACGCGATGCATTTGATAACTGCATCACAGTTTGCAATATGGAAAATGTTGACCCATTGGGAATTCATACCGGTGAATCAATTGTCGTTGCTCCGAGTCAAACGTTATCAAACCGAGAGTACAATATGTTGCGAACCACTGCGATCCGAGTGATTCGACATTTTGGCGTCGTTGGTGAGTGTAACATTCAATATGCTTTGAATCCCCACTCGGAGGAATATTACATTATTGAAGTGAACGCTCGGTTATCACGAAGTTCCGCATTAGCCAGTAAAGCCACAGGTTATCCTTTGGCTTATGTTGCAGCCAAACTAGCATTAGGTTCTTCTCTTCCGGACATCAAAAACTCCGTTACTGGTGAAACAACAGCCTGTTTTGAACCTAGCTTAGATTACTGTGTTGTAAAAATTCCACGCTGGGATTTAAGTAAATTCAGTCGTGTAAGCTCCAAAATCGGTAGTTCCA-TGAAGAGTGTCGGTGAAGTTATGGCAATTGGTCGTAAATTCGAAGAAGCATTTCAAAAGGCGCTACGTATGGTCGATGAGAACGTTAGTGGTT-------TTGATCCAAACTTACAGAAAATAAATGATGCTGATCTAAAAGAACCAACTGAT----AAGCGGATGTTTGTCATTGCCGCTTCGCTGAAAGCCGGTTACACTGTCGAAAAACTTCATGATTTAACGAAAATTGATCCATGGTTTTTACAGAAGATGAAGAATATCATTGATTTACACACGATGTTAACATTGTT----GGATCAACAAGGAG---TGACACGTTCTATTCTACATCAAGCGAAATGTTATGGGTTTTCGGATAAGCAAATCGCTAACGCTGTTGCAAGTACGGAATTGGCTATACGGTTACATCGAAAAGAGTGTGGTGTTTTACCGTACGTGAAGCAGATTGACACAGTAGCAGCCGAATGGCCAGCTTGTACCAATTACTTATATTTGACGTA------CAATG-CTGATTCCCACGATATAGAATTCCCTGGTGGGTATATTATGGTGATTGGATCCGGC--GTTTATCGTATCGGAAGTTCAGTAGAGTTCGA--TTGGTGT-----GCTGTTG-GTTCTCTACGTGAATTGCGTAAACTTGGAAAG--------AAAACGATTATGGTTAACTATAACCCGGAAACGGTTAGTACTGATTACGATATGAGCGATCGAC-TGTATTTCGAAGAAATATCCTTTGAAGTGGT-TATGG-ATATCTATAACCA--------------------Tanzanberotha   0110000000??000?0000??????000211111--00010110111000?0001?10110010001?????111000211?111??????????????011------------------------------------------------------------------------------------------------------------------------------------------------------------------------------------------------------------------------------------------------------------------------------------------------------------------------------------------------------------------------------------------------------------------------------------------------------------------------------------------------------------------------------------------------------------------------------------------------------------------------------------------------------------------------------------------------------------------------------------------------------------------------------------------------------------------------------------------------------------------------------------------------------------------------------------------------------------------------------------------------------------------------------------------------------------------------------------------------------------------------------------------------------------------------------------------------------------------------------------------------------------------------------------------------------------------------------------------------------------------------------------------------------------------------------------------------------------------------------------------------------------------------------------------------------------------------------------------------------------------------------------------------------------------------------------------------------------------------------------------------------------------------------------------------------------------------------------------------------------------------------------------------------------------------------------------------------------------------------------------------------------------------------------------------------------------------------------------------------------------------------------------------------------------------------------------------------------------------------------------------------------------------------------------------------------------------------------------------------------------------------------------------------------------------------------------------------------------------------------------------------------------------------------------------------------------------------------------------------------------------------------------------------------------------------------------------------------------------------------------------------------------------------------------------------------------------------------------------------------------------------------------------------------------------------------------------------------------------------------------------------------------------------------------------------------------------------------------------------------------------------------------------------------------------------------------------------------------------------------------------------------------------------------------------------------------------------------------------------------------------------------------------------------------------------------------------------------------------------------------------------------------------------------------------------------------------------------------------------------------------------------------------------------------------------------------------------------------------------------------------------------------------------------------------------------------------------------------------------------------------------------------------------------------------------------------------------------------------------------------------------------------------------------------------------------------------------------------------------------------------------------------------------------------------------------------------------------------------------------------------------------------------------------------------------------------------------------------------------------------------------------------------------------------------------------------------------------------------------------------------------------------------------------------------------------------------------------------------------------------------------------------------------------------------------------------------------------------------------------------------------------------------------------------------------------------------------------------------------------------------------------------------------------------------------------------------------------------------------------------------------------------------------------------------------------------------------------------------------------------------------------------------------------------------------------------------------------------------------------------------------------------------------------------------------------------------------------------------------------------------------------------------------------------------------------------------------------------------------------------------------------------------------------------------------------------------------------------------------------------------------------------------------------------------------------------------------------------------------------------------------------------------------------------------------------------------------------------------------------------------------------------------------------------------------------------------------------------------------------------------------------------------------------------------------------------------------------------------------------------------------------------------------Trichoberotha   ??1??0000???00??0?0??????00110100100?0001-100100000?0?01?101010100001000021010?10??11100111?000001?0011------------------------------------------------------------------------------------------------------------------------------------------------------------------------------------------------------------------------------------------------------------------------------------------------------------------------------------------------------------------------------------------------------------------------------------------------------------------------------------------------------------------------------------------------------------------------------------------------------------------------------------------------------------------------------------------------------------------------------------------------------------------------------------------------------------------------------------------------------------------------------------------------------------------------------------------------------------------------------------------------------------------------------------------------------------------------------------------------------------------------------------------------------------------------------------------------------------------------------------------------------------------------------------------------------------------------------------------------------------------------------------------------------------------------------------------------------------------------------------------------------------------------------------------------------------------------------------------------------------------------------------------------------------------------------------------------------------------------------------------------------------------------------------------------------------------------------------------------------------------------------------------------------------------------------------------------------------------------------------------------------------------------------------------------------------------------------------------------------------------------------------------------------------------------------------------------------------------------------------------------------------------------------------------------------------------------------------------------------------------------------------------------------------------------------------------------------------------------------------------------------------------------------------------------------------------------------------------------------------------------------------------------------------------------------------------------------------------------------------------------------------------------------------------------------------------------------------------------------------------------------------------------------------------------------------------------------------------------------------------------------------------------------------------------------------------------------------------------------------------------------------------------------------------------------------------------------------------------------------------------------------------------------------------------------------------------------------------------------------------------------------------------------------------------------------------------------------------------------------------------------------------------------------------------------------------------------------------------------------------------------------------------------------------------------------------------------------------------------------------------------------------------------------------------------------------------------------------------------------------------------------------------------------------------------------------------------------------------------------------------------------------------------------------------------------------------------------------------------------------------------------------------------------------------------------------------------------------------------------------------------------------------------------------------------------------------------------------------------------------------------------------------------------------------------------------------------------------------------------------------------------------------------------------------------------------------------------------------------------------------------------------------------------------------------------------------------------------------------------------------------------------------------------------------------------------------------------------------------------------------------------------------------------------------------------------------------------------------------------------------------------------------------------------------------------------------------------------------------------------------------------------------------------------------------------------------------------------------------------------------------------------------------------------------------------------------------------------------------------------------------------------------------------------------------------------------------------------------------------------------------------------------------------------------------------------------------------------------------------------------------------------------------------------------------------------------------------------------------------------------------------------------------------------------------------------------------------------------------------------------------------------------------------------------------------------------------------------------------------------------------------------------------------------------------------------------------------------------------------------------------------------------------Trichoma   011000000000000000001110100100101100110011100111000000010101000100001000021010110-211100321?000111?1011------------------------------------------------------------------------------------------------------------------------------------------------------------------------------------------------------------------------------------------------------------------------------------------------------------------------------------------------------------------------------------------------------------------------------------------------------------------------------------------------------------------------------------------------------------------------------------------------------------------------------------------------------------------------------------------------------------------------------------------------------------------------------------------------------------------------------------------------------------------------------------------------------------------------------------------------------------------------------------------------------------------------------------------------------------------------------------------------------------------------------------------------------------------------------------------------------------------------------------------------------------------------------------------------------------------------------------------------------------------------------------------------------------------------------------------------------------------------------------------------------------------------------------------------------------------------------------------------------------------------------------------------------------------------------------------------------------------------------------------------------------------------------------------------------------------------------------------------------------------------------------------------------------------------------------------------------------------------------------------------------------------------------------------------------------------------------------------------------------------------------------------------------------------------------------------------------------------------------------------------------------------------------------------------------------------------------------------------------------------------------------------------------------------------------------------------------------------------------------------------------------------------------------------------------------------------------------------------------------------------------------------------------------------------------------------------------------------------------------------------------------------------------------------------------------------------------------------------------------------------------------------------------------------------------------------------------------------------------------------------------------------------------------------------------------------------------------------------------------------------------------------------------------------------------------------------------------------------------------------------------------------------------------------------------------------------------------------------------------------------------------------------------------------------------------------------------------------------------------------------------------------------------------------------------------------------------------------------------------------------------------------------------------------------------------------------------------------------------------------------------------------------------------------------------------------------------------------------------------------------------------------------------------------------------------------------------------------------------------------------------------------------------------------------------------------------------------------------------------------------------------------------------------------------------------------------------------------------------------------------------------------------------------------------------------------------------------------------------------------------------------------------------------------------------------------------------------------------------------------------------------------------------------------------------------------------------------------------------------------------------------------------------------------------------------------------------------------------------------------------------------------------------------------------------------------------------------------------------------------------------------------------------------------------------------------------------------------------------------------------------------------------------------------------------------------------------------------------------------------------------------------------------------------------------------------------------------------------------------------------------------------------------------------------------------------------------------------------------------------------------------------------------------------------------------------------------------------------------------------------------------------------------------------------------------------------------------------------------------------------------------------------------------------------------------------------------------------------------------------------------------------------------------------------------------------------------------------------------------------------------------------------------------------------------------------------------------------------------------------------------------------------------------------------------------------------------------------------------------------------------------------------------Sinosmylites   ?????0000???0000?0?00?01?0000101001--10110111101001?0001?10012110000???1???????????????????????????????------------------------------------------------------------------------------------------------------------------------------------------------------------------------------------------------------------------------------------------------------------------------------------------------------------------------------------------------------------------------------------------------------------------------------------------------------------------------------------------------------------------------------------------------------------------------------------------------------------------------------------------------------------------------------------------------------------------------------------------------------------------------------------------------------------------------------------------------------------------------------------------------------------------------------------------------------------------------------------------------------------------------------------------------------------------------------------------------------------------------------------------------------------------------------------------------------------------------------------------------------------------------------------------------------------------------------------------------------------------------------------------------------------------------------------------------------------------------------------------------------------------------------------------------------------------------------------------------------------------------------------------------------------------------------------------------------------------------------------------------------------------------------------------------------------------------------------------------------------------------------------------------------------------------------------------------------------------------------------------------------------------------------------------------------------------------------------------------------------------------------------------------------------------------------------------------------------------------------------------------------------------------------------------------------------------------------------------------------------------------------------------------------------------------------------------------------------------------------------------------------------------------------------------------------------------------------------------------------------------------------------------------------------------------------------------------------------------------------------------------------------------------------------------------------------------------------------------------------------------------------------------------------------------------------------------------------------------------------------------------------------------------------------------------------------------------------------------------------------------------------------------------------------------------------------------------------------------------------------------------------------------------------------------------------------------------------------------------------------------------------------------------------------------------------------------------------------------------------------------------------------------------------------------------------------------------------------------------------------------------------------------------------------------------------------------------------------------------------------------------------------------------------------------------------------------------------------------------------------------------------------------------------------------------------------------------------------------------------------------------------------------------------------------------------------------------------------------------------------------------------------------------------------------------------------------------------------------------------------------------------------------------------------------------------------------------------------------------------------------------------------------------------------------------------------------------------------------------------------------------------------------------------------------------------------------------------------------------------------------------------------------------------------------------------------------------------------------------------------------------------------------------------------------------------------------------------------------------------------------------------------------------------------------------------------------------------------------------------------------------------------------------------------------------------------------------------------------------------------------------------------------------------------------------------------------------------------------------------------------------------------------------------------------------------------------------------------------------------------------------------------------------------------------------------------------------------------------------------------------------------------------------------------------------------------------------------------------------------------------------------------------------------------------------------------------------------------------------------------------------------------------------------------------------------------------------------------------------------------------------------------------------------------------------------------------------------------------------------------------------------------------------------------------------------------------------------------------------------------------------------------------------------Berothone   ?????????????????????????0001211101--010?0200011000?0?01???????????????????????????????????????????????------------------------------------------------------------------------------------------------------------------------------------------------------------------------------------------------------------------------------------------------------------------------------------------------------------------------------------------------------------------------------------------------------------------------------------------------------------------------------------------------------------------------------------------------------------------------------------------------------------------------------------------------------------------------------------------------------------------------------------------------------------------------------------------------------------------------------------------------------------------------------------------------------------------------------------------------------------------------------------------------------------------------------------------------------------------------------------------------------------------------------------------------------------------------------------------------------------------------------------------------------------------------------------------------------------------------------------------------------------------------------------------------------------------------------------------------------------------------------------------------------------------------------------------------------------------------------------------------------------------------------------------------------------------------------------------------------------------------------------------------------------------------------------------------------------------------------------------------------------------------------------------------------------------------------------------------------------------------------------------------------------------------------------------------------------------------------------------------------------------------------------------------------------------------------------------------------------------------------------------------------------------------------------------------------------------------------------------------------------------------------------------------------------------------------------------------------------------------------------------------------------------------------------------------------------------------------------------------------------------------------------------------------------------------------------------------------------------------------------------------------------------------------------------------------------------------------------------------------------------------------------------------------------------------------------------------------------------------------------------------------------------------------------------------------------------------------------------------------------------------------------------------------------------------------------------------------------------------------------------------------------------------------------------------------------------------------------------------------------------------------------------------------------------------------------------------------------------------------------------------------------------------------------------------------------------------------------------------------------------------------------------------------------------------------------------------------------------------------------------------------------------------------------------------------------------------------------------------------------------------------------------------------------------------------------------------------------------------------------------------------------------------------------------------------------------------------------------------------------------------------------------------------------------------------------------------------------------------------------------------------------------------------------------------------------------------------------------------------------------------------------------------------------------------------------------------------------------------------------------------------------------------------------------------------------------------------------------------------------------------------------------------------------------------------------------------------------------------------------------------------------------------------------------------------------------------------------------------------------------------------------------------------------------------------------------------------------------------------------------------------------------------------------------------------------------------------------------------------------------------------------------------------------------------------------------------------------------------------------------------------------------------------------------------------------------------------------------------------------------------------------------------------------------------------------------------------------------------------------------------------------------------------------------------------------------------------------------------------------------------------------------------------------------------------------------------------------------------------------------------------------------------------------------------------------------------------------------------------------------------------------------------------------------------------------------------------------------------------------------------------------------------------------------------------------------------------------------------------------------------------------------------------Krokhathone   ??????????0??????????????000000100???020?01111110???0?01???????????????????????????????????????????????------------------------------------------------------------------------------------------------------------------------------------------------------------------------------------------------------------------------------------------------------------------------------------------------------------------------------------------------------------------------------------------------------------------------------------------------------------------------------------------------------------------------------------------------------------------------------------------------------------------------------------------------------------------------------------------------------------------------------------------------------------------------------------------------------------------------------------------------------------------------------------------------------------------------------------------------------------------------------------------------------------------------------------------------------------------------------------------------------------------------------------------------------------------------------------------------------------------------------------------------------------------------------------------------------------------------------------------------------------------------------------------------------------------------------------------------------------------------------------------------------------------------------------------------------------------------------------------------------------------------------------------------------------------------------------------------------------------------------------------------------------------------------------------------------------------------------------------------------------------------------------------------------------------------------------------------------------------------------------------------------------------------------------------------------------------------------------------------------------------------------------------------------------------------------------------------------------------------------------------------------------------------------------------------------------------------------------------------------------------------------------------------------------------------------------------------------------------------------------------------------------------------------------------------------------------------------------------------------------------------------------------------------------------------------------------------------------------------------------------------------------------------------------------------------------------------------------------------------------------------------------------------------------------------------------------------------------------------------------------------------------------------------------------------------------------------------------------------------------------------------------------------------------------------------------------------------------------------------------------------------------------------------------------------------------------------------------------------------------------------------------------------------------------------------------------------------------------------------------------------------------------------------------------------------------------------------------------------------------------------------------------------------------------------------------------------------------------------------------------------------------------------------------------------------------------------------------------------------------------------------------------------------------------------------------------------------------------------------------------------------------------------------------------------------------------------------------------------------------------------------------------------------------------------------------------------------------------------------------------------------------------------------------------------------------------------------------------------------------------------------------------------------------------------------------------------------------------------------------------------------------------------------------------------------------------------------------------------------------------------------------------------------------------------------------------------------------------------------------------------------------------------------------------------------------------------------------------------------------------------------------------------------------------------------------------------------------------------------------------------------------------------------------------------------------------------------------------------------------------------------------------------------------------------------------------------------------------------------------------------------------------------------------------------------------------------------------------------------------------------------------------------------------------------------------------------------------------------------------------------------------------------------------------------------------------------------------------------------------------------------------------------------------------------------------------------------------------------------------------------------------------------------------------------------------------------------------------------------------------------------------------------------------------------------------------------------------------------------------------------------------------------------------------------------------------------------------------------------------------------------------------------------Mesithone   ???????????????????????????0111110???011(0 1)0110101000?0001???????????????????????????????????????????????------------------------------------------------------------------------------------------------------------------------------------------------------------------------------------------------------------------------------------------------------------------------------------------------------------------------------------------------------------------------------------------------------------------------------------------------------------------------------------------------------------------------------------------------------------------------------------------------------------------------------------------------------------------------------------------------------------------------------------------------------------------------------------------------------------------------------------------------------------------------------------------------------------------------------------------------------------------------------------------------------------------------------------------------------------------------------------------------------------------------------------------------------------------------------------------------------------------------------------------------------------------------------------------------------------------------------------------------------------------------------------------------------------------------------------------------------------------------------------------------------------------------------------------------------------------------------------------------------------------------------------------------------------------------------------------------------------------------------------------------------------------------------------------------------------------------------------------------------------------------------------------------------------------------------------------------------------------------------------------------------------------------------------------------------------------------------------------------------------------------------------------------------------------------------------------------------------------------------------------------------------------------------------------------------------------------------------------------------------------------------------------------------------------------------------------------------------------------------------------------------------------------------------------------------------------------------------------------------------------------------------------------------------------------------------------------------------------------------------------------------------------------------------------------------------------------------------------------------------------------------------------------------------------------------------------------------------------------------------------------------------------------------------------------------------------------------------------------------------------------------------------------------------------------------------------------------------------------------------------------------------------------------------------------------------------------------------------------------------------------------------------------------------------------------------------------------------------------------------------------------------------------------------------------------------------------------------------------------------------------------------------------------------------------------------------------------------------------------------------------------------------------------------------------------------------------------------------------------------------------------------------------------------------------------------------------------------------------------------------------------------------------------------------------------------------------------------------------------------------------------------------------------------------------------------------------------------------------------------------------------------------------------------------------------------------------------------------------------------------------------------------------------------------------------------------------------------------------------------------------------------------------------------------------------------------------------------------------------------------------------------------------------------------------------------------------------------------------------------------------------------------------------------------------------------------------------------------------------------------------------------------------------------------------------------------------------------------------------------------------------------------------------------------------------------------------------------------------------------------------------------------------------------------------------------------------------------------------------------------------------------------------------------------------------------------------------------------------------------------------------------------------------------------------------------------------------------------------------------------------------------------------------------------------------------------------------------------------------------------------------------------------------------------------------------------------------------------------------------------------------------------------------------------------------------------------------------------------------------------------------------------------------------------------------------------------------------------------------------------------------------------------------------------------------------------------------------------------------------------------------------------------------Epimesoberotha   ?????????????????????????0000101?0?--01(0 1)?010?101??????01???????????????????????????????????????????????------------------------------------------------------------------------------------------------------------------------------------------------------------------------------------------------------------------------------------------------------------------------------------------------------------------------------------------------------------------------------------------------------------------------------------------------------------------------------------------------------------------------------------------------------------------------------------------------------------------------------------------------------------------------------------------------------------------------------------------------------------------------------------------------------------------------------------------------------------------------------------------------------------------------------------------------------------------------------------------------------------------------------------------------------------------------------------------------------------------------------------------------------------------------------------------------------------------------------------------------------------------------------------------------------------------------------------------------------------------------------------------------------------------------------------------------------------------------------------------------------------------------------------------------------------------------------------------------------------------------------------------------------------------------------------------------------------------------------------------------------------------------------------------------------------------------------------------------------------------------------------------------------------------------------------------------------------------------------------------------------------------------------------------------------------------------------------------------------------------------------------------------------------------------------------------------------------------------------------------------------------------------------------------------------------------------------------------------------------------------------------------------------------------------------------------------------------------------------------------------------------------------------------------------------------------------------------------------------------------------------------------------------------------------------------------------------------------------------------------------------------------------------------------------------------------------------------------------------------------------------------------------------------------------------------------------------------------------------------------------------------------------------------------------------------------------------------------------------------------------------------------------------------------------------------------------------------------------------------------------------------------------------------------------------------------------------------------------------------------------------------------------------------------------------------------------------------------------------------------------------------------------------------------------------------------------------------------------------------------------------------------------------------------------------------------------------------------------------------------------------------------------------------------------------------------------------------------------------------------------------------------------------------------------------------------------------------------------------------------------------------------------------------------------------------------------------------------------------------------------------------------------------------------------------------------------------------------------------------------------------------------------------------------------------------------------------------------------------------------------------------------------------------------------------------------------------------------------------------------------------------------------------------------------------------------------------------------------------------------------------------------------------------------------------------------------------------------------------------------------------------------------------------------------------------------------------------------------------------------------------------------------------------------------------------------------------------------------------------------------------------------------------------------------------------------------------------------------------------------------------------------------------------------------------------------------------------------------------------------------------------------------------------------------------------------------------------------------------------------------------------------------------------------------------------------------------------------------------------------------------------------------------------------------------------------------------------------------------------------------------------------------------------------------------------------------------------------------------------------------------------------------------------------------------------------------------------------------------------------------------------------------------------------------------------------------------------------------------------------------------------------------------------------------------------------------------------------------------------------------------------------------------Pseudosisyra   ??????????????????????????001111111--0201011101101000001???????????????????????????????????????????????------------------------------------------------------------------------------------------------------------------------------------------------------------------------------------------------------------------------------------------------------------------------------------------------------------------------------------------------------------------------------------------------------------------------------------------------------------------------------------------------------------------------------------------------------------------------------------------------------------------------------------------------------------------------------------------------------------------------------------------------------------------------------------------------------------------------------------------------------------------------------------------------------------------------------------------------------------------------------------------------------------------------------------------------------------------------------------------------------------------------------------------------------------------------------------------------------------------------------------------------------------------------------------------------------------------------------------------------------------------------------------------------------------------------------------------------------------------------------------------------------------------------------------------------------------------------------------------------------------------------------------------------------------------------------------------------------------------------------------------------------------------------------------------------------------------------------------------------------------------------------------------------------------------------------------------------------------------------------------------------------------------------------------------------------------------------------------------------------------------------------------------------------------------------------------------------------------------------------------------------------------------------------------------------------------------------------------------------------------------------------------------------------------------------------------------------------------------------------------------------------------------------------------------------------------------------------------------------------------------------------------------------------------------------------------------------------------------------------------------------------------------------------------------------------------------------------------------------------------------------------------------------------------------------------------------------------------------------------------------------------------------------------------------------------------------------------------------------------------------------------------------------------------------------------------------------------------------------------------------------------------------------------------------------------------------------------------------------------------------------------------------------------------------------------------------------------------------------------------------------------------------------------------------------------------------------------------------------------------------------------------------------------------------------------------------------------------------------------------------------------------------------------------------------------------------------------------------------------------------------------------------------------------------------------------------------------------------------------------------------------------------------------------------------------------------------------------------------------------------------------------------------------------------------------------------------------------------------------------------------------------------------------------------------------------------------------------------------------------------------------------------------------------------------------------------------------------------------------------------------------------------------------------------------------------------------------------------------------------------------------------------------------------------------------------------------------------------------------------------------------------------------------------------------------------------------------------------------------------------------------------------------------------------------------------------------------------------------------------------------------------------------------------------------------------------------------------------------------------------------------------------------------------------------------------------------------------------------------------------------------------------------------------------------------------------------------------------------------------------------------------------------------------------------------------------------------------------------------------------------------------------------------------------------------------------------------------------------------------------------------------------------------------------------------------------------------------------------------------------------------------------------------------------------------------------------------------------------------------------------------------------------------------------------------------------------------------------------------------------------------------------------------------------------------------------------------------------------------------------------------------------------------Oloberotha   ?????0??0????????????????0?0121?00???00110110101000?0001?00001010000102000?????????????????????????????------------------------------------------------------------------------------------------------------------------------------------------------------------------------------------------------------------------------------------------------------------------------------------------------------------------------------------------------------------------------------------------------------------------------------------------------------------------------------------------------------------------------------------------------------------------------------------------------------------------------------------------------------------------------------------------------------------------------------------------------------------------------------------------------------------------------------------------------------------------------------------------------------------------------------------------------------------------------------------------------------------------------------------------------------------------------------------------------------------------------------------------------------------------------------------------------------------------------------------------------------------------------------------------------------------------------------------------------------------------------------------------------------------------------------------------------------------------------------------------------------------------------------------------------------------------------------------------------------------------------------------------------------------------------------------------------------------------------------------------------------------------------------------------------------------------------------------------------------------------------------------------------------------------------------------------------------------------------------------------------------------------------------------------------------------------------------------------------------------------------------------------------------------------------------------------------------------------------------------------------------------------------------------------------------------------------------------------------------------------------------------------------------------------------------------------------------------------------------------------------------------------------------------------------------------------------------------------------------------------------------------------------------------------------------------------------------------------------------------------------------------------------------------------------------------------------------------------------------------------------------------------------------------------------------------------------------------------------------------------------------------------------------------------------------------------------------------------------------------------------------------------------------------------------------------------------------------------------------------------------------------------------------------------------------------------------------------------------------------------------------------------------------------------------------------------------------------------------------------------------------------------------------------------------------------------------------------------------------------------------------------------------------------------------------------------------------------------------------------------------------------------------------------------------------------------------------------------------------------------------------------------------------------------------------------------------------------------------------------------------------------------------------------------------------------------------------------------------------------------------------------------------------------------------------------------------------------------------------------------------------------------------------------------------------------------------------------------------------------------------------------------------------------------------------------------------------------------------------------------------------------------------------------------------------------------------------------------------------------------------------------------------------------------------------------------------------------------------------------------------------------------------------------------------------------------------------------------------------------------------------------------------------------------------------------------------------------------------------------------------------------------------------------------------------------------------------------------------------------------------------------------------------------------------------------------------------------------------------------------------------------------------------------------------------------------------------------------------------------------------------------------------------------------------------------------------------------------------------------------------------------------------------------------------------------------------------------------------------------------------------------------------------------------------------------------------------------------------------------------------------------------------------------------------------------------------------------------------------------------------------------------------------------------------------------------------------------------------------------------------------------------------------------------------------------------------------------------------------------------------------------------------------Sibelliberotha   0?1010000?0?00000?0?010100000001000101201011100001000001?0100001000110200?????????????002????0?0???????------------------------------------------------------------------------------------------------------------------------------------------------------------------------------------------------------------------------------------------------------------------------------------------------------------------------------------------------------------------------------------------------------------------------------------------------------------------------------------------------------------------------------------------------------------------------------------------------------------------------------------------------------------------------------------------------------------------------------------------------------------------------------------------------------------------------------------------------------------------------------------------------------------------------------------------------------------------------------------------------------------------------------------------------------------------------------------------------------------------------------------------------------------------------------------------------------------------------------------------------------------------------------------------------------------------------------------------------------------------------------------------------------------------------------------------------------------------------------------------------------------------------------------------------------------------------------------------------------------------------------------------------------------------------------------------------------------------------------------------------------------------------------------------------------------------------------------------------------------------------------------------------------------------------------------------------------------------------------------------------------------------------------------------------------------------------------------------------------------------------------------------------------------------------------------------------------------------------------------------------------------------------------------------------------------------------------------------------------------------------------------------------------------------------------------------------------------------------------------------------------------------------------------------------------------------------------------------------------------------------------------------------------------------------------------------------------------------------------------------------------------------------------------------------------------------------------------------------------------------------------------------------------------------------------------------------------------------------------------------------------------------------------------------------------------------------------------------------------------------------------------------------------------------------------------------------------------------------------------------------------------------------------------------------------------------------------------------------------------------------------------------------------------------------------------------------------------------------------------------------------------------------------------------------------------------------------------------------------------------------------------------------------------------------------------------------------------------------------------------------------------------------------------------------------------------------------------------------------------------------------------------------------------------------------------------------------------------------------------------------------------------------------------------------------------------------------------------------------------------------------------------------------------------------------------------------------------------------------------------------------------------------------------------------------------------------------------------------------------------------------------------------------------------------------------------------------------------------------------------------------------------------------------------------------------------------------------------------------------------------------------------------------------------------------------------------------------------------------------------------------------------------------------------------------------------------------------------------------------------------------------------------------------------------------------------------------------------------------------------------------------------------------------------------------------------------------------------------------------------------------------------------------------------------------------------------------------------------------------------------------------------------------------------------------------------------------------------------------------------------------------------------------------------------------------------------------------------------------------------------------------------------------------------------------------------------------------------------------------------------------------------------------------------------------------------------------------------------------------------------------------------------------------------------------------------------------------------------------------------------------------------------------------------------------------------------------------------------------------------------------------------------------------------------------------------------------------------------------------------------------------------------------Araripeberotha   ?0????????????????????????0???0100???0101?1???????????0??000?10?0001???????????????????????????????????------------------------------------------------------------------------------------------------------------------------------------------------------------------------------------------------------------------------------------------------------------------------------------------------------------------------------------------------------------------------------------------------------------------------------------------------------------------------------------------------------------------------------------------------------------------------------------------------------------------------------------------------------------------------------------------------------------------------------------------------------------------------------------------------------------------------------------------------------------------------------------------------------------------------------------------------------------------------------------------------------------------------------------------------------------------------------------------------------------------------------------------------------------------------------------------------------------------------------------------------------------------------------------------------------------------------------------------------------------------------------------------------------------------------------------------------------------------------------------------------------------------------------------------------------------------------------------------------------------------------------------------------------------------------------------------------------------------------------------------------------------------------------------------------------------------------------------------------------------------------------------------------------------------------------------------------------------------------------------------------------------------------------------------------------------------------------------------------------------------------------------------------------------------------------------------------------------------------------------------------------------------------------------------------------------------------------------------------------------------------------------------------------------------------------------------------------------------------------------------------------------------------------------------------------------------------------------------------------------------------------------------------------------------------------------------------------------------------------------------------------------------------------------------------------------------------------------------------------------------------------------------------------------------------------------------------------------------------------------------------------------------------------------------------------------------------------------------------------------------------------------------------------------------------------------------------------------------------------------------------------------------------------------------------------------------------------------------------------------------------------------------------------------------------------------------------------------------------------------------------------------------------------------------------------------------------------------------------------------------------------------------------------------------------------------------------------------------------------------------------------------------------------------------------------------------------------------------------------------------------------------------------------------------------------------------------------------------------------------------------------------------------------------------------------------------------------------------------------------------------------------------------------------------------------------------------------------------------------------------------------------------------------------------------------------------------------------------------------------------------------------------------------------------------------------------------------------------------------------------------------------------------------------------------------------------------------------------------------------------------------------------------------------------------------------------------------------------------------------------------------------------------------------------------------------------------------------------------------------------------------------------------------------------------------------------------------------------------------------------------------------------------------------------------------------------------------------------------------------------------------------------------------------------------------------------------------------------------------------------------------------------------------------------------------------------------------------------------------------------------------------------------------------------------------------------------------------------------------------------------------------------------------------------------------------------------------------------------------------------------------------------------------------------------------------------------------------------------------------------------------------------------------------------------------------------------------------------------------------------------------------------------------------------------------------------------------------------------------------------------------------------------------------------------------------------------------------------------------------------------------------------------------Caririberotha   00?0??????????????????????0???11?0???010??1???????????0???00?00?0??????????????????????????????????????------------------------------------------------------------------------------------------------------------------------------------------------------------------------------------------------------------------------------------------------------------------------------------------------------------------------------------------------------------------------------------------------------------------------------------------------------------------------------------------------------------------------------------------------------------------------------------------------------------------------------------------------------------------------------------------------------------------------------------------------------------------------------------------------------------------------------------------------------------------------------------------------------------------------------------------------------------------------------------------------------------------------------------------------------------------------------------------------------------------------------------------------------------------------------------------------------------------------------------------------------------------------------------------------------------------------------------------------------------------------------------------------------------------------------------------------------------------------------------------------------------------------------------------------------------------------------------------------------------------------------------------------------------------------------------------------------------------------------------------------------------------------------------------------------------------------------------------------------------------------------------------------------------------------------------------------------------------------------------------------------------------------------------------------------------------------------------------------------------------------------------------------------------------------------------------------------------------------------------------------------------------------------------------------------------------------------------------------------------------------------------------------------------------------------------------------------------------------------------------------------------------------------------------------------------------------------------------------------------------------------------------------------------------------------------------------------------------------------------------------------------------------------------------------------------------------------------------------------------------------------------------------------------------------------------------------------------------------------------------------------------------------------------------------------------------------------------------------------------------------------------------------------------------------------------------------------------------------------------------------------------------------------------------------------------------------------------------------------------------------------------------------------------------------------------------------------------------------------------------------------------------------------------------------------------------------------------------------------------------------------------------------------------------------------------------------------------------------------------------------------------------------------------------------------------------------------------------------------------------------------------------------------------------------------------------------------------------------------------------------------------------------------------------------------------------------------------------------------------------------------------------------------------------------------------------------------------------------------------------------------------------------------------------------------------------------------------------------------------------------------------------------------------------------------------------------------------------------------------------------------------------------------------------------------------------------------------------------------------------------------------------------------------------------------------------------------------------------------------------------------------------------------------------------------------------------------------------------------------------------------------------------------------------------------------------------------------------------------------------------------------------------------------------------------------------------------------------------------------------------------------------------------------------------------------------------------------------------------------------------------------------------------------------------------------------------------------------------------------------------------------------------------------------------------------------------------------------------------------------------------------------------------------------------------------------------------------------------------------------------------------------------------------------------------------------------------------------------------------------------------------------------------------------------------------------------------------------------------------------------------------------------------------------------------------------------------------------------------------------------------------------------------------------------------------------------------------------------------------------------------------------------------Cantabroberotha   001000000?0?0000?????????0000101001--01110111011?0???001?00000000001???????????????????????????1???????------------------------------------------------------------------------------------------------------------------------------------------------------------------------------------------------------------------------------------------------------------------------------------------------------------------------------------------------------------------------------------------------------------------------------------------------------------------------------------------------------------------------------------------------------------------------------------------------------------------------------------------------------------------------------------------------------------------------------------------------------------------------------------------------------------------------------------------------------------------------------------------------------------------------------------------------------------------------------------------------------------------------------------------------------------------------------------------------------------------------------------------------------------------------------------------------------------------------------------------------------------------------------------------------------------------------------------------------------------------------------------------------------------------------------------------------------------------------------------------------------------------------------------------------------------------------------------------------------------------------------------------------------------------------------------------------------------------------------------------------------------------------------------------------------------------------------------------------------------------------------------------------------------------------------------------------------------------------------------------------------------------------------------------------------------------------------------------------------------------------------------------------------------------------------------------------------------------------------------------------------------------------------------------------------------------------------------------------------------------------------------------------------------------------------------------------------------------------------------------------------------------------------------------------------------------------------------------------------------------------------------------------------------------------------------------------------------------------------------------------------------------------------------------------------------------------------------------------------------------------------------------------------------------------------------------------------------------------------------------------------------------------------------------------------------------------------------------------------------------------------------------------------------------------------------------------------------------------------------------------------------------------------------------------------------------------------------------------------------------------------------------------------------------------------------------------------------------------------------------------------------------------------------------------------------------------------------------------------------------------------------------------------------------------------------------------------------------------------------------------------------------------------------------------------------------------------------------------------------------------------------------------------------------------------------------------------------------------------------------------------------------------------------------------------------------------------------------------------------------------------------------------------------------------------------------------------------------------------------------------------------------------------------------------------------------------------------------------------------------------------------------------------------------------------------------------------------------------------------------------------------------------------------------------------------------------------------------------------------------------------------------------------------------------------------------------------------------------------------------------------------------------------------------------------------------------------------------------------------------------------------------------------------------------------------------------------------------------------------------------------------------------------------------------------------------------------------------------------------------------------------------------------------------------------------------------------------------------------------------------------------------------------------------------------------------------------------------------------------------------------------------------------------------------------------------------------------------------------------------------------------------------------------------------------------------------------------------------------------------------------------------------------------------------------------------------------------------------------------------------------------------------------------------------------------------------------------------------------------------------------------------------------------------------------------------------------------------------------------------------------------------------------------------------------------------------------------------------------------------------------------------------------Aggregataberotha   001010000000000000000110?000000100001020101110(0 1)1011??001?10110020001?1????????????????001?01100011?????------------------------------------------------------------------------------------------------------------------------------------------------------------------------------------------------------------------------------------------------------------------------------------------------------------------------------------------------------------------------------------------------------------------------------------------------------------------------------------------------------------------------------------------------------------------------------------------------------------------------------------------------------------------------------------------------------------------------------------------------------------------------------------------------------------------------------------------------------------------------------------------------------------------------------------------------------------------------------------------------------------------------------------------------------------------------------------------------------------------------------------------------------------------------------------------------------------------------------------------------------------------------------------------------------------------------------------------------------------------------------------------------------------------------------------------------------------------------------------------------------------------------------------------------------------------------------------------------------------------------------------------------------------------------------------------------------------------------------------------------------------------------------------------------------------------------------------------------------------------------------------------------------------------------------------------------------------------------------------------------------------------------------------------------------------------------------------------------------------------------------------------------------------------------------------------------------------------------------------------------------------------------------------------------------------------------------------------------------------------------------------------------------------------------------------------------------------------------------------------------------------------------------------------------------------------------------------------------------------------------------------------------------------------------------------------------------------------------------------------------------------------------------------------------------------------------------------------------------------------------------------------------------------------------------------------------------------------------------------------------------------------------------------------------------------------------------------------------------------------------------------------------------------------------------------------------------------------------------------------------------------------------------------------------------------------------------------------------------------------------------------------------------------------------------------------------------------------------------------------------------------------------------------------------------------------------------------------------------------------------------------------------------------------------------------------------------------------------------------------------------------------------------------------------------------------------------------------------------------------------------------------------------------------------------------------------------------------------------------------------------------------------------------------------------------------------------------------------------------------------------------------------------------------------------------------------------------------------------------------------------------------------------------------------------------------------------------------------------------------------------------------------------------------------------------------------------------------------------------------------------------------------------------------------------------------------------------------------------------------------------------------------------------------------------------------------------------------------------------------------------------------------------------------------------------------------------------------------------------------------------------------------------------------------------------------------------------------------------------------------------------------------------------------------------------------------------------------------------------------------------------------------------------------------------------------------------------------------------------------------------------------------------------------------------------------------------------------------------------------------------------------------------------------------------------------------------------------------------------------------------------------------------------------------------------------------------------------------------------------------------------------------------------------------------------------------------------------------------------------------------------------------------------------------------------------------------------------------------------------------------------------------------------------------------------------------------------------------------------------------------------------------------------------------------------------------------------------------------------------------------------------------------Ansoberotha   0011?000000000000100001??0010000001--00010111100001??001?1000(1 2)01010101210?????????????1???10?0?201?????------------------------------------------------------------------------------------------------------------------------------------------------------------------------------------------------------------------------------------------------------------------------------------------------------------------------------------------------------------------------------------------------------------------------------------------------------------------------------------------------------------------------------------------------------------------------------------------------------------------------------------------------------------------------------------------------------------------------------------------------------------------------------------------------------------------------------------------------------------------------------------------------------------------------------------------------------------------------------------------------------------------------------------------------------------------------------------------------------------------------------------------------------------------------------------------------------------------------------------------------------------------------------------------------------------------------------------------------------------------------------------------------------------------------------------------------------------------------------------------------------------------------------------------------------------------------------------------------------------------------------------------------------------------------------------------------------------------------------------------------------------------------------------------------------------------------------------------------------------------------------------------------------------------------------------------------------------------------------------------------------------------------------------------------------------------------------------------------------------------------------------------------------------------------------------------------------------------------------------------------------------------------------------------------------------------------------------------------------------------------------------------------------------------------------------------------------------------------------------------------------------------------------------------------------------------------------------------------------------------------------------------------------------------------------------------------------------------------------------------------------------------------------------------------------------------------------------------------------------------------------------------------------------------------------------------------------------------------------------------------------------------------------------------------------------------------------------------------------------------------------------------------------------------------------------------------------------------------------------------------------------------------------------------------------------------------------------------------------------------------------------------------------------------------------------------------------------------------------------------------------------------------------------------------------------------------------------------------------------------------------------------------------------------------------------------------------------------------------------------------------------------------------------------------------------------------------------------------------------------------------------------------------------------------------------------------------------------------------------------------------------------------------------------------------------------------------------------------------------------------------------------------------------------------------------------------------------------------------------------------------------------------------------------------------------------------------------------------------------------------------------------------------------------------------------------------------------------------------------------------------------------------------------------------------------------------------------------------------------------------------------------------------------------------------------------------------------------------------------------------------------------------------------------------------------------------------------------------------------------------------------------------------------------------------------------------------------------------------------------------------------------------------------------------------------------------------------------------------------------------------------------------------------------------------------------------------------------------------------------------------------------------------------------------------------------------------------------------------------------------------------------------------------------------------------------------------------------------------------------------------------------------------------------------------------------------------------------------------------------------------------------------------------------------------------------------------------------------------------------------------------------------------------------------------------------------------------------------------------------------------------------------------------------------------------------------------------------------------------------------------------------------------------------------------------------------------------------------------------------------------------------------------Cornoberotha   00110000000?00000000010100000000001--00000111101000??001?1000202010(0 1)01210(1 3)0??0??0?????10??10?10201?????------------------------------------------------------------------------------------------------------------------------------------------------------------------------------------------------------------------------------------------------------------------------------------------------------------------------------------------------------------------------------------------------------------------------------------------------------------------------------------------------------------------------------------------------------------------------------------------------------------------------------------------------------------------------------------------------------------------------------------------------------------------------------------------------------------------------------------------------------------------------------------------------------------------------------------------------------------------------------------------------------------------------------------------------------------------------------------------------------------------------------------------------------------------------------------------------------------------------------------------------------------------------------------------------------------------------------------------------------------------------------------------------------------------------------------------------------------------------------------------------------------------------------------------------------------------------------------------------------------------------------------------------------------------------------------------------------------------------------------------------------------------------------------------------------------------------------------------------------------------------------------------------------------------------------------------------------------------------------------------------------------------------------------------------------------------------------------------------------------------------------------------------------------------------------------------------------------------------------------------------------------------------------------------------------------------------------------------------------------------------------------------------------------------------------------------------------------------------------------------------------------------------------------------------------------------------------------------------------------------------------------------------------------------------------------------------------------------------------------------------------------------------------------------------------------------------------------------------------------------------------------------------------------------------------------------------------------------------------------------------------------------------------------------------------------------------------------------------------------------------------------------------------------------------------------------------------------------------------------------------------------------------------------------------------------------------------------------------------------------------------------------------------------------------------------------------------------------------------------------------------------------------------------------------------------------------------------------------------------------------------------------------------------------------------------------------------------------------------------------------------------------------------------------------------------------------------------------------------------------------------------------------------------------------------------------------------------------------------------------------------------------------------------------------------------------------------------------------------------------------------------------------------------------------------------------------------------------------------------------------------------------------------------------------------------------------------------------------------------------------------------------------------------------------------------------------------------------------------------------------------------------------------------------------------------------------------------------------------------------------------------------------------------------------------------------------------------------------------------------------------------------------------------------------------------------------------------------------------------------------------------------------------------------------------------------------------------------------------------------------------------------------------------------------------------------------------------------------------------------------------------------------------------------------------------------------------------------------------------------------------------------------------------------------------------------------------------------------------------------------------------------------------------------------------------------------------------------------------------------------------------------------------------------------------------------------------------------------------------------------------------------------------------------------------------------------------------------------------------------------------------------------------------------------------------------------------------------------------------------------------------------------------------------------------------------------------------------------------------------------------------------------------------------------------------------------------------------------------------------------------------------------------Dasyberotha   0?1??000000?00?0?????????0010001001--010?0110111001??001?100??0???????????????????????????1??1??01?????------------------------------------------------------------------------------------------------------------------------------------------------------------------------------------------------------------------------------------------------------------------------------------------------------------------------------------------------------------------------------------------------------------------------------------------------------------------------------------------------------------------------------------------------------------------------------------------------------------------------------------------------------------------------------------------------------------------------------------------------------------------------------------------------------------------------------------------------------------------------------------------------------------------------------------------------------------------------------------------------------------------------------------------------------------------------------------------------------------------------------------------------------------------------------------------------------------------------------------------------------------------------------------------------------------------------------------------------------------------------------------------------------------------------------------------------------------------------------------------------------------------------------------------------------------------------------------------------------------------------------------------------------------------------------------------------------------------------------------------------------------------------------------------------------------------------------------------------------------------------------------------------------------------------------------------------------------------------------------------------------------------------------------------------------------------------------------------------------------------------------------------------------------------------------------------------------------------------------------------------------------------------------------------------------------------------------------------------------------------------------------------------------------------------------------------------------------------------------------------------------------------------------------------------------------------------------------------------------------------------------------------------------------------------------------------------------------------------------------------------------------------------------------------------------------------------------------------------------------------------------------------------------------------------------------------------------------------------------------------------------------------------------------------------------------------------------------------------------------------------------------------------------------------------------------------------------------------------------------------------------------------------------------------------------------------------------------------------------------------------------------------------------------------------------------------------------------------------------------------------------------------------------------------------------------------------------------------------------------------------------------------------------------------------------------------------------------------------------------------------------------------------------------------------------------------------------------------------------------------------------------------------------------------------------------------------------------------------------------------------------------------------------------------------------------------------------------------------------------------------------------------------------------------------------------------------------------------------------------------------------------------------------------------------------------------------------------------------------------------------------------------------------------------------------------------------------------------------------------------------------------------------------------------------------------------------------------------------------------------------------------------------------------------------------------------------------------------------------------------------------------------------------------------------------------------------------------------------------------------------------------------------------------------------------------------------------------------------------------------------------------------------------------------------------------------------------------------------------------------------------------------------------------------------------------------------------------------------------------------------------------------------------------------------------------------------------------------------------------------------------------------------------------------------------------------------------------------------------------------------------------------------------------------------------------------------------------------------------------------------------------------------------------------------------------------------------------------------------------------------------------------------------------------------------------------------------------------------------------------------------------------------------------------------------------------------------------------------------------------------------------------------------------------------------------------------------------------------------------------------------------------------------Dolichoberotha   001100000000000000000101?001011000010000?011110(0 1)000??001?1000(1 2)0100000(1 2)200101?0????????????10?00201?????------------------------------------------------------------------------------------------------------------------------------------------------------------------------------------------------------------------------------------------------------------------------------------------------------------------------------------------------------------------------------------------------------------------------------------------------------------------------------------------------------------------------------------------------------------------------------------------------------------------------------------------------------------------------------------------------------------------------------------------------------------------------------------------------------------------------------------------------------------------------------------------------------------------------------------------------------------------------------------------------------------------------------------------------------------------------------------------------------------------------------------------------------------------------------------------------------------------------------------------------------------------------------------------------------------------------------------------------------------------------------------------------------------------------------------------------------------------------------------------------------------------------------------------------------------------------------------------------------------------------------------------------------------------------------------------------------------------------------------------------------------------------------------------------------------------------------------------------------------------------------------------------------------------------------------------------------------------------------------------------------------------------------------------------------------------------------------------------------------------------------------------------------------------------------------------------------------------------------------------------------------------------------------------------------------------------------------------------------------------------------------------------------------------------------------------------------------------------------------------------------------------------------------------------------------------------------------------------------------------------------------------------------------------------------------------------------------------------------------------------------------------------------------------------------------------------------------------------------------------------------------------------------------------------------------------------------------------------------------------------------------------------------------------------------------------------------------------------------------------------------------------------------------------------------------------------------------------------------------------------------------------------------------------------------------------------------------------------------------------------------------------------------------------------------------------------------------------------------------------------------------------------------------------------------------------------------------------------------------------------------------------------------------------------------------------------------------------------------------------------------------------------------------------------------------------------------------------------------------------------------------------------------------------------------------------------------------------------------------------------------------------------------------------------------------------------------------------------------------------------------------------------------------------------------------------------------------------------------------------------------------------------------------------------------------------------------------------------------------------------------------------------------------------------------------------------------------------------------------------------------------------------------------------------------------------------------------------------------------------------------------------------------------------------------------------------------------------------------------------------------------------------------------------------------------------------------------------------------------------------------------------------------------------------------------------------------------------------------------------------------------------------------------------------------------------------------------------------------------------------------------------------------------------------------------------------------------------------------------------------------------------------------------------------------------------------------------------------------------------------------------------------------------------------------------------------------------------------------------------------------------------------------------------------------------------------------------------------------------------------------------------------------------------------------------------------------------------------------------------------------------------------------------------------------------------------------------------------------------------------------------------------------------------------------------------------------------------------------------------------------------------------------------------------------------------------------------------------------------------------------------------------------Haploberotha   011000000000000001000110?0000001001--1200011111101000001?0000002000100210111?0?????????????????????????------------------------------------------------------------------------------------------------------------------------------------------------------------------------------------------------------------------------------------------------------------------------------------------------------------------------------------------------------------------------------------------------------------------------------------------------------------------------------------------------------------------------------------------------------------------------------------------------------------------------------------------------------------------------------------------------------------------------------------------------------------------------------------------------------------------------------------------------------------------------------------------------------------------------------------------------------------------------------------------------------------------------------------------------------------------------------------------------------------------------------------------------------------------------------------------------------------------------------------------------------------------------------------------------------------------------------------------------------------------------------------------------------------------------------------------------------------------------------------------------------------------------------------------------------------------------------------------------------------------------------------------------------------------------------------------------------------------------------------------------------------------------------------------------------------------------------------------------------------------------------------------------------------------------------------------------------------------------------------------------------------------------------------------------------------------------------------------------------------------------------------------------------------------------------------------------------------------------------------------------------------------------------------------------------------------------------------------------------------------------------------------------------------------------------------------------------------------------------------------------------------------------------------------------------------------------------------------------------------------------------------------------------------------------------------------------------------------------------------------------------------------------------------------------------------------------------------------------------------------------------------------------------------------------------------------------------------------------------------------------------------------------------------------------------------------------------------------------------------------------------------------------------------------------------------------------------------------------------------------------------------------------------------------------------------------------------------------------------------------------------------------------------------------------------------------------------------------------------------------------------------------------------------------------------------------------------------------------------------------------------------------------------------------------------------------------------------------------------------------------------------------------------------------------------------------------------------------------------------------------------------------------------------------------------------------------------------------------------------------------------------------------------------------------------------------------------------------------------------------------------------------------------------------------------------------------------------------------------------------------------------------------------------------------------------------------------------------------------------------------------------------------------------------------------------------------------------------------------------------------------------------------------------------------------------------------------------------------------------------------------------------------------------------------------------------------------------------------------------------------------------------------------------------------------------------------------------------------------------------------------------------------------------------------------------------------------------------------------------------------------------------------------------------------------------------------------------------------------------------------------------------------------------------------------------------------------------------------------------------------------------------------------------------------------------------------------------------------------------------------------------------------------------------------------------------------------------------------------------------------------------------------------------------------------------------------------------------------------------------------------------------------------------------------------------------------------------------------------------------------------------------------------------------------------------------------------------------------------------------------------------------------------------------------------------------------------------------------------------------------------------------------------------------------------------------------------------------------------------------------------------------------------Protoberotha   001010000000000000000100?0000001001--0201011100101100?01?0011002000123-002????????????????0??0?011?????------------------------------------------------------------------------------------------------------------------------------------------------------------------------------------------------------------------------------------------------------------------------------------------------------------------------------------------------------------------------------------------------------------------------------------------------------------------------------------------------------------------------------------------------------------------------------------------------------------------------------------------------------------------------------------------------------------------------------------------------------------------------------------------------------------------------------------------------------------------------------------------------------------------------------------------------------------------------------------------------------------------------------------------------------------------------------------------------------------------------------------------------------------------------------------------------------------------------------------------------------------------------------------------------------------------------------------------------------------------------------------------------------------------------------------------------------------------------------------------------------------------------------------------------------------------------------------------------------------------------------------------------------------------------------------------------------------------------------------------------------------------------------------------------------------------------------------------------------------------------------------------------------------------------------------------------------------------------------------------------------------------------------------------------------------------------------------------------------------------------------------------------------------------------------------------------------------------------------------------------------------------------------------------------------------------------------------------------------------------------------------------------------------------------------------------------------------------------------------------------------------------------------------------------------------------------------------------------------------------------------------------------------------------------------------------------------------------------------------------------------------------------------------------------------------------------------------------------------------------------------------------------------------------------------------------------------------------------------------------------------------------------------------------------------------------------------------------------------------------------------------------------------------------------------------------------------------------------------------------------------------------------------------------------------------------------------------------------------------------------------------------------------------------------------------------------------------------------------------------------------------------------------------------------------------------------------------------------------------------------------------------------------------------------------------------------------------------------------------------------------------------------------------------------------------------------------------------------------------------------------------------------------------------------------------------------------------------------------------------------------------------------------------------------------------------------------------------------------------------------------------------------------------------------------------------------------------------------------------------------------------------------------------------------------------------------------------------------------------------------------------------------------------------------------------------------------------------------------------------------------------------------------------------------------------------------------------------------------------------------------------------------------------------------------------------------------------------------------------------------------------------------------------------------------------------------------------------------------------------------------------------------------------------------------------------------------------------------------------------------------------------------------------------------------------------------------------------------------------------------------------------------------------------------------------------------------------------------------------------------------------------------------------------------------------------------------------------------------------------------------------------------------------------------------------------------------------------------------------------------------------------------------------------------------------------------------------------------------------------------------------------------------------------------------------------------------------------------------------------------------------------------------------------------------------------------------------------------------------------------------------------------------------------------------------------------------------------------------------------------------------------------------------------------------------------------------------------------------------------------------------------------------Xiaoberotha   001000000000000000000111?0001001001--0100011001000000001?0000101000001010200?1??1?????????0?100001?????------------------------------------------------------------------------------------------------------------------------------------------------------------------------------------------------------------------------------------------------------------------------------------------------------------------------------------------------------------------------------------------------------------------------------------------------------------------------------------------------------------------------------------------------------------------------------------------------------------------------------------------------------------------------------------------------------------------------------------------------------------------------------------------------------------------------------------------------------------------------------------------------------------------------------------------------------------------------------------------------------------------------------------------------------------------------------------------------------------------------------------------------------------------------------------------------------------------------------------------------------------------------------------------------------------------------------------------------------------------------------------------------------------------------------------------------------------------------------------------------------------------------------------------------------------------------------------------------------------------------------------------------------------------------------------------------------------------------------------------------------------------------------------------------------------------------------------------------------------------------------------------------------------------------------------------------------------------------------------------------------------------------------------------------------------------------------------------------------------------------------------------------------------------------------------------------------------------------------------------------------------------------------------------------------------------------------------------------------------------------------------------------------------------------------------------------------------------------------------------------------------------------------------------------------------------------------------------------------------------------------------------------------------------------------------------------------------------------------------------------------------------------------------------------------------------------------------------------------------------------------------------------------------------------------------------------------------------------------------------------------------------------------------------------------------------------------------------------------------------------------------------------------------------------------------------------------------------------------------------------------------------------------------------------------------------------------------------------------------------------------------------------------------------------------------------------------------------------------------------------------------------------------------------------------------------------------------------------------------------------------------------------------------------------------------------------------------------------------------------------------------------------------------------------------------------------------------------------------------------------------------------------------------------------------------------------------------------------------------------------------------------------------------------------------------------------------------------------------------------------------------------------------------------------------------------------------------------------------------------------------------------------------------------------------------------------------------------------------------------------------------------------------------------------------------------------------------------------------------------------------------------------------------------------------------------------------------------------------------------------------------------------------------------------------------------------------------------------------------------------------------------------------------------------------------------------------------------------------------------------------------------------------------------------------------------------------------------------------------------------------------------------------------------------------------------------------------------------------------------------------------------------------------------------------------------------------------------------------------------------------------------------------------------------------------------------------------------------------------------------------------------------------------------------------------------------------------------------------------------------------------------------------------------------------------------------------------------------------------------------------------------------------------------------------------------------------------------------------------------------------------------------------------------------------------------------------------------------------------------------------------------------------------------------------------------------------------------------------------------------------------------------------------------------------------------------------------------------------------------------------------------------------Jersiberotha   0010?0000000000000000110?0010001001--010101110110(0 1)0?0001?0000002000123--0?????????????00?????1???1?????------------------------------------------------------------------------------------------------------------------------------------------------------------------------------------------------------------------------------------------------------------------------------------------------------------------------------------------------------------------------------------------------------------------------------------------------------------------------------------------------------------------------------------------------------------------------------------------------------------------------------------------------------------------------------------------------------------------------------------------------------------------------------------------------------------------------------------------------------------------------------------------------------------------------------------------------------------------------------------------------------------------------------------------------------------------------------------------------------------------------------------------------------------------------------------------------------------------------------------------------------------------------------------------------------------------------------------------------------------------------------------------------------------------------------------------------------------------------------------------------------------------------------------------------------------------------------------------------------------------------------------------------------------------------------------------------------------------------------------------------------------------------------------------------------------------------------------------------------------------------------------------------------------------------------------------------------------------------------------------------------------------------------------------------------------------------------------------------------------------------------------------------------------------------------------------------------------------------------------------------------------------------------------------------------------------------------------------------------------------------------------------------------------------------------------------------------------------------------------------------------------------------------------------------------------------------------------------------------------------------------------------------------------------------------------------------------------------------------------------------------------------------------------------------------------------------------------------------------------------------------------------------------------------------------------------------------------------------------------------------------------------------------------------------------------------------------------------------------------------------------------------------------------------------------------------------------------------------------------------------------------------------------------------------------------------------------------------------------------------------------------------------------------------------------------------------------------------------------------------------------------------------------------------------------------------------------------------------------------------------------------------------------------------------------------------------------------------------------------------------------------------------------------------------------------------------------------------------------------------------------------------------------------------------------------------------------------------------------------------------------------------------------------------------------------------------------------------------------------------------------------------------------------------------------------------------------------------------------------------------------------------------------------------------------------------------------------------------------------------------------------------------------------------------------------------------------------------------------------------------------------------------------------------------------------------------------------------------------------------------------------------------------------------------------------------------------------------------------------------------------------------------------------------------------------------------------------------------------------------------------------------------------------------------------------------------------------------------------------------------------------------------------------------------------------------------------------------------------------------------------------------------------------------------------------------------------------------------------------------------------------------------------------------------------------------------------------------------------------------------------------------------------------------------------------------------------------------------------------------------------------------------------------------------------------------------------------------------------------------------------------------------------------------------------------------------------------------------------------------------------------------------------------------------------------------------------------------------------------------------------------------------------------------------------------------------------------------------------------------------------------------------------------------------------------------------------------------------------------------------------------------------------Nascimberotha   0????0000?0?0??0?0?0?????0001100001--00000201001000??001?100020?1010?1?????????????????????????????????------------------------------------------------------------------------------------------------------------------------------------------------------------------------------------------------------------------------------------------------------------------------------------------------------------------------------------------------------------------------------------------------------------------------------------------------------------------------------------------------------------------------------------------------------------------------------------------------------------------------------------------------------------------------------------------------------------------------------------------------------------------------------------------------------------------------------------------------------------------------------------------------------------------------------------------------------------------------------------------------------------------------------------------------------------------------------------------------------------------------------------------------------------------------------------------------------------------------------------------------------------------------------------------------------------------------------------------------------------------------------------------------------------------------------------------------------------------------------------------------------------------------------------------------------------------------------------------------------------------------------------------------------------------------------------------------------------------------------------------------------------------------------------------------------------------------------------------------------------------------------------------------------------------------------------------------------------------------------------------------------------------------------------------------------------------------------------------------------------------------------------------------------------------------------------------------------------------------------------------------------------------------------------------------------------------------------------------------------------------------------------------------------------------------------------------------------------------------------------------------------------------------------------------------------------------------------------------------------------------------------------------------------------------------------------------------------------------------------------------------------------------------------------------------------------------------------------------------------------------------------------------------------------------------------------------------------------------------------------------------------------------------------------------------------------------------------------------------------------------------------------------------------------------------------------------------------------------------------------------------------------------------------------------------------------------------------------------------------------------------------------------------------------------------------------------------------------------------------------------------------------------------------------------------------------------------------------------------------------------------------------------------------------------------------------------------------------------------------------------------------------------------------------------------------------------------------------------------------------------------------------------------------------------------------------------------------------------------------------------------------------------------------------------------------------------------------------------------------------------------------------------------------------------------------------------------------------------------------------------------------------------------------------------------------------------------------------------------------------------------------------------------------------------------------------------------------------------------------------------------------------------------------------------------------------------------------------------------------------------------------------------------------------------------------------------------------------------------------------------------------------------------------------------------------------------------------------------------------------------------------------------------------------------------------------------------------------------------------------------------------------------------------------------------------------------------------------------------------------------------------------------------------------------------------------------------------------------------------------------------------------------------------------------------------------------------------------------------------------------------------------------------------------------------------------------------------------------------------------------------------------------------------------------------------------------------------------------------------------------------------------------------------------------------------------------------------------------------------------------------------------------------------------------------------------------------------------------------------------------------------------------------------------------------------------------------------------------------------------------------------------------------------------------------------------------------------------------------------------------------------------------------------Plesiorobius   1?1010??0000000000000?00?0001201001--120?0210101001???01?100??0???????????00?1?????????????????????????------------------------------------------------------------------------------------------------------------------------------------------------------------------------------------------------------------------------------------------------------------------------------------------------------------------------------------------------------------------------------------------------------------------------------------------------------------------------------------------------------------------------------------------------------------------------------------------------------------------------------------------------------------------------------------------------------------------------------------------------------------------------------------------------------------------------------------------------------------------------------------------------------------------------------------------------------------------------------------------------------------------------------------------------------------------------------------------------------------------------------------------------------------------------------------------------------------------------------------------------------------------------------------------------------------------------------------------------------------------------------------------------------------------------------------------------------------------------------------------------------------------------------------------------------------------------------------------------------------------------------------------------------------------------------------------------------------------------------------------------------------------------------------------------------------------------------------------------------------------------------------------------------------------------------------------------------------------------------------------------------------------------------------------------------------------------------------------------------------------------------------------------------------------------------------------------------------------------------------------------------------------------------------------------------------------------------------------------------------------------------------------------------------------------------------------------------------------------------------------------------------------------------------------------------------------------------------------------------------------------------------------------------------------------------------------------------------------------------------------------------------------------------------------------------------------------------------------------------------------------------------------------------------------------------------------------------------------------------------------------------------------------------------------------------------------------------------------------------------------------------------------------------------------------------------------------------------------------------------------------------------------------------------------------------------------------------------------------------------------------------------------------------------------------------------------------------------------------------------------------------------------------------------------------------------------------------------------------------------------------------------------------------------------------------------------------------------------------------------------------------------------------------------------------------------------------------------------------------------------------------------------------------------------------------------------------------------------------------------------------------------------------------------------------------------------------------------------------------------------------------------------------------------------------------------------------------------------------------------------------------------------------------------------------------------------------------------------------------------------------------------------------------------------------------------------------------------------------------------------------------------------------------------------------------------------------------------------------------------------------------------------------------------------------------------------------------------------------------------------------------------------------------------------------------------------------------------------------------------------------------------------------------------------------------------------------------------------------------------------------------------------------------------------------------------------------------------------------------------------------------------------------------------------------------------------------------------------------------------------------------------------------------------------------------------------------------------------------------------------------------------------------------------------------------------------------------------------------------------------------------------------------------------------------------------------------------------------------------------------------------------------------------------------------------------------------------------------------------------------------------------------------------------------------------------------------------------------------------------------------------------------------------------------------------------------------------------------------------------------------------------------------------------------------------------------------------------------------------------------------------------------------------Microberotha   011000000000000000000110?00100001101?02010111100010??001???11001000123--1011?0??????10?????????????????------------------------------------------------------------------------------------------------------------------------------------------------------------------------------------------------------------------------------------------------------------------------------------------------------------------------------------------------------------------------------------------------------------------------------------------------------------------------------------------------------------------------------------------------------------------------------------------------------------------------------------------------------------------------------------------------------------------------------------------------------------------------------------------------------------------------------------------------------------------------------------------------------------------------------------------------------------------------------------------------------------------------------------------------------------------------------------------------------------------------------------------------------------------------------------------------------------------------------------------------------------------------------------------------------------------------------------------------------------------------------------------------------------------------------------------------------------------------------------------------------------------------------------------------------------------------------------------------------------------------------------------------------------------------------------------------------------------------------------------------------------------------------------------------------------------------------------------------------------------------------------------------------------------------------------------------------------------------------------------------------------------------------------------------------------------------------------------------------------------------------------------------------------------------------------------------------------------------------------------------------------------------------------------------------------------------------------------------------------------------------------------------------------------------------------------------------------------------------------------------------------------------------------------------------------------------------------------------------------------------------------------------------------------------------------------------------------------------------------------------------------------------------------------------------------------------------------------------------------------------------------------------------------------------------------------------------------------------------------------------------------------------------------------------------------------------------------------------------------------------------------------------------------------------------------------------------------------------------------------------------------------------------------------------------------------------------------------------------------------------------------------------------------------------------------------------------------------------------------------------------------------------------------------------------------------------------------------------------------------------------------------------------------------------------------------------------------------------------------------------------------------------------------------------------------------------------------------------------------------------------------------------------------------------------------------------------------------------------------------------------------------------------------------------------------------------------------------------------------------------------------------------------------------------------------------------------------------------------------------------------------------------------------------------------------------------------------------------------------------------------------------------------------------------------------------------------------------------------------------------------------------------------------------------------------------------------------------------------------------------------------------------------------------------------------------------------------------------------------------------------------------------------------------------------------------------------------------------------------------------------------------------------------------------------------------------------------------------------------------------------------------------------------------------------------------------------------------------------------------------------------------------------------------------------------------------------------------------------------------------------------------------------------------------------------------------------------------------------------------------------------------------------------------------------------------------------------------------------------------------------------------------------------------------------------------------------------------------------------------------------------------------------------------------------------------------------------------------------------------------------------------------------------------------------------------------------------------------------------------------------------------------------------------------------------------------------------------------------------------------------------------------------------------------------------------------------------------------------------------------------------------------------Xenoberotha   0??0?0?00???0????????????001?0?0?0???0100011110?0?????01???????????????????????????????????????????????------------------------------------------------------------------------------------------------------------------------------------------------------------------------------------------------------------------------------------------------------------------------------------------------------------------------------------------------------------------------------------------------------------------------------------------------------------------------------------------------------------------------------------------------------------------------------------------------------------------------------------------------------------------------------------------------------------------------------------------------------------------------------------------------------------------------------------------------------------------------------------------------------------------------------------------------------------------------------------------------------------------------------------------------------------------------------------------------------------------------------------------------------------------------------------------------------------------------------------------------------------------------------------------------------------------------------------------------------------------------------------------------------------------------------------------------------------------------------------------------------------------------------------------------------------------------------------------------------------------------------------------------------------------------------------------------------------------------------------------------------------------------------------------------------------------------------------------------------------------------------------------------------------------------------------------------------------------------------------------------------------------------------------------------------------------------------------------------------------------------------------------------------------------------------------------------------------------------------------------------------------------------------------------------------------------------------------------------------------------------------------------------------------------------------------------------------------------------------------------------------------------------------------------------------------------------------------------------------------------------------------------------------------------------------------------------------------------------------------------------------------------------------------------------------------------------------------------------------------------------------------------------------------------------------------------------------------------------------------------------------------------------------------------------------------------------------------------------------------------------------------------------------------------------------------------------------------------------------------------------------------------------------------------------------------------------------------------------------------------------------------------------------------------------------------------------------------------------------------------------------------------------------------------------------------------------------------------------------------------------------------------------------------------------------------------------------------------------------------------------------------------------------------------------------------------------------------------------------------------------------------------------------------------------------------------------------------------------------------------------------------------------------------------------------------------------------------------------------------------------------------------------------------------------------------------------------------------------------------------------------------------------------------------------------------------------------------------------------------------------------------------------------------------------------------------------------------------------------------------------------------------------------------------------------------------------------------------------------------------------------------------------------------------------------------------------------------------------------------------------------------------------------------------------------------------------------------------------------------------------------------------------------------------------------------------------------------------------------------------------------------------------------------------------------------------------------------------------------------------------------------------------------------------------------------------------------------------------------------------------------------------------------------------------------------------------------------------------------------------------------------------------------------------------------------------------------------------------------------------------------------------------------------------------------------------------------------------------------------------------------------------------------------------------------------------------------------------------------------------------------------------------------------------------------------------------------------------------------------------------------------------------------------------------------------------------------------------------------------------------------------------------------------------------------------------------------------------------------------------------------------------------------Elektroberotha   ?0?0?100000000000100011?10001111001--00100110111000?00011000?201?000010102????????????102?1??10211?????------------------------------------------------------------------------------------------------------------------------------------------------------------------------------------------------------------------------------------------------------------------------------------------------------------------------------------------------------------------------------------------------------------------------------------------------------------------------------------------------------------------------------------------------------------------------------------------------------------------------------------------------------------------------------------------------------------------------------------------------------------------------------------------------------------------------------------------------------------------------------------------------------------------------------------------------------------------------------------------------------------------------------------------------------------------------------------------------------------------------------------------------------------------------------------------------------------------------------------------------------------------------------------------------------------------------------------------------------------------------------------------------------------------------------------------------------------------------------------------------------------------------------------------------------------------------------------------------------------------------------------------------------------------------------------------------------------------------------------------------------------------------------------------------------------------------------------------------------------------------------------------------------------------------------------------------------------------------------------------------------------------------------------------------------------------------------------------------------------------------------------------------------------------------------------------------------------------------------------------------------------------------------------------------------------------------------------------------------------------------------------------------------------------------------------------------------------------------------------------------------------------------------------------------------------------------------------------------------------------------------------------------------------------------------------------------------------------------------------------------------------------------------------------------------------------------------------------------------------------------------------------------------------------------------------------------------------------------------------------------------------------------------------------------------------------------------------------------------------------------------------------------------------------------------------------------------------------------------------------------------------------------------------------------------------------------------------------------------------------------------------------------------------------------------------------------------------------------------------------------------------------------------------------------------------------------------------------------------------------------------------------------------------------------------------------------------------------------------------------------------------------------------------------------------------------------------------------------------------------------------------------------------------------------------------------------------------------------------------------------------------------------------------------------------------------------------------------------------------------------------------------------------------------------------------------------------------------------------------------------------------------------------------------------------------------------------------------------------------------------------------------------------------------------------------------------------------------------------------------------------------------------------------------------------------------------------------------------------------------------------------------------------------------------------------------------------------------------------------------------------------------------------------------------------------------------------------------------------------------------------------------------------------------------------------------------------------------------------------------------------------------------------------------------------------------------------------------------------------------------------------------------------------------------------------------------------------------------------------------------------------------------------------------------------------------------------------------------------------------------------------------------------------------------------------------------------------------------------------------------------------------------------------------------------------------------------------------------------------------------------------------------------------------------------------------------------------------------------------------------------------------------------------------------------------------------------------------------------------------------------------------------------------------------------------------------------------------------------------------------------------------------------------------------------------------------------------------------------------------------------------------------------;END;begin mrbayes;	set autoclose = no nowarn = no;        lset coding = variable rates = gamma;	        calibrate Sinosmylites = unif ( 145 , 168.3 ) Berothone = unif ( 158 , 164 ) Krokhathone = unif ( 158 , 164 ) Mesithone = unif ( 129.4 , 164 ) Epimesoberotha = unif ( 140.2 , 145 ) Pseudosisyra = unif ( 129.4 , 131 ) Oloberotha = unif ( 125 , 129.4 ) Sibelliberotha = unif ( 125.5 , 129.4 ) Araripeberotha = unif ( 113.2 , 121.4 ) Caririberotha = unif ( 113.2 , 121.4 ) Cantabroberotha = unif ( 108.8 , 113 ) Aggregataberotha = unif ( 98.17 , 99.41 ) Ansoberotha = unif ( 98.17 , 99.41 ) Cornoberotha = unif ( 98.17 , 99.41 ) Dasyberotha = unif ( 98.17 , 99.41 ) Dolichoberotha = unif ( 98.17 , 99.41 ) Haploberotha = unif ( 98.17 , 99.41 ) Protoberotha = unif ( 98.17 , 99.41 ) Xiaoberotha = unif ( 98.17 , 99.41 ) Jersiberotha = unif ( 89.8 , 99.41 ) Nascimberotha = unif ( 89.8 , 93.9 ) Plesiorobius = unif ( 78 , 84 ) Microberotha = unif ( 47.8 , 56 ) Xenoberotha = unif ( 48.5 , 53.5 ) Elektroberotha = unif ( 33.9 , 37.71 );	        prset nodeagepr = calibrated;	prset brlenspr = clock : fossilization;	prset speciationpr = exp ( 100 );	prset extinctionpr = beta ( 1 , 1 );	prset fossilizationpr = beta ( 1 , 1 );	prset sampleprob = 0.0425;	prset treeagepr = offsetgamma ( 163.5, 165.9, 2.4 );	prset clockratepr = gamma ( 2 , 100 );	prset clockvarpr = igr;	prset igrvarpr = exp ( 10 );	propset NodesliderClock$prob = 40;	propset AddBranch $prob = 0;	propset DelBranch $prob = 0;	constraint ingroup = 4-.;	prset topologypr = constraint(ingroup);	mcmcp nrun = 2 nchain = 4 ngen = 80000000 samplefr = 2000 printfr = 40000 diagnfr = 400000 temp = 0.04 stopval = 0.005 stoprule = yes;	mcmc;	sump;	sumt relburnin = yes burninfrac = 0.25 contype = allcompat Outputname = Bero_c6013t53_DNA+morph_1109_alcp;	sumt relburnin = yes burninfrac = 0.25 contype = halfcompat Outputname = Bero_c6013t53_DNA+morph_1109_halfcp;end;
