# Supplementary figures and images for: Dynamic interplay between niche variation and flight adaptability drove a hundred million years’ dispersion in iconic lacewings
Source: Proc Natl Acad Sci U S A. 2025 May 2;122(19):e2414549122. doi: 10.1073/pnas.2414549122 (PMC12087969; doi:10.1073/pnas.2414549122)

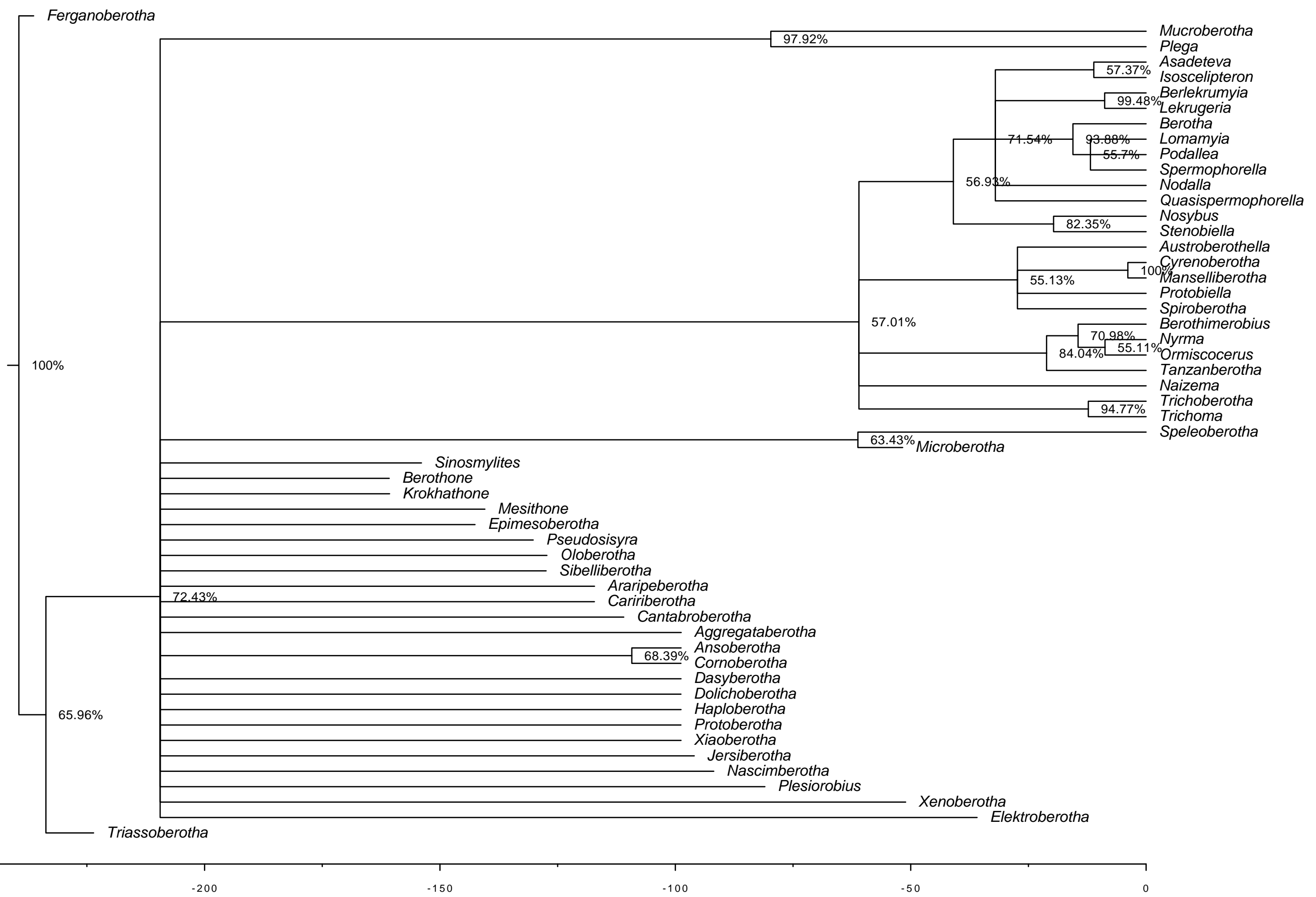

Supplement: Supplementary file 10 — Dataset S09 (PDF) [file pnas.2414549122.sd09.pdf]
